# Modifiable Dietary Habits, Inflammatory Mediation, and Senile Cataract: Unraveling Causality via Mendelian Randomization

**DOI:** 10.1002/fsn3.71366

**Published:** 2025-12-17

**Authors:** Jian Deng, Liduo Pan, Jiangfeng Huang, Jianli Yang, Meng Wang, Tao Wang, Yonghao Li

**Affiliations:** ^1^ Department of Ophthalmology Aier Eye Hospital, Jinan University Guangzhou People's Republic of China; ^2^ Department of Dermatology The Third Xiangya Hospital of Central South University Changsha People's Republic of China; ^3^ Department of Ophthalmology The Third Affiliated Hospital of Sun Yat‐Sen University Guangzhou People's Republic of China; ^4^ Department of Ophthalmology, Shenzhen Eye Hospital, Shenzhen Eye Medical Center Southern Medical University Shenzhen People's Republic of China

**Keywords:** inflammation factors, mendelian randomization, modifiable dietary habits, population aging, senile cataract

## Abstract

Senile cataract (SC) is a major cause of global vision impairment and blindness, posing a significant public health challenge in aging populations. This study employed Mendelian randomization (MR) using genetic instruments for 83 dietary habits from the UK Biobank GWAS, with SC data from the FinnGen R12 as the outcome. We employed multiple MR methods, including bidirectional MR, replication sample MR, and multivariable MR (MVMR), to identify dietary habits significantly associated with SC. The potential mediating effects of inflammatory biomarkers were further assessed using a two‐step MR. Finally, to validate the core findings, additional analyses employed independent datasets for the overall cataract phenotype and the cataract surgical endpoint. After false discovery rate correction and validation in an independent cohort, meal‐aligned alcohol consumption, compared to drinking apart from meals, demonstrated a substantial and robust protective causal effect against SC (OR = 0.67; 95% CI = 0.55–0.82; *p* = 5.86 × 10^−5^). MVMR sustained this independent protective effect after adjusting for 15 outcome‐related confounders. Mediation analysis indicated partial mediation through inflammatory pathways, with IL‐6 accounting for 11% and CRP for 6% of the effect. Further analyses using independent datasets also linked this drinking pattern to a reduced likelihood of undergoing cataract surgery (OR = 0.99; 95% CI = 0.98–0.99; *p* = 2.34 × 10^−6^) and a lower risk of overall cataract (OR = 0.83; 95% CI = 0.76–0.91; *p* = 5.26 × 10^−5^). This study reveals that, compared to drinking without meals, consuming alcohol with meals causally reduces SC risk among current drinkers, partially mediated through inflammatory pathways. These findings suggest that alcohol‐related nutritional strategies for SC should therefore shift focus from solely type and quantity to include consumption timing.

## Graphical abstract

This Mendelian randomization study indicates that specific drinking habits influence the risk of senile cataract (SC) through inflammatory modulation. Genetic analyses revealed that, compared to consuming alcohol apart from meals, alcohol intake aligned with meals reduces the risk of SC among current drinkers, partially through lowered inflammation levels (mediated in part by IL‐6 and CRP). These findings suggest that alcohol‐related nutritional strategies for SC should therefore focus not only on the type and amount of alcohol consumed but also on the dimension of drinking timing.

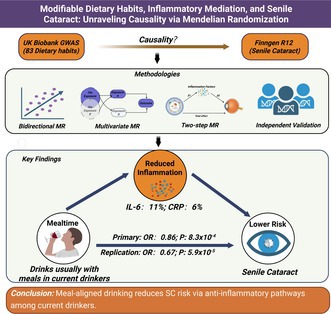

## 1 Introduction

Cataracts refer to the opacification of the lens that obstructs light transmission and leads to significant visual impairment, representing the primary cause of vision loss and blindness among the elderly globally (GBD 2019 Blindness and Vision Impairment Collaborators and Vision Loss Expert Group of the Global Burden of Disease Study 2021). The World Health Organization estimates that around 180 million people worldwide are visually impaired, with cataracts accounting for 46% of these cases, posing a substantial public health burden (Asbell et al. 2005; Bourne et al. 2013). Senile cataracts (SC), or age‐related cataracts, are the most common type among adults. In 2020, approximately 13.4 million people were blind due to SC, constituting 34.8% of all blindness cases (GBD 2019 Blindness and Vision Impairment Collaborators and Vision Loss Expert Group of the Global Burden of Disease Study 2021). The development of SC is associated with multiple factors, including aging, genetics, nutritional deficiencies, metabolic and immune abnormalities, and environmental influences, which collectively contribute to lens metabolic dysfunction (Asbell et al. 2005). Despite advancements in cataract surgery techniques and the introduction of various artificial lenses, SC continues to pose a substantial disease burden and economic strain, particularly in developing countries where surgical resources and qualified ophthalmologists are limited (He et al. 2017; Yan et al. 2019). Surgery is regarded as the sole curative treatment for this disease; however, it presents notable limitations, including variability in efficacy among individuals, heightened contraindications, risks of complications, and substantial costs (Lee and Afshari 2023; Priyadarshini et al. 2023). Consequently, due to the challenges associated with the rising incidence rate, complex etiology, and the limitations of surgical treatment in addressing all patient needs, it is essential to identify the risk factors for SC to inform prevention strategies and mitigate its progression.

Diet is a key modifiable lifestyle factor. Numerous observational studies have suggested that dietary habits may influence SC risk (Falkowska et al. 2023; Zhou et al. 2022). For instance, high‐sugar, high‐fat, and pro‐inflammatory diets may promote cataract formation through mechanisms such as advanced glycation end products (AGEs), oxidative stress, and chronic inflammation. High glucose exposure can prolong lens protein damage, leading to aggregation and opacity (Franke et al. 2003; Ramos‐Martínez et al. 2021). High‐fat diet consumption can result in lipid peroxidation, which induces oxidative stress in lens epithelial cells, thereby causing cellular damage and elevating the risk of lens opacification (Lu et al. 2005). Pro‐inflammatory diets may elevate markers like IL‐6 and CRP, promoting lens epithelial changes and cataract formation (Shivappa et al. 2017; Wu et al. 2018). Some studies, such as that by Camacho‐Barcia et al., suggest protective effects of yogurt consumption (Camacho‐Barcia et al. 2019), whereas others, like Tan et al., found no significant link between whole grains and SC (Tan et al. 2020). However, observational studies are limited by potential reverse causality and confounding factors. Broader, genetically informed approaches are needed to evaluate the role of dietary patterns in SC systematically.

Mendelian randomization (MR) serves as a robust approach for investigating the presumed causal link between various dietary habits and SC. Single‐nucleotide polymorphisms (SNPs) associated with exposure serve as instrumental variables (IVs) to examine the causal relationship between exposure (dietary habits) and outcome (SC) (Smith and Ebrahim 2003). MR exploits the random allocation of genetic variants during meiosis, offering a “natural” randomized controlled trial (RCT) to infer causality. The natural random allocation mechanism of genetic variation prior to exposure effectively avoids confounding bias and reverse causality interference (Davies et al. 2018). MR offers robust evidence supporting causal relationships among variables.

This study employed MR and extensive genome‐wide association studies (GWAS) to elucidate the causal relationship between prevalent dietary habits and the risk of SC, offering scientific strategies for the prevention of modifiable risk factors associated with SC.

## 2 Methods

### 2.1 Study Design

**FIGURE 1:**
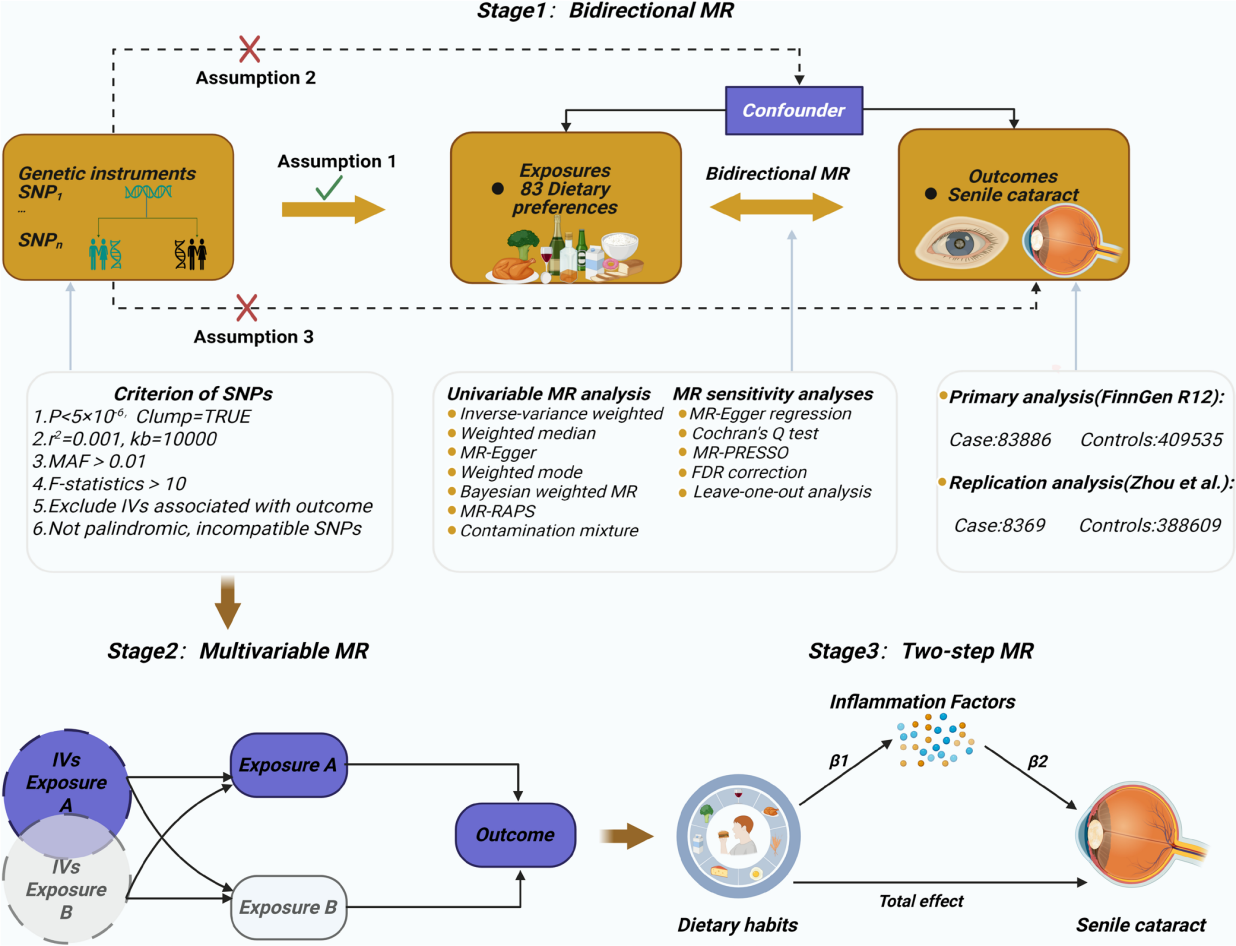
An overview of the study design. FDR, false discovery rate; IVs, instrumental variables; MAF, minor allele frequency; MR, mendelian randomization; RAPS, robust adjusted profile score; SNPs, single‐nucleotide polymorphisms.

This research employed the MR method utilizing publicly accessible GWAS data to integrate dietary habits with SC genetic information. We systematically investigated the causal relationship between dietary habits and the incidence risk of SC, as well as its potential mechanisms, using two‐sample MR, multivariate MR, and two‐step MR methods. As shown in Figure 1. To ensure the reliability of MR analysis, the study adhered to three core assumptions: first, instrumental variables (IVs) must be significantly correlated with the exposure factor (correlation assumption); second, IVs must be independent of confounding factors (independence assumption); and third, IVs must influence the outcome variable solely through the target exposure (exclusivity assumption). Furthermore, to ensure methodological quality control during the process, this study followed the Strengthening the Reporting of Observational Studies in Epidemiology (STROBE) guidelines (Skrivankova et al. 2021) (Data S3).

### 2.2 Data Sources

To minimize confounding and ensure robust results, all exposure and outcome data were derived exclusively from European‐ancestry populations with no sample overlap. Dietary habits data were sourced from the official website of the Type 2 Diabetes Knowledge Portal (http://www.kp4cd.org/dataset_downloads/t2d), as provided by Cole et al., using GWAS results on 83 dietary habits collected from food frequency questionnaires completed by 449,210 individuals across Europe (Cole et al. 2020) (Table S1). Table S2 presents additional details on the questionnaires. The GWAS data for SC were sourced from the FinnGen consortium (https://r12.finngen.fi/), comprising 83,886 cases and 409,535 controls. Simultaneously, we utilized GWAS data for SC, as summarized by Zhou et al. (2018), to validate the prior MR analysis findings. Aggregate statistical data utilized as confounding variables in multivariate Mendelian Randomization analysis are accessible on the official website of the Integrated Epidemiological Units (IEU) Open GWAS Project (https://gwas.mrcieu.ac.uk/). The data encompass dysglycemia phenotypes (Hemoglobin A1c, Fasting glucose, Two‐hour glucose, Type 2 diabetes), adiposity phenotypes (Body Mass Index, Waist‐to‐hip ratio, Body fat percentage), blood pressure dysregulation phenotype (Systolic Blood Pressure, Diastolic Blood Pressure, Hypertension), dyslipidemia phenotypes (Low‐Density Lipoprotein, High‐Density Lipoprotein, Triglycerides), and lifestyle habits and environmental factors (Smoking status, Educational attainment). Furthermore, to investigate the mediating effect of dietary habits on SC, we concentrated on 12 inflammatory markers. These markers were chosen based on their established associations with both dietary factors and cataract formation, as identified in existing GWAS datasets and prior literature (Ma et al. 2022; Shivappa et al. 2017; Vergroesen et al. 2023). The specific markers include: C‐Reactive Protein (CRP), Fibroblast Growth Factor 23 (FBG‐23), FBG Basic, Monocyte Chemoattractant Protein‐1 (MCP‐1), Platelet‐Derived Growth Factor BB (PDGFBB), Tumor Necrosis Factor Alpha (TNF‐α), TNF‐Related Apoptosis‐Inducing Ligand, Vascular Endothelial Growth Factor (VEGF), Interleukin‐6 (IL‐6), IL‐10, IL‐16, and Interleukin‐1 Receptor Antagonist (IL‐1Ra). To strengthen the validity of significant findings in SC, we conducted further analysis using summarized GWAS data from Boutin et al. (2020) on Cataracts operation and Sakaue et al. (2021) on Cataracts. Comprehensive details on the GWAS summary datasets are presented in Table S1.

### 2.3 Instrumental Variable Selection

To ensure the accuracy and authenticity of the study results, the following criteria were employed to identify the instrumental variables (Nazarian et al. 2019; Su, Li, et al. 2025). First, SNPs associated with dietary habits were selected based on a GWAS threshold of *p* < 5 × 10^−6^ and subjected to linkage disequilibrium clumping with an *r* ^2^ < 0.001 and clumping distance of 10,000 kb. Second, SNPs with minor allele frequencies (MAF) < 0.01 were excluded to maintain population representativeness. Third, we retained only SNPs with high instrumental variable strength, requiring *F*‐statistic values to be ≥ 10 (where *F* = *β*_exposure^2^/SE_exposure^2^) (Burgess and Thompson 2011). Fourth, using the FastTraitR method (Wang et al. 2025), we strictly excluded SNPs significantly associated with SC outcomes or other potential confounding phenotypes (e.g., age, myopia, axial length, diabetes, smoking) with *p* < 5 × 10^−5^. Finally, we excluded all palindromic SNPs to avoid uncertainty in genotype chain direction and applied MR pleiotropy residuals and outlier (MR‐PRESSO) tests to identify and remove abnormal SNPs with significant pleiotropy.

### 2.4 Bidirectional MR Analyses

Initially, a bidirectional MR analysis was performed to evaluate the relationship between dietary habits and SC. The inverse‐variance weighted (IVW) method served as the primary analytical approach, supplemented by additional methods including MR‐Egger, weighted median, weighted mode, Bayesian weighted MR (BWMR), robust adjusted profile score (RAPS), and contamination mixture (ConMix) (Lawlor et al. 2008). The MR analysis's effect estimates are presented as odds ratios (ORs) together with the associated 95% CIs. The advantage of the IVW method lies in its ability to account for heterogeneity among instrumental variable SNPs and to deliver reliable causal estimates even in the presence of directional pleiotropy (Lawlor et al. 2008; Verbanck et al. 2018). However, recognizing that IVW results can be influenced by weak instruments and pleiotropy, we applied a suite of complementary MR analysis methods to evaluate the genetic causality between dietary habits and SC from multiple angles, thereby enhancing the accuracy and robustness of our findings. For detailed descriptions of additional MR methods, please refer to Data S4. Then, reverse MR was performed on key exposures to rule out reverse causation, using the same method as forward MR but with the exposure and outcome swapped (Su, Lu, et al. 2025). Moreover, positive results were further validated using GWAS summary data from the replication sample as the outcome.

### 2.5 Multivariable MR Analyses

Dietary habits with significant correlations in both primary and replicated bidirectional MR analyses were selected for MVMR validation to assess the independence of causal effects from potential confounders. Hyperglycemia is a key covariate, as it impairs ocular health and increases cataract risk by inducing oxidative stress and accumulating AGEs (Meng et al. 2024). Hypertension also drives cataract progression, as it elevates inflammatory factors (Yu et al. 2014). Additionally, obesity and elevated serum lipids are recognized covariates associated with a higher incidence of cataracts (Bosello et al. 2024; Jee and Park 2021; Li et al. 2018). Beyond these physiological factors, environmental and behavioral elements contribute, including genetic susceptibility to smoking (Nordström et al. 2025). Lower educational attainment, which often correlates with poorer dietary habits, further increases cataract susceptibility (Nam et al. 2015). The primary method employed was IVW, supported by auxiliary techniques, including MR‐Egger, weighted median, MR‐Lasso, and MR‐Robust, in the MVMR analysis. Heterogeneity was assessed using Cochran's Q statistic, and pleiotropy was evaluated with the MVMR‐Egger intercept test.

### 2.6 Two‐Step MR Analyses

A two‐step MR framework was employed to investigate whether 12 inflammatory markers, closely associated with diet and cataracts, mediate the relationship between dietary habits and SC, elucidating causal links identified in prior analyses.

### 2.7 Sensitivity Analysis

To mitigate the impact of heterogeneity and pleiotropy in MR analysis, several sensitivity tests were conducted. Specifically, Cochran's Q test indicates heterogeneity when the *p*‐value < 0.05, a condition that is acceptable if IVW with random effects is the primary outcome (Greco et al. 2015). Funnel plots were generated to examine potential directional pleiotropy, and MR‐Egger intercept tests were utilized to evaluate horizontal pleiotropy. Additionally, leave‐one‐out analysis was conducted to evaluate whether the MR estimates were influenced by a single SNP or subject to bias. Potential pleiotropy and outliers were assessed using MR‐PRESSO; a global test was used to detect the presence of horizontal pleiotropy and outliers in the instrumental variables. If outliers existed, the MR analysis was repeated after eliminating these outliers (Verbanck et al. 2018). False discovery rate (FDR) correction was applied for multiple testing, with *p* < 0.05 and *q* < 0.1 indicating a significant causal association, while *p* < 0.05 and *q* > 0.1 suggesting a potential causal effect (Chen et al. 2023). This approach ensures robust statistical evaluation and provides a reliable basis for understanding the complex relationships between dietary habits and SC.

Finally, to validate the robustness of the core findings, we conducted additional analysis utilizing summarized GWAS data from Zhou et al. (2018) regarding Cataract operations and Sakaue et al. (2021) on Cataracts.

### 2.8 Statistics Analysis

Statistical analyses were performed by R software (version 4.4.3) with the following packages: “TwoSample MR” (version 0.6.15), “MendelianRandomization” (version 0.10.0), “MVMR” (version 0.4), “FastTraitR” (version 1.0.1), “MRPRESSO” (version 1.0), “fdrtool” (version 1.2.18).

## 3 Results

### 3.1 Bidirectional MR Analysis

Following a series of quality control, 5262 SNPs strongly linked with 83 dietary habits were included. All F‐statistics were above 10 (range: 19.44–682.57), indicating robust instrument strength and mitigating weak instrument bias. The SNP number per exposure ranged between 8 and 217, as specified in Tables S3 and S6.

#### 3.1.1 Causal Effects of Dietary Habits on Senile Cataract

**FIGURE 2:**
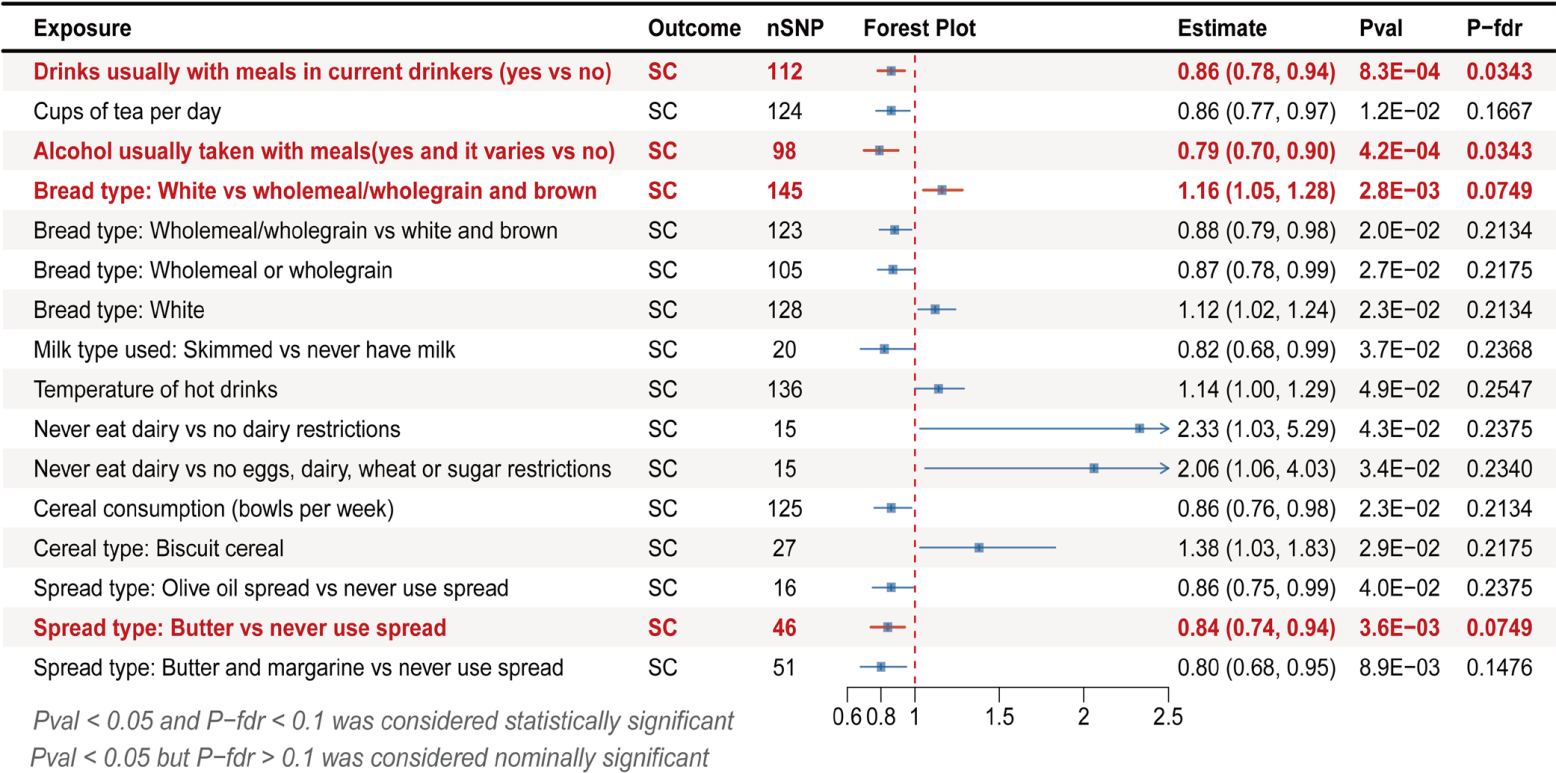
Forest plot of the forward MR analysis using inverse‐variance weighted method. FDR, false discovery rate; nSNP, the number of SNPs; SC, senile cataract.

Among 83 dietary habits, 16 were identified as associated exposures to SC in forward bidirectional MR (Figure 2). Specifically, 10 dietary habits were associated with a reduced risk of SC, including “drinks usually with meals in current drinkers (yes vs no)” (OR = 0.86; 95% CI: 0.78–0.94; *p* = 8.26 × 10^−4^), “cups of tea per day” (OR = 0.86; 95% CI: 0.77–0.97; *p* = 1.20 × 10^−2^), “alcohol usually taken with meals (yes and it varies vs no)” (OR = 0.79; 95% CI: 0.70–0.90; *p* = 4.15 × 10^−4^), “bread type: wholemeal/wholegrain vs white and brown” (OR = 0.88; 95% CI: 0.79–0.98; *p* = 1.96 × 10^−2^), “bread type: wholemeal or wholegrain” (OR = 0.87; 95% CI: 0.78–0.99; *p* = 2.73 × 10^−2^), “milk type used: skimmed vs never have milk” (OR = 0.82; 95% CI: 0.68–0.99; *p* = 3.71 × 10^−2^), “cereal consumption (bowls per week)” (OR = 0.86; 95% CI: 0.76–0.98; *p* = 2.31 × 10^−2^), “spread type: olive oil spread vs never use spread” (OR = 0.86; 95% CI: 0.75–0.99; *p* = 4.02 × 10^−2^), “spread type: butter vs never use spread” (OR = 0.84; 95% CI: 0.74–0.94; *p* = 3.61 × 10^−3^), “spread type: butter and margarine vs never use spread” (OR = 0.80; 95% CI: 0.68–0.95; *p* = 8.89 × 10^−3^). On the contrary, six dietary habits were associated with an increased risk of SC, including “bread type: white vs wholemeal/wholegrain and brown” (OR = 1.16; 95% CI: 1.05–1.28; *p* = 2.76 × 10^−3^), “bread type: white” (OR = 1.12; 95% CI: 1.02–1.24; *p* = 2.26 × 10^−2^), “temperature of hot drinks” (OR = 1.14; 95% CI: 1.00–1.29; *p* = 4.91 × 10^−2^), “never eat dairy vs no dairy restrictions” (OR = 2.33; 95% CI: 1.03–5.29; *p* = 4.29 × 10^−2^), “never eat dairy vs no eggs, dairy, wheat, or sugar restrictions” (OR = 2.06; 95% CI: 1.06–4.03; *p* = 3.38 × 10^−2^), “cereal type: biscuit cereal” (OR = 1.38; 95% CI: 1.03–1.83; *p* = 2.88 × 10^−2^). The forward MR analysis results are provided in Table S4, Figure S1 (scatter plots), and Figure S12 (complete forest plot).

Sensitivity analyses were conducted to assess the robustness of the findings. MR‐Egger regression was conducted on 16 significant dietary habits, revealing no pleiotropy in the MR‐Egger intercept test. Since random effects IVW served as the primary analysis, the Cochran Q test demonstrated that heterogeneity was acceptable. The funnel plots exhibited symmetrical distributions (Figure S2). Furthermore, leave‐one‐out analyses indicated that no single SNP markedly influenced the MR estimates (Figure S3). The results of the sensitivity analyses are presented in Table S5.

FDR correction was applied for multiple testing. Among 16 dietary habits, four showed significant associations with SC risk after FDR adjustment: “drinks usually with meals in current drinkers (yes vs no)” (IVW; *p*‐FDR = 0.03), “alcohol usually taken with meals(yes and it varies vs no)” (IVW; *p*‐FDR = 0.03), “bread type: white vs wholemeal/wholegrain and brown” (IVW; *p*‐FDR = 0.07), “spread type: Butter vs never use spread”(IVW; *p*‐FDR = 0.07). These associations were further supported by ConMix and RAPS methods, indicating that these four habits exhibit the strongest correlation with SC, while the remaining twelve show weaker associations.

#### 3.1.2 Causal Effects of Senile Cataract on Dietary Habits

**FIGURE 3:**
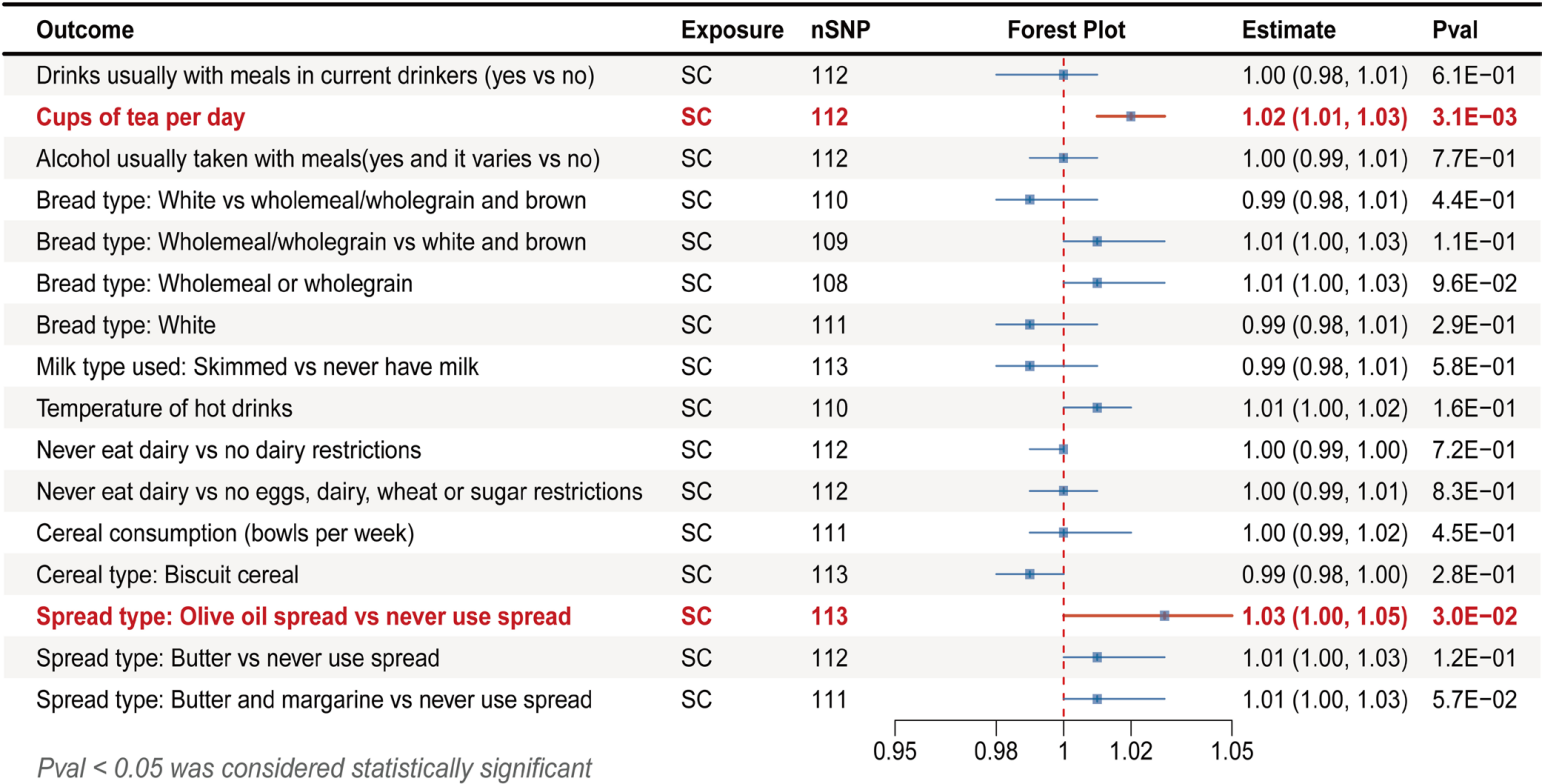
Forest plot of the reverse MR analysis using inverse‐variance weighted method. FDR, false discovery rate; nSNP, the number of SNPs; SC, senile cataract.

To identify potential exposures for downstream analysis and exclude exposures bidirectionally associated with SC, we conducted a reverse MR analysis (Figure 3). The results indicated a causal relationship between SC and “cups of tea per day” (OR = 1.02; 95% CI: 1.01–1.03; *p* = 3.09 × 10^−03^), as well as with “spread type: olive oil spread vs. never use spread” (IVW‐OR: 1.03, 95% CI 1.00–1.05, *p* = 2.99 × 10^−02^), the reverse MR analysis results are provided in Table S7, Figure S4 (scatter plots), and Figure S13 (complete forest plot). Additionally, when the “spread type Butter vs never use spread” was used as the outcome, MR‐Egger regression revealed evidence of pleiotropy. The complete sensitivity analysis results are provided in Table S8, with symmetrical funnel plots (Figure S5) and leave‐one‐out analyses indicating no single SNP markedly influenced the MR estimates (Figure S6).

### 3.2 Replication Sample MR Analysis

In the bidirectional MR analysis, exposures with evidence of bidirectional causality or pleiotropy were excluded. The remaining 13 dietary habits showing significant or suggestive associations with SC were subsequently validated through MR analysis in a replication cohort. A total of 1074 SNPs were included, all demonstrating strong instrument strength (*F*‐statistics ranging from 20.20 to 126.50) with no risk of weak instrument bias (Table S9).

**FIGURE 4:**
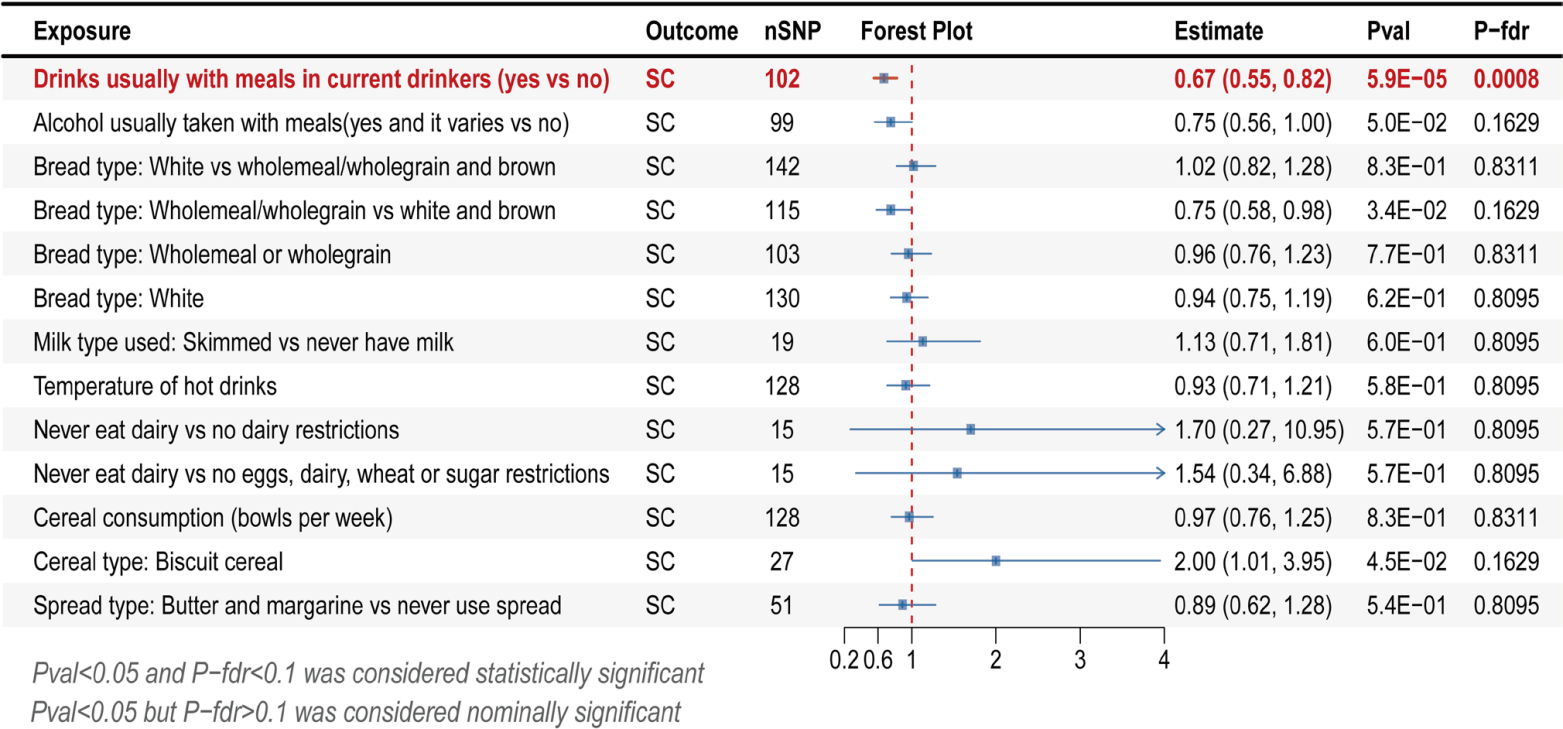
Forest plot of the replication MR analysis using the inverse‐variance weighted method. FDR, false discovery rate; nSNP, the number of SNPs; SC, senile cataract.

MR analysis replication showed that among the 4 associations significant after FDR correction in the initial analysis, only “drinks usually with meals in current drinkers (yes vs no)” maintained a significant causal effect on SC in independent validation (OR = 0.67; 95% CI = 0.55–0.82; *p* = 5.86 × 10^−5^; *p*‐FDR < 0.001), indicating a notable protective effect against SC (Figure 4). The other nine dietary habits with suggestive relationships showed no significant causal effects in the replication sample. The replication sample MR analysis results are provided in Table S10, Figure S7 (scatter plots), and Figure S14 (complete forest plot).

Neither Cochran's Q test nor the MR‐Egger intercept test found any evidence of heterogeneity or pleiotropy in the association between “drinks usually with meals in current drinkers (yes vs no)” and SC. Sensitivity analyses revealed symmetrical funnel plots (Figure S8) and demonstrated no markedly influential single SNP in leave‐one‐out analyses (Figure S9), with full results detailed in Table S11.

### 3.3 Multivariable MR Analyses

**FIGURE 5:**
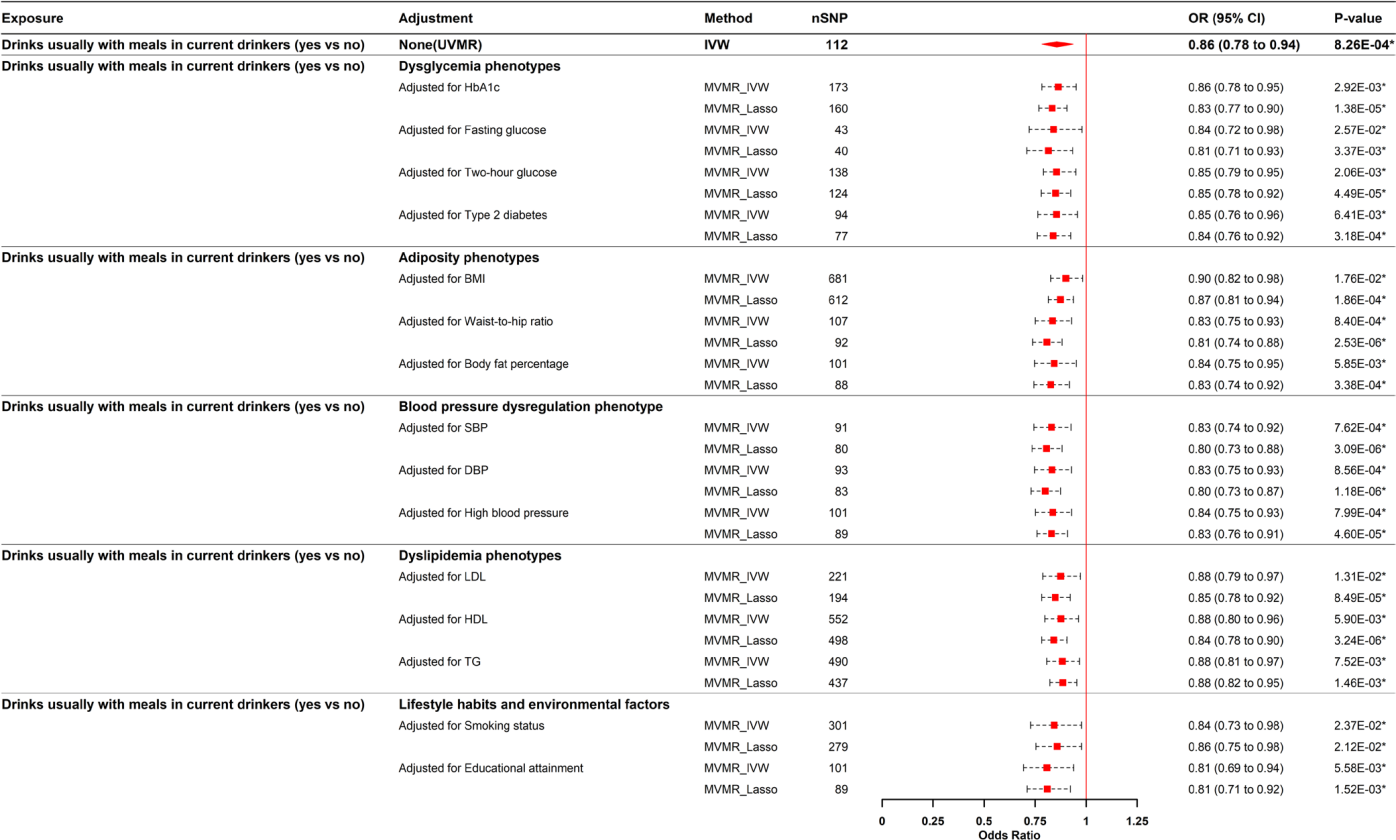
Results of the multivariable MR analyses. BMI, body mass index; DBP, diastolic blood pressure; HbA1c, glycated hemoglobin A1c; HDL, high‐density lipoprotein; IVW, inverse‐variance weighted; Lasso, least absolute shrinkage and selection operator; LDL, low‐density lipoprotein; nSNP, the number of SNPs; SBP, systolic blood pressure; TG, triglycerides; UVMR, Univariate Mendelian Randomization. *p‐value < 0.05.

MVMR analysis examined the significant causal relationship found in both the primary MR analysis and the replication cohort, specifically regarding “drinking alcohol with meals among current drinkers (yes vs. no)”. After adjustment for 15 confounding factors—covering genetically predicted dysglycemia, adiposity, blood pressure, dyslipidemia phenotypes, lifestyle habits, and environmental factors—results consistently indicated a decreased risk of SC for those consuming alcohol with meals. The three MVMR methods (Robust, Median, LASSO) produced causal estimates aligning with the multivariate IVW analysis, supporting the robustness of the findings. Results are shown in Figure 5 and Table S12. The MVMR‐Egger intercept test indicated no evidence of level pleiotropy (Table S13).

### 3.4 Two‐Step MR Analyses

MVMR analysis confirmed that the protective effect of “drinking alcohol with meals among current drinkers (yes vs no)” against SC is independent of potential confounders. To explore the underlying mechanisms, a two‐step MR analysis was conducted to assess whether inflammatory markers mediate this association.

In the first step, 1411 SNPs were selected, with *F*‐statistic ranges from 20.68 to 57.42 (Table S14). The results showed that “drinks usually with meals in current drinkers (yes vs no)” was negatively causally associated with IL‐6 (*β* = −0.19; *p* = 1.98 × 10^−2^), CRP (*β* = −0.21; *p* = 4.38 × 10^−25^), and FGF23 (*β* = −0.13; *p* = 3.77 × 10^−2^) (Table S15). Additionally, no pleiotropy was observed, and heterogeneity was found in IVs for CRP (Table S16). In the second step, a total of 513 SNPs were selected, with F‐statistic ranges between 20.25 and 2377.07 (Table S17). The findings indicated that IL‐6 (*β* = 0.10; *p* = 1.34 × 10^−3^), CRP (*β* = 0.05; *p* = 3.83 × 10^−2^), MCP‐1 (*β* = 0.05; *p* = 3.43 × 10^−2^), and bFGF (*β* = 0.09; *p* = 1.63 × 10^−2^) exhibited a causal positive correlation with the risk of SC (Table S18). Heterogeneity was noted in IVs for CRP, with no evidence of pleiotropy identified at this step (Table S19).

**FIGURE 6:**
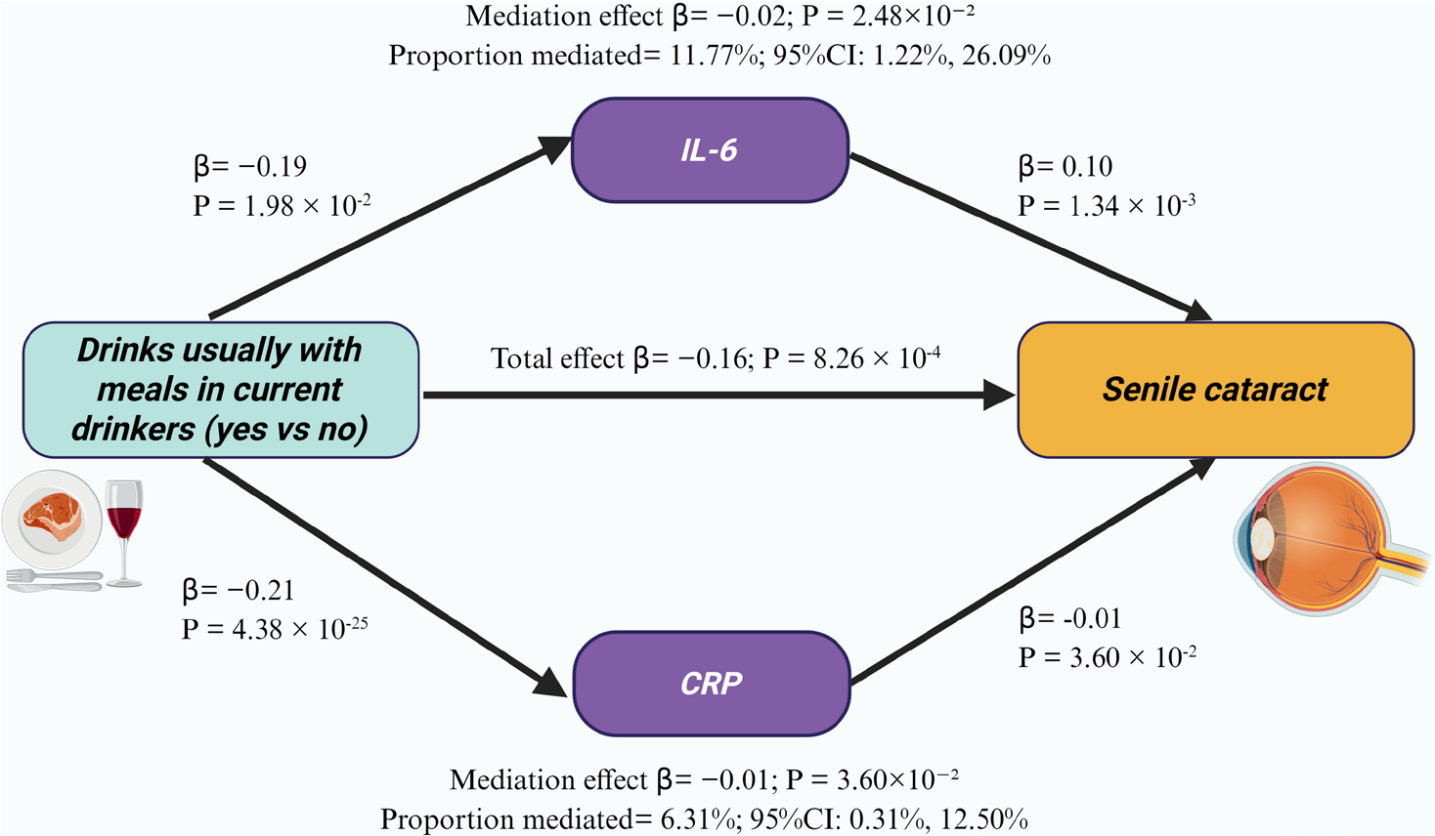
Summary plot of the causal associations among drinks usually with meals in current drinkers (yes vs. no), IL‐6, CRP, and Senile cataract. CRP, C‐reactive protein; IL‐6 interleukin‐6.

Considering all factors, IL‐6 (*β* = −0.02; *p* = 2.48 × 10^−2^) and CRP (*β* = −0.01; *p* = 3.60 × 10^−2^) were recognized as significant mediators of the causal relationship between “drinks usually with meals in current drinkers (yes vs no)” and SC, accounting for 11.77% and 6.31%, respectively (Figure 6). The remaining ten inflammatory markers did not demonstrate significant mediation effects, and their complete data can be accessed in Table S20.

### 3.5 Validation of Key Findings in Cataracts and Surgical Endpoints

To confirm key findings' validity, additional analyses utilized independent GWAS cohorts for the overall cataract phenotype and cataract surgical endpoint. A total of 219 SNPs were included, with F‐statistics ranging from 20.68 to 57.42 (Table S21). The MR analysis results indicated that the genetic prediction of “drinks usually with meals in current drinkers (yes vs. no)” exerted a protective causal effect on cataracts (OR = 0.83; 95% CI = 0.76–0.91; *p* = 5.26 × 10^−5^). An independent validation of cataract operation indicated a protective effect (OR = 0.99; 95% CI = 0.98–0.99; *p* = 2.34 × 10^−6^). The sensitivity analyses yielded robust results, supported by symmetrical funnel plots and leave‐one‐out analyses indicating no markedly influential SNP (Figures S10 and S11), with all complete data in Table S22. Reverse MR analysis did not identify bidirectional causality (Tables S24 and S25). The MR‐Egger intercept test failed to detect pleiotropy. The Cochran's Q test and pleiotropy test results are presented in Tables S23 and S26.

## 4 Discussion

This study is the first to employ MR to systematically explore the causal relationship between 83 dietary habits and SC. A key finding was that, among drinkers, consuming alcohol with meals is associated with a reduced risk of SC compared to drinking without meals. This is noteworthy since previous research has primarily focused on alcohol quantity or drinking status rather than drinking patterns. The results underscore the significance of modifiable dietary behaviors, particularly drinking patterns, in determining cataract risk.

Initially, we used bidirectional MR to assess the impact of dietary habits on SC. Of 83 dietary traits examined, four remained significantly associated with SC after FDR correction, including “drinks usually with meals in current drinkers (yes vs no)”, “alcohol usually taken with meals (yes and it varies vs no)”, “bread type: white vs wholemeal/wholegrain and brown”, and “spread type: butter vs never use spread”. Notably, “drinks usually with meals in current drinkers (yes vs. no)” was consistently linked to reduced SC risk in replication analysis, establishing it as the most influential modifiable dietary factor. We then performed MVMR adjusting for 15 confounders, which confirmed that drinking alcohol with meals independently lowers SC risk, beyond conventional risk factors. Moreover, mediation MR further revealed that IL‐6 and CRP mediated 11.77% and 6.31% of this effect, respectively. To confirm the validity of the core findings, additional analyses using independent GWAS cohorts for the overall cataract phenotype and cataract surgical endpoint consistently showed that consuming drinks with meals significantly reduces the risk of cataract and cataract surgery among current drinkers, compared to drinking without meals.

Our study provides genetic evidence for a potential causal association between the habit of drinking with meals and a reduced risk of SC. Interestingly, recent evidence indicates that consuming alcohol with meals, as opposed to outside of meals, correlates with reduced risks of all‐cause mortality, cardiovascular disease mortality, cirrhosis, type 2 diabetes, hypertension, and cancer mortality (Hernández‐Hernández et al. 2023; Ma et al. 2021, 2022; Simpson et al. 2019). These findings underscore the impact of alcohol consumption timing relative to meals, with inflammation serving as a key pathological mechanism. Notably, SC is recognized as a systemic condition associated with chronic low‐grade inflammation, which promotes oxidative stress, alters lens proteins, and ultimately leads to opacity (Huang et al. 2024). We further explored the potential mechanisms using a two‐step MR mediation analysis. The results indicate that the protective effect of consuming alcohol with meals on SC risk is partly mediated by reduced levels of systemic inflammatory markers, specifically CRP and IL‐6. As is known to all, CRP is a key inflammatory marker, and elevated levels are indicative of chronic low‐grade inflammation. Studies based on the large UK Biobank cohort indicate that a higher proportion of alcohol consumption during meals correlates with reduced circulating CRP concentrations in individuals. Conversely, in non‐meal drinkers, increased alcohol consumption correlates with elevated CRP concentrations (Ma et al. 2022). Similarly, following the findings of Estruch et al., alcohol consumption during meals significantly reduces serum high‐sensitivity CRP levels, thereby mitigating systemic inflammatory responses (Estruch et al. 2004). Randomized double‐blind studies have confirmed that elevated plasma CRP levels significantly increase the risk of SC, possibly by exacerbating cataract development through synergy with local ocular inflammatory factors (Schaumberg et al. 1999). Moreover, Tsai et al. identified CRP as a risk factor for early‐onset cataracts, supporting its use in risk prediction monitoring (Tsai et al. 2022). Furthermore, the Dietary Inflammatory Index (DII) quantifies the inflammatory potential of diet based on six circulating markers (CRP, IL‐6, IL‐4, IL‐10, IL‐1, TNF‐α). Higher DII scores, indicating a more pro‐inflammatory diet, are significantly associated with increased prevalence of SC (Shivappa et al. 2017; Vergroesen et al. 2023), underscoring the central role of inflammation in SC pathogenesis. These findings collectively support our conclusion that, among alcohol consumers, the protective effect of meal‐time drinking against SC is mediated at least partially through reduced CRP levels.

IL‐6 is a cytokine that regulates inflammation and immune responses via classical and trans‐signaling pathways (Schmidt‐Arras and Rose‐John 2016). Studies show that consuming alcohol with meals significantly reduces serum IL‐6 and other pro‐inflammatory factors (Torres et al. 2015). The Mediterranean diet is widely regarded as a healthy dietary pattern associated with benefits for aging and longevity (Andreo‐López et al. 2023). A key element of this diet is the Mediterranean alcohol‐drinking pattern (MADP), characterized by alcohol intake with meals. Studies indicate that MADP exerts anti‐inflammatory effects and can lower circulating inflammatory factors, including IL‐6 (Mena et al. 2009; Santos‐Buelga et al. 2021). Furthermore, Barbara et al. found that plasma IL‐6 levels are significantly associated with the risk of SC (Klein et al. 2006). Additionally, research involving animal models has indicated that increased IL‐6 levels in the aqueous humor are linked to lens opacification (Meacock et al. 2000). From this perspective, in alcohol‐consuming populations, consuming alcohol with meals may reduce the risk of SC compared to drinking without meals. The rationale is based on the understanding that inflammation promotes SC progression and that alcohol consumption during meals decreases systemic inflammation. The mechanisms through which CRP and IL‐6 mediate the causal relationship between this drinking pattern and SC remain ambiguous.

Beyond mediation by CRP and IL‐6, our findings are reinforced by the evidence that consuming alcohol with meals lowers blood alcohol concentration (BAC). Prolonged elevated BAC promotes formation of reactive oxygen and nitrogen species, leading to oxidative stress—a key factor in cataract development (Barbería‐Latasa et al. 2022). When BAC exceeds 0.8‰–1‰ (lower in females), hepatic alcohol dehydrogenase becomes saturated, and metabolism shifts to the microsomal ethanol‐oxidizing system, which generates oxidative free radicals that promote lens protein aggregation and cataract formation (Lieber 2004; Wang et al. 2008). Research indicates that drinking with meals not only slows gastrointestinal ethanol absorption and lowers peak BAC but also enhances ethanol metabolism and elimination (Jones 2000; Ramchandani et al. 2001). Foods containing various components exhibit comparable effects on the acceleration of alcohol elimination, suggesting that the influence of food is not derived from interactions with particular food components (Ramchandani et al. 2001). Additionally, fasting alcohol intake increases hyperhomocysteinemia risk fivefold (Rouillier et al. 2006), which may exacerbate cataracts via mTOR activation, impaired autophagy, and reduced connexin expression (Liu et al. 2024). Thus, we propose that meal‐time drinking may delay SC by reducing ethanol‐induced oxidative stress, accelerating alcohol elimination, and lowering homocysteine levels.

An interesting observation is that habitual consumption of alcohol with meals is associated with a significant reduction in the overall incidence risk of cataracts and a decrease in the risk of stage‐specific lesions necessitating surgical intervention. Nonetheless, the change in odds ratio indicates that its protective effect against the risk of cataract surgery has been somewhat diminished. This demonstrates that, in alcohol‐consuming populations, meal‐accompanied alcohol consumption may primarily inhibit the progression of lens damage in the early stages of cataracts. In contrast, its protective effect against advanced structural lesions is restricted compared to non‐meal‐accompanied alcohol consumption. This underscores the significance of early lifestyle intervention in preventing cataract progression.

This study has several significant strengths. Initially, we utilized summary statistics from various extensive GWAS to identify instrumental SNPs. Significantly, this study effectively utilized MR to mimic the effects of a well‐structured RCT, which is considered the gold standard in causal inference. Second, by integrating bidirectional MR, MVMR, and mediational MR, we attained a more precise evaluation of the causal relationship between the target exposure and outcome. The MR method effectively addresses the confounding effect of reverse causality, a challenge often difficult to resolve in observational studies.

This study, while possessing notable strengths, also has several limitations. First, the study samples were limited to individuals of European ancestry, potentially hindering the direct generalization of the findings to other ethnic groups. Significant differences in genetic susceptibility to alcohol metabolism exist between Asian and Western populations, particularly concerning the capacity for alcohol metabolism and the types of alcohol consumed with meals (Im et al. 2023). Second, dietary exposure data were collected using food frequency questionnaires (FFQs), a self‐report tool susceptible to recall bias and social desirability bias. This inherent measurement error may lead to misestimation of the effect estimates. Third, due to the insufficient summary data with adequate statistical power for other cataract subtypes in existing public GWAS databases, the relationship between dietary habits and cataract subtypes was not evaluated. This study concentrated on SC and their extended phenotypes to enhance the scientific value and interpretability of results. Fourth, despite the enforcement of stringent measures, the complete eradication of heterogeneity's potential impact on this study remains unattainable. Additionally, the causal association between drinks usually with meals in current drinkers and SC cannot disentangle the distinct contributions of alcohol beverage type and consumption level. Consequently, future research using more granular data and stratified analyses is needed to investigate these specific factors. Finally, although this study found that habitual meal‐time alcohol consumption may prevent SC onset by reducing inflammatory markers (e.g., IL‐6, CRP) in current drinkers, its precise mechanisms remain unclear and require further research.

## 5 Conclusion

In summary, this study provides strong genetic evidence that consuming alcohol with meals exerts a protective causal effect against SC among current drinkers. This protective relationship is partially mediated by the inflammatory biomarkers CRP and IL‐6. Critically, these findings do not encourage alcohol consumption for prevention. Instead, they highlight the importance of considering the timing of alcohol consumption—not just the amount and type—in developing alcohol‐related nutritional strategies toward SC prevention in aging populations who are current drinkers. Future research should focus on more precisely defining drinking patterns and elucidating their specific roles in SC pathogenesis.

## Author Contributions

**Tao Wang:** formal analysis, writing – review and editing. **Jian Deng:** conceptualization, methodology, data curation, project administration, investigation, resources, writing – original draft. **Liduo Pan:** data curation, writing – review and editing. **Jianli Yang:** data curation, visualization. **Meng Wang:** conceptualization, resources. **Yonghao Li:** funding acquisition, writing – review and editing. **Jiangfeng Huang:** data curation, supervision, validation.

## Funding

This study was supported by the Shenzhen Basic Research Project for Natural Science Foundation (Grant No. JCYJ20250604184001002) to Yonghao Li.

## Ethics Statement

The ethical clearance for each of the original studies is documented in the corresponding primary literature.

## Conflicts of Interest

The authors declare no conflicts of interest.

## Supporting information


**Data S1:** fsn371366‐sup‐0001‐Figures.docx.

**Data S2:** fsn371366‐sup‐0002‐Tables.xlsx.

**Data S3:** fsn371366‐sup‐0003‐Supinfo1.docx.

**Data S4:** fsn371366‐sup‐0004‐Supinfo2.docx.

## Data Availability

All genetic association data used in this study are publicly available through the IEU Open GWAS database (https://gwas.mrcieu.ac.uk/) and FinnGen release R12 (https://www.finngen.fi/en).

## References

[fsn371366-bib-0001] Andreo‐López, M. C. , V. Contreras‐Bolívar , M. Muñoz‐Torres , B. García‐Fontana , and C. García‐Fontana . 2023. “Influence of the Mediterranean Diet on Healthy Aging.” International Journal of Molecular Sciences 24, no. 5: 4491. 10.3390/ijms24054491. 36901921 PMC10003249

[fsn371366-bib-0002] Asbell, P. A. , I. Dualan , J. Mindel , D. Brocks , M. Ahmad , and S. Epstein . 2005. “Age‐Related Cataract.” Lancet 365, no. 9459: 599–609. 10.1016/s0140-6736(05)17911-2. 15708105

[fsn371366-bib-0003] Barbería‐Latasa, M. , A. Gea , and M. A. Martínez‐González . 2022. “Alcohol, Drinking Pattern, and Chronic Disease.” Nutrients 14, no. 9: 1954. 10.3390/nu14091954. 35565924 PMC9100270

[fsn371366-bib-0004] Bosello, F. , A. Vanzo , C. Zaffalon , et al. 2024. “Obesity, Body Fat Distribution and Eye Diseases.” Eating and Weight Disorders: EWD 29, no. 1: 33. 10.1007/s40519-024-01662-8. 38710948 PMC11074037

[fsn371366-bib-0005] Bourne, R. R. , G. A. Stevens , R. A. White , et al. 2013. “Causes of Vision Loss Worldwide, 1990–2010: A Systematic Analysis.” Lancet Global Health 1, no. 6: e339–e349. 10.1016/s2214-109x(13)70113-x. 25104599

[fsn371366-bib-0006] Boutin, T. S. , D. G. Charteris , A. Chandra , et al. 2020. “Insights Into the Genetic Basis of Retinal Detachment.” Human Molecular Genetics 29, no. 4: 689–702. 10.1093/hmg/ddz294. 31816047 PMC7068119

[fsn371366-bib-0007] Burgess, S. , and S. G. Thompson . 2011. “Avoiding Bias From Weak Instruments in Mendelian Randomization Studies.” International Journal of Epidemiology 40, no. 3: 755–764. 10.1093/ije/dyr036. 21414999

[fsn371366-bib-0008] Camacho‐Barcia, L. , M. Bulló , J. F. García‐Gavilán , et al. 2019. “Dairy Products Intake and the Risk of Incident Cataracts Surgery in an Elderly Mediterranean Population: Results From the Predimed Study.” European Journal of Nutrition 58, no. 2: 619–627. 10.1007/s00394-018-1647-8. 29589119

[fsn371366-bib-0009] Chen, H. , B. Ye , W. Su , et al. 2023. “The Causal Role of Gut Microbiota in Susceptibility and Severity of Covid‐19: A Bidirectional Mendelian Randomization Study.” Journal of Medical Virology 95, no. 7: e28880. 10.1002/jmv.28880. 37409643

[fsn371366-bib-0010] Cole, J. B. , J. C. Florez , and J. N. Hirschhorn . 2020. “Comprehensive Genomic Analysis of Dietary Habits in UK Biobank Identifies Hundreds of Genetic Associations.” Nature Communications 11, no. 1: 1467. 10.1038/s41467-020-15193-0. PMC7081342 32193382

[fsn371366-bib-0011] Davies, N. M. , M. V. Holmes , and G. Davey Smith . 2018. “Reading Mendelian Randomisation Studies: A Guide, Glossary, and Checklist for Clinicians.” British Medical Journal 362: k601. 10.1136/bmj.k601. 30002074 PMC6041728

[fsn371366-bib-0012] Estruch, R. , E. Sacanella , E. Badia , et al. 2004. “Different Effects of Red Wine and Gin Consumption on Inflammatory Biomarkers of Atherosclerosis: A Prospective Randomized Crossover Trial. Effects of Wine on Inflammatory Markers.” Atherosclerosis 175, no. 1: 117–123. 10.1016/j.atherosclerosis.2004.03.006. 15186955

[fsn371366-bib-0013] Falkowska, M. , M. Młynarczyk , Z. Micun , J. Konopińska , and K. Socha . 2023. “Influence of Diet, Dietary Products and Vitamins on Age‐Related Cataract Incidence: A Systematic Review.” Nutrients 15, no. 21: 4585. 10.3390/nu15214585. 37960238 PMC10650191

[fsn371366-bib-0014] Franke, S. , J. Dawczynski , J. Strobel , T. Niwa , P. Stahl , and G. Stein . 2003. “Increased Levels of Advanced Glycation End Products in Human Cataractous Lenses.” Journal of Cataract and Refractive Surgery 29, no. 5: 998–1004. 10.1016/s0886-3350(02)01841-2. 12781289

[fsn371366-bib-0015] GBD 2019 Blindness and Vision Impairment Collaborators and Vision Loss Expert Group of the Global Burden of Disease Study . 2021. “Causes of Blindness and Vision Impairment in 2020 and Trends Over 30 Years, and Prevalence of Avoidable Blindness in Relation to Vision 2020: The Right to Sight: An Analysis for the Global Burden of Disease Study.” Lancet Global Health 9, no. 2: e144–e160. 10.1016/s2214-109x(20)30489-7. 33275949 PMC7820391

[fsn371366-bib-0016] Greco, M. F. , C. Minelli , N. A. Sheehan , and J. R. Thompson . 2015. “Detecting Pleiotropy in Mendelian Randomisation Studies With Summary Data and a Continuous Outcome.” Statistics in Medicine 34, no. 21: 2926–2940. 10.1002/sim.6522. 25950993

[fsn371366-bib-0017] He, M. , W. Wang , and W. Huang . 2017. “Variations and Trends in Health Burden of Visual Impairment due to Cataract: A Global Analysis.” Investigative Ophthalmology & Visual Science 58, no. 10: 4299–4306. 10.1167/iovs.17-21459. 28846778

[fsn371366-bib-0018] Hernández‐Hernández, A. , D. Oliver , M. Martínez‐González , et al. 2023. “Mediterranean Alcohol‐Drinking Pattern and Arterial Hypertension in the ‘Seguimiento Universidad De Navarra’ (Sun) Prospective Cohort Study.” Nutrients 15, no. 2: 20307. 10.3390/nu15020307. PMC9865916 36678178

[fsn371366-bib-0019] Huang, J. , H. Wu , F. Yu , et al. 2024. “Association Between Systemic Immune‐Inflammation Index and Cataract Among Outpatient us Adults.” Frontiers in Medicine 11: 1469200. 10.3389/fmed.2024.1469200. 39359932 PMC11445128

[fsn371366-bib-0020] Im, P. K. , N. Wright , L. Yang , et al. 2023. “Alcohol Consumption and Risks of More Than 200 Diseases in Chinese Men.” Nature Medicine 29, no. 6: 1476–1486. 10.1038/s41591-023-02383-8. PMC10287564 37291211

[fsn371366-bib-0021] Jee, D. , and S. Park . 2021. “Hyperglycemia and Hypo‐Hdl‐Cholesterolemia Are Primary Risk Factors for Age‐Related Cataract, and a Korean‐Style Balanced Diet Has a Negative Association, Based on the Korean Genome and Epidemiology Study.” Journal of Korean Medical Science 36, no. 23: e155. 10.3346/jkms.2021.36.e155. 34128595 PMC8203849

[fsn371366-bib-0022] Jones, A. W. 2000. “Aspects of In‐Vivo Pharmacokinetics of Ethanol.” Alcoholism, Clinical and Experimental Research 24, no. 4: 400–402. 10798564

[fsn371366-bib-0023] Klein, B. E. , R. Klein , K. E. Lee , B. E. K. Klein , M. D. Knudtson , and M. Y. Tsai . 2006. “Markers of Inflammation, Vascular Endothelial Dysfunction, and Age‐Related Cataract.” American Journal of Ophthalmology 141, no. 1: 116–122. 10.1016/j.ajo.2005.08.021. 16386984

[fsn371366-bib-0024] Lawlor, D. A. , R. M. Harbord , J. A. Sterne , J. A. C. Sterne , N. Timpson , and G. Davey Smith . 2008. “Mendelian Randomization: Using Genes as Instruments for Making Causal Inferences in Epidemiology.” Statistics in Medicine 27, no. 8: 1133–1163. 10.1002/sim.3034. 17886233

[fsn371366-bib-0025] Lee, B. J. , and N. A. Afshari . 2023. “Advances in Drug Therapy and Delivery for Cataract Treatment.” Current Opinion in Ophthalmology 34, no. 1: 3–8. 10.1097/icu.0000000000000910. 36484206

[fsn371366-bib-0026] Li, S. , D. Li , Y. Zhang , J. Teng , M. Shao , and W. Cao . 2018. “Association Between Serum Lipids Concentration and Patients With Age‐Related Cataract in China: A Cross‐Sectional, Case‐Control Study.” BMJ Open 8, no. 4: e021496. 10.1136/bmjopen-2018-021496. PMC5892756 29626052

[fsn371366-bib-0027] Lieber, C. S. 2004. “The Discovery of the Microsomal Ethanol Oxidizing System and Its Physiologic and Pathologic Role.” Drug Metabolism Reviews 36, no. 3–4: 511–529. 10.1081/dmr-200033441. 15554233

[fsn371366-bib-0028] Liu, W. N. , H. L. Huang , Y. Lan , et al. 2024. “Hyperhomocysteine Promotes Cataract Development Through Mtor‐Mediated Inhibition of Autophagy and Connexins Expression.” International Immunopharmacology 140: 112827. 10.1016/j.intimp.2024.112827. 39116497

[fsn371366-bib-0029] Lu, M. , A. Taylor , L. T. Chylack Jr. , et al. 2005. “Dietary Fat Intake and Early Age‐Related Lens Opacities.” American Journal of Clinical Nutrition 81, no. 4: 773–779. 10.1093/ajcn/81.4.773. 15817851

[fsn371366-bib-0030] Ma, H. , X. Li , T. Zhou , et al. 2021. “Alcohol Consumption Levels as Compared With Drinking Habits in Predicting All‐Cause Mortality and Cause‐Specific Mortality in Current Drinkers.” Mayo Clinic Proceedings 96, no. 7: 1758–1769. 10.1016/j.mayocp.2021.02.011. 34218856 PMC8262073

[fsn371366-bib-0031] Ma, H. , X. Wang , X. Li , Y. Heianza , and L. Qi . 2022. “Moderate Alcohol Drinking With Meals Is Related to Lower Incidence of Type 2 Diabetes.” American Journal of Clinical Nutrition 116, no. 6: 1507–1514. 10.1093/ajcn/nqac207. 36250602 PMC9761774

[fsn371366-bib-0032] Meacock, W. R. , D. J. Spalton , and M. R. Stanford . 2000. “Role of Cytokines in the Pathogenesis of Posterior Capsule Opacification.” British Journal of Ophthalmology 84, no. 3: 332–336. 10.1136/bjo.84.3.332. 10684849 PMC1723397

[fsn371366-bib-0033] Mena, M. P. , E. Sacanella , M. Vazquez‐Agell , et al. 2009. “Inhibition of Circulating Immune Cell Activation: A Molecular Antiinflammatory Effect of the Mediterranean Diet.” American Journal of Clinical Nutrition 89, no. 1: 248–256. 10.3945/ajcn.2008.26094. 19056596

[fsn371366-bib-0034] Meng, Y. , Z. Tan , A. Sawut , L. Li , and C. Chen . 2024. “Association Between Life's Essential 8 and Cataract Among us Adults.” Scientific Reports 14, no. 1: 13101. 10.1038/s41598-024-63973-1. 38849465 PMC11161494

[fsn371366-bib-0035] Nam, G. E. , K. Han , S. G. Ha , et al. 2015. “Relationship Between Socioeconomic and Lifestyle Factors and Cataracts in Koreans: The Korea National Health and Nutrition Examination Survey 2008‐2011.” Eye (London, England) 29, no. 7: 913–920. 10.1038/eye.2015.66. 25976646 PMC4506346

[fsn371366-bib-0036] Nazarian, A. , K. G. Arbeev , A. P. Yashkin , and A. M. Kulminski . 2019. “Genetic Heterogeneity of Alzheimer's Disease in Subjects With and Without Hypertension.” Geroscience 41, no. 2: 137–154. 10.1007/s11357-019-00071-5. 31055733 PMC6544706

[fsn371366-bib-0037] Nordström, M. , M. Zetterberg , K. Torén , L. Schiöler , and M. Holm . 2025. “The More Smoking the More Cataract: A Study on Smoking, Snus Use and Cataract in a Swedish Population.” Acta Ophthalmologica 103, no. 1: 77–84. 10.1111/aos.16770. 39422508 PMC11704833

[fsn371366-bib-0038] Priyadarshini, K. , N. Sharma , M. Kaur , and J. S. Titiyal . 2023. “Cataract Surgery in Ocular Surface Disease.” Indian Journal of Ophthalmology 71, no. 4: 1167–1175. 10.4103/ijo.Ijo_3395_22. 37026248 PMC10276679

[fsn371366-bib-0039] Ramchandani, V. A. , P. Y. Kwo , and T. K. Li . 2001. “Effect of Food and Food Composition on Alcohol Elimination Rates in Healthy Men and Women.” Journal of Clinical Pharmacology 41, no. 12: 1345–1350. 10.1177/00912700122012814. 11762562

[fsn371366-bib-0040] Ramos‐Martínez, I. , O. Vivanco‐Rojas , B. Juárez‐Domínguez , et al. 2021. “Abnormal N‐Glycosylation of Human Lens Epithelial Cells in Type‐2 Diabetes May Contribute to Cataract Progression.” Clinical Ophthalmology 15: 1365–1373. 10.2147/opth.S300242. 33833495 PMC8020457

[fsn371366-bib-0041] Rouillier, P. , S. Bertrais , J. J. Daudin , et al. 2006. “Drinking Patterns Are Associated With Variations in Atherosclerotic Risk Factors in French Men.” European Journal of Nutrition 45, no. 2: 79–87. 10.1007/s00394-005-0567-6. 16003590

[fsn371366-bib-0042] Sakaue, S. , M. Kanai , Y. Tanigawa , et al. 2021. “A Cross‐Population Atlas of Genetic Associations for 220 Human Phenotypes.” Nature Genetics 53, no. 10: 1415–1424. 10.1038/s41588-021-00931-x. 34594039 PMC12208603

[fsn371366-bib-0043] Santos‐Buelga, C. , S. González‐Manzano , and A. M. González‐Paramás . 2021. “Wine, Polyphenols, and Mediterranean Diets. What Else Is There to Say?” Molecules 26, no. 18: 5537. 10.3390/molecules26185537. 34577008 PMC8468969

[fsn371366-bib-0044] Schaumberg, D. A. , P. M. Ridker , R. J. Glynn , W. G. Christen , M. R. Dana , and C. H. Hennekens . 1999. “High Levels of Plasma C‐Reactive Protein and Future Risk of Age‐Related Cataract.” Annals of Epidemiology 9, no. 3: 166–171. 10.1016/s1047-2797(98)00049-0. 10192648

[fsn371366-bib-0045] Schmidt‐Arras, D. , and S. Rose‐John . 2016. “Il‐6 Pathway in the Liver: From Physiopathology to Therapy.” Journal of Hepatology 64, no. 6: 1403–1415. 10.1016/j.jhep.2016.02.004. 26867490

[fsn371366-bib-0046] Shivappa, N. , J. R. Hébert , B. Rashidkhani , and M. Ghanavati . 2017. “Inflammatory Potential of Diet Is Associated With Increased Odds of Cataract in a Case‐Control Study From Iran.” International Journal for Vitamin and Nutrition Research 87, no. 1–2: 17–24. 10.1024/0300-9831/a000420. 29327971

[fsn371366-bib-0047] Simpson, R. F. , C. Hermon , B. Liu , et al. 2019. “Alcohol Drinking Patterns and Liver Cirrhosis Risk: Analysis of the Prospective UK Million Women Study.” Lancet Public Health 4, no. 1: e41–e48. 10.1016/s2468-2667(18)30230-5. 30472032 PMC6323353

[fsn371366-bib-0048] Skrivankova, V. W. , R. C. Richmond , B. A. R. Woolf , et al. 2021. “Strengthening the Reporting of Observational Studies in Epidemiology Using Mendelian Randomization: The Strobe‐Mr Statement.” JAMA 326, no. 16: 1614–1621. 10.1001/jama.2021.18236. 34698778

[fsn371366-bib-0049] Smith, G. D. , and S. Ebrahim . 2003. “Mendelian Randomization': Can Genetic Epidemiology Contribute to Understanding Environmental Determinants of Disease?” International Journal of Epidemiology 32, no. 1: 1–22. 10.1093/ije/dyg070. 12689998

[fsn371366-bib-0050] Su, Q. , J. Li , Y. Lu , et al. 2025. “Spleen Volume in Relation to Ulcerative Colitis and Crohn's Disease: A Mendelian Randomization Study.” Scientific Reports 15, no. 1: 6588. 10.1038/s41598-025-90104-1. 39994250 PMC11850802

[fsn371366-bib-0051] Su, Q. , Y. Lu , S. He , et al. 2025. “Assessing Inflammatory Protein Factors in Inflammatory Bowel Disease Using Multivariable Mendelian Randomization.” Scientific Reports 15, no. 1: 210. 10.1038/s41598-024-84447-4. 39747981 PMC11696058

[fsn371366-bib-0052] Tan, A. G. , V. M. Flood , A. Kifley , et al. 2020. “Wholegrain and Legume Consumption and the 5‐Year Incidence of Age‐Related Cataract in the Blue Mountains Eye Study.” British Journal of Nutrition 124, no. 3: 306–315. 10.1017/s000711452000104x. 32189601

[fsn371366-bib-0053] Torres, A. , V. Cachofeiro , J. Millán , et al. 2015. “Red Wine Intake but Not Other Alcoholic Beverages Increases Total Antioxidant Capacity and Improves Pro‐Inflammatory Profile After an Oral Fat Diet in Healthy Volunteers.” Revista Clínica Española 215, no. 9: 486–494. 10.1016/j.rce.2015.07.002. 26297333

[fsn371366-bib-0054] Tsai, L. H. , C. C. Chen , C. J. Lin , S.‐P. Lin , C.‐Y. Cheng , and H.‐P. Hsieh . 2022. “Risk Factor Analysis of Early‐Onset Cataracts in Taiwan.” Journal of Clinical Medicine 11, no. 9: 2374. 10.3390/jcm11092374. 35566498 PMC9101402

[fsn371366-bib-0055] Verbanck, M. , C. Y. Chen , B. Neale , and R. Do . 2018. “Detection of Widespread Horizontal Pleiotropy in Causal Relationships Inferred From Mendelian Randomization Between Complex Traits and Diseases.” Nature Genetics 50, no. 5: 693–698. 10.1038/s41588-018-0099-7. 29686387 PMC6083837

[fsn371366-bib-0056] Vergroesen, J. E. , E. F. Thee , T. O. E. de Crom , et al. 2023. “The Inflammatory Potential of Diet Is Associated With the Risk of Age‐Related Eye Diseases.” Clinical Nutrition (Edinburgh, Scotland) 42, no. 12: 2404–2413. 10.1016/j.clnu.2023.10.008. 37865012

[fsn371366-bib-0057] Wang, B. , X. Bai , Y. Yang , and H. Yang . 2025. “Possible Linking and Treatment Between Parkinson's Disease and Inflammatory Bowel Disease: A Study of Mendelian Randomization Based on Gut‐Brain Axis.” Journal of Translational Medicine 23, no. 1: 45. 10.1186/s12967-024-06045-2. 39799347 PMC11725218

[fsn371366-bib-0058] Wang, S. , J. J. Wang , and T. Y. Wong . 2008. “Alcohol and Eye Diseases.” Survey of Ophthalmology 53, no. 5: 512–525. 10.1016/j.survophthal.2008.06.003. 18929762

[fsn371366-bib-0059] Wu, X. , Z. Liu , D. Wang , et al. 2018. “Preoperative Profile of Inflammatory Factors in Aqueous Humor Correlates With Postoperative Inflammatory Response in Patients With Congenital Cataract.” Molecular Vision 24: 414–424. 29930475 PMC5993531

[fsn371366-bib-0060] Yan, W. , W. Wang , P. van Wijngaarden , A. Mueller , and M. He . 2019. “Longitudinal Changes in Global Cataract Surgery Rate Inequality and Associations With Socioeconomic Indices.” Clinical & Experimental Ophthalmology 47, no. 4: 453–460. 10.1111/ceo.13430. 30362287

[fsn371366-bib-0061] Yu, X. , D. Lyu , X. Dong , J. He , and K. Yao . 2014. “Hypertension and Risk of Cataract: A Meta‐Analysis.” PLoS One 9, no. 12: e114012. 10.1371/journal.pone.0114012. 25474403 PMC4256215

[fsn371366-bib-0062] Zhou, J. , L. Lou , K. Jin , and J. Ye . 2022. “Association Between Healthy Eating Index‐2015 and Age‐Related Cataract in American Adults: A Cross‐Sectional Study of Nhanes 2005–2008.” Nutrients 15, no. 1: 98. 10.3390/nu15010098. 36615757 PMC9823857

[fsn371366-bib-0063] Zhou, W. , J. B. Nielsen , L. G. Fritsche , et al. 2018. “Efficiently Controlling for Case‐Control Imbalance and Sample Relatedness in Large‐Scale Genetic Association Studies.” Nature Genetics 50, no. 9: 1335–1341. 10.1038/s41588-018-0184-y. 30104761 PMC6119127

