# Supplementary material for: Modifiable Dietary Habits, Inflammatory Mediation, and Senile Cataract: Unraveling Causality via Mendelian Randomization
Source: Food Sci Nutr. 2025 Dec 17;13(12):e71366. doi: 10.1002/fsn3.71366 (PMC12710447; doi:10.1002/fsn3.71366)
Supplement: Supplementary file 1 — Data S1: fsn371366‐sup‐0001‐Figures.docx. [file FSN3-13-e71366-s001.docx]

**Supplementary Figures**

**Figure S1.** Scatter plots for the association between 16 significant dietary habits and SC in the forward analysis of bidirectional MR.

**Figure S2.** Funnel plot for the association between 16 significant dietary habits and SC in the forward analysis of bidirectional MR.

**Figure S3.** Leave-one-out analysis for the association between 16 significant dietary habits and SC in the forward analysis of bidirectional MR.

**Figure S4.** Scatter plots for the association between 16 significant dietary habits and SC in the reverse analysis of bidirectional MR.

**Figure S5.** Funnel plot for the association between 16 significant dietary habits and SC in the reverse analysis of bidirectional MR.

**Figure S6.** Leave-one-out analysis for the association between 16 significant dietary habits and SC in the reverse analysis of bidirectional MR.

**Figure S7.** Scatter plots for the association between 13 significant dietary habits and SC in the replication sample MR analysis.

**Figure S8.** Funnel plot for the association between 13 significant dietary habits and SC in the replication sample MR analysis.

**Figure S9.** Leave-one-out analysis for the association between 13 significant dietary habits and SC in the replication sample MR analysis.

**Figure S10.** Bidirectional MR results of Drinks usually with meals in current drinkers (yes vs no) on Cataract.

**Figure S11.** Bidirectional MR results of Drinks usually with meals in current drinkers (yes vs no) on Cataracts operation.
**Figure S12.** Results of the forward MR analysis.
**Figure S13.** Results of the reverse MR analysis.
**Figure S14.** Results of the replication MR analysis.

**Abbreviations:** **MR**, mendelian randomization; **SC**, senile cataract; **SNP**, Single-nucleotide polymorphism.

**Figure S1.** Scatter plots for the association between 16 significant dietary habits and SC in the forward analysis of bidirectional MR.


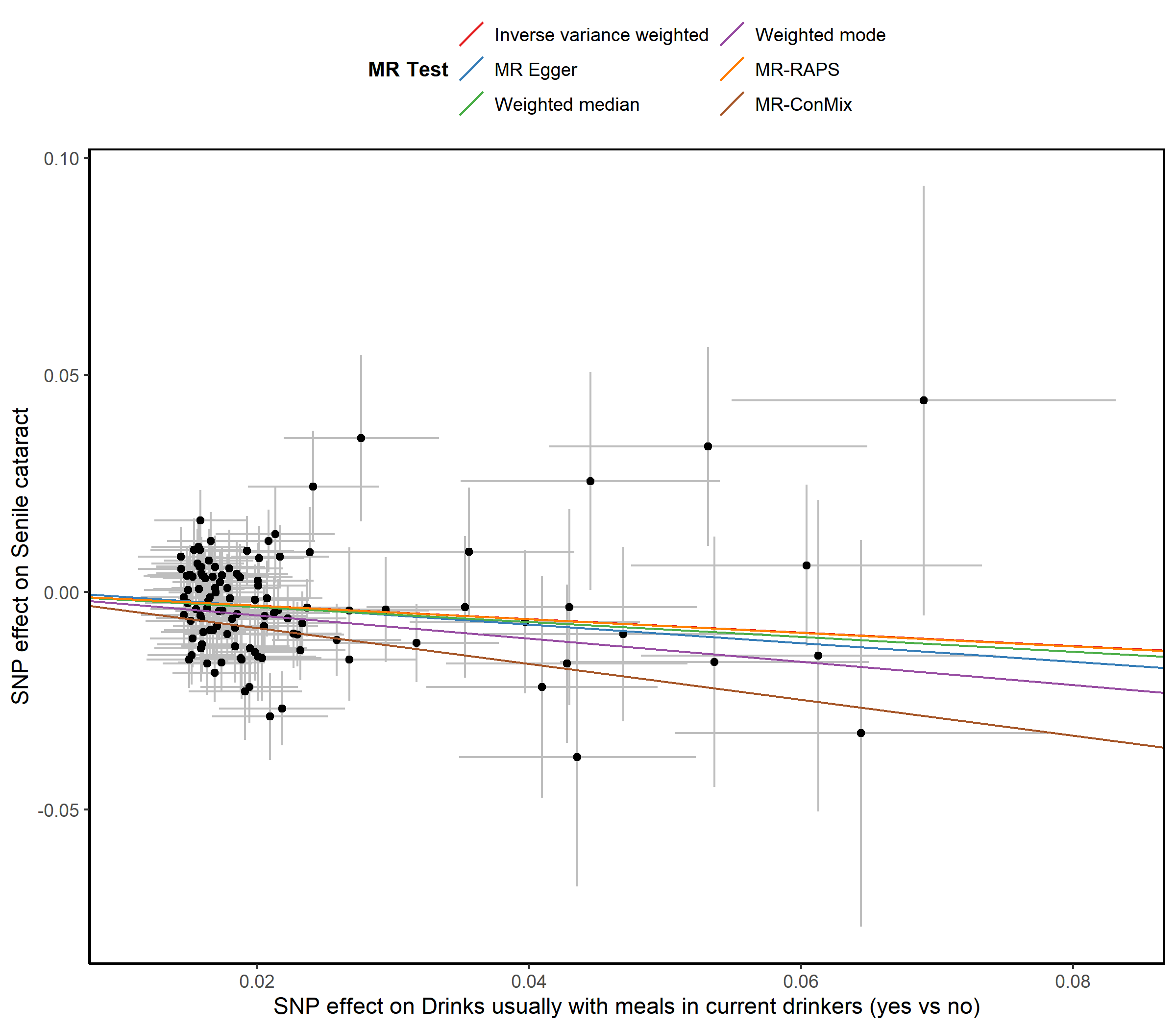


Figure S1.1 Scatter plot of SNPs associated with Drinks usually with meals in current drinkers (yes vs no) on SC.


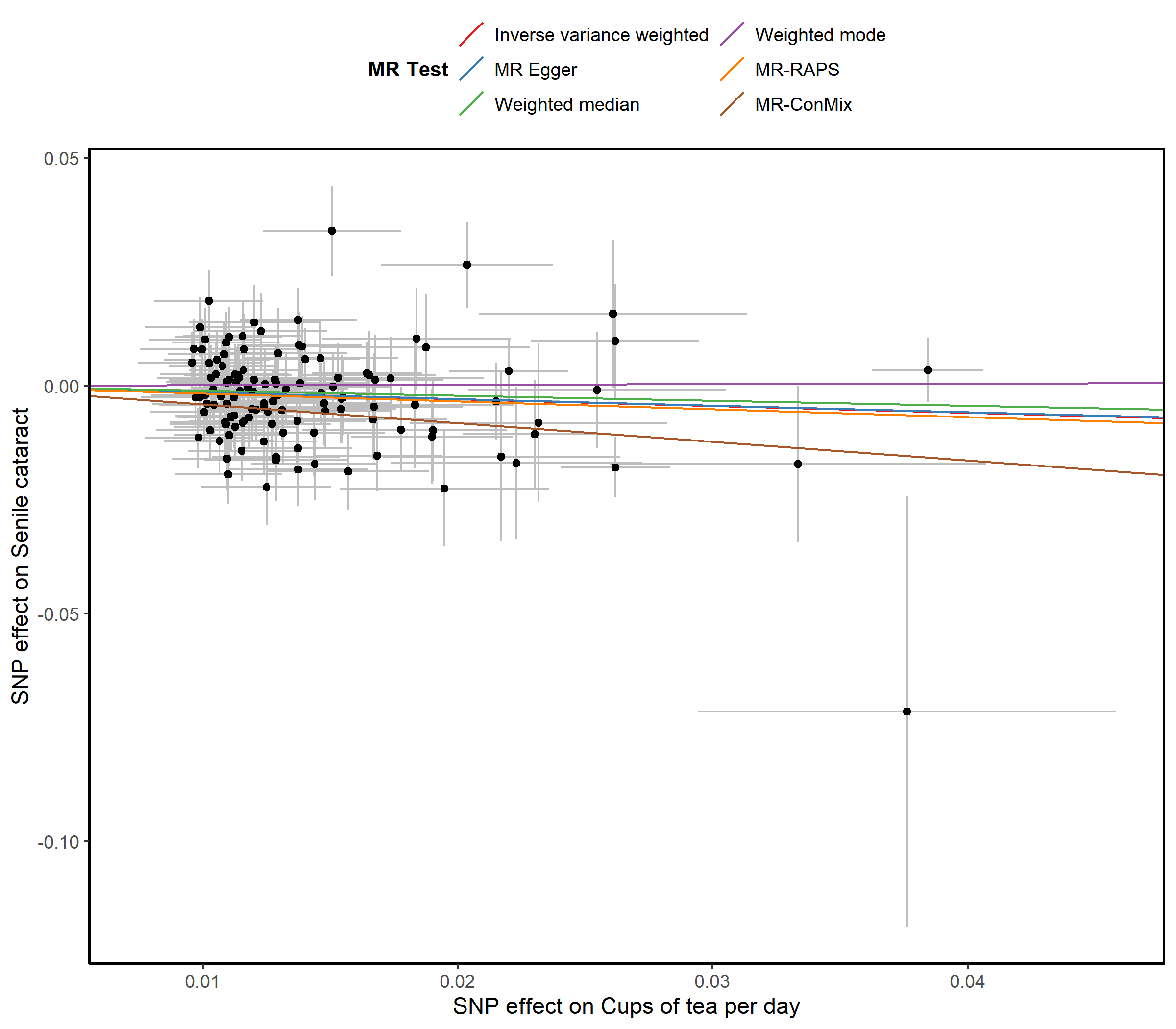


Figure S1.2 Scatter plot of SNPs associated with Cups of tea per day on SC.


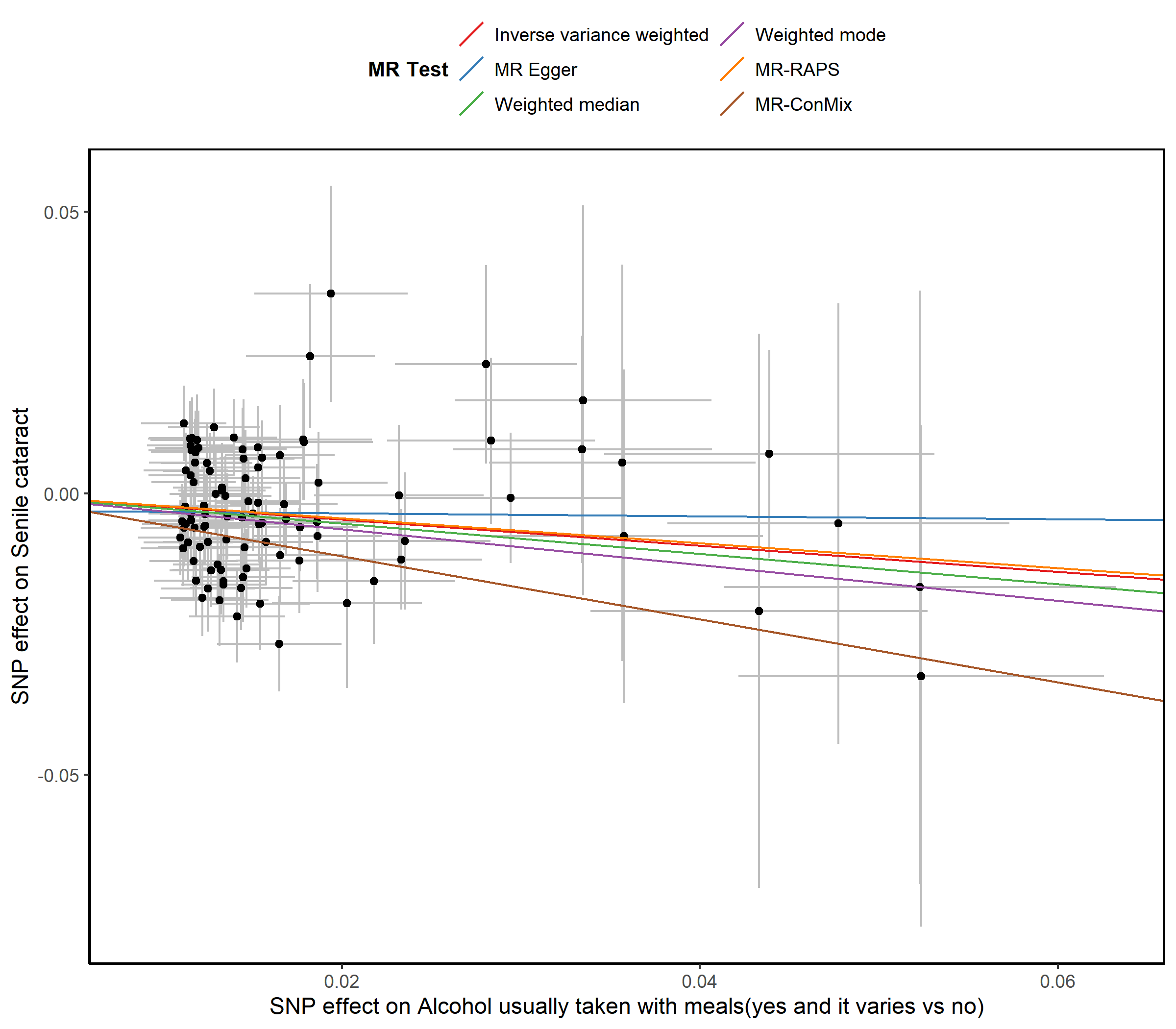


Figure S1.3 Scatter plot of SNPs associated with Acohol usually taken with meals (yes and it varies vs no) on SC.


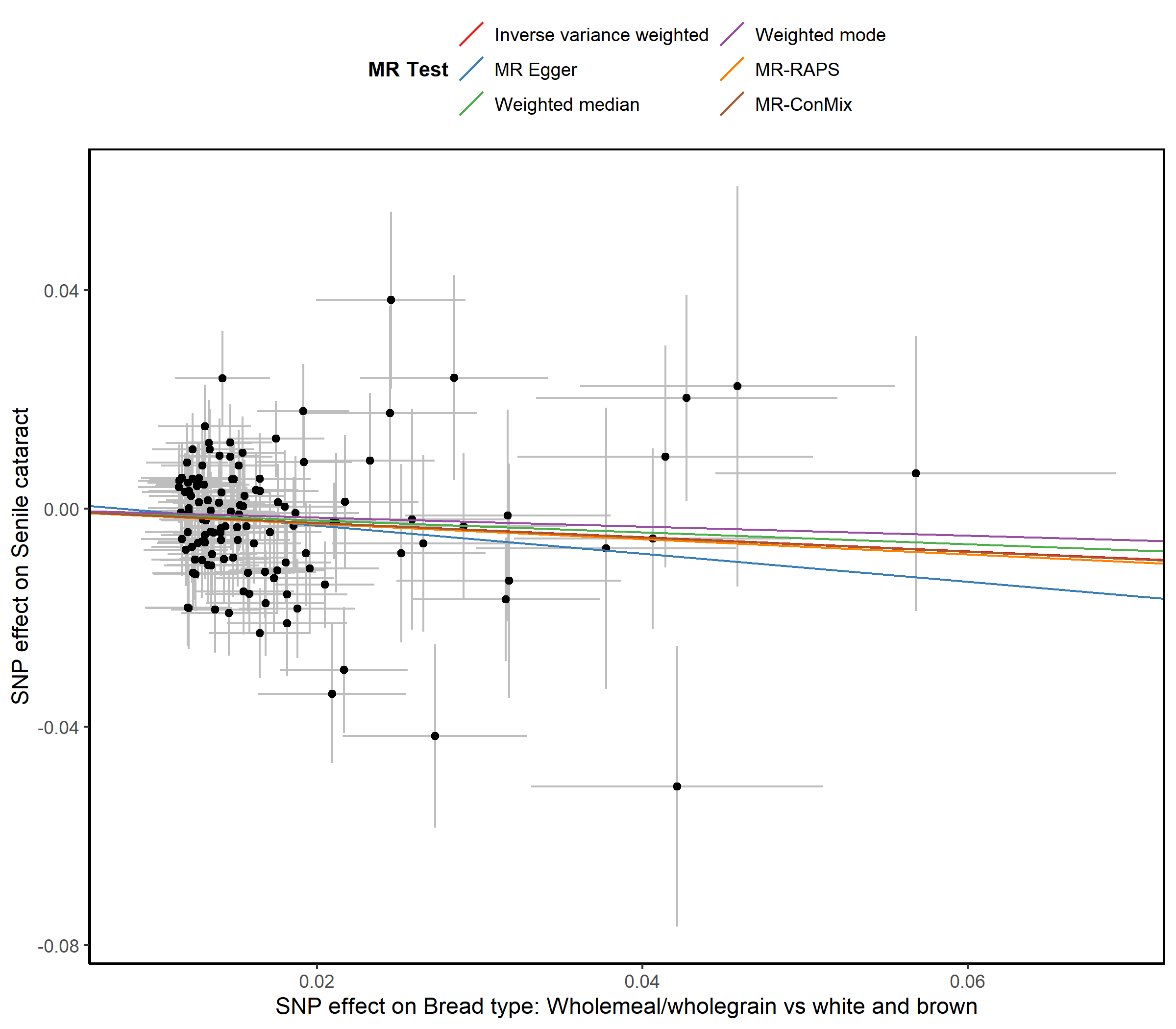


Figure S1.4 Scatter plot of SNPs associated with Bread type: wholemeal/wholegrain vs white and brown on SC.


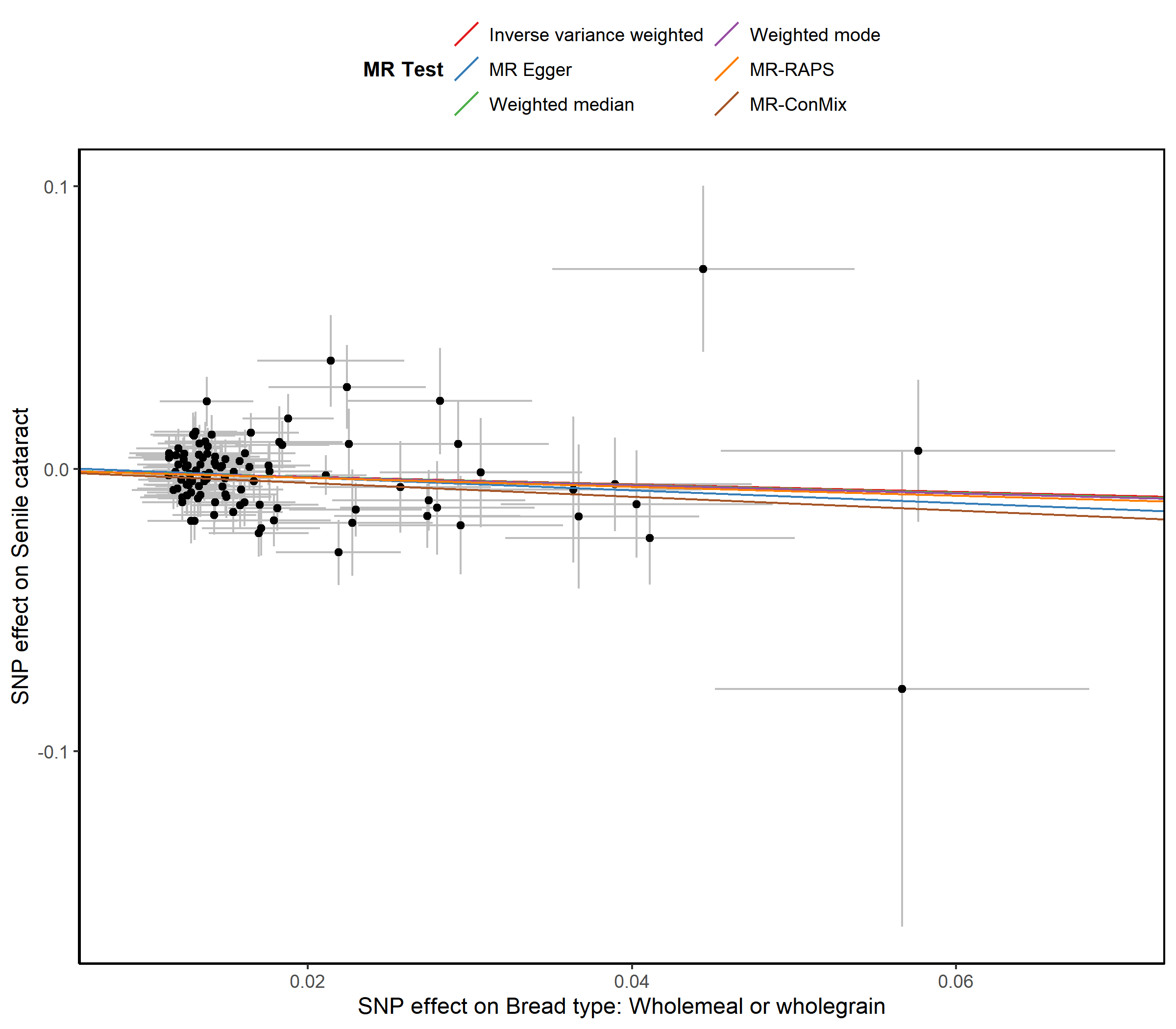


Figure S1.5 Scatter plot of SNPs associated with Bread type: wholemeal or wholegrain on SC.


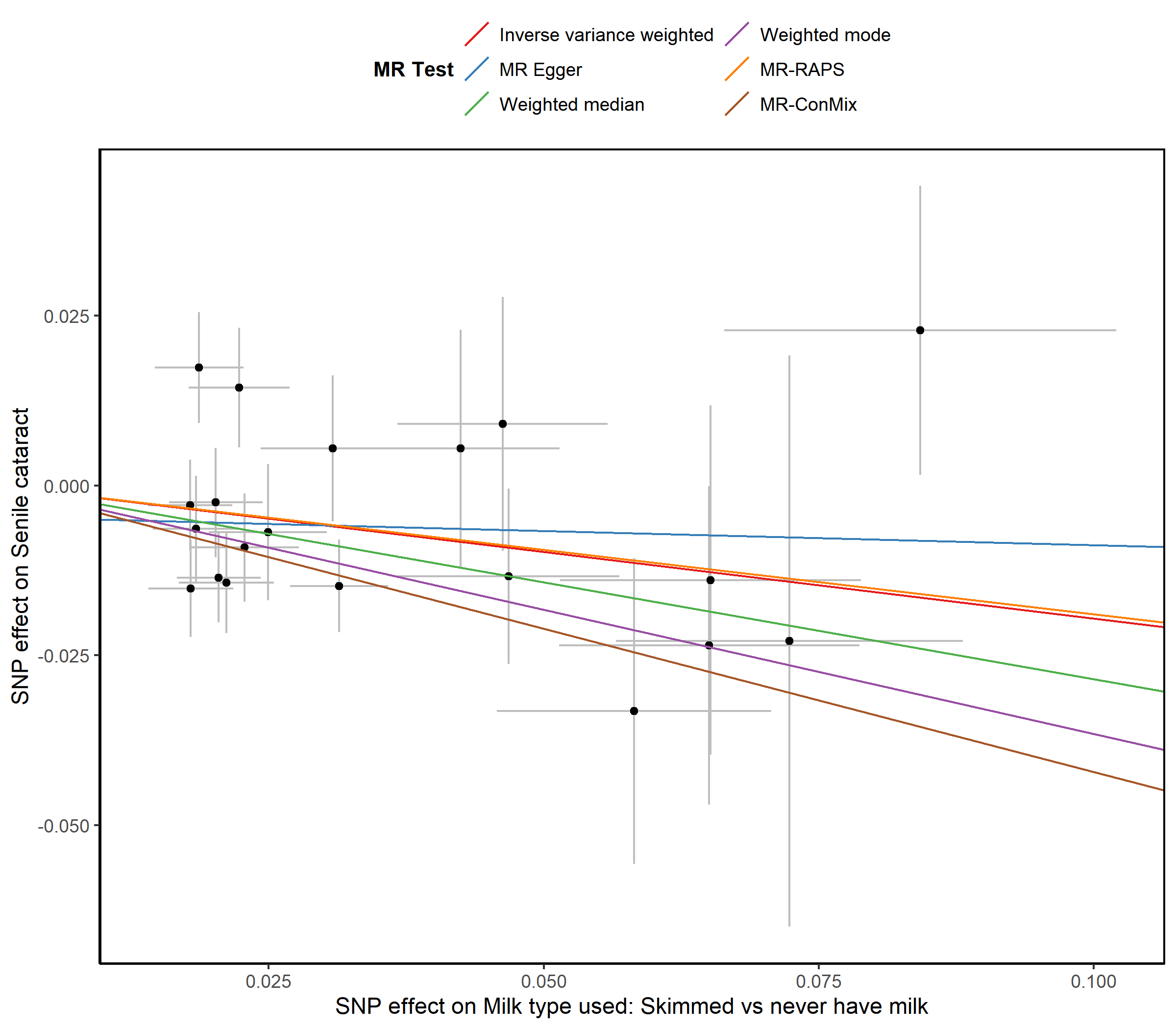


Figure S1.6 Scatter plot of SNPs associated with Milk type used: skimmed vs never have milk on SC.


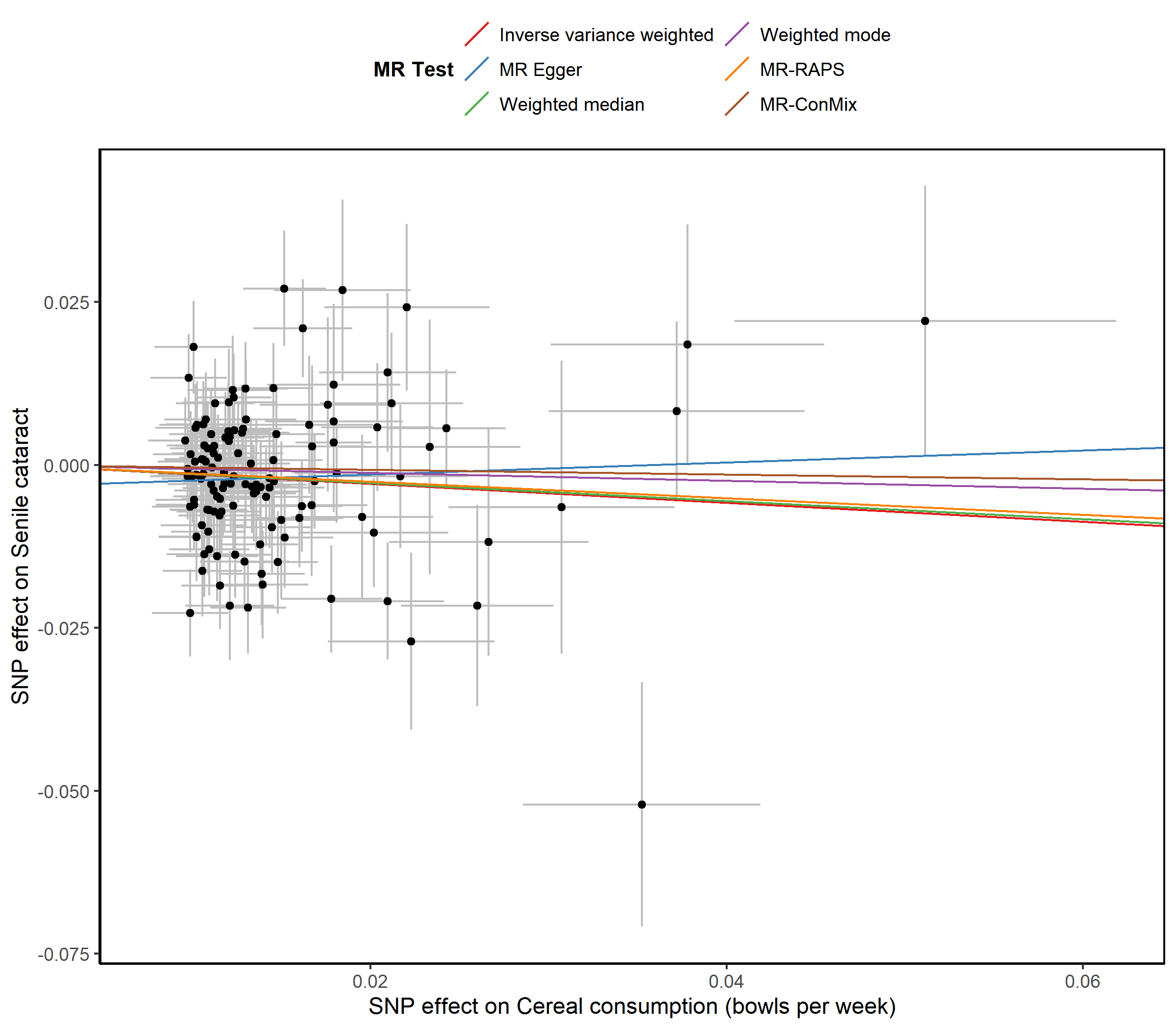


Figure S1.7 Scatter plot of SNPs associated with Cereal consumption (bowls per week) on SC.


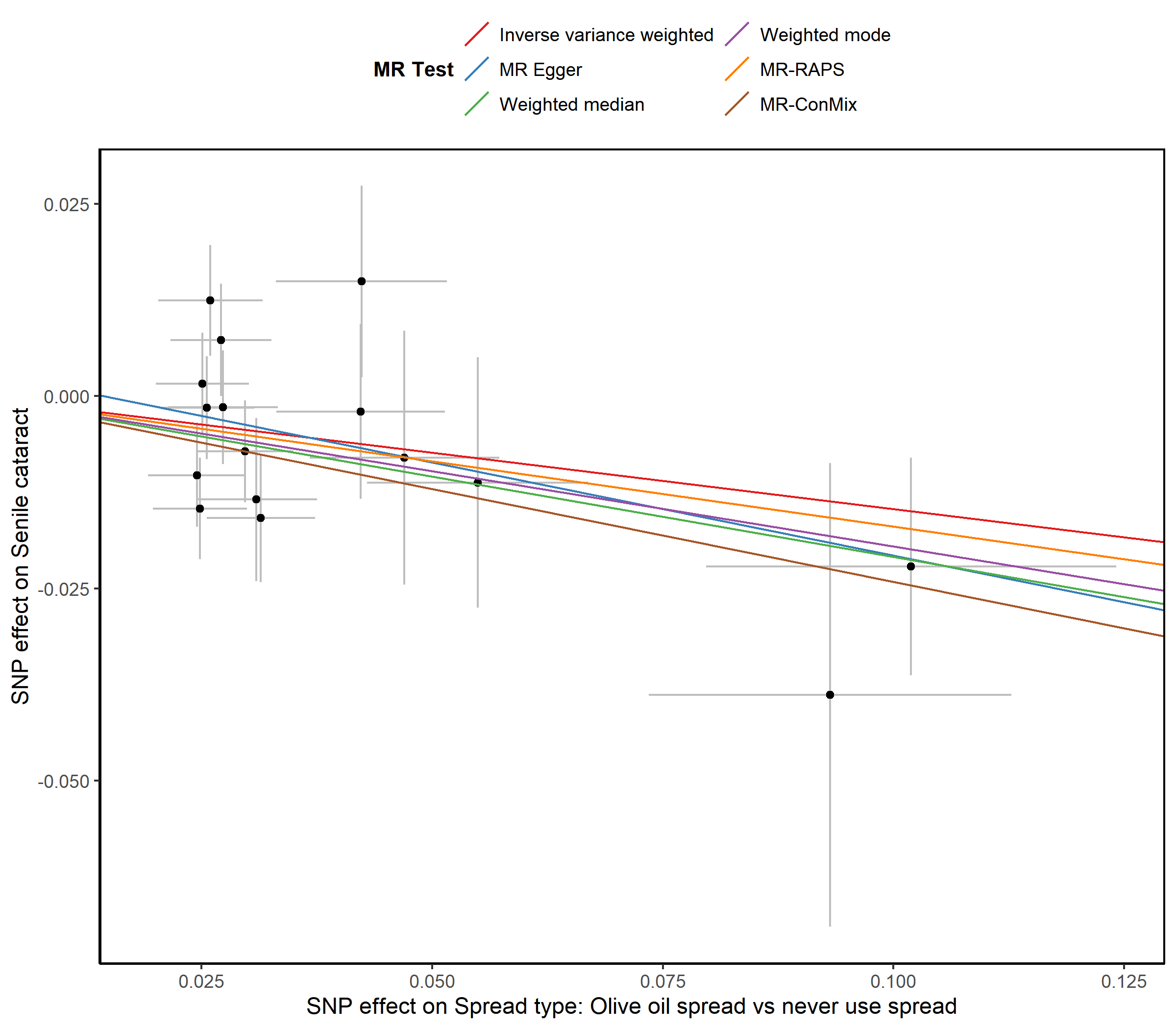


Figure S1.8 Scatter plot of SNPs associated with Spread type: olive oil spread vs never use spread on SC.


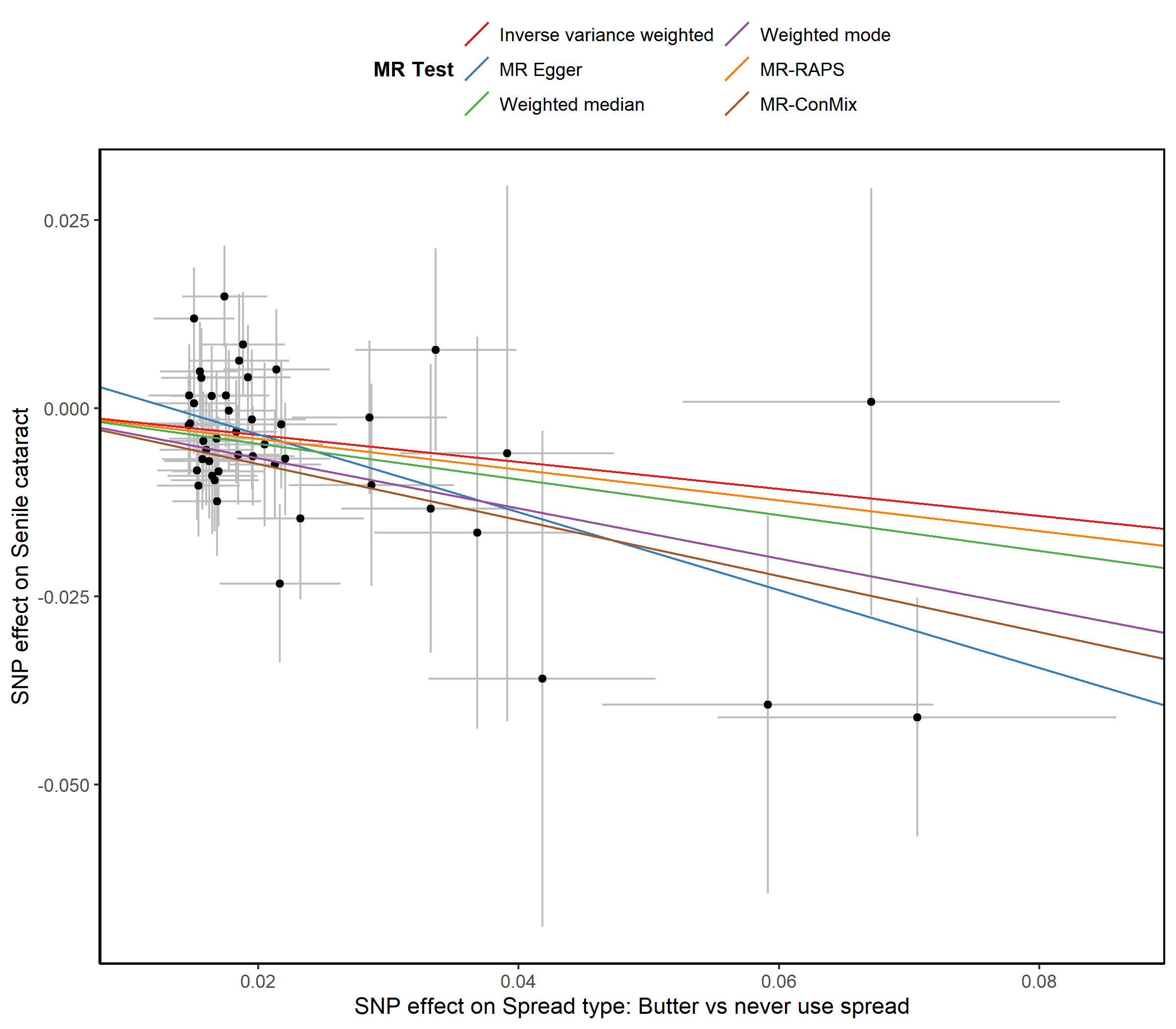


Figure S1.9 Scatter plot of SNPs associated with Spread type: butter vs never use spread on SC.


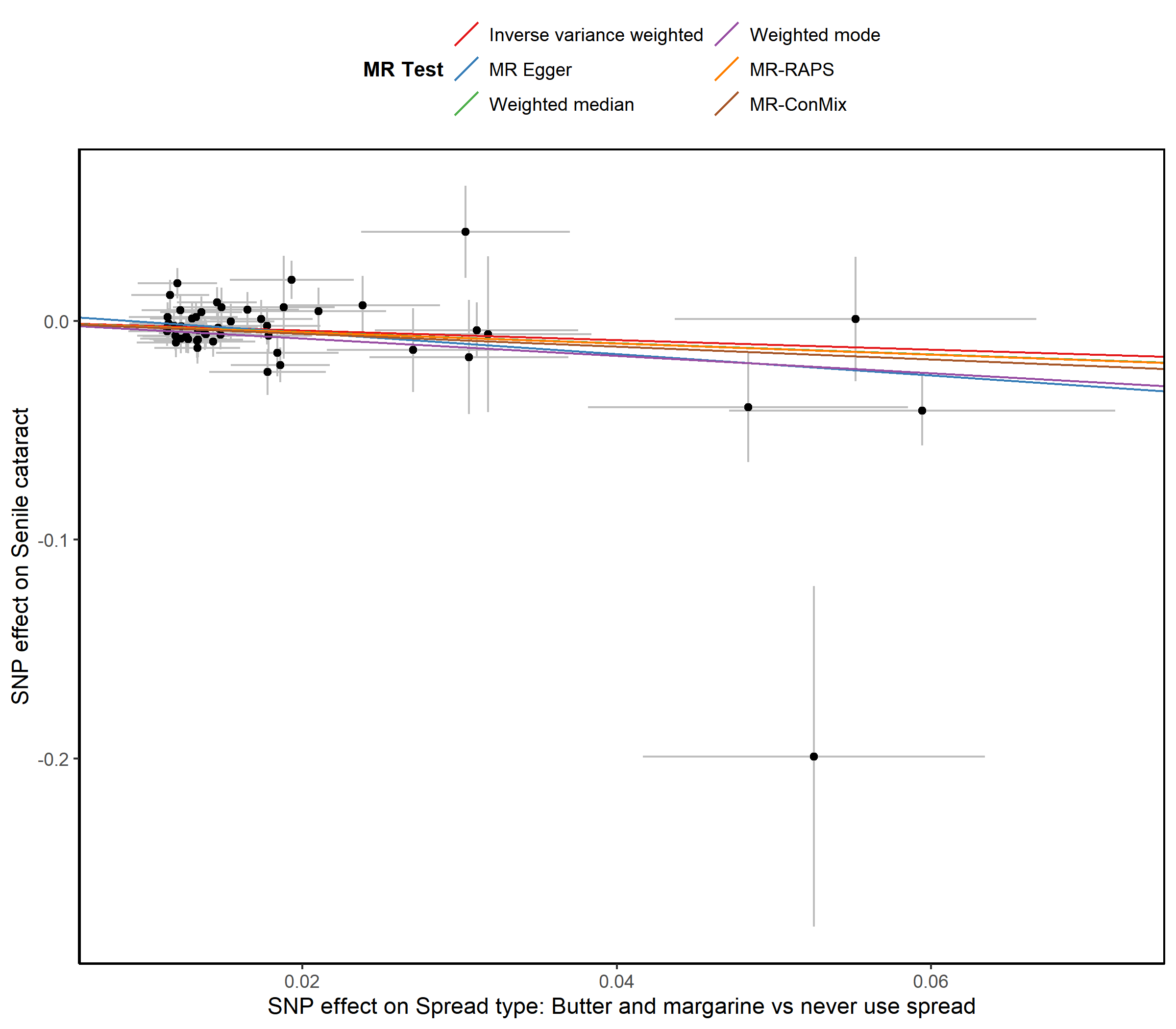


Figure S1.10 Scatter plot of SNPs associated with Spread type: butter and margarine vs never using spread on SC.


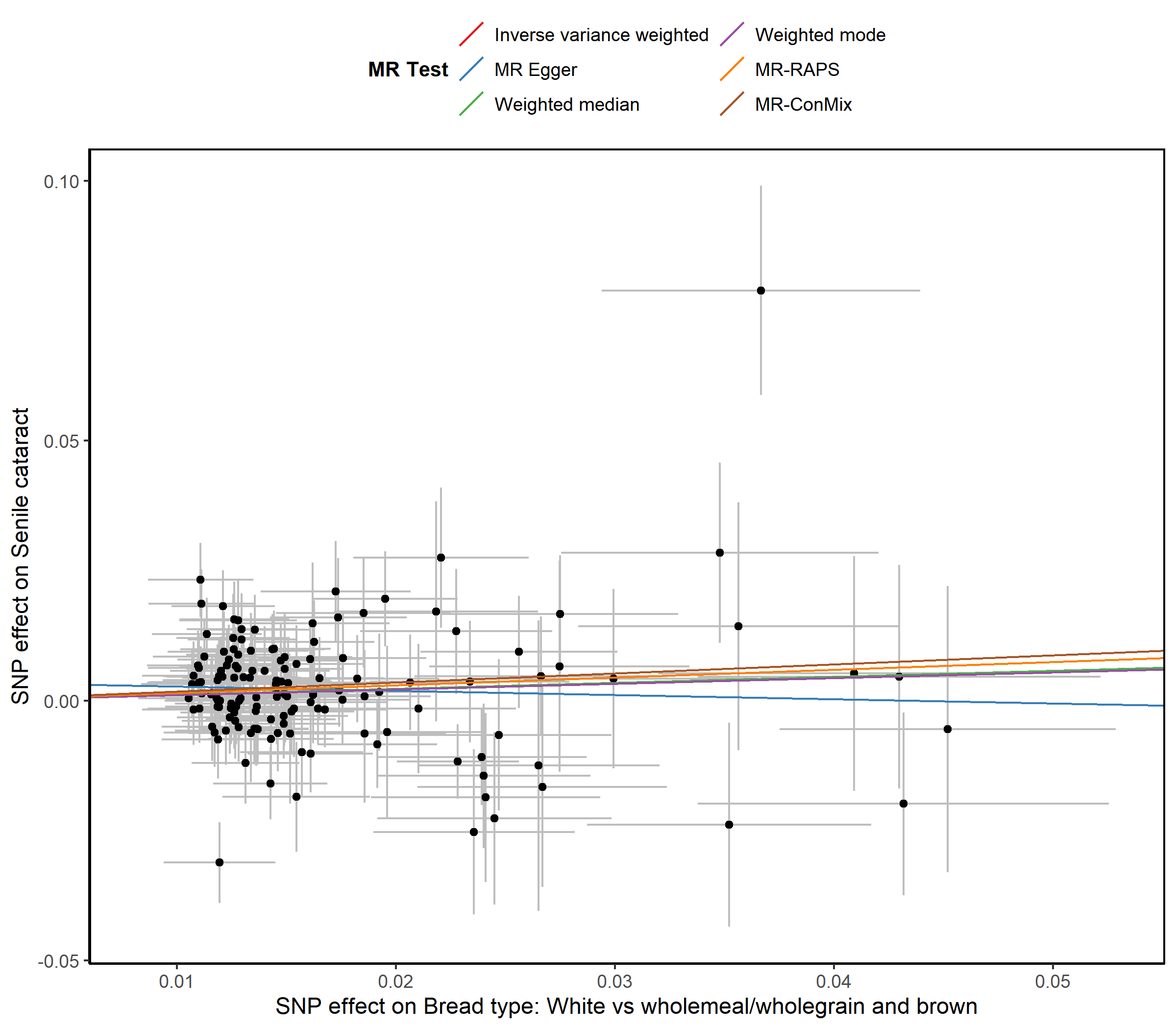


Figure S1.11 Scatter plot of SNPs associated with Bread type: white vs wholemeal/wholegrain and brown on SC.


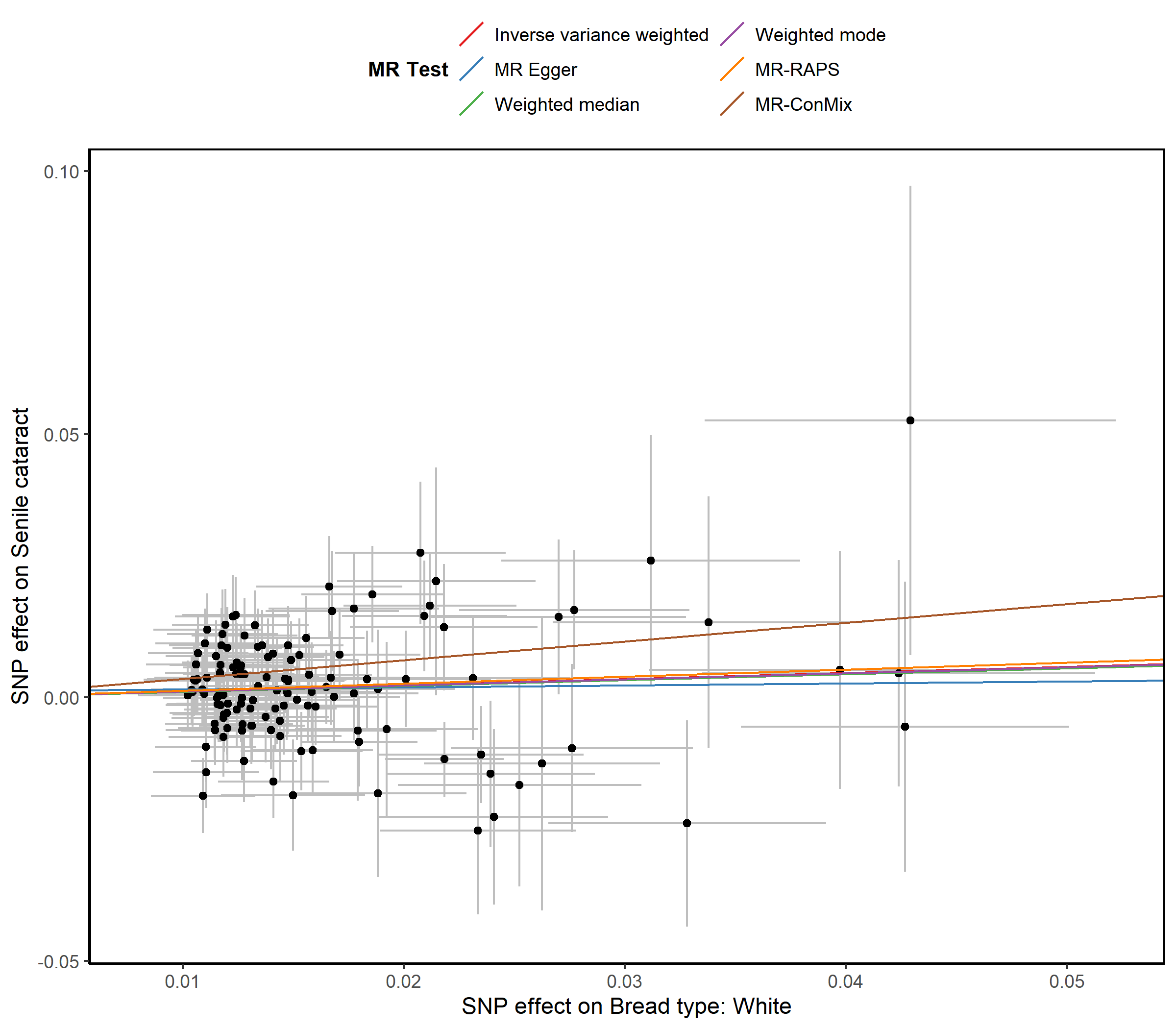


Figure S1.12 Scatter plot of SNPs associated with bread type: white on SC.


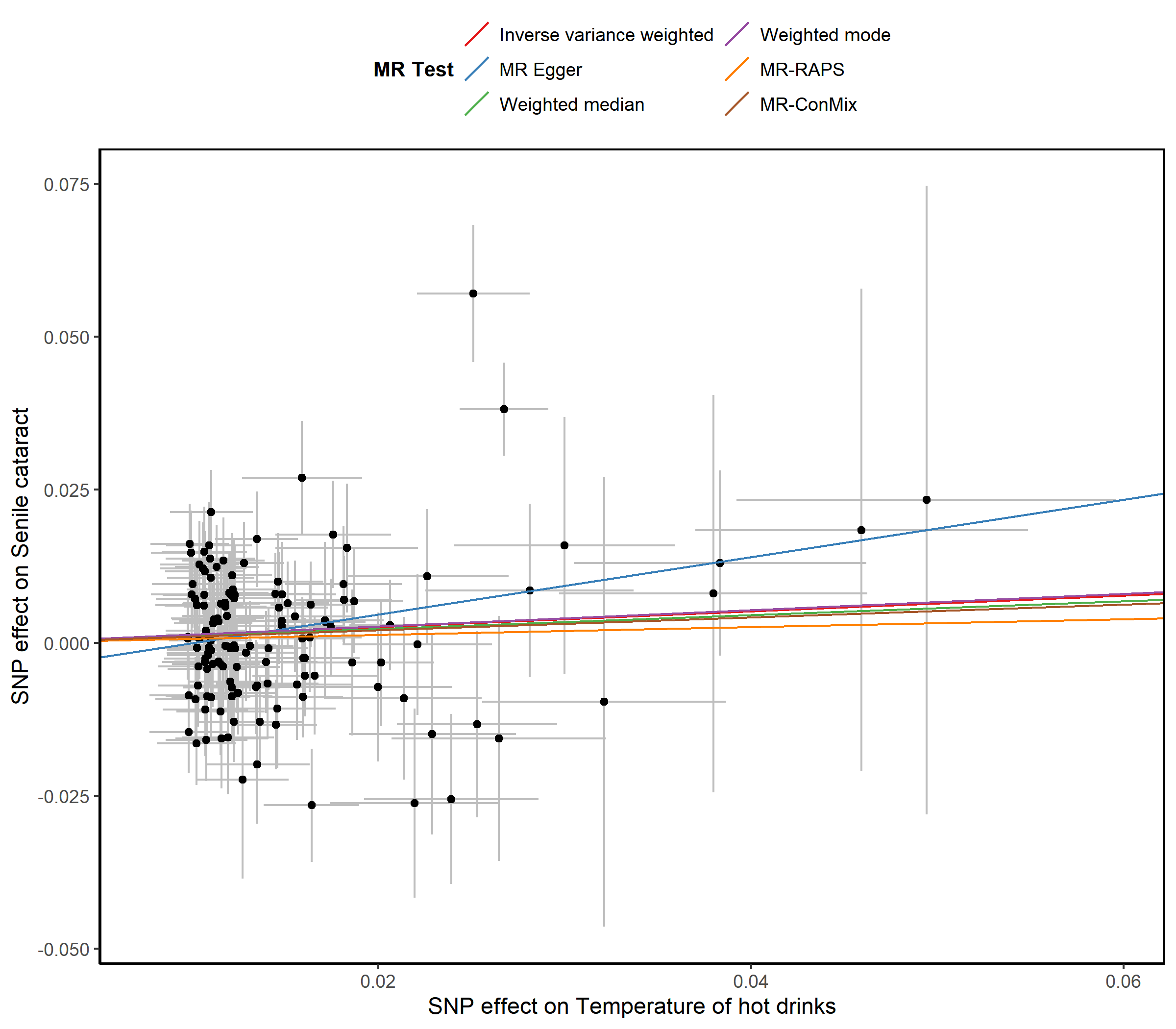


Figure S1.13 Scatter plot of SNPs associated with Temperature of hot drinks on SC.


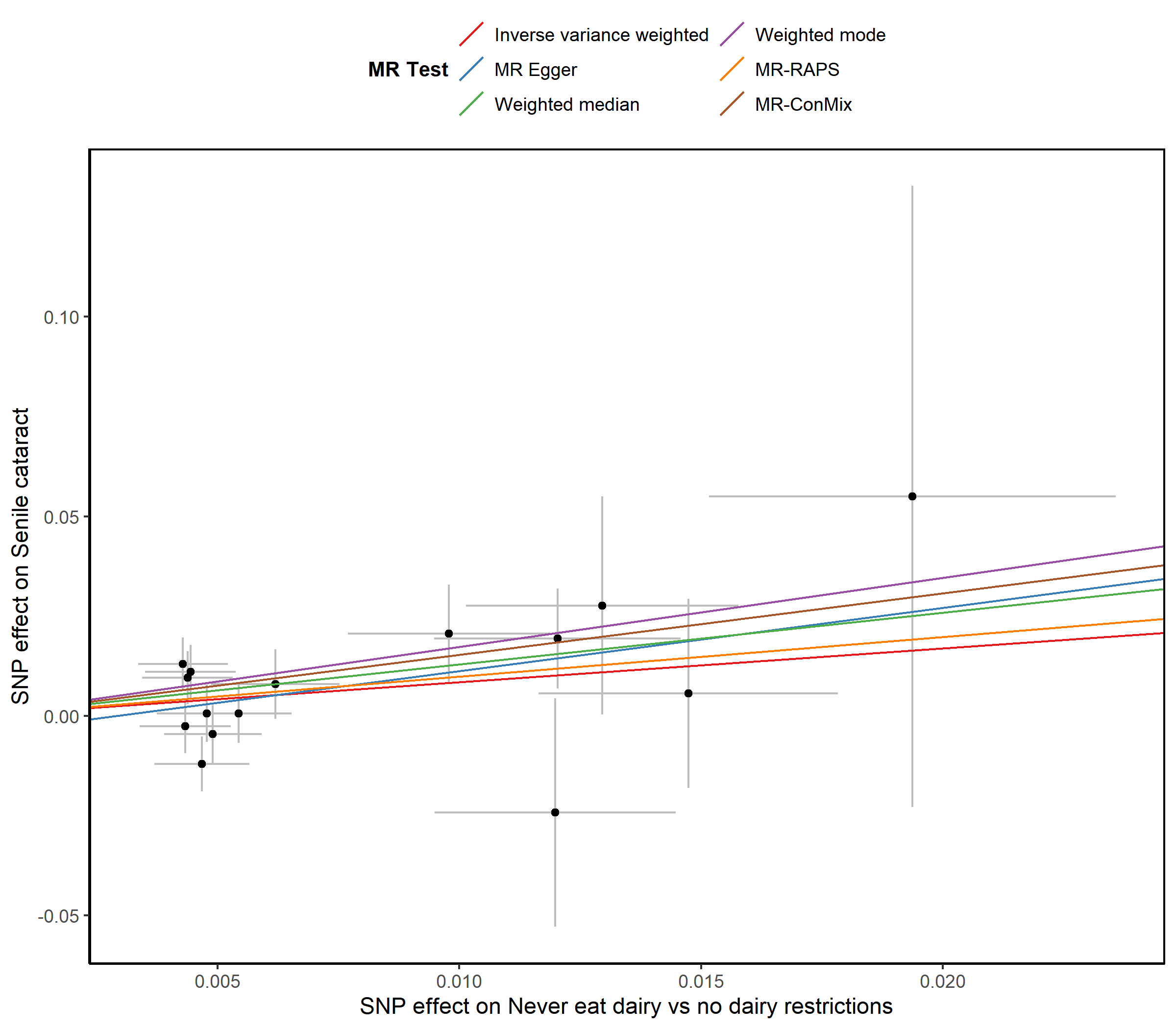


Figure S1.14 Scatter plot of SNPs associated with Never eat dairy vs no dairy restrictions on SC.


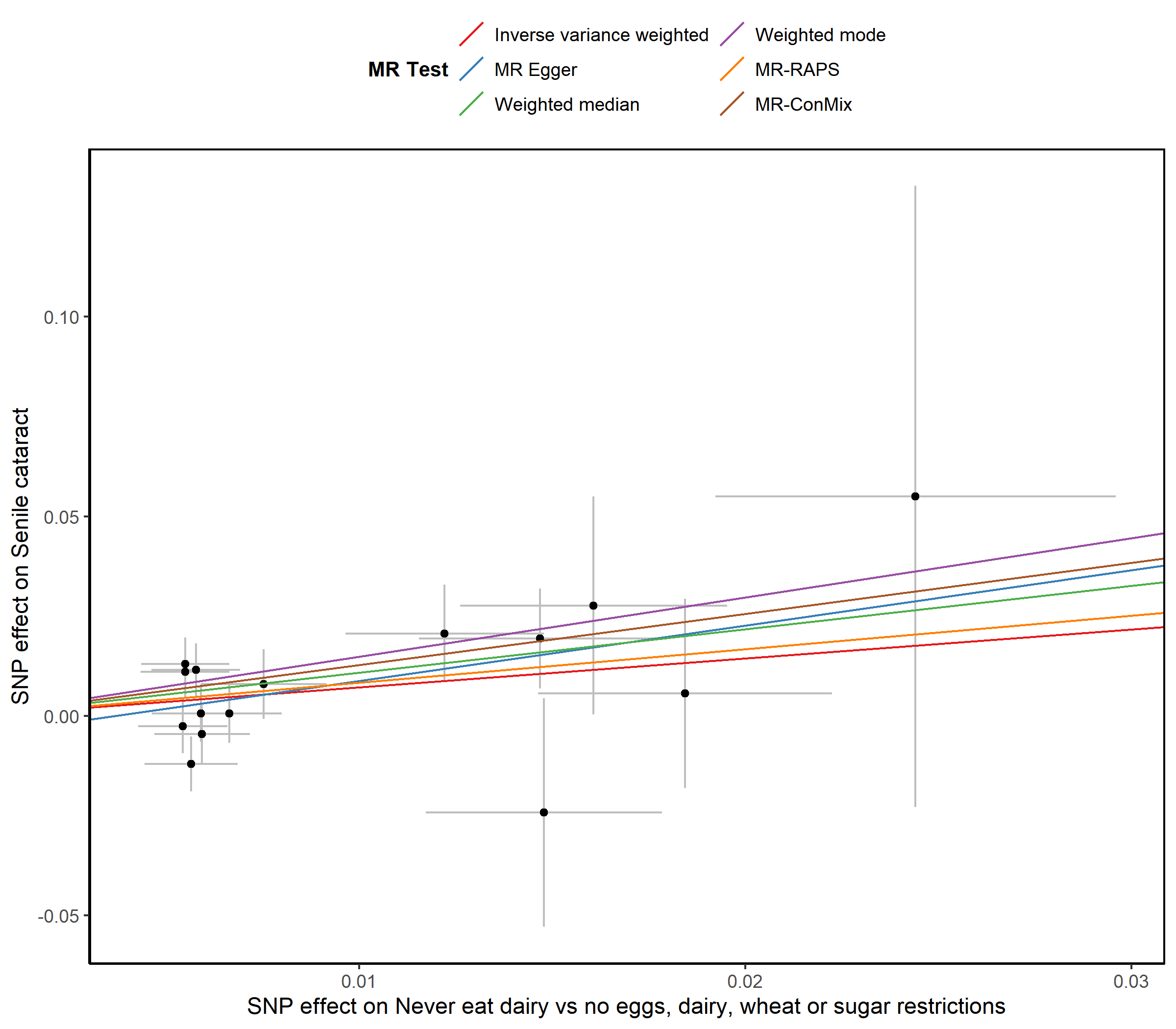


Figure S1.15 Scatter plot of SNPs associated with Never eat dairy vs no eggs, dairy, wheat, or sugar restrictions on SC.


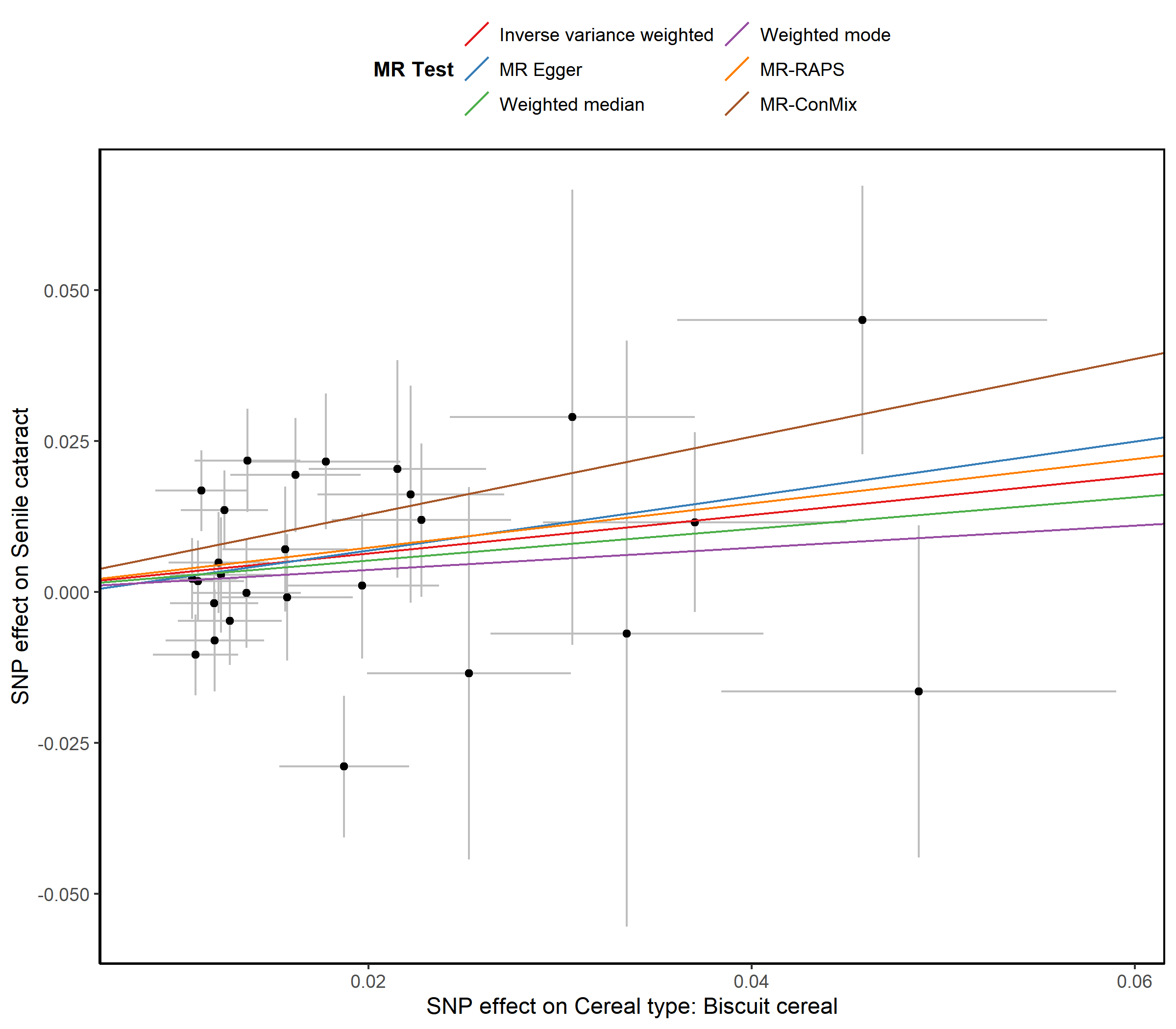


Figure S1.16 Scatter plot of SNPs associated with Cereal type: biscuit cereal on SC.

**Figure S2.** Funnel plot for the association between 16 significant dietary habits and SC in the forward analysis of bidirectional MR.


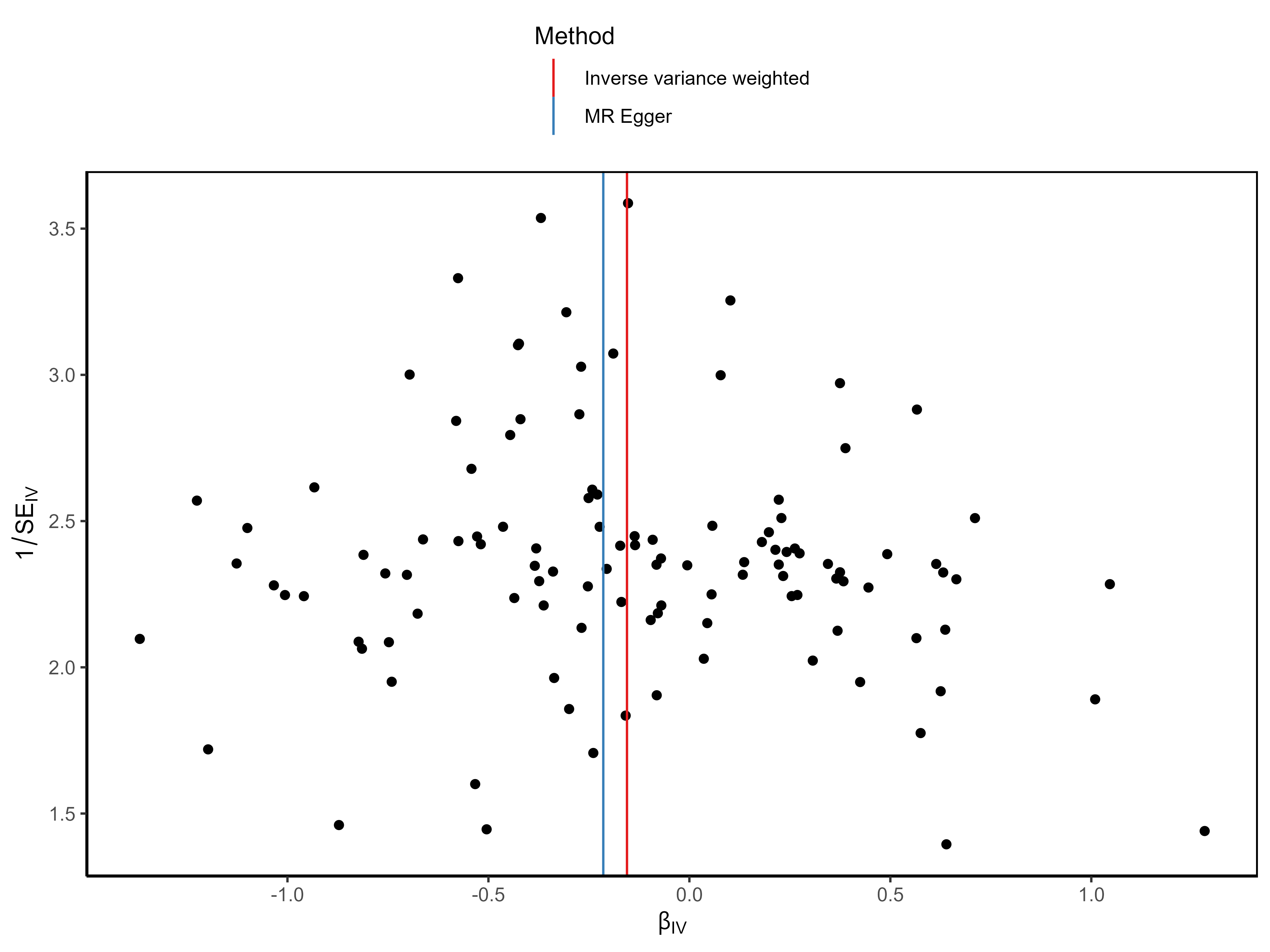


Figure S2.1 Funnel plot of SNPs associated with Drinks usually with meals in current drinkers (yes vs no) on SC.


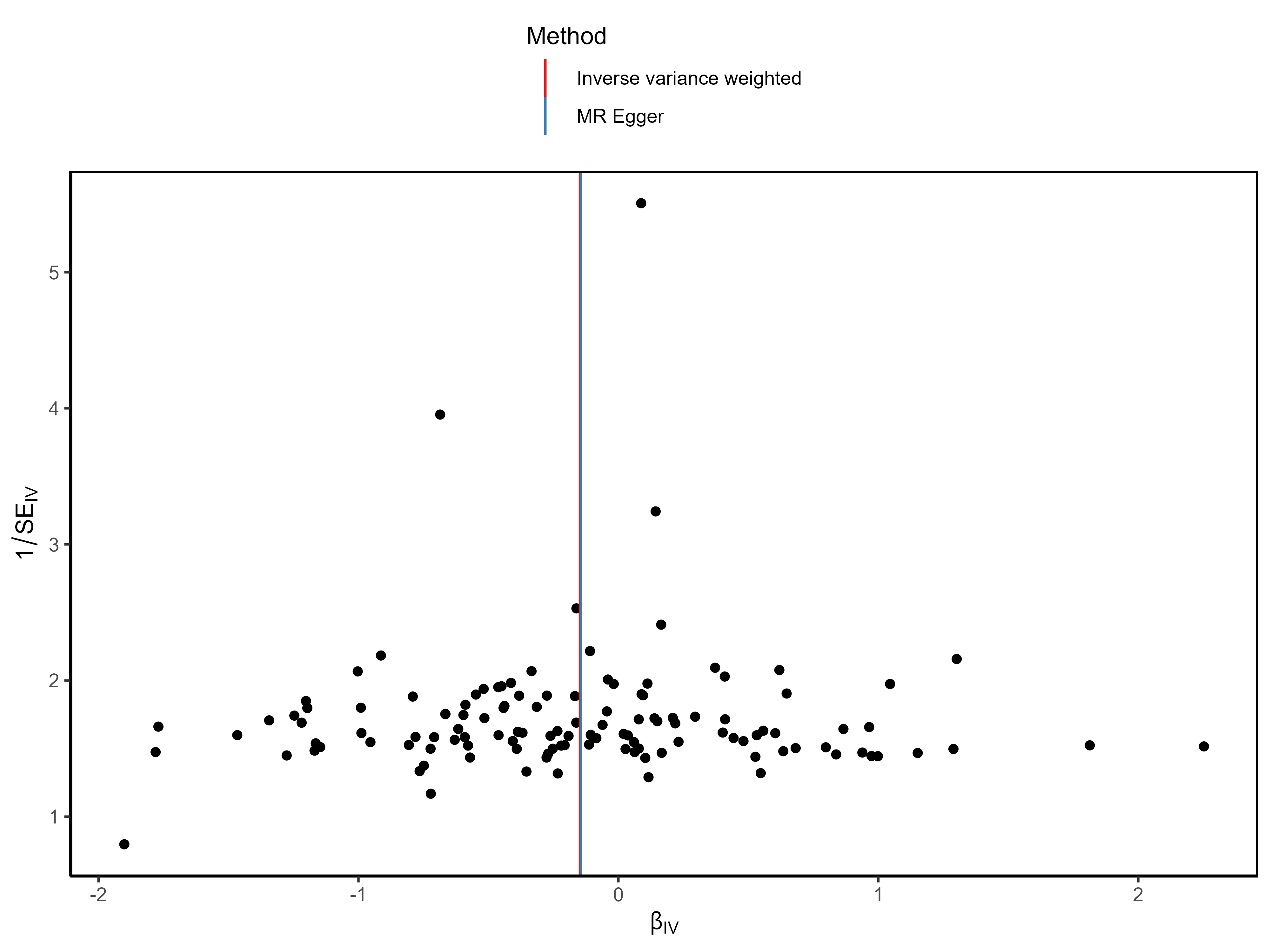


Figure S2.2 Funnel plot of SNPs associated with Cups of tea per day on SC.


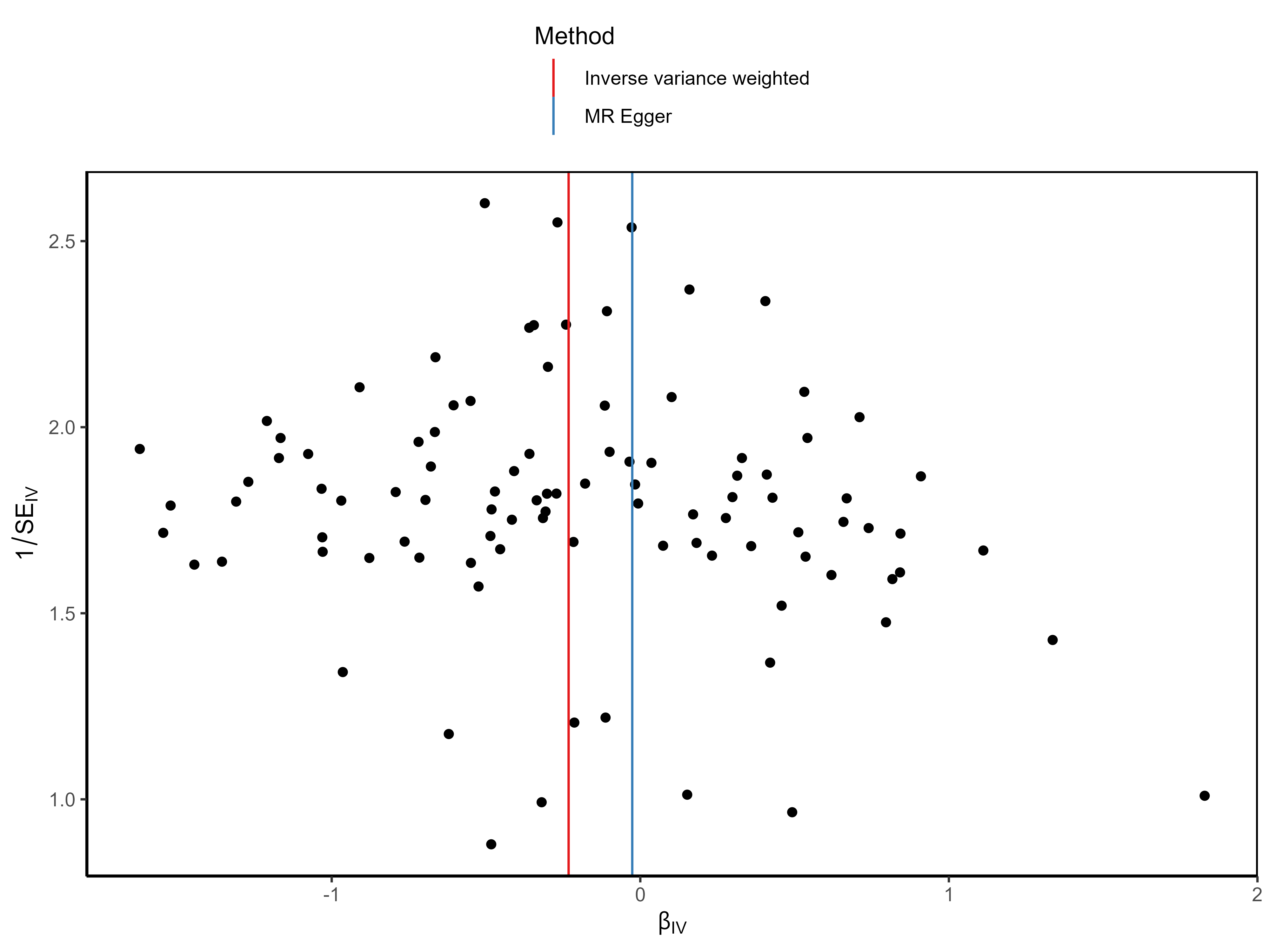


Figure S2.3 Funnel plot of SNPs associated with Alcohol usually taken with meals (yes and it varies vs no) on SC.


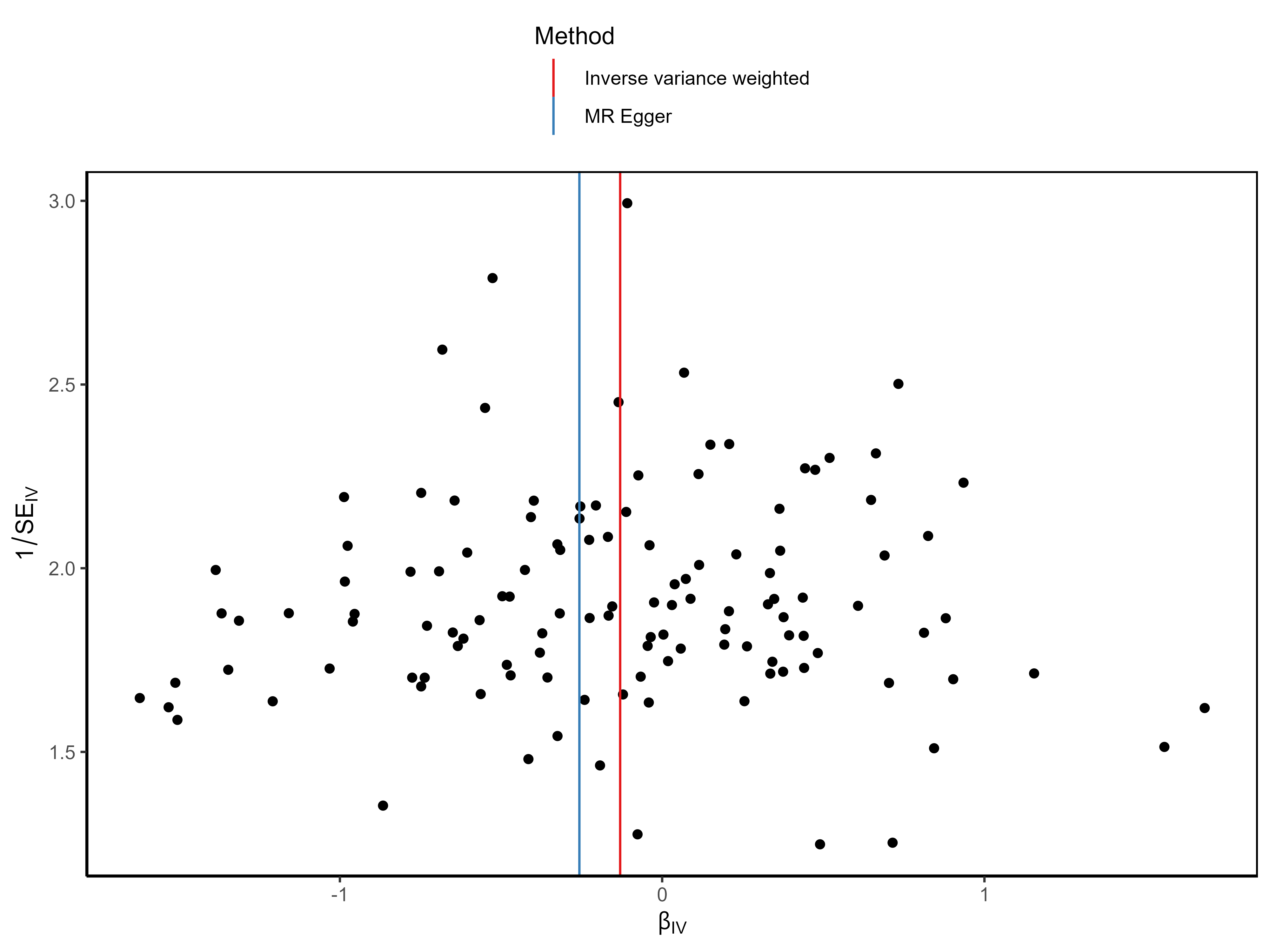


Figure S2.4 Funnel plot of SNPs associated with Bread type: wholemeal/wholegrain vs white and brown on SC.


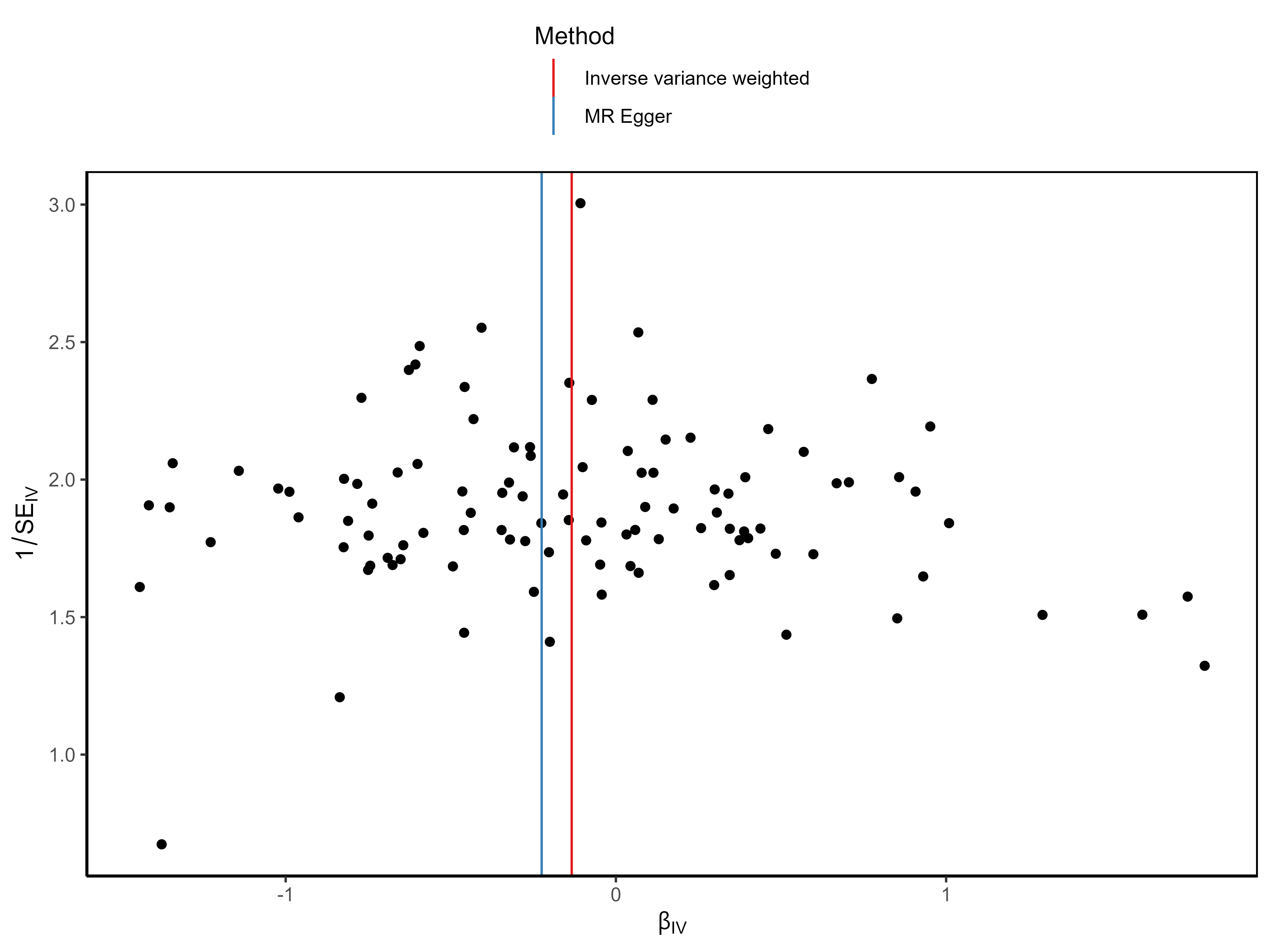


Figure S2.5 Funnel plot of SNPs associated with bread type: wholemeal or wholegrain on SC.


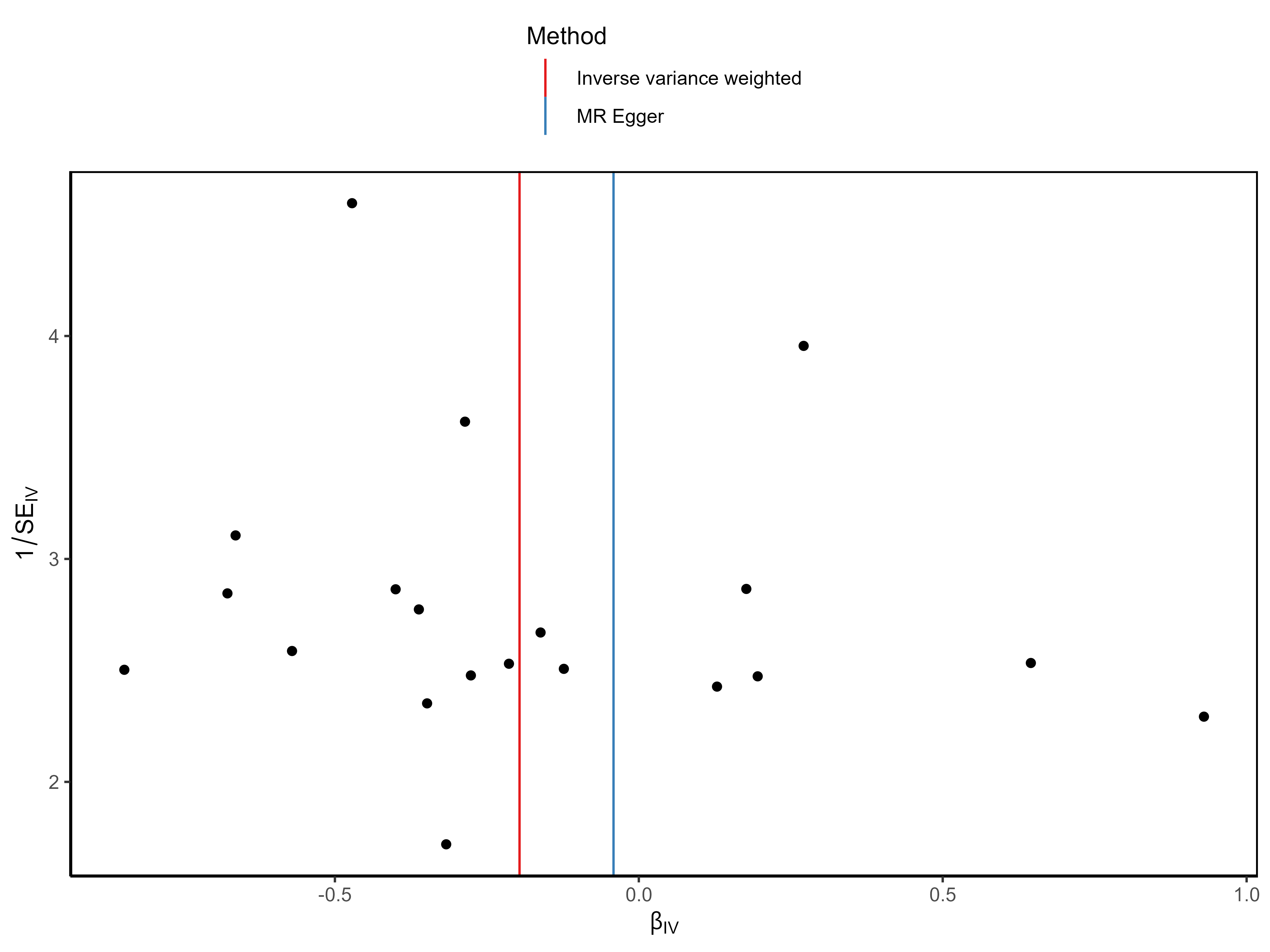


Figure S2.6 Funnel plot of SNPs associated with Milk type used: skimmed vs never have milk on SC.


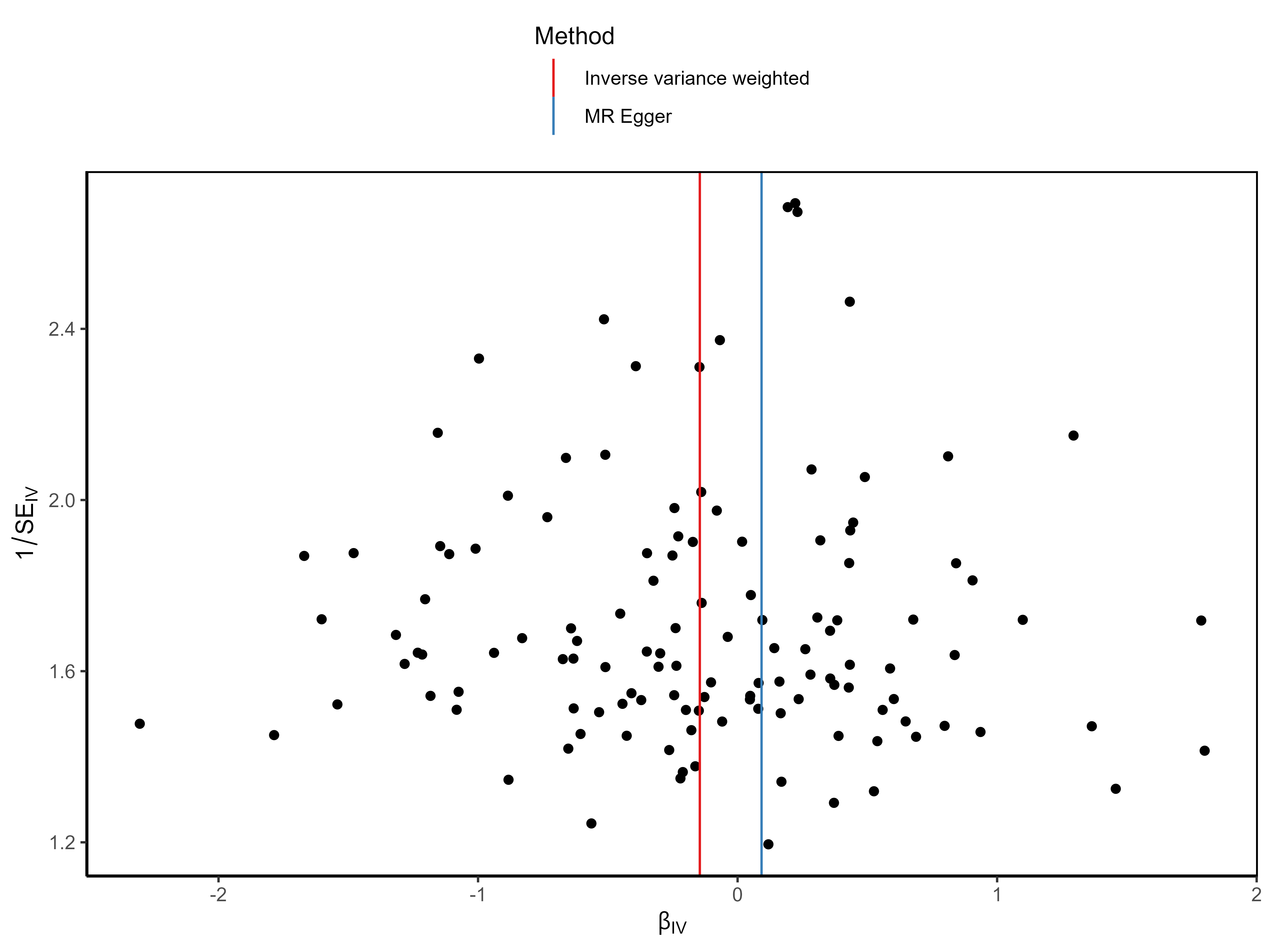


Figure S2.7 Funnel plot of SNPs associated with Cereal consumption (bowls per week) on SC.


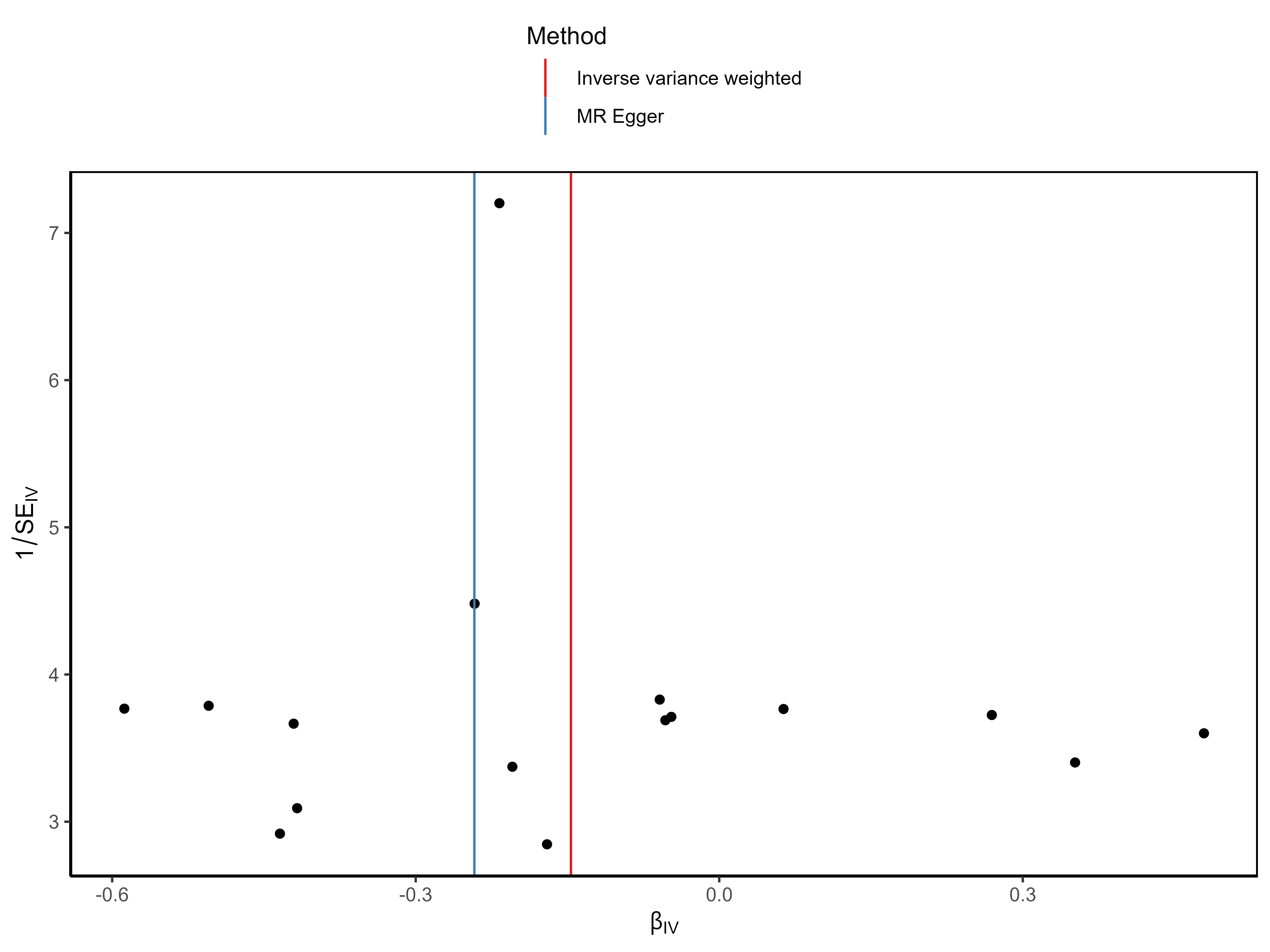


Figure S2.8 Funnel plot of SNPs associated with Spread type: olive oil spread vs never use spread on SC.


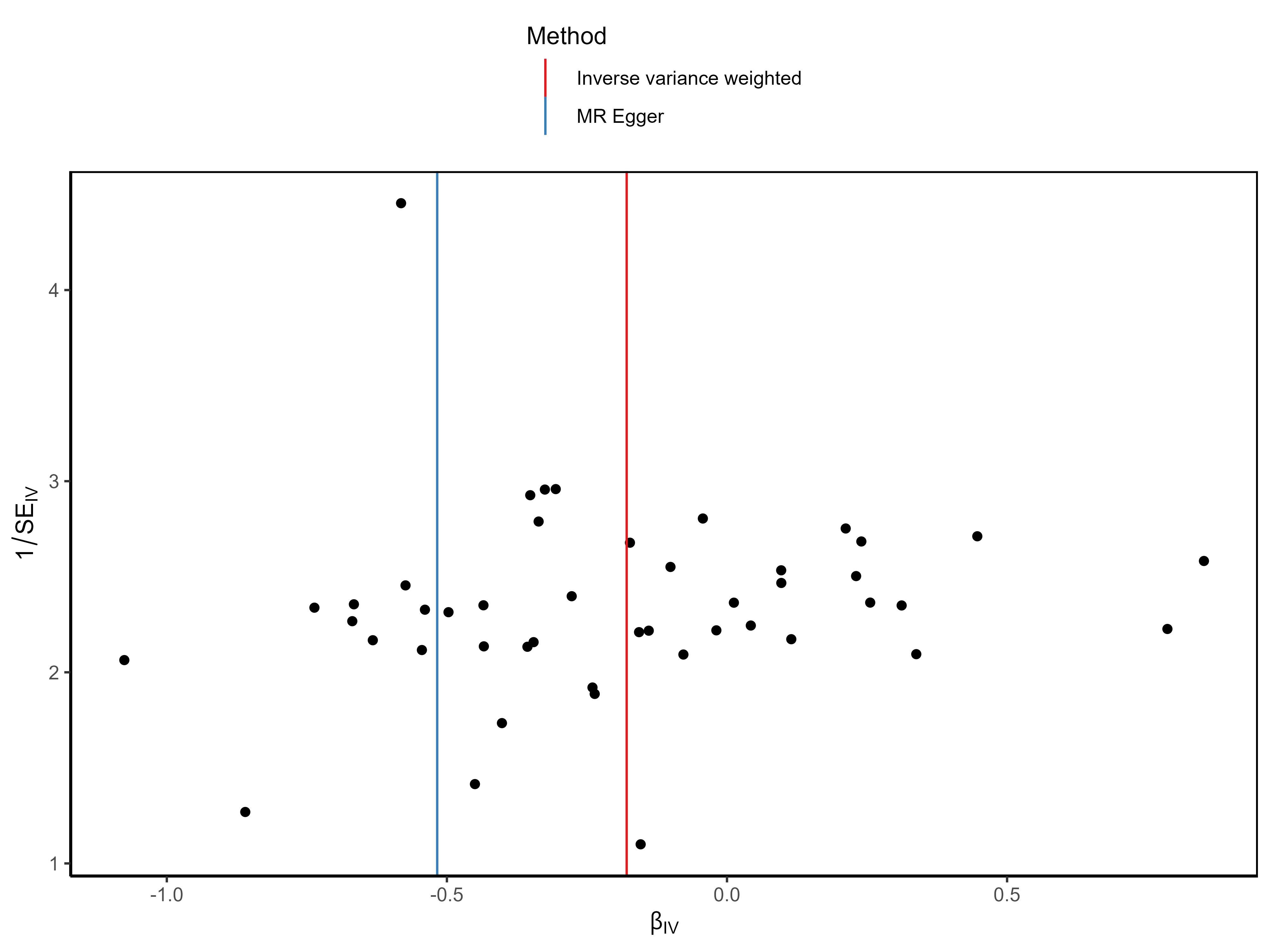


Figure S2.9 Funnel plot of SNPs associated with Spread type: butter vs never use spread on SC.


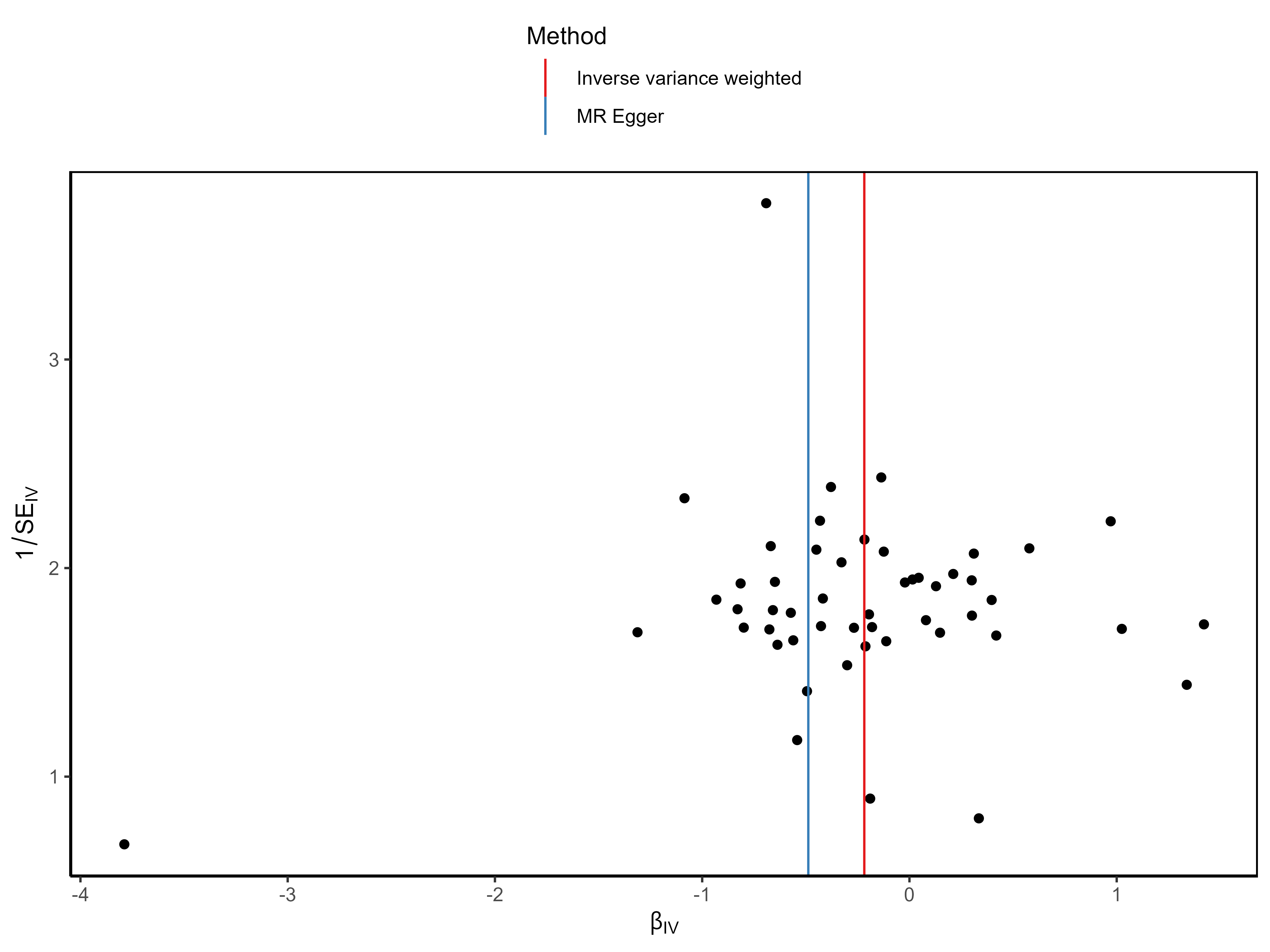


Figure S2.10 Funnel plot of SNPs associated with Spread type: butter and margarine vs never use spread on SC.


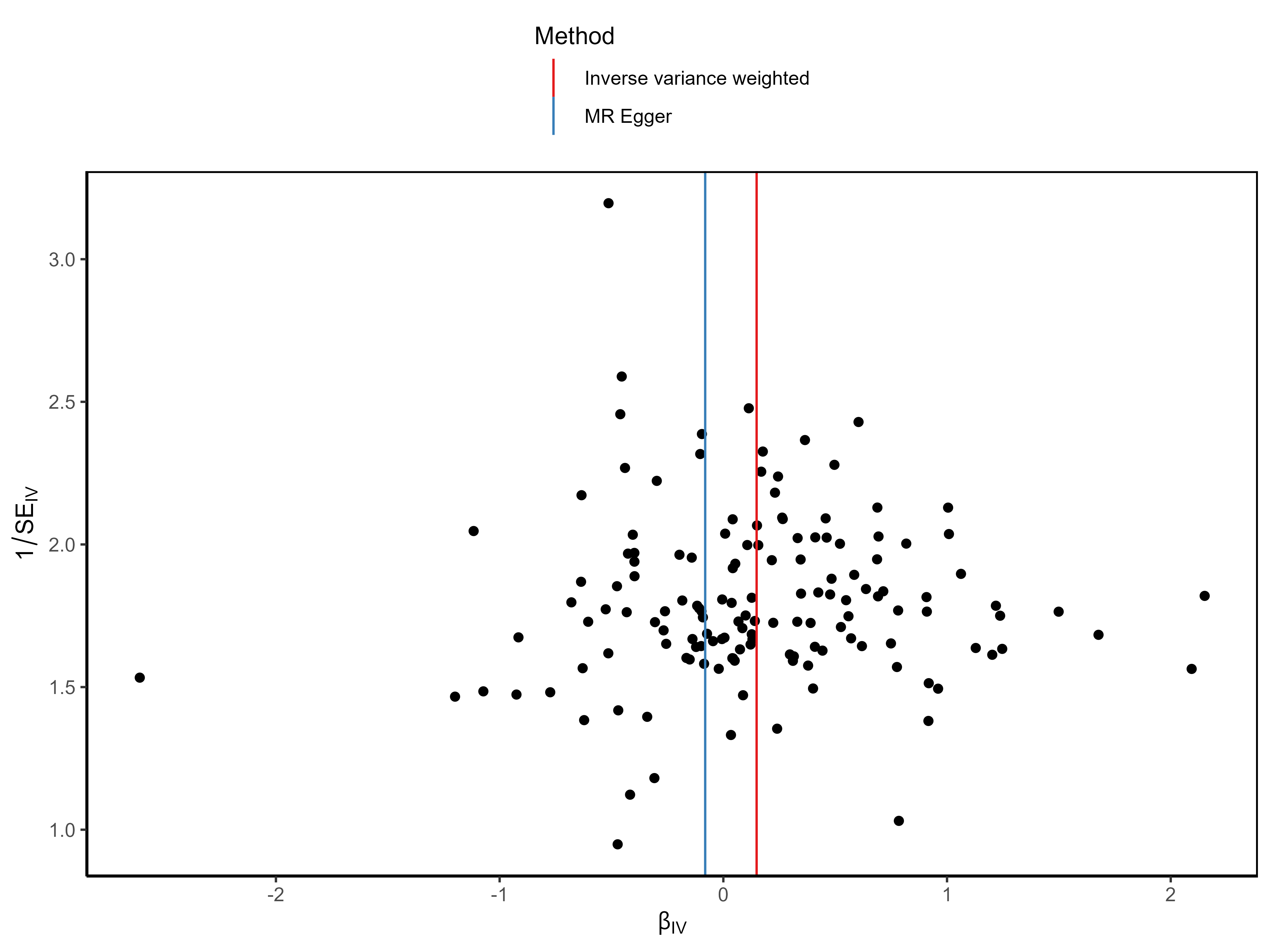


Figure S2.11 Funnel plot of SNPs associated with Bread type: white vs wholemeal/wholegrain and brown on SC.


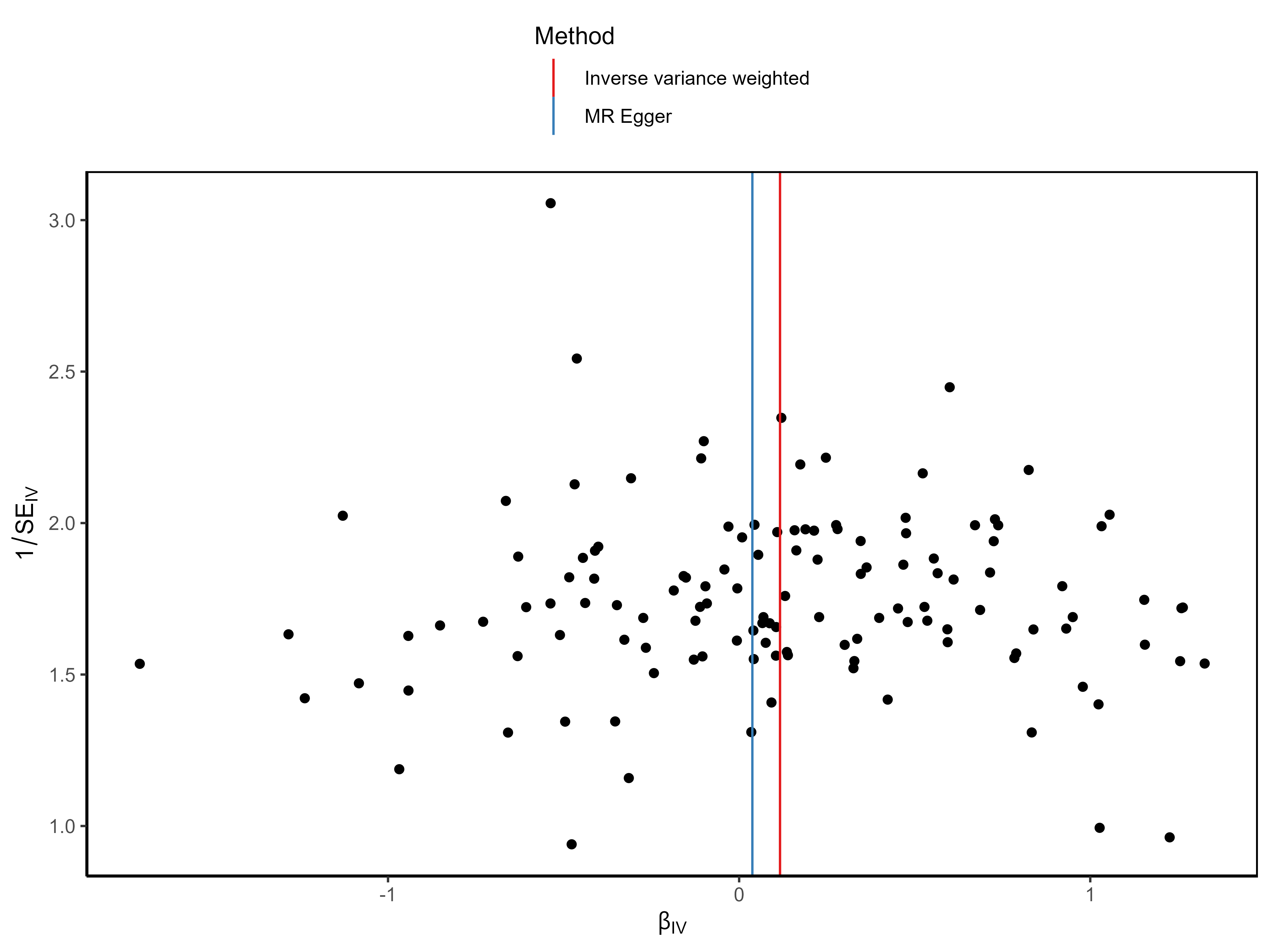


Figure S2.12 Funnel plot of SNPs associated with Bread type: white on SC.


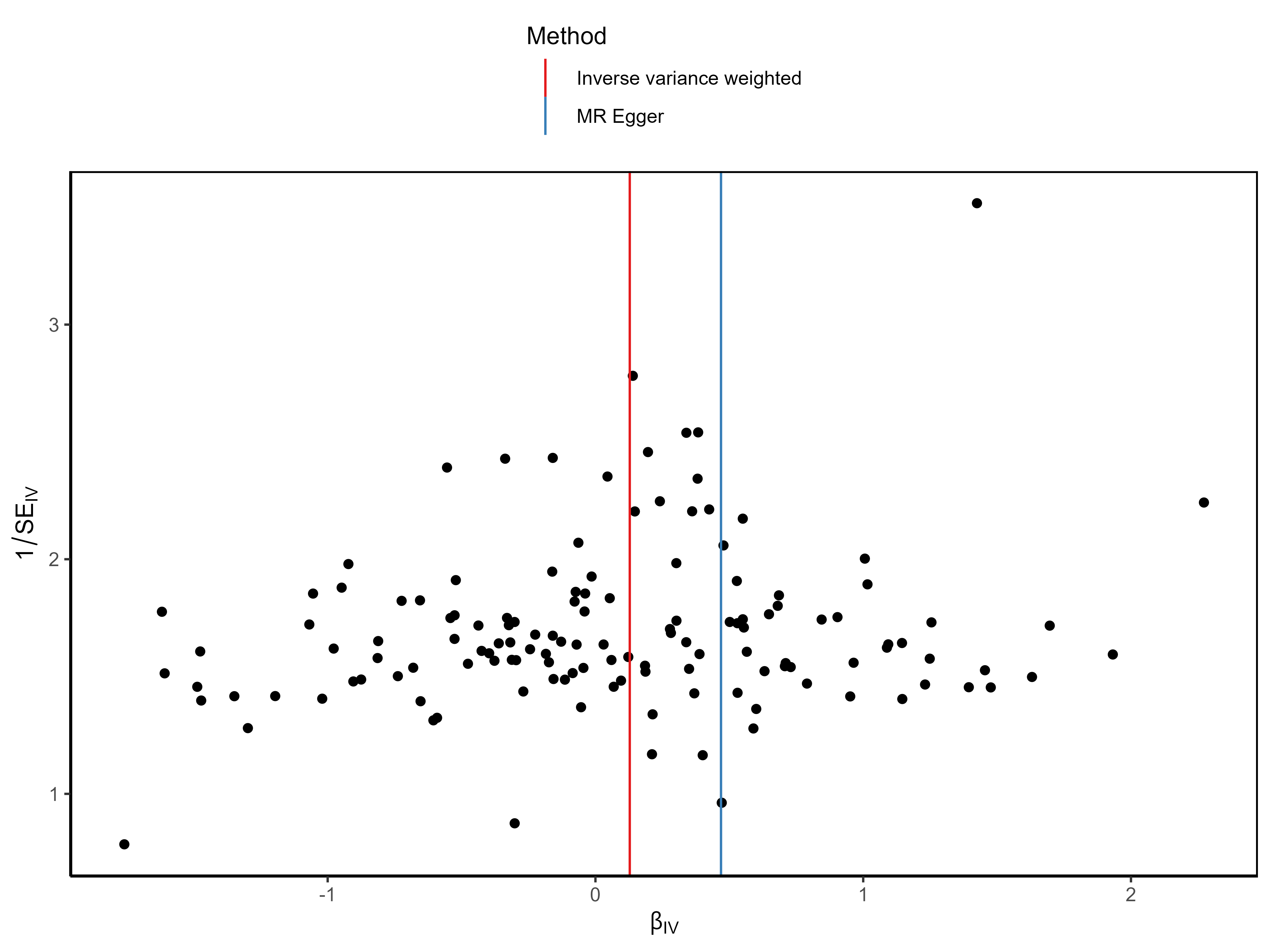


Figure S2.13 Funnel plot of SNPs associated with Temperature of hot drinks on SC.


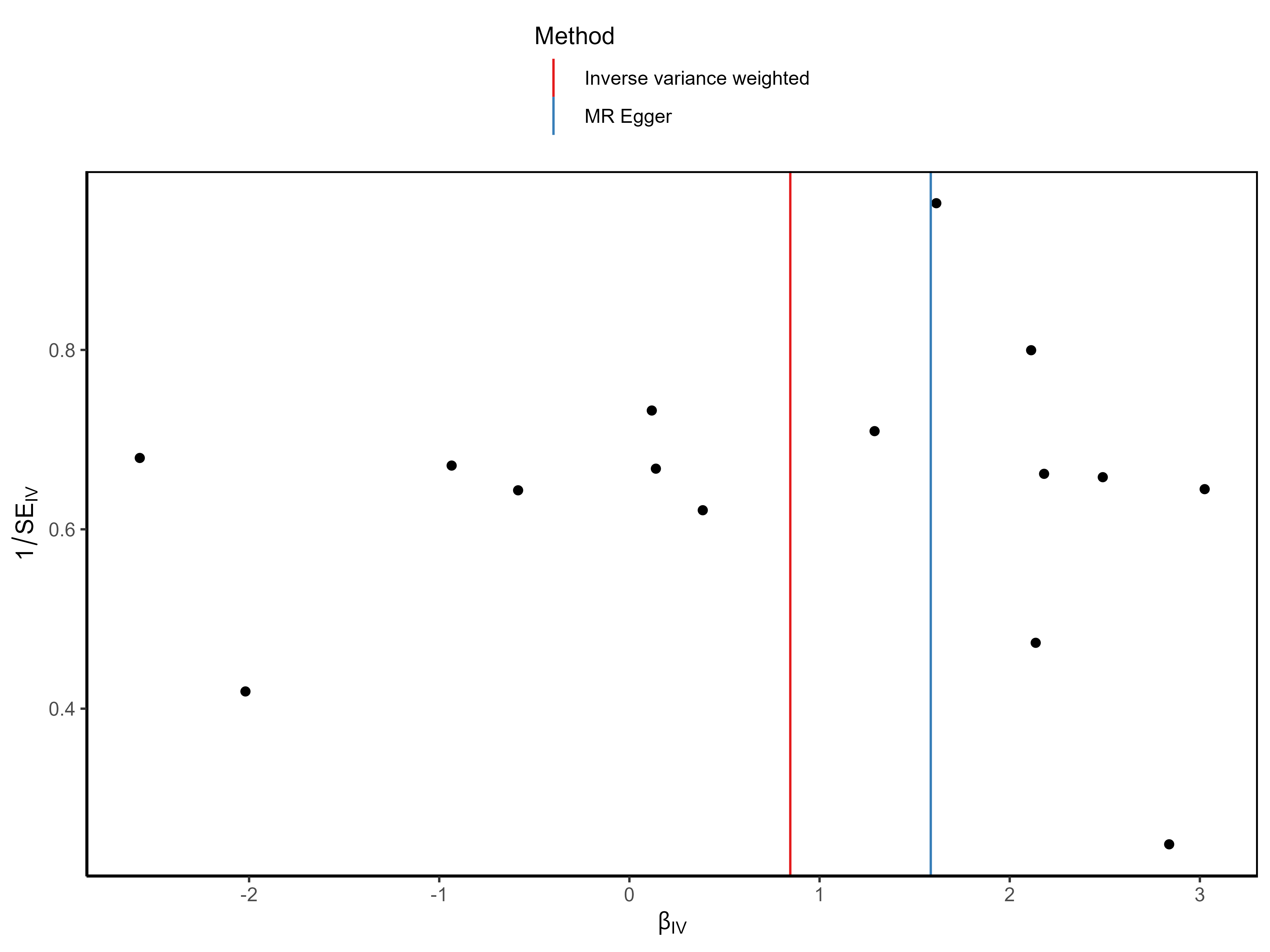


Figure S2.14 Funnel plot of SNPs associated with Never eat dairy vs no dairy restrictions on SC.


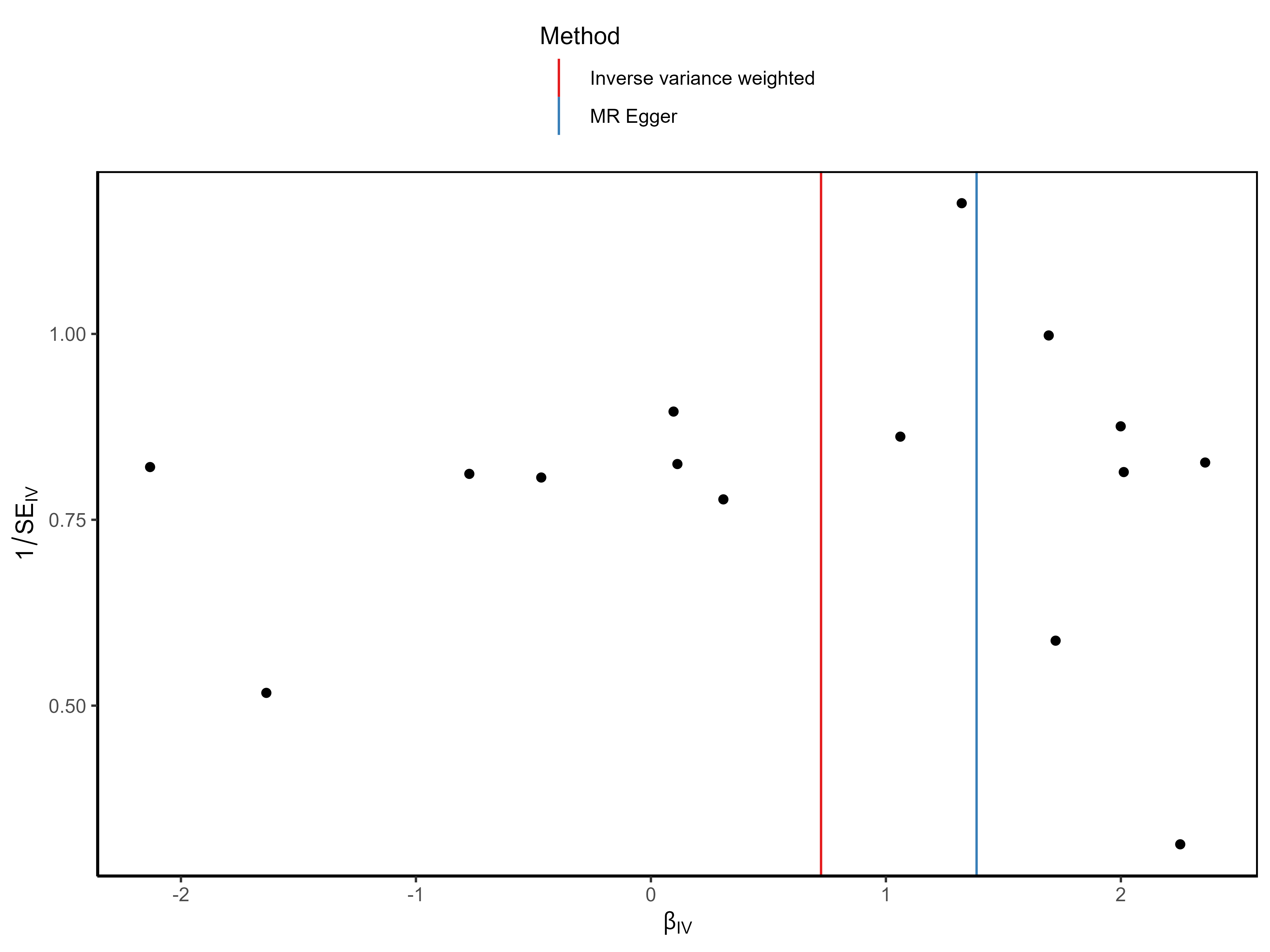


Figure S2.15 Funnel plot of SNPs associated with Never eat dairy vs no eggs, dairy, wheat, or sugar restrictions on SC.


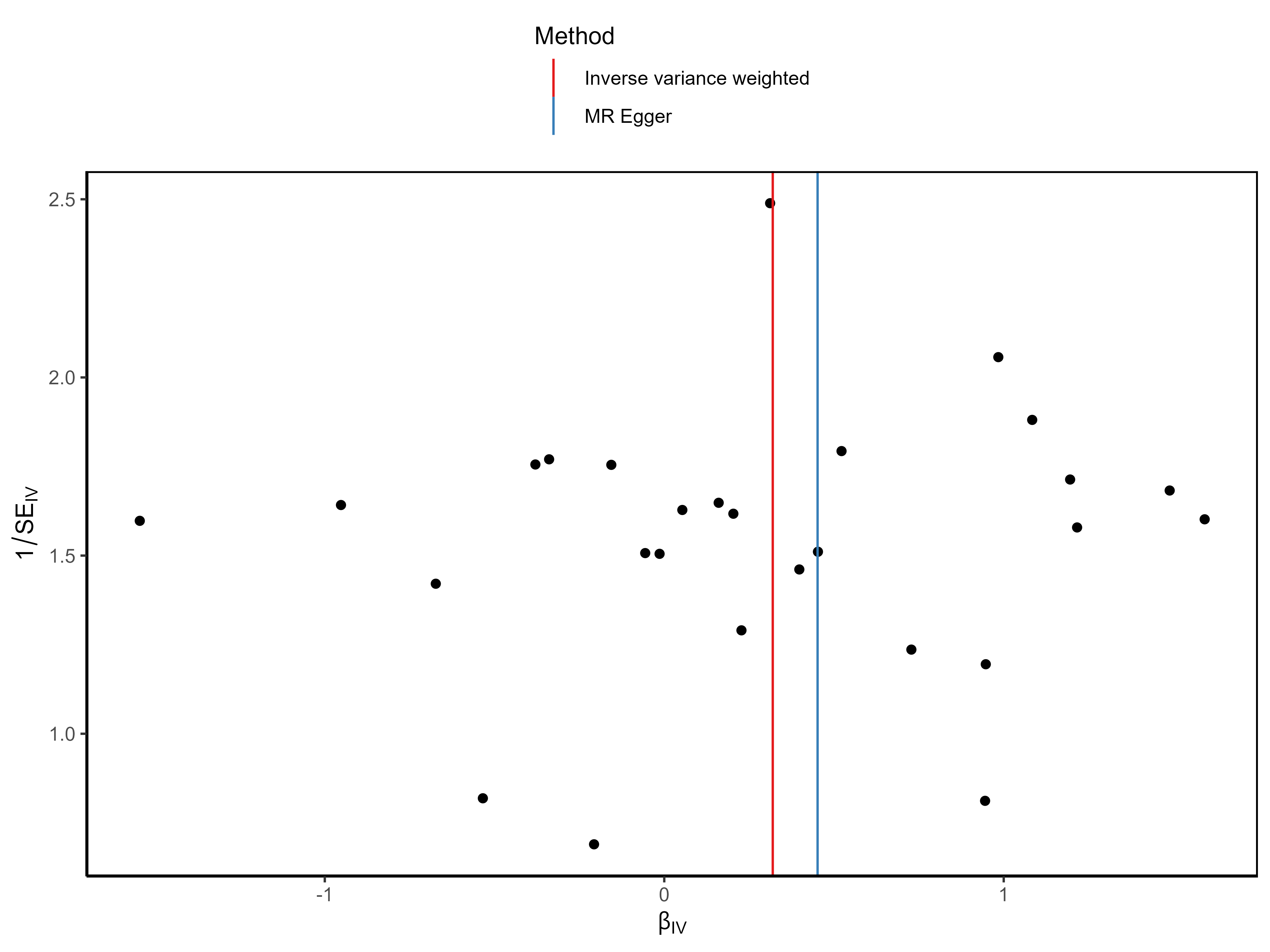


Figure S2.16 Funnel plot of SNPs associated with Cereal type: biscuit cereal on SC.

**FigureS3.** Leave-one-out analysis for the association between 16 significant dietary habits and SC in the forward analysis of bidirectional MR.


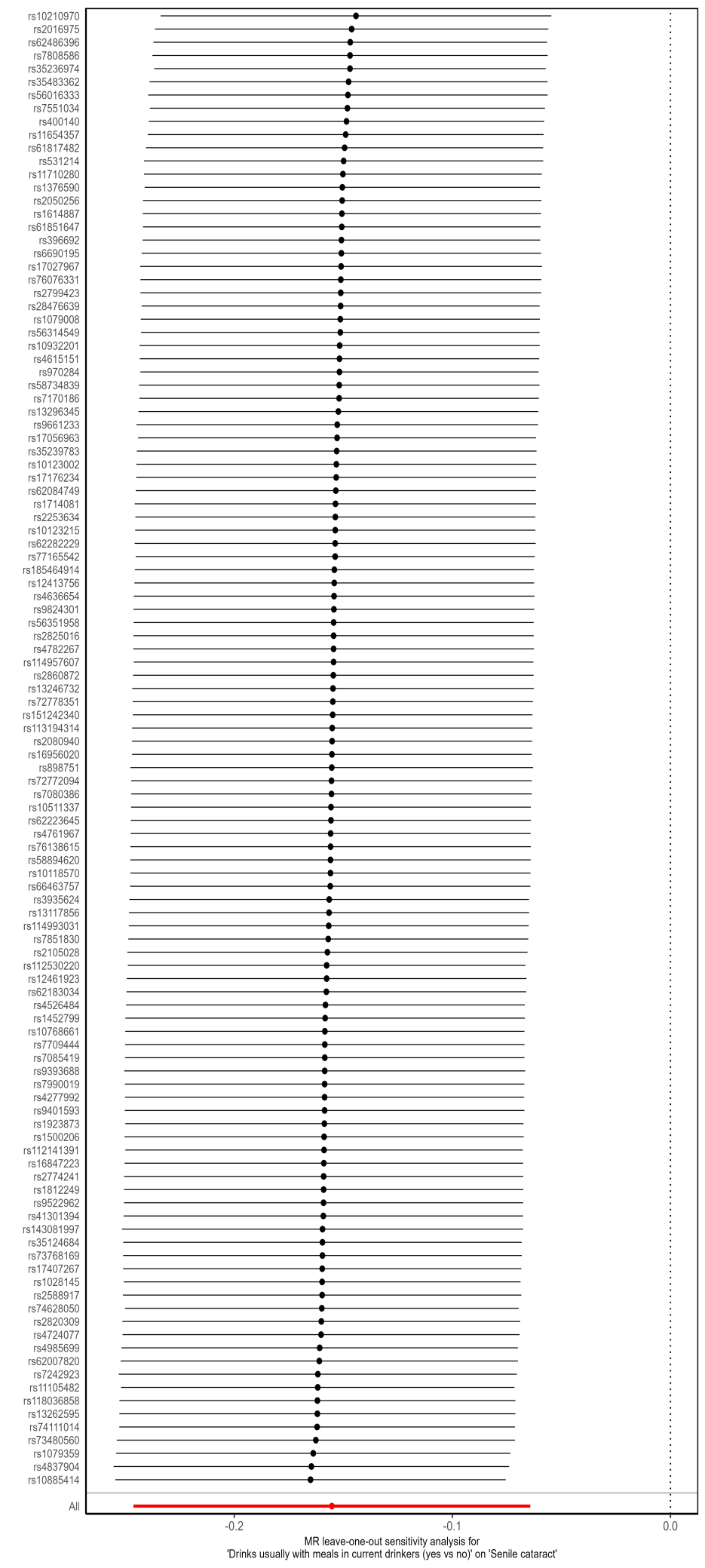


Figure S3.1 Leave-one-out analysis of SNPs associated with Drinks usually with meals in current drinkers (yes vs no) on SC.


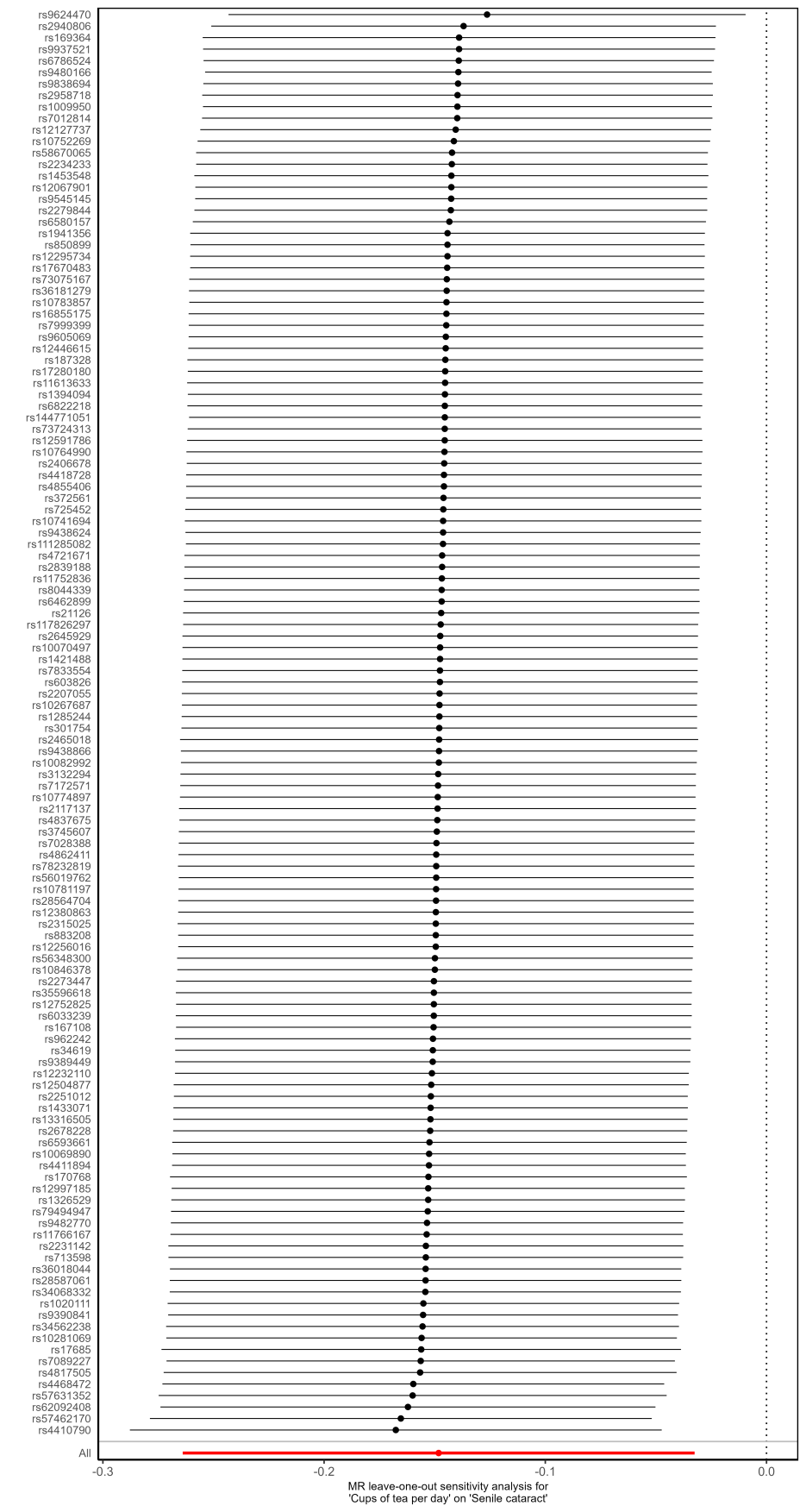


Figure S3.2 Leave-one-out analysis of SNPs associated with Cups of tea per day on SC.


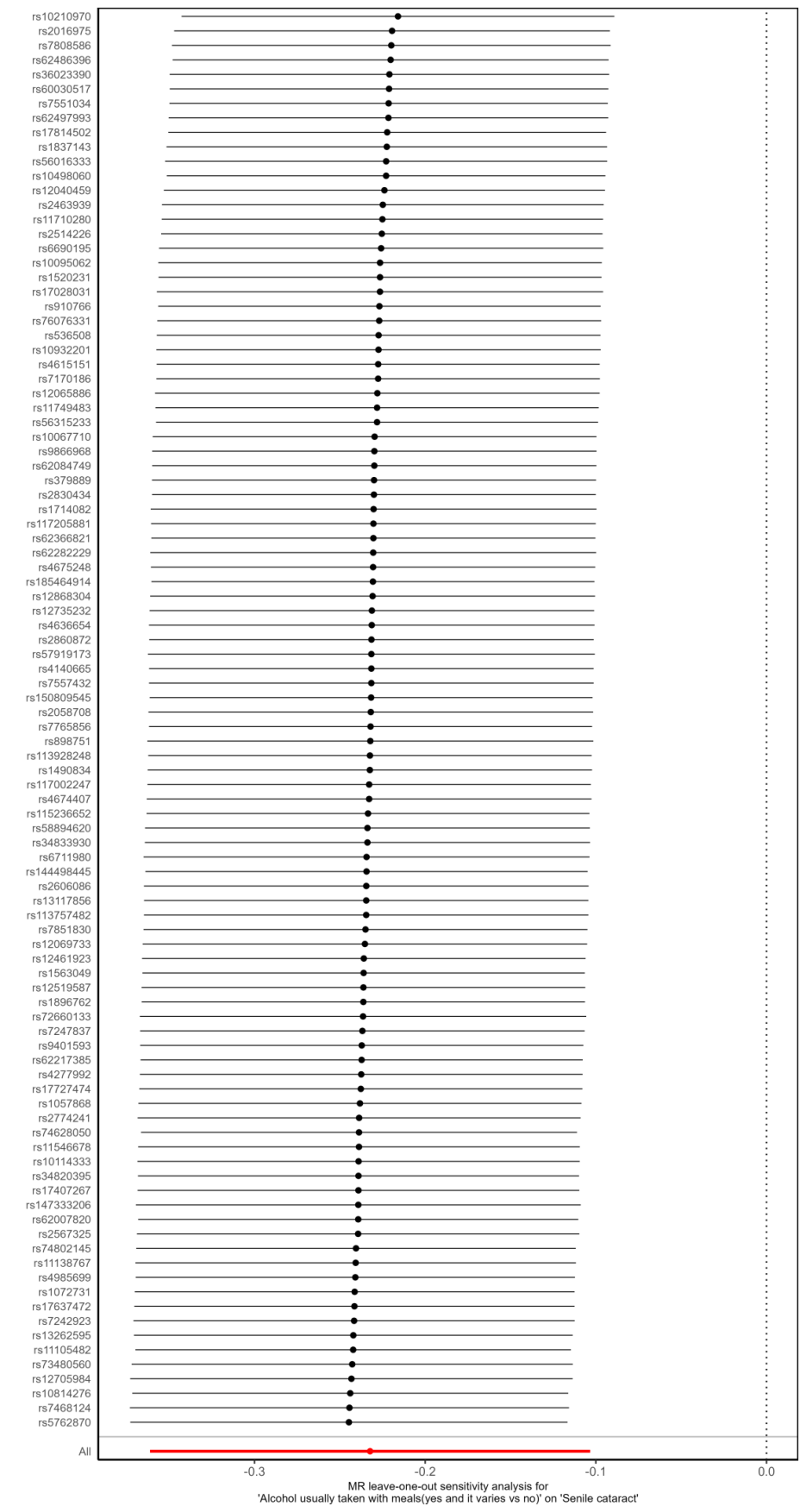


Figure S3.3 Leave-one-out analysis of SNPs associated with Alcohol usually taken with meals (yes and it varies vs no) on SC.


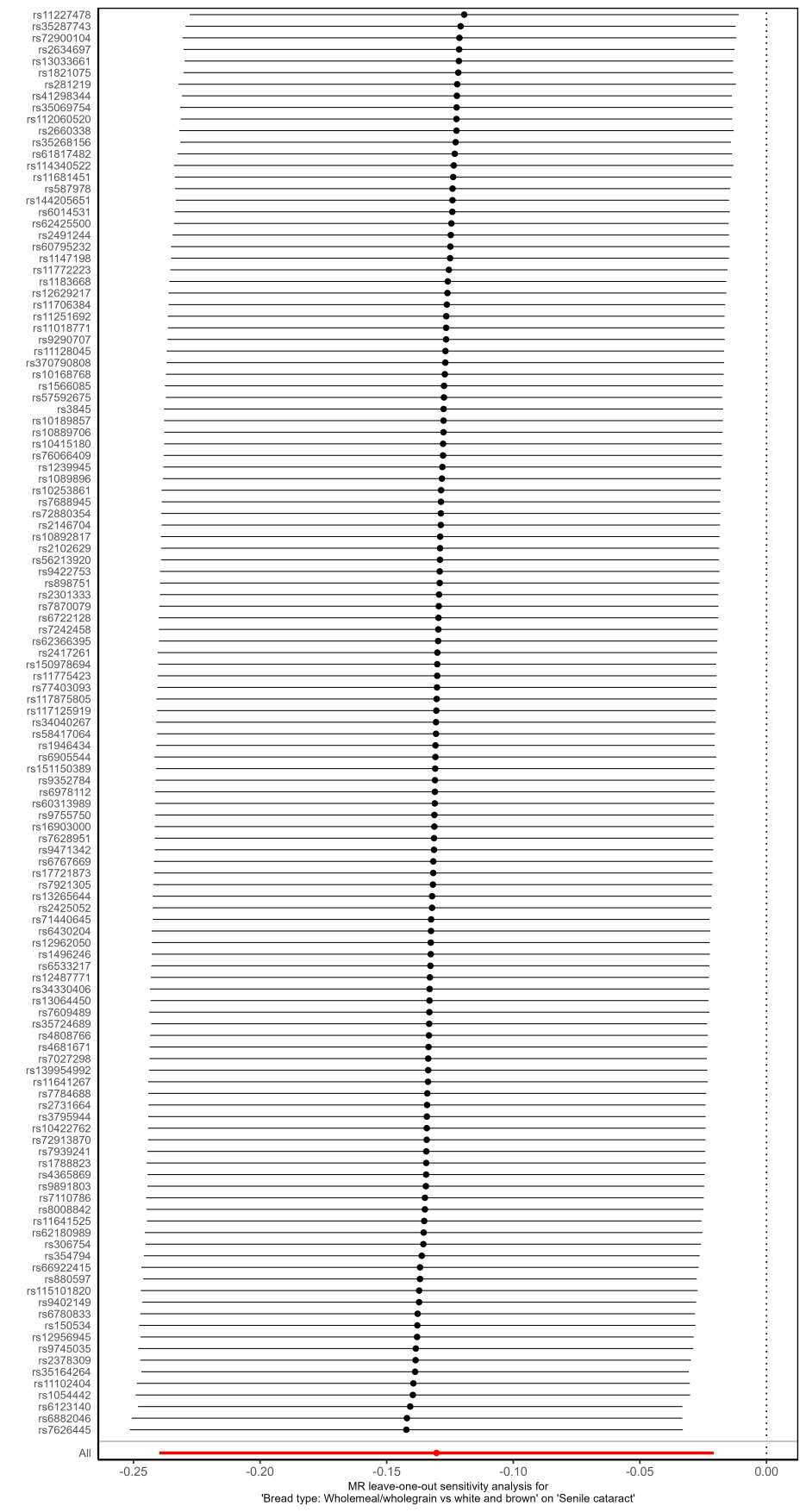


Figure S3.4 Leave-one-out analysis of SNPs associated with Bread type: wholemeal/wholegrain vs white and brown on SC.


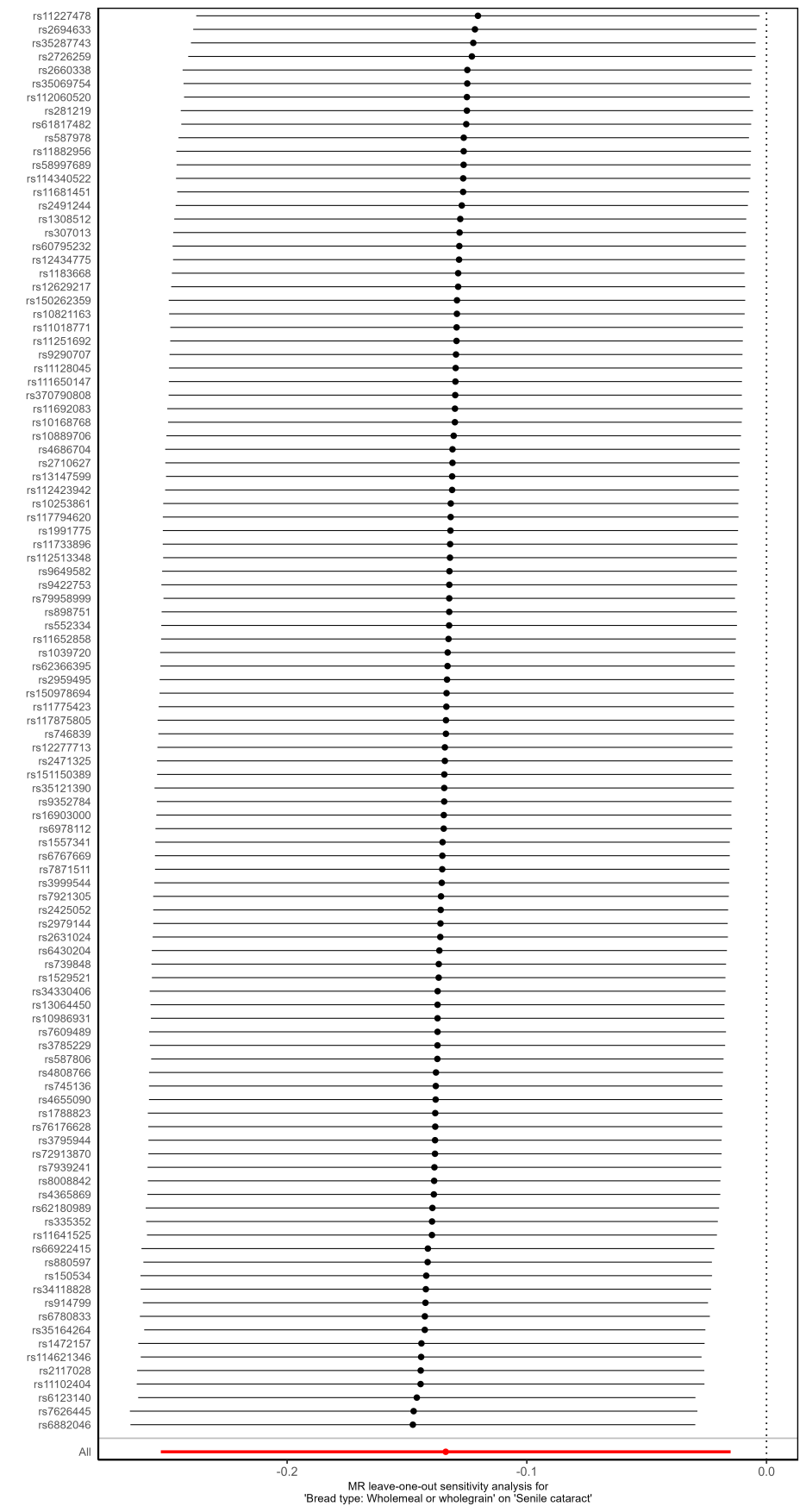


Figure S3.5 Leave-one-out analysis of SNPs associated with Bread type: wholemeal or wholegrain on SC.


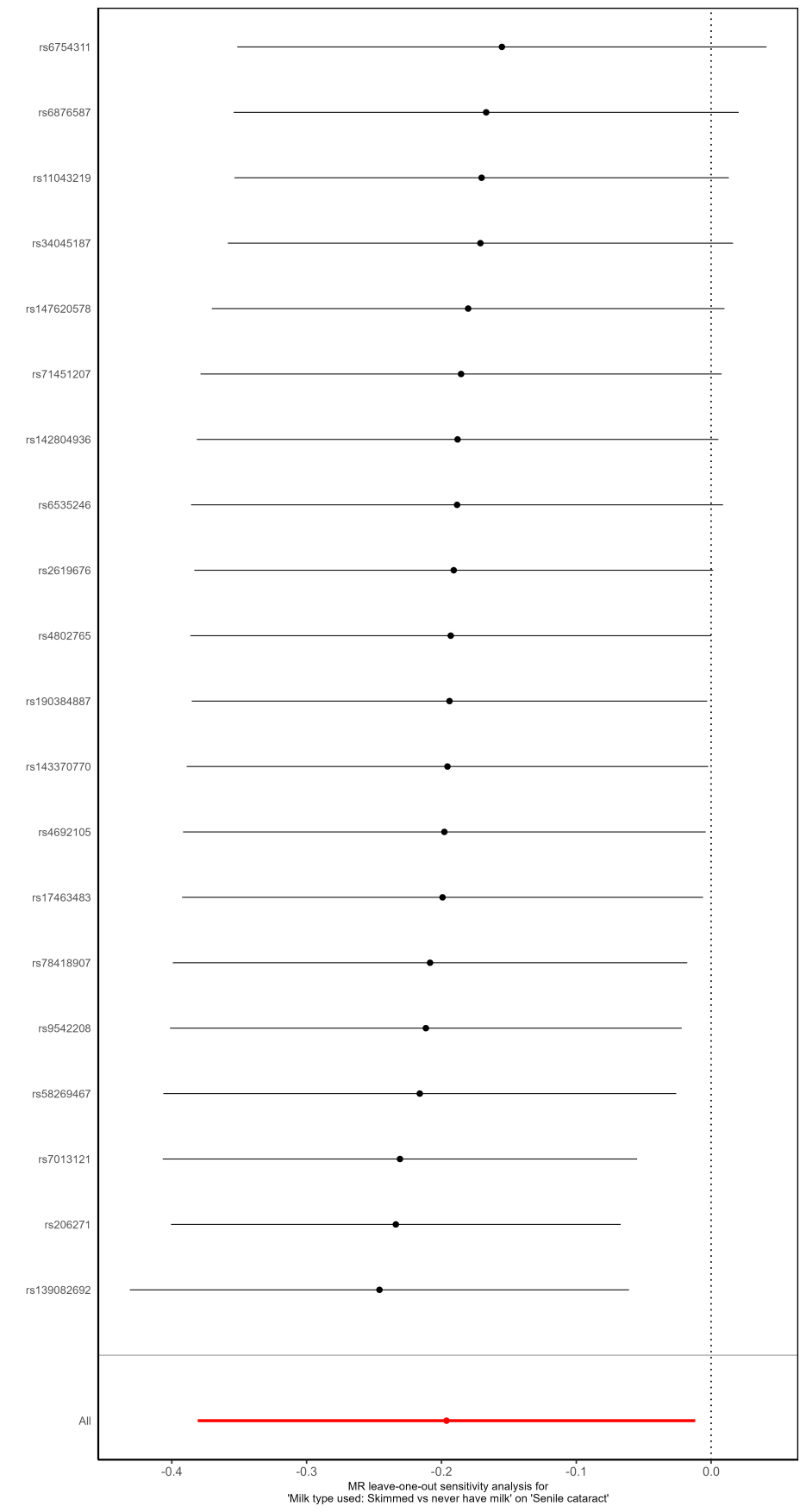


Figure S3.6 Leave-one-out analysis of SNPs associated with Milk type used: skimmed vs never have milk on SC.


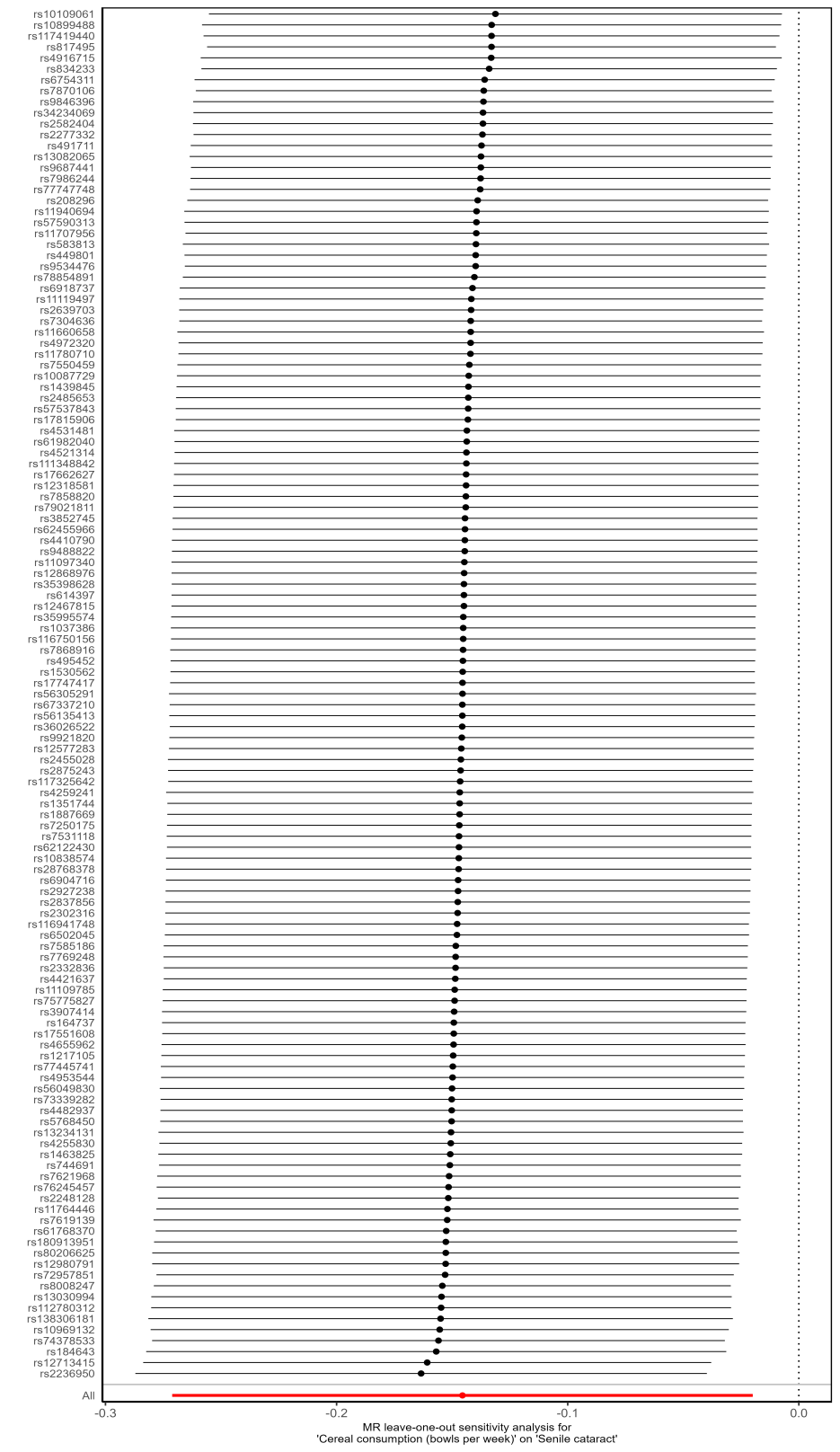


Figure S3.7 Leave-one-out analysis of SNPs associated with Cereal consumption (bowls per week) on SC.


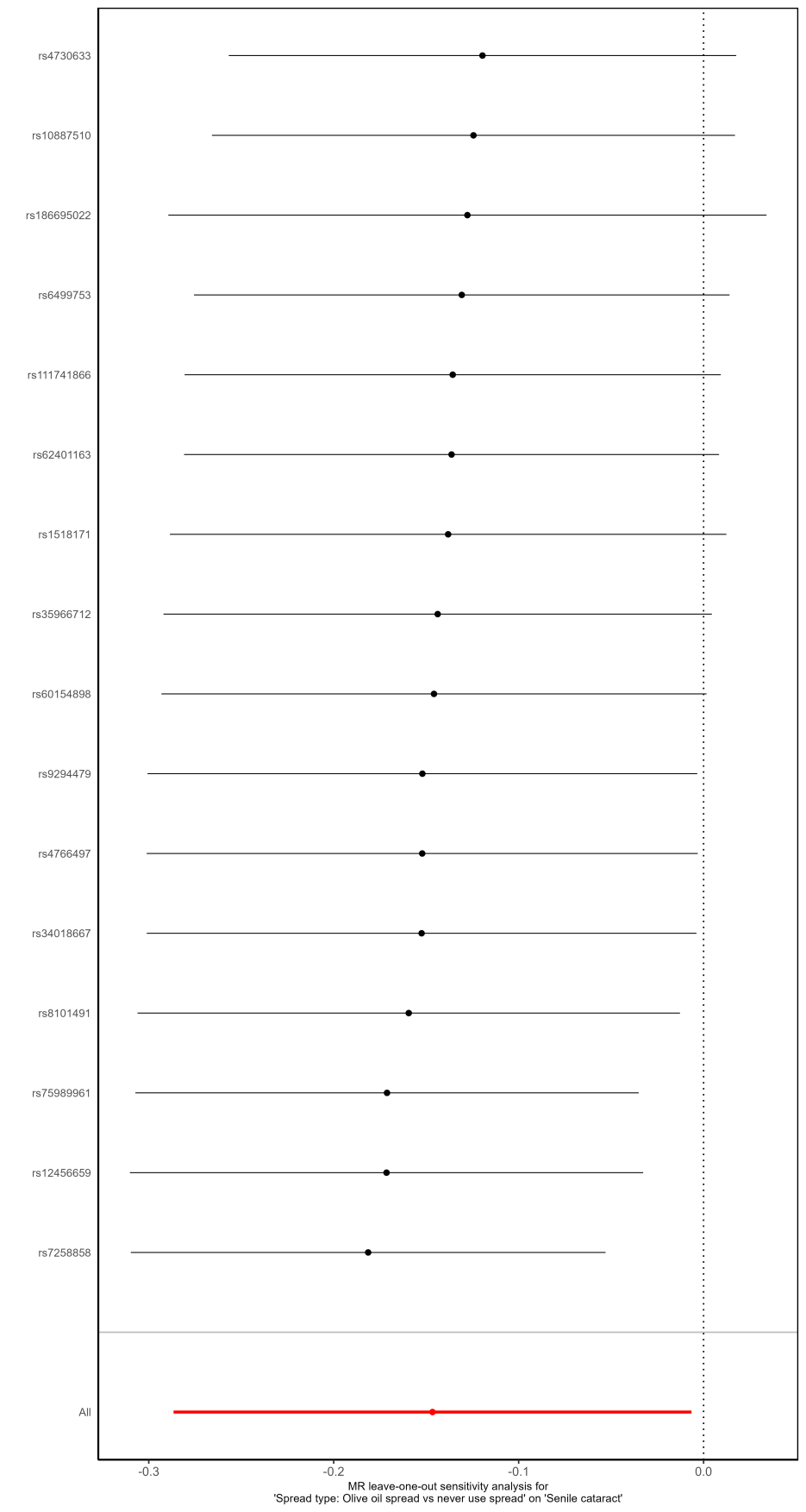


Figure S3.8 Leave-one-out analysis of SNPs associated with Spread type: olive oil spread vs never use spread on SC.


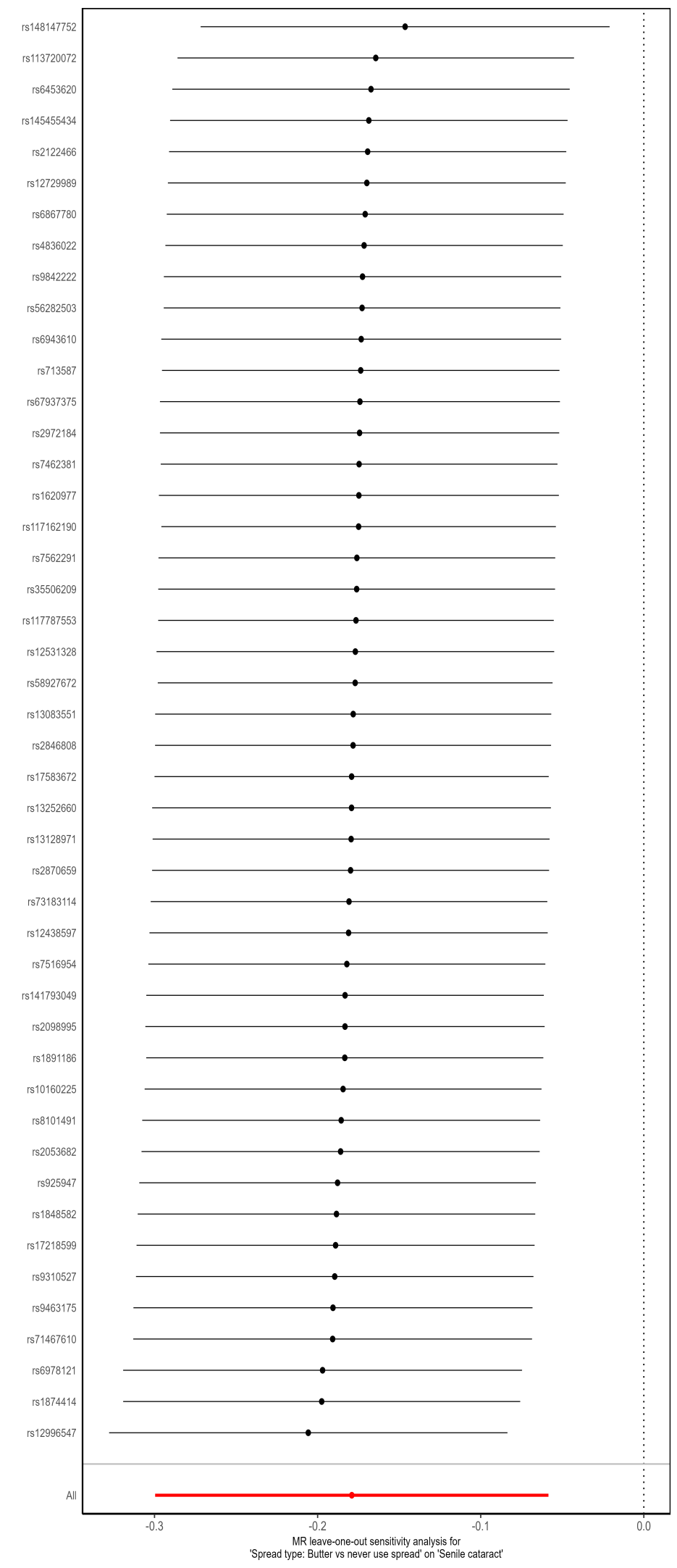


Figure S3.9 Leave-one-out analysis of SNPs associated with Spread type: butter vs never use spread on SC.


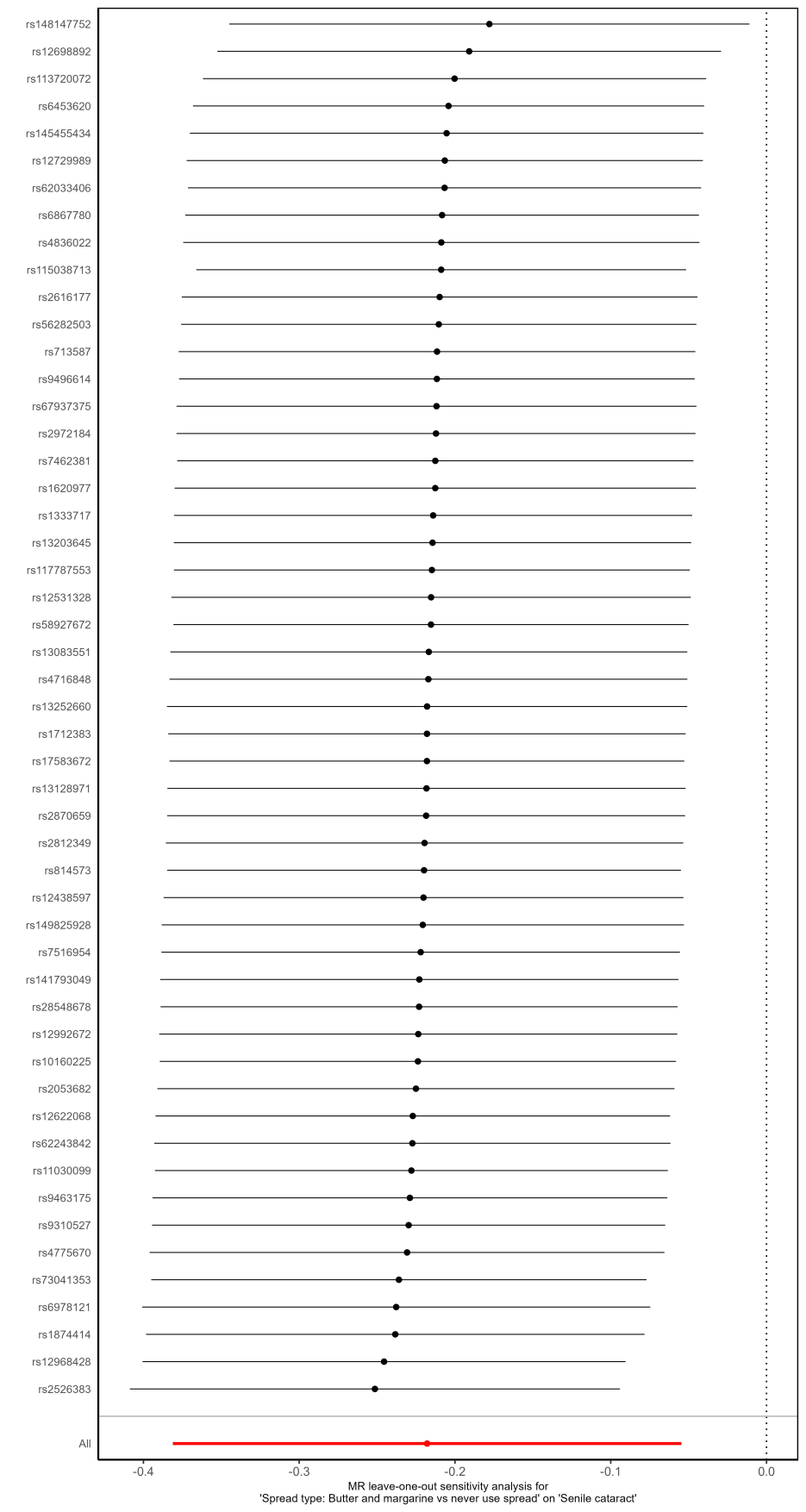


Figure S3.10 Leave-one-out analysis of SNPs associated with Spread type: butter and margarine vs never use spread on SC.


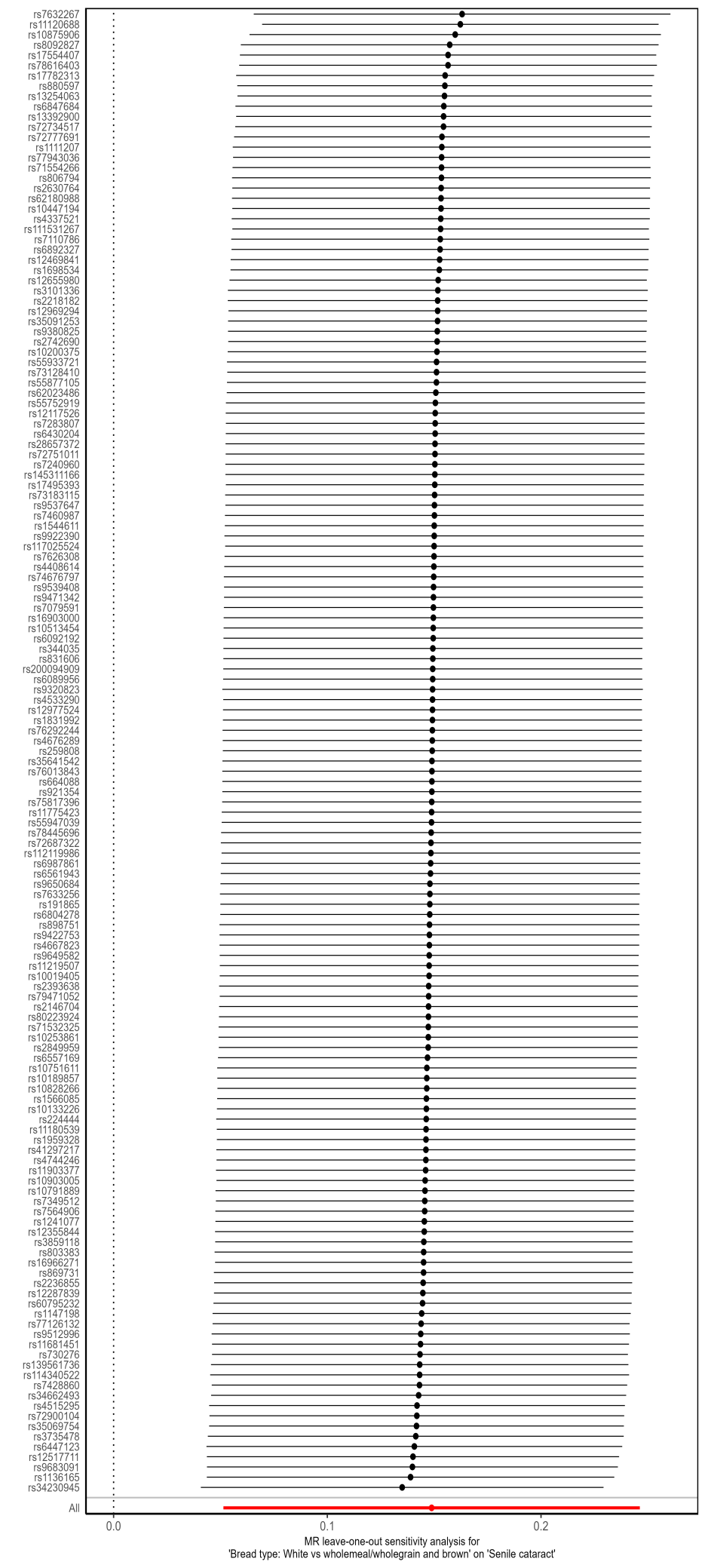


Figure S3.11 Leave-one-out analysis of SNPs associated with Bread type: white vs wholemeal/wholegrain and brown on SC.


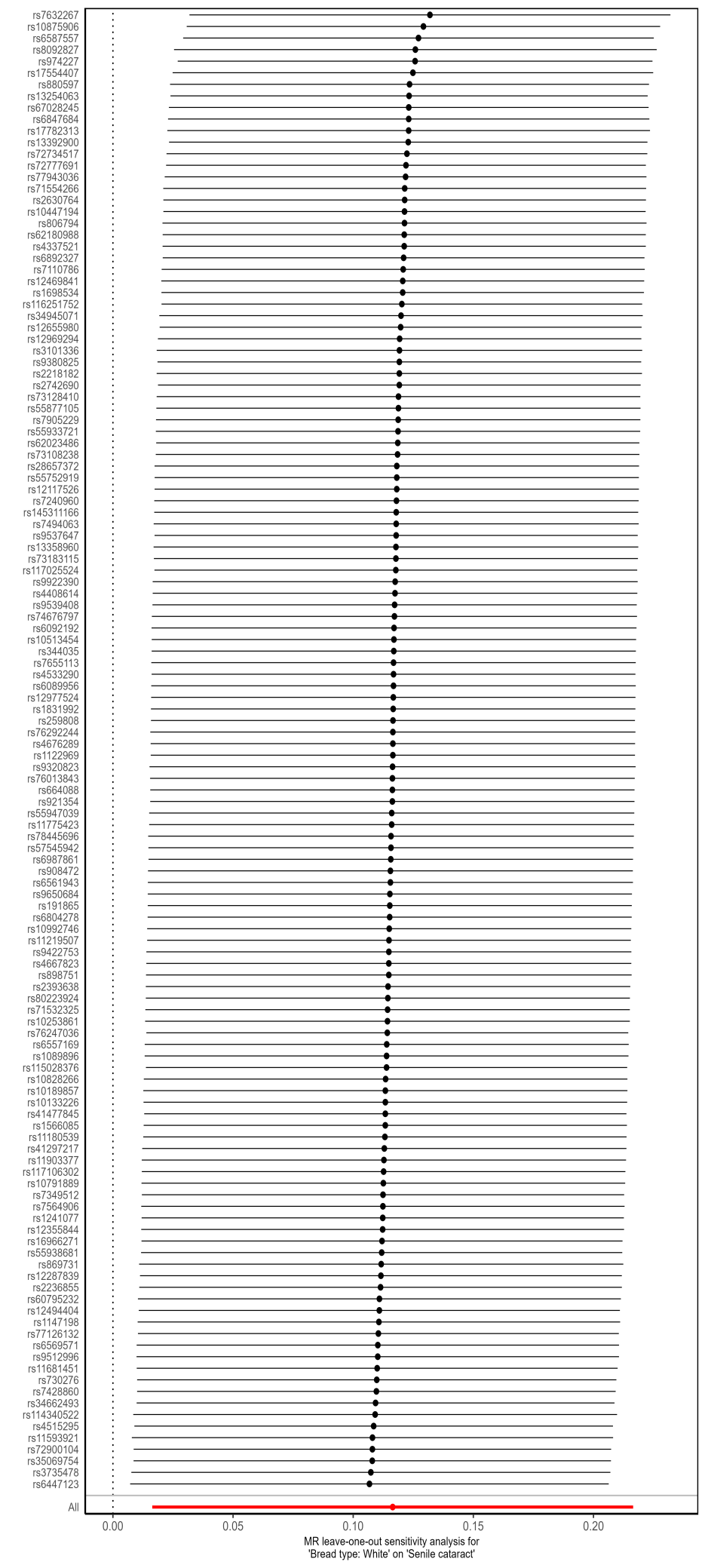


Figure S3.12 Leave-one-out analysis of SNPs associated with Bread type: white on SC.

Figure S3.13 Leave-one-out analysis of SNPs associated with Bread type: white vs wholemeal/wholegrain and brown on SC.

Figure S3.14 Leave-one-out analysis of SNPs associated with Temperature of hot drinks on SC.


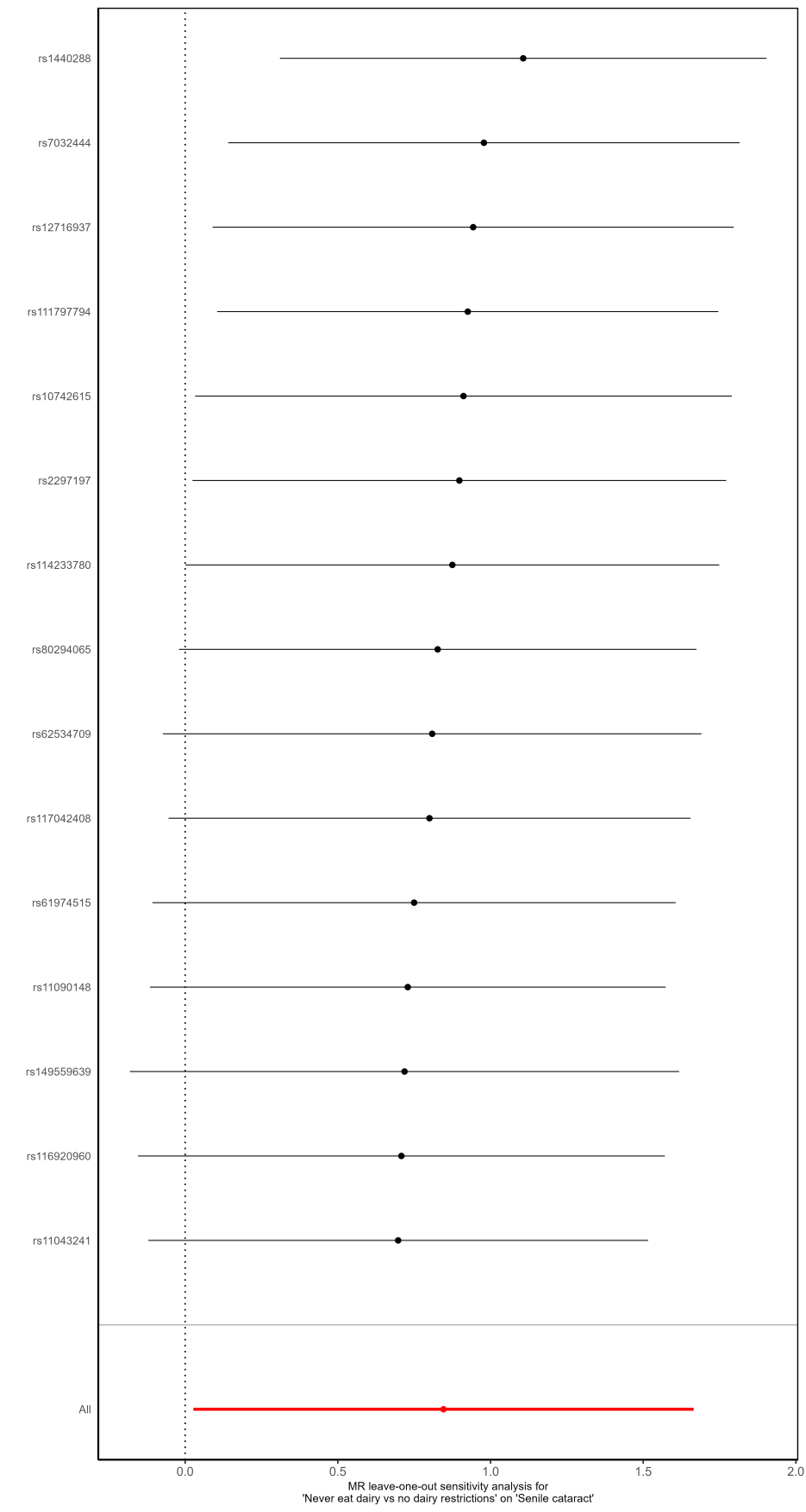


Figure S3.14 Leave-one-out analysis of SNPs associated with Never eat dairy vs no dairy restrictions on SC.


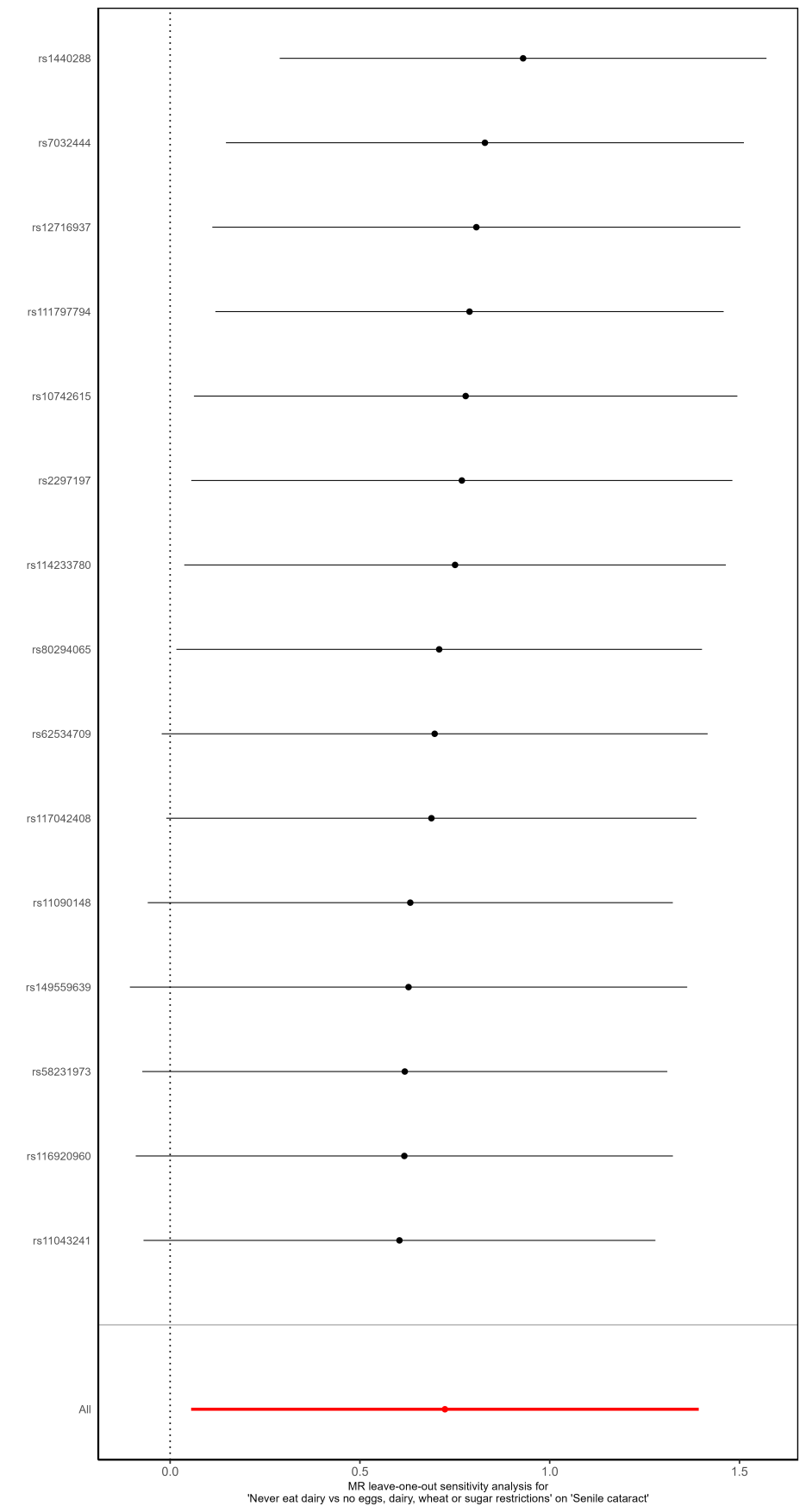


Figure S3.15 Leave-one-out analysis of SNPs associated with Never eat dairy vs no eggs, dairy, wheat, or sugar restrictions on SC.


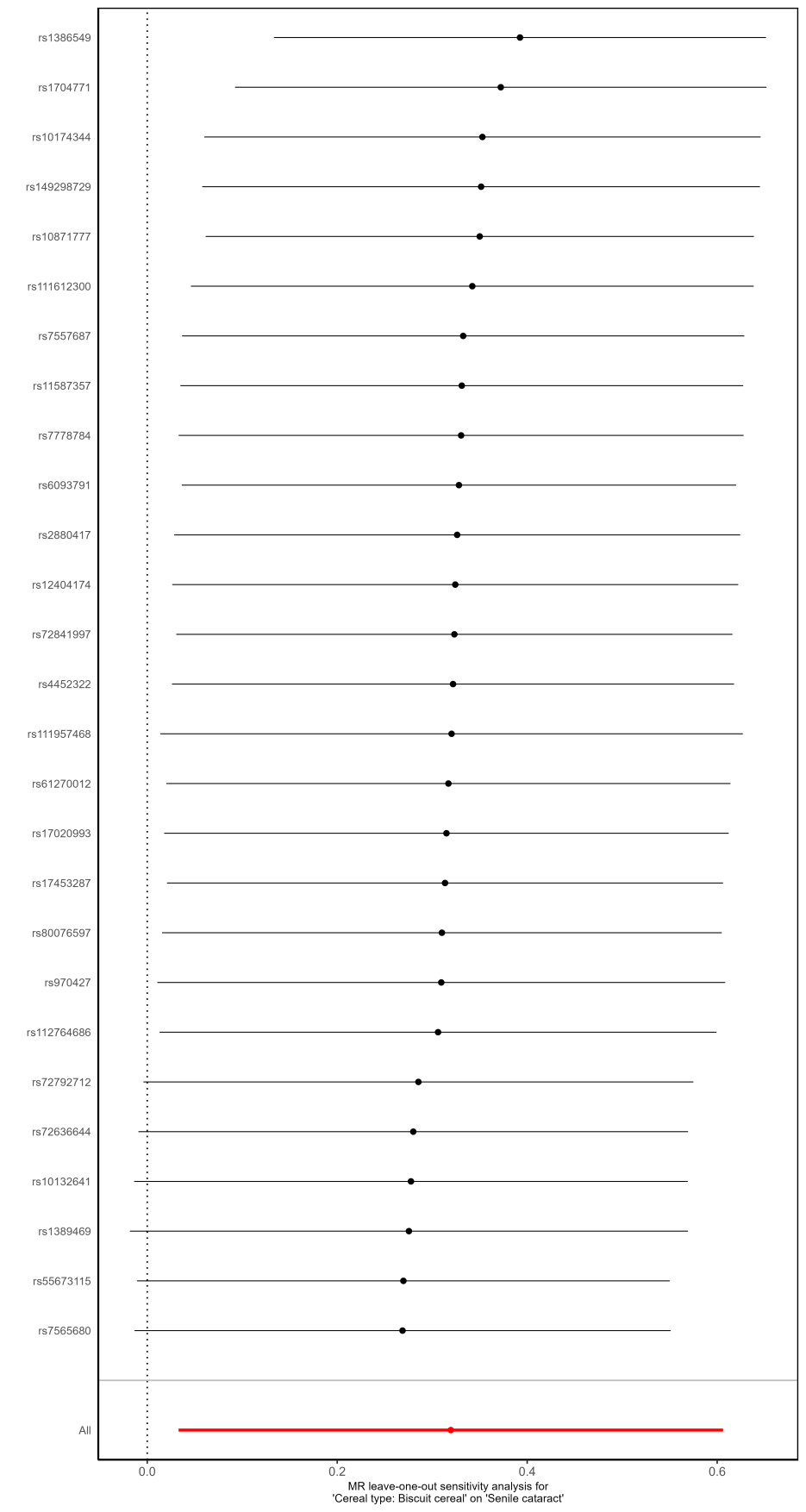


Figure S3.16 Leave-one-out analysis of SNPs associated with Cereal type: biscuit cereal on SC.

**Figure S4.** Scatter plots for the association between 16 significant dietary habits and SC in the reverse analysis of bidirectional MR.


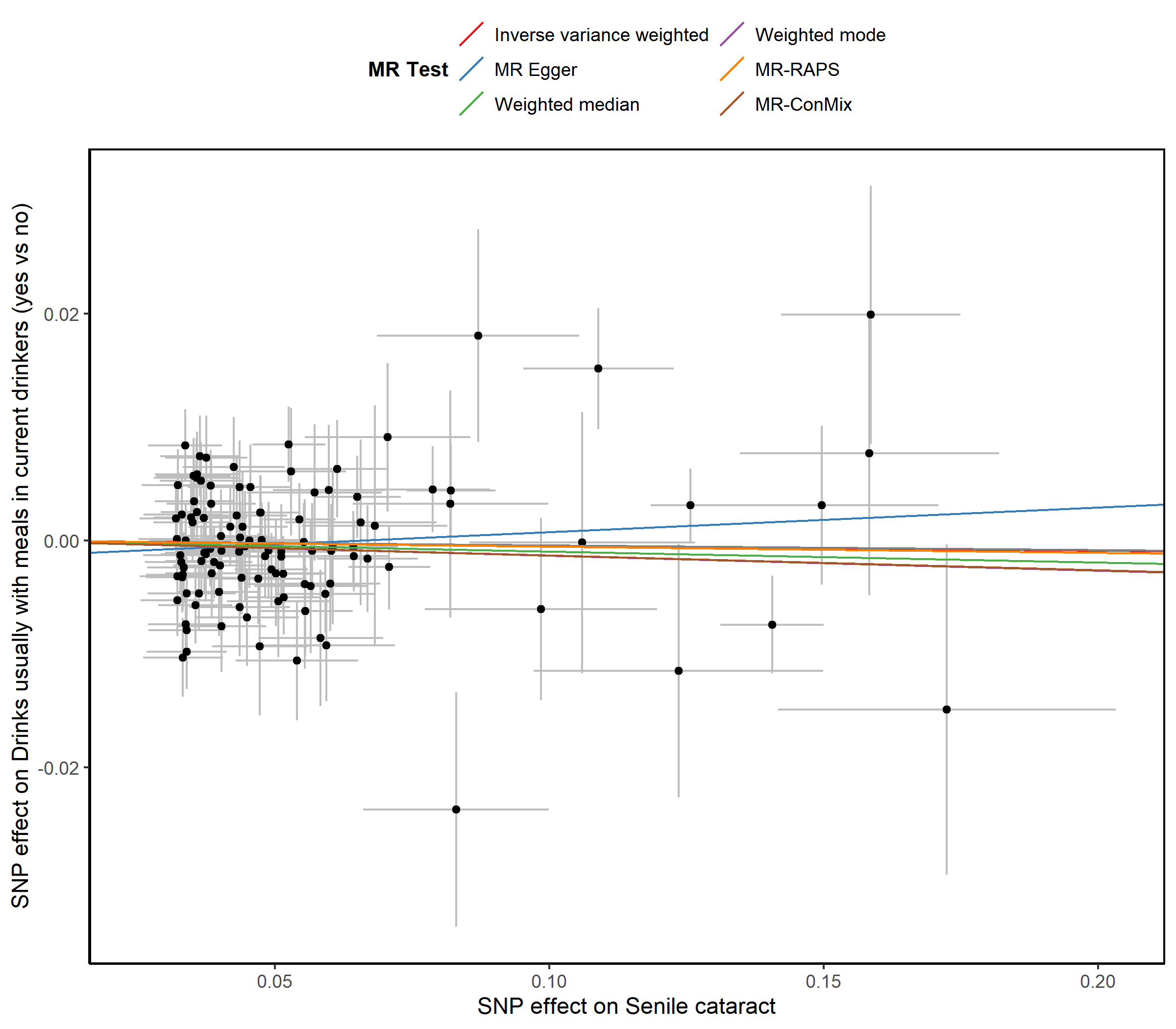


Figure S4.1 Scatter plot of SNPs associated with SC on Drinks usually with meals in current drinkers (yes vs no).


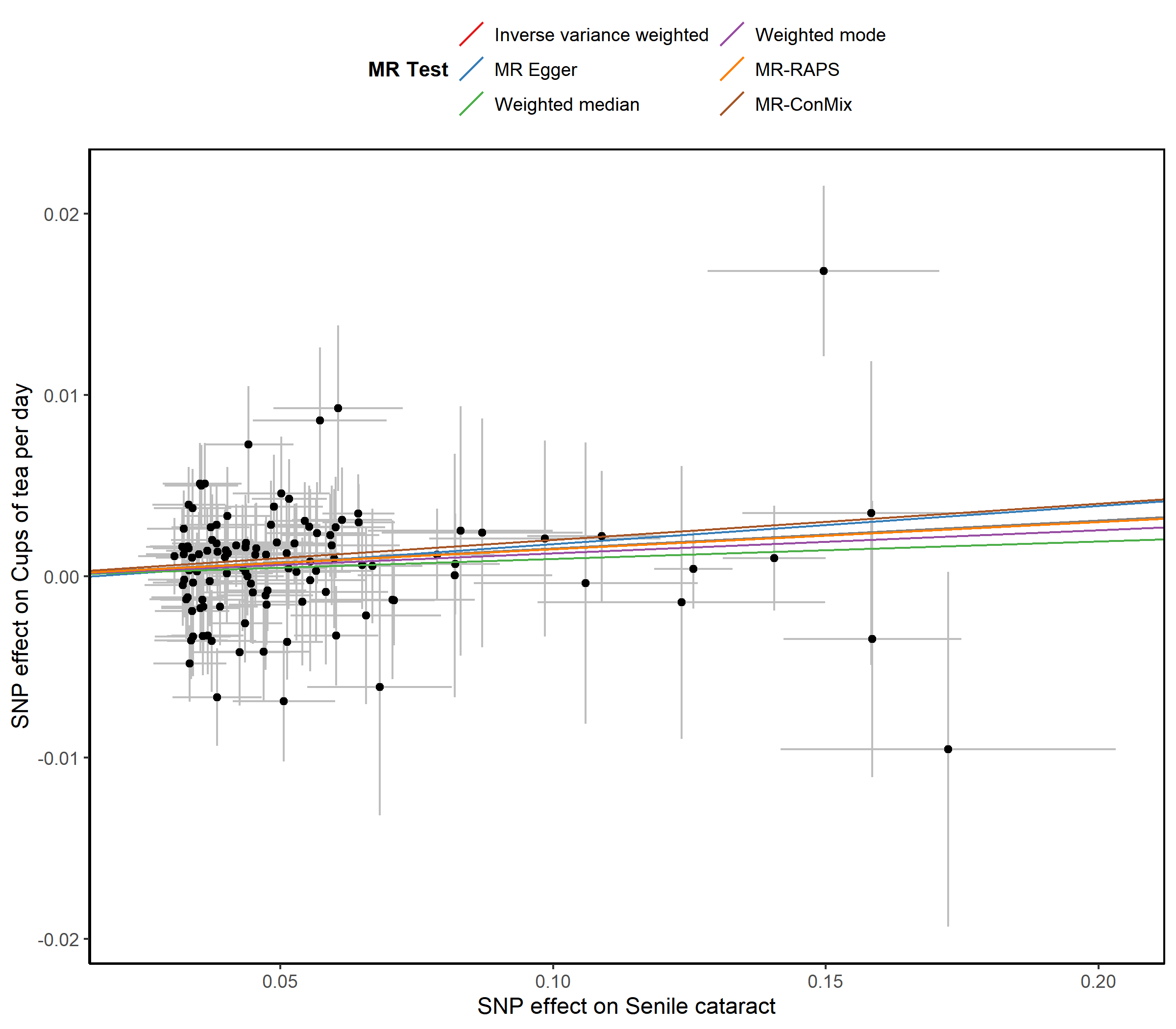


Figure S4.2 Scatter plot of SNPs associated with SC on Cups of tea per day.


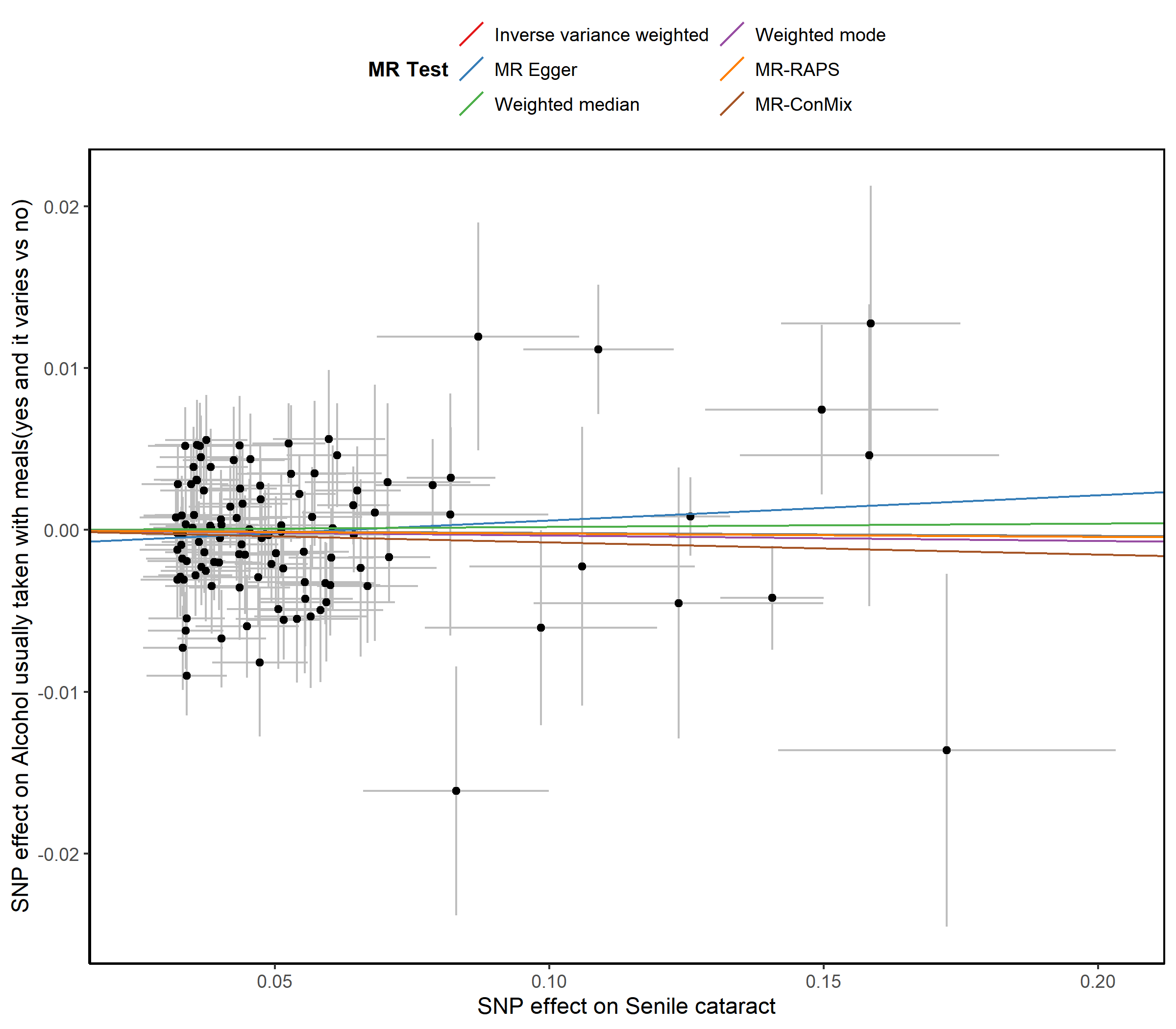


Figure S4.3 Scatter plot of SNPs associated with SC on Alcohol usually taken with meals (yes and it varies vs no).


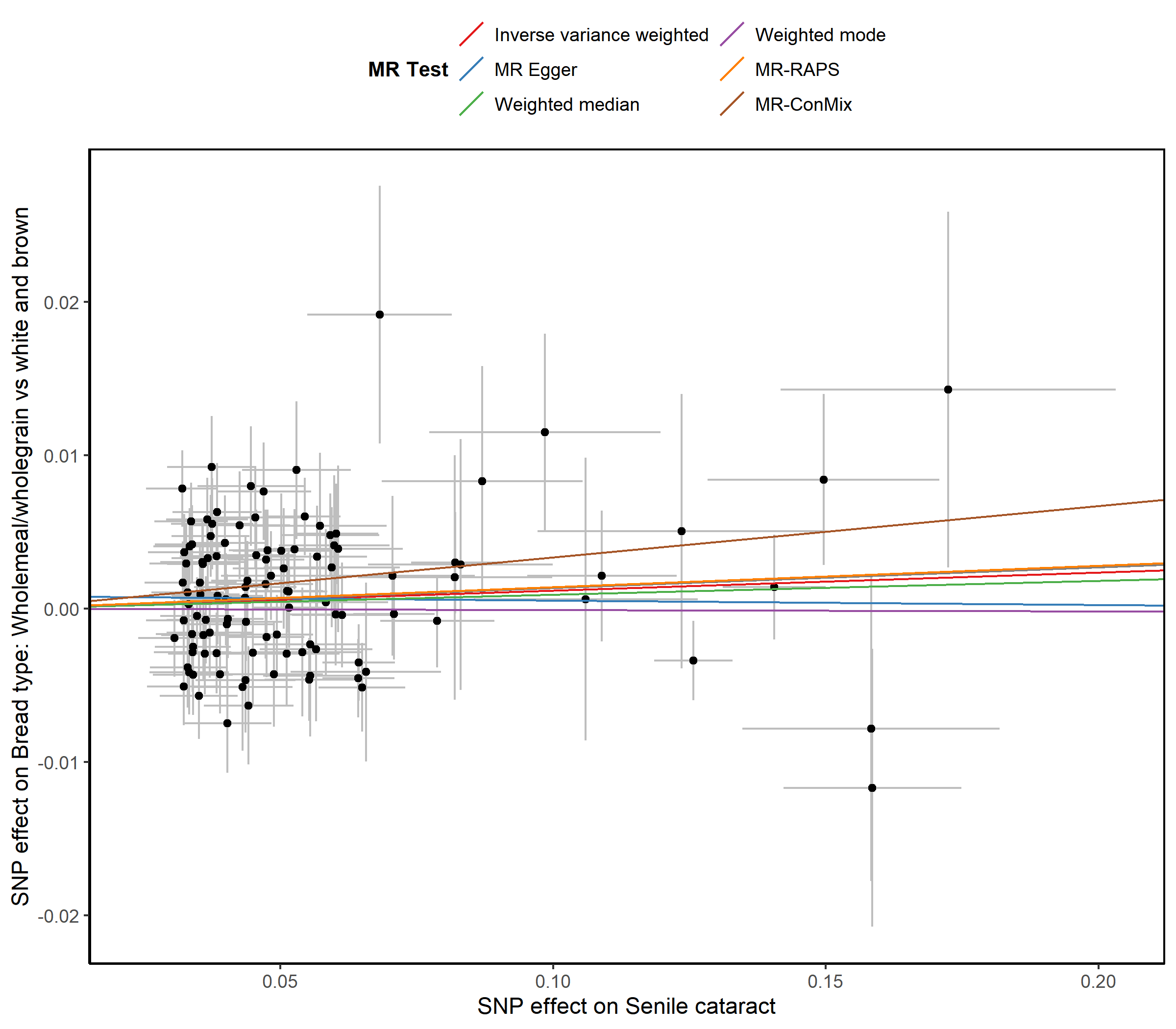


Figure S4.4 Scatter plot of SNPs associated with SC on Bread type: wholemeal/wholegrain vs white and brown.


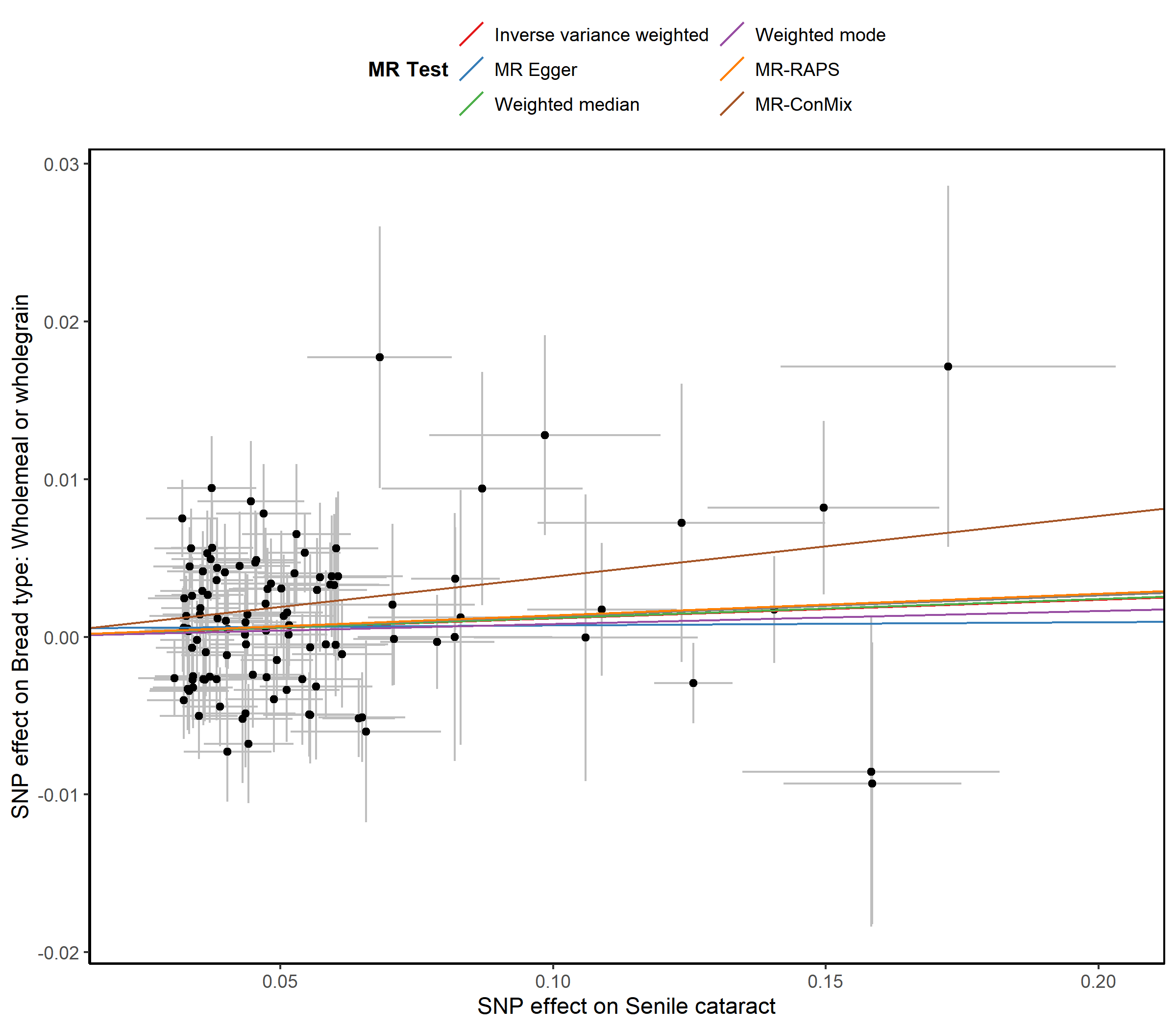


Figure S4.5 Scatter plot of SNPs associated with SC on Bread type: wholemeal or wholegrain.


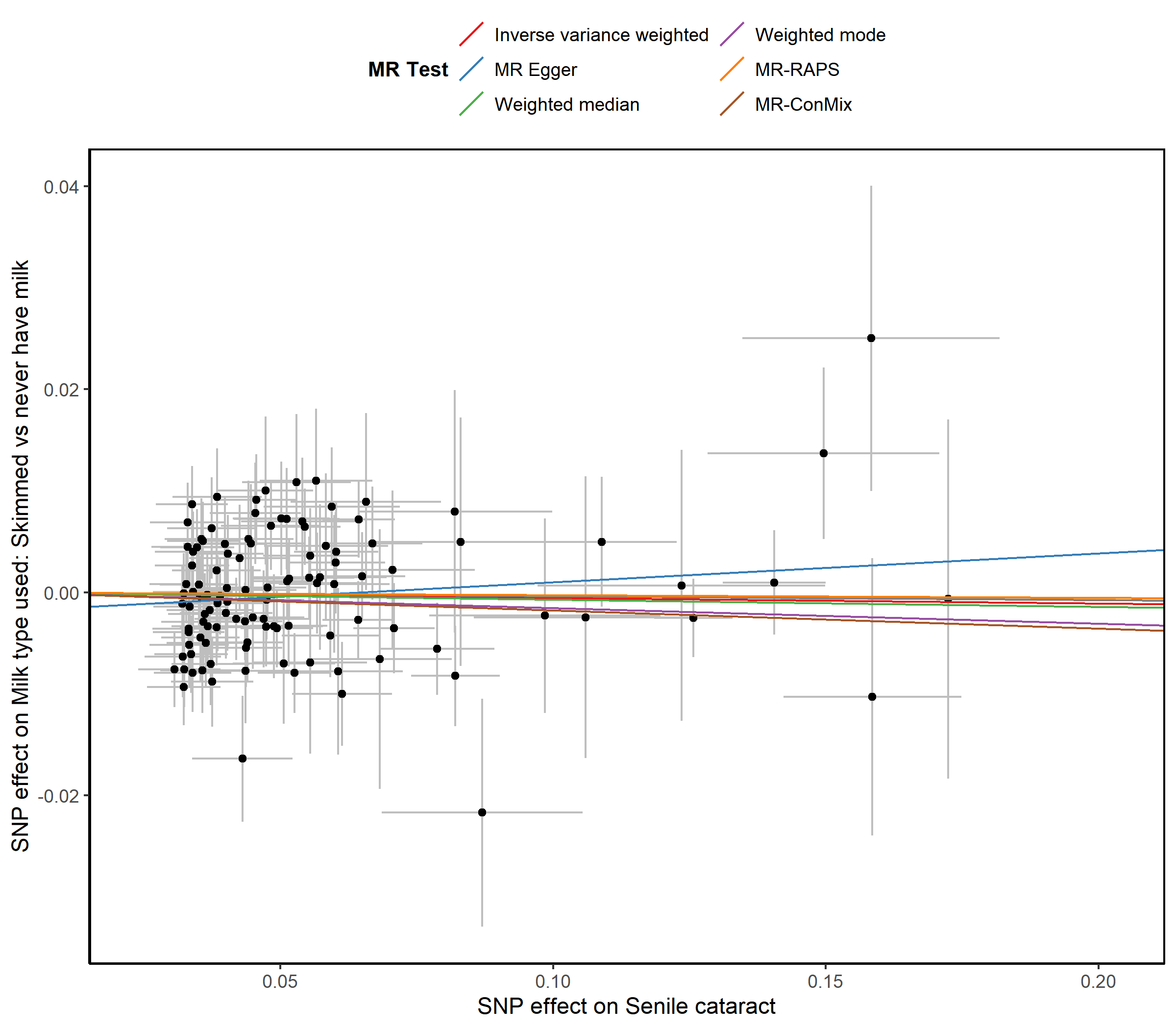


Figure S4.6 Scatter plot of SNPs associated with SC on Milk type used: skimmed vs never have milk.


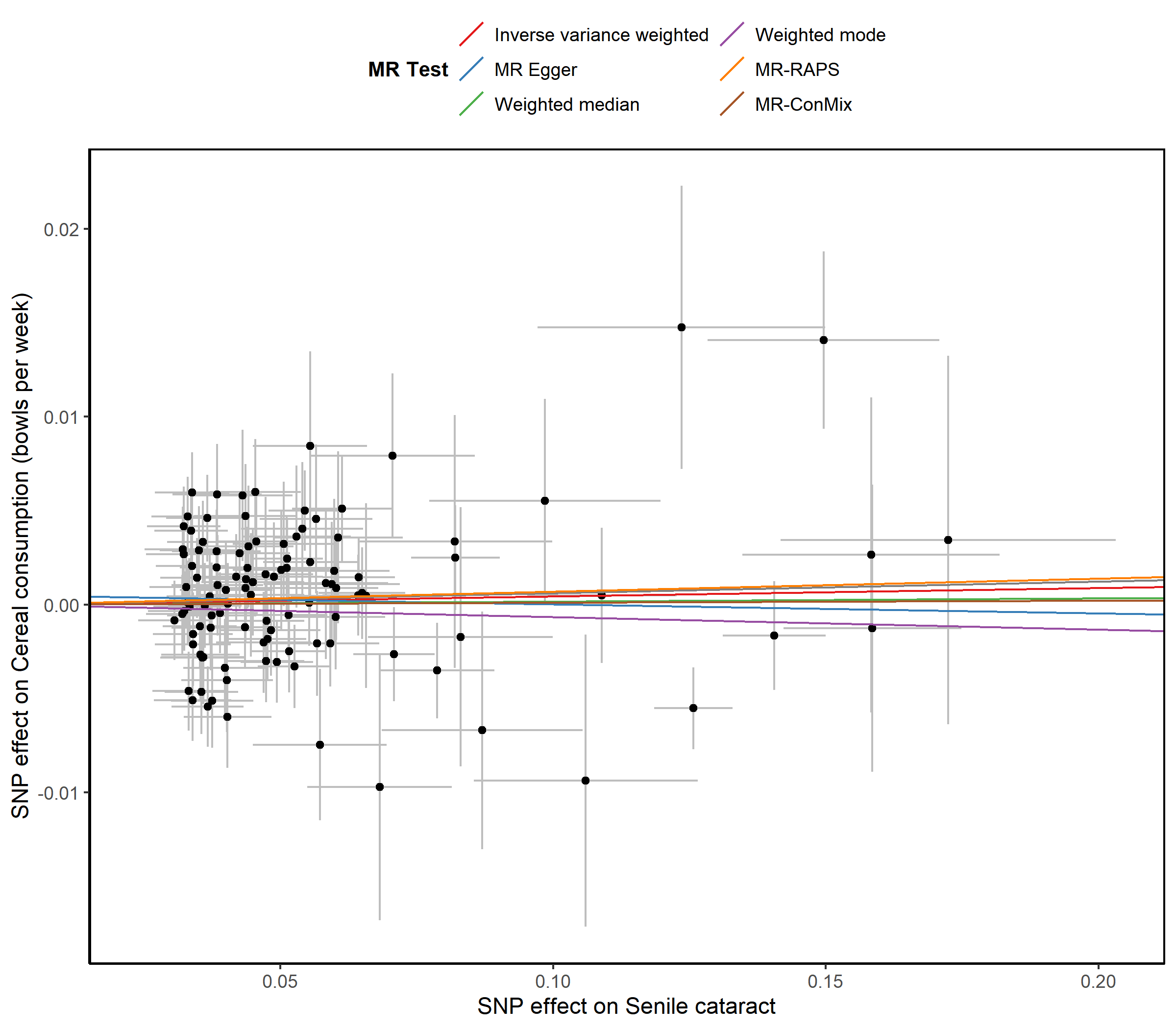


Figure S4.7 Scatter plot of SNPs associated with SC on Cereal consumption (bowls per week).


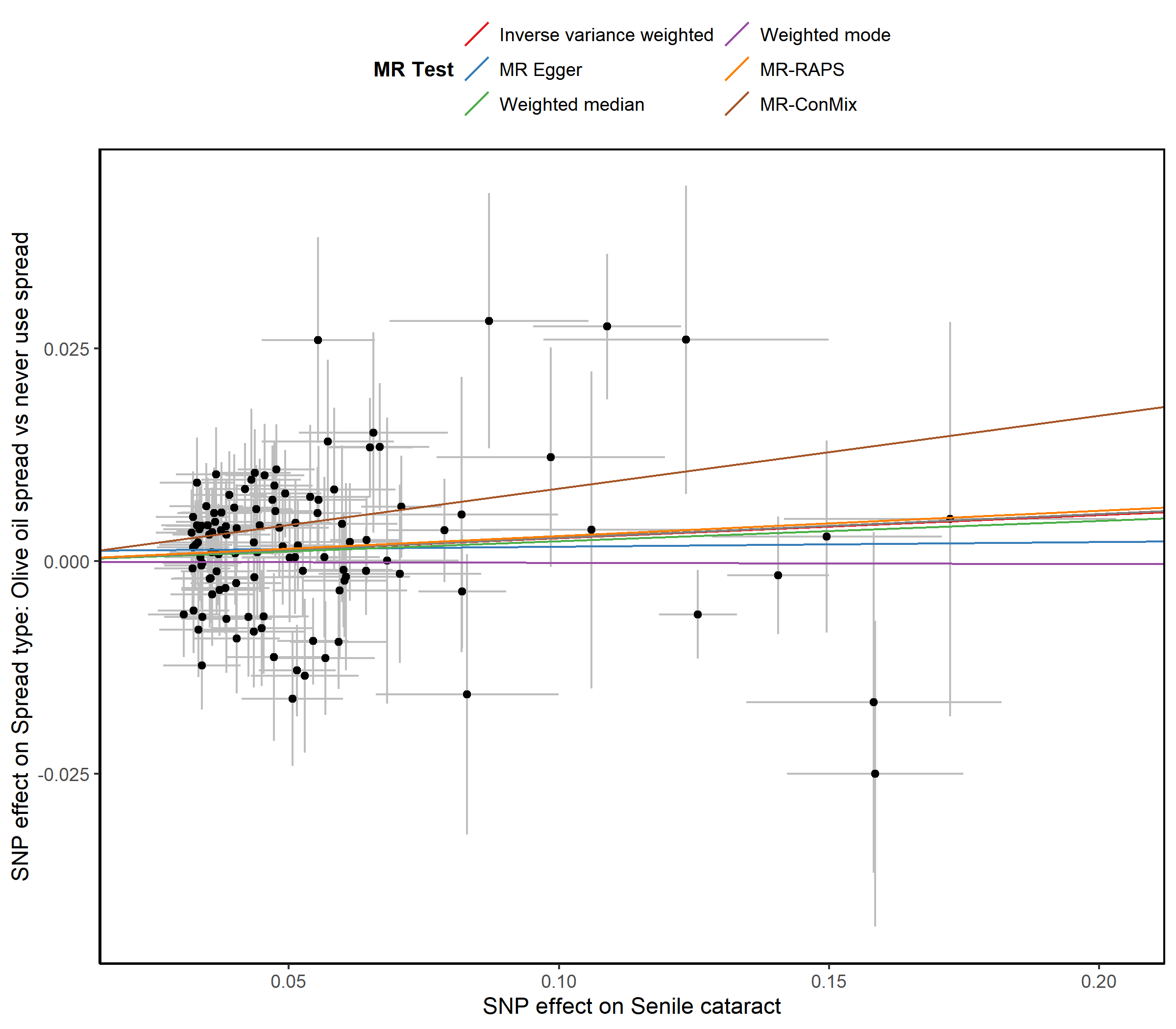


Figure S4.8 Scatter plot of SNPs associated with SC on Spread type: olive oil spread vs never use spread.


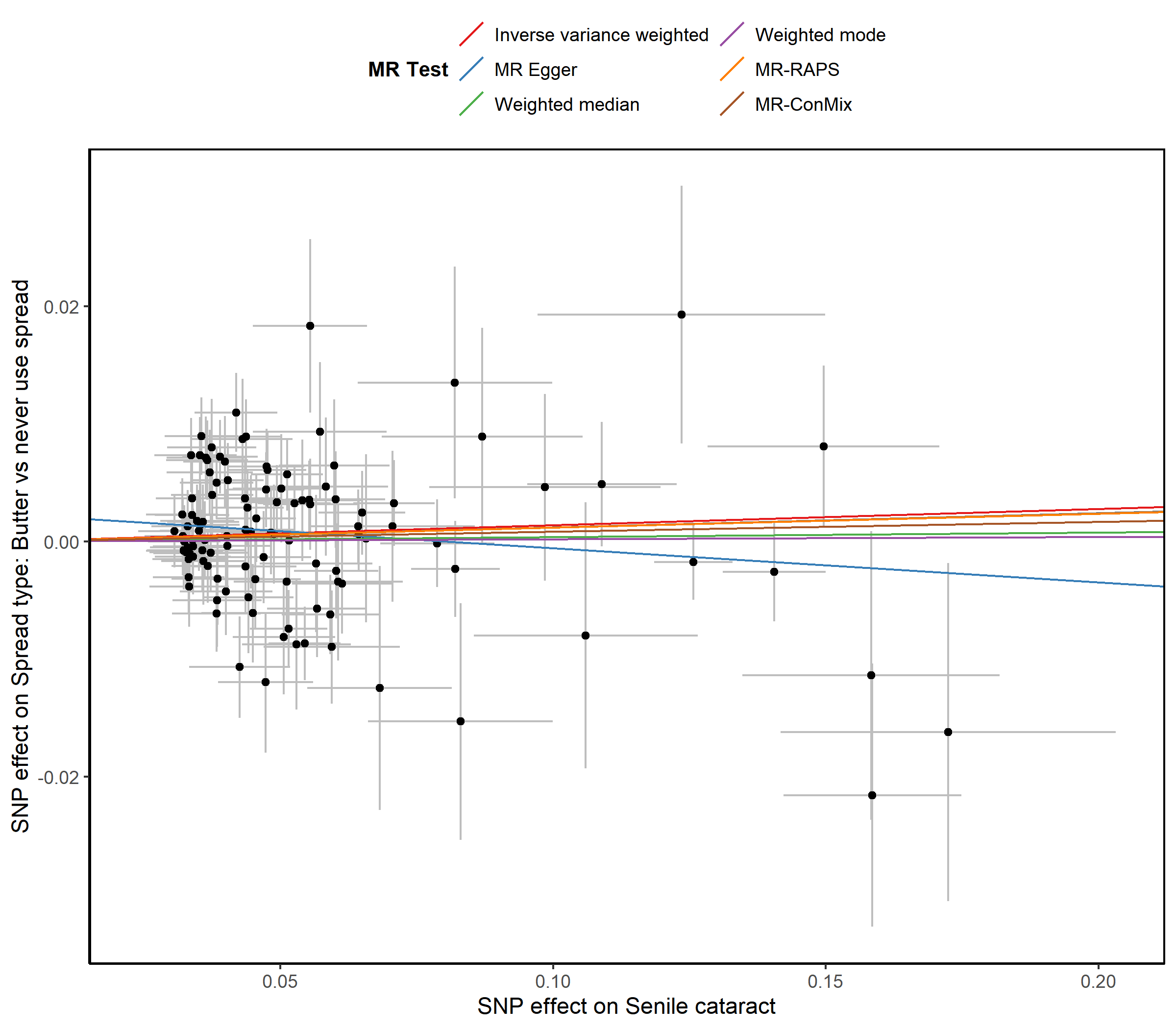


Figure S4.9 Scatter plot of SNPs associated with SC on Spread type: butter vs never use spread.


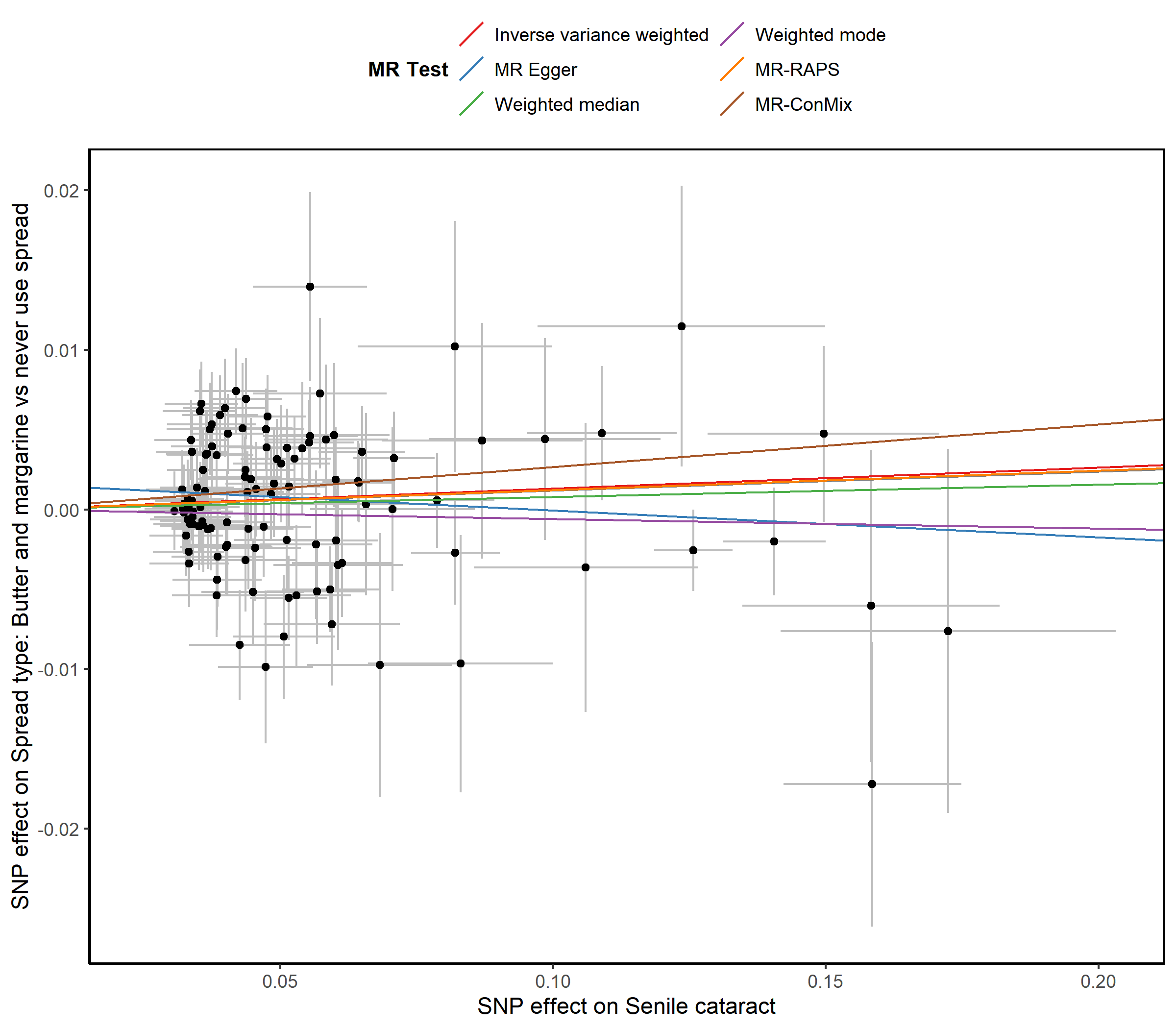


Figure S4.10 Scatter plot of SNPs associated with SC on Spread type: butter and margarine vs never use spread.


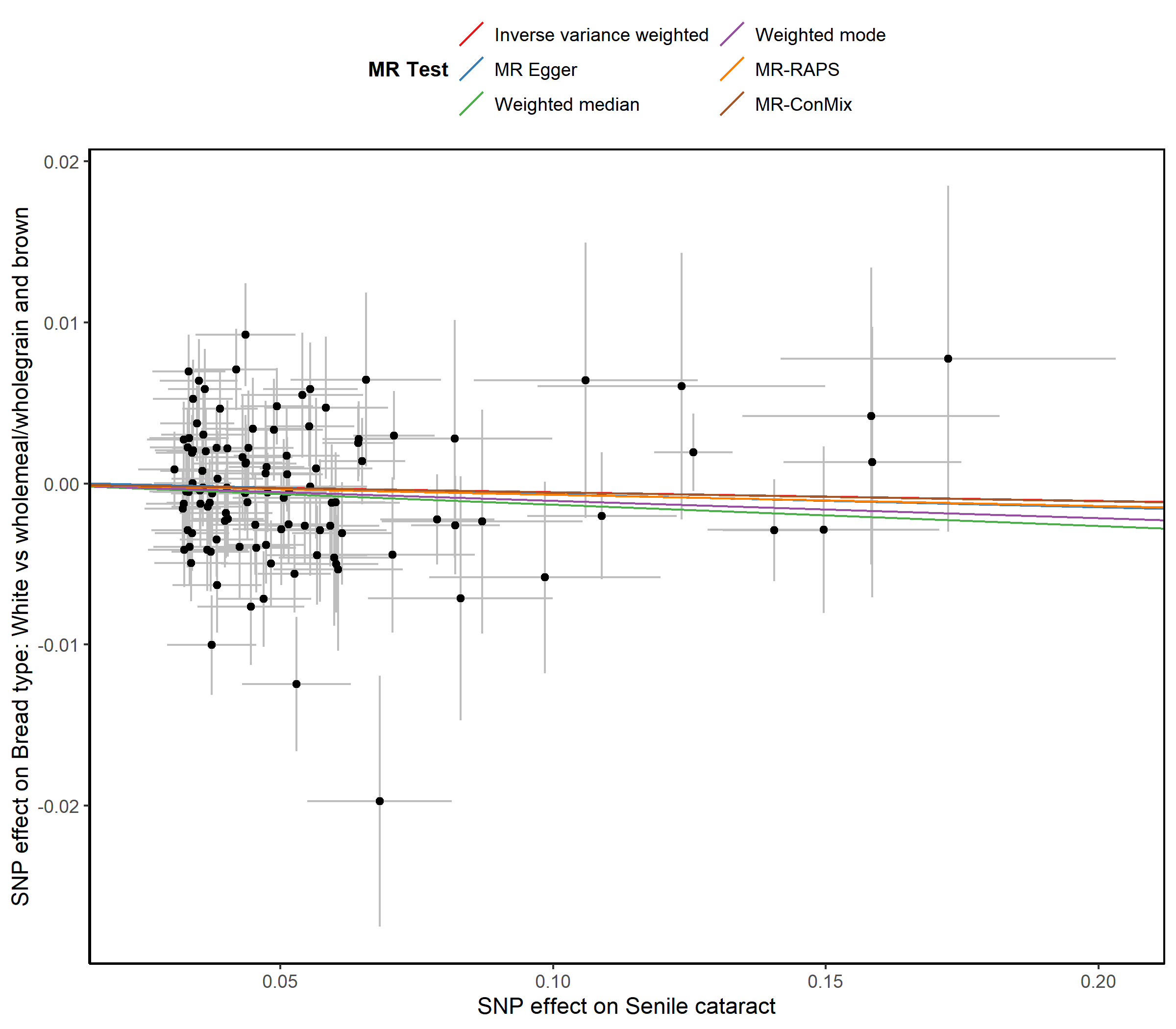


Figure S4.11 Scatter plot of SNPs associated with SC on Bread type: white vs wholemeal/wholegrain and brown.


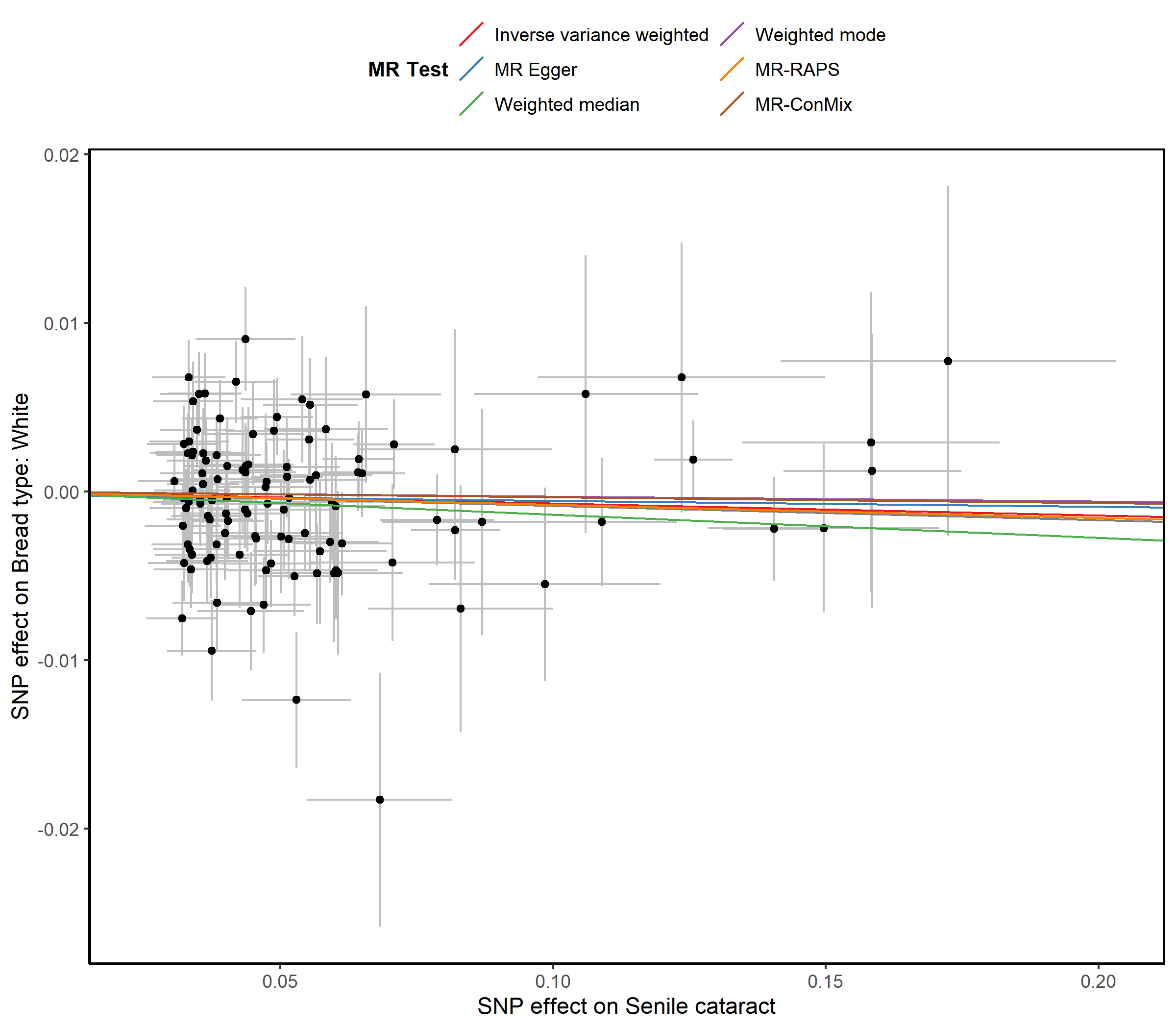


Figure S4.12 Scatter plot of SNPs associated with SC on Bread type: white


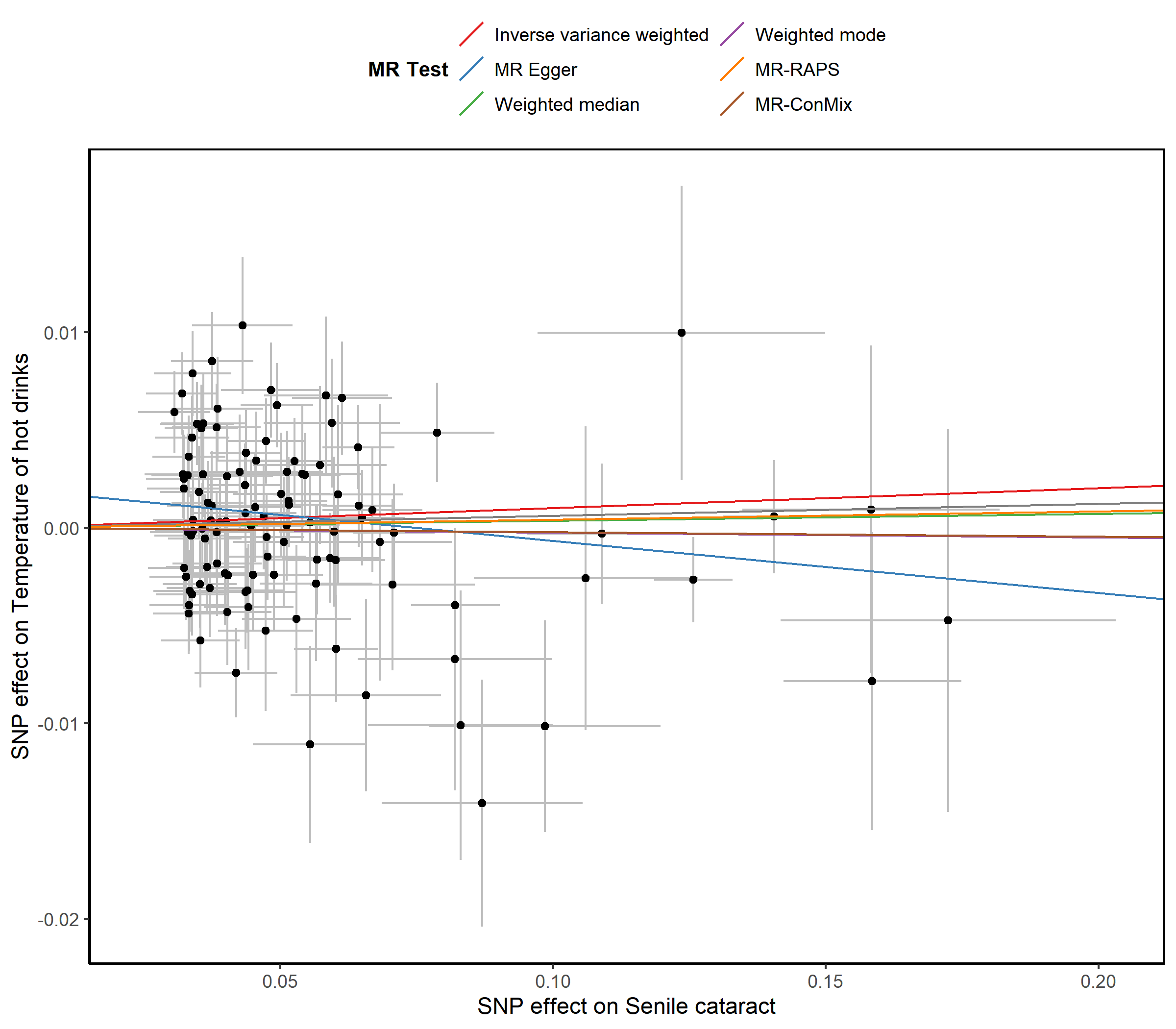


Figure S4.13 Scatter plot of SNPs associated with SC on Temperature of hot drinks.


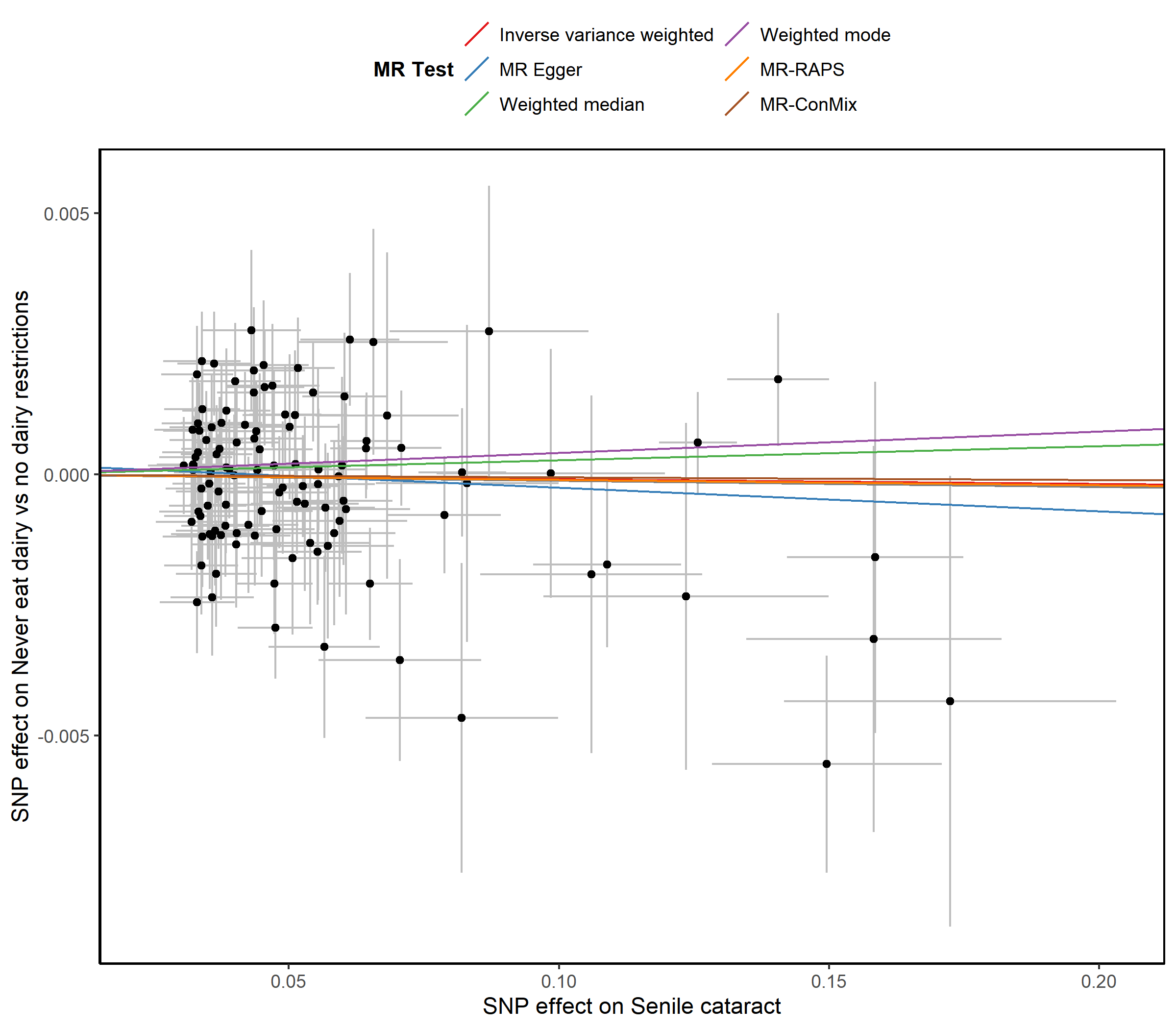

Figure S4.14 Scatter plot of SNPs associated with SC on Never eat dairy vs no dairy restrictions.


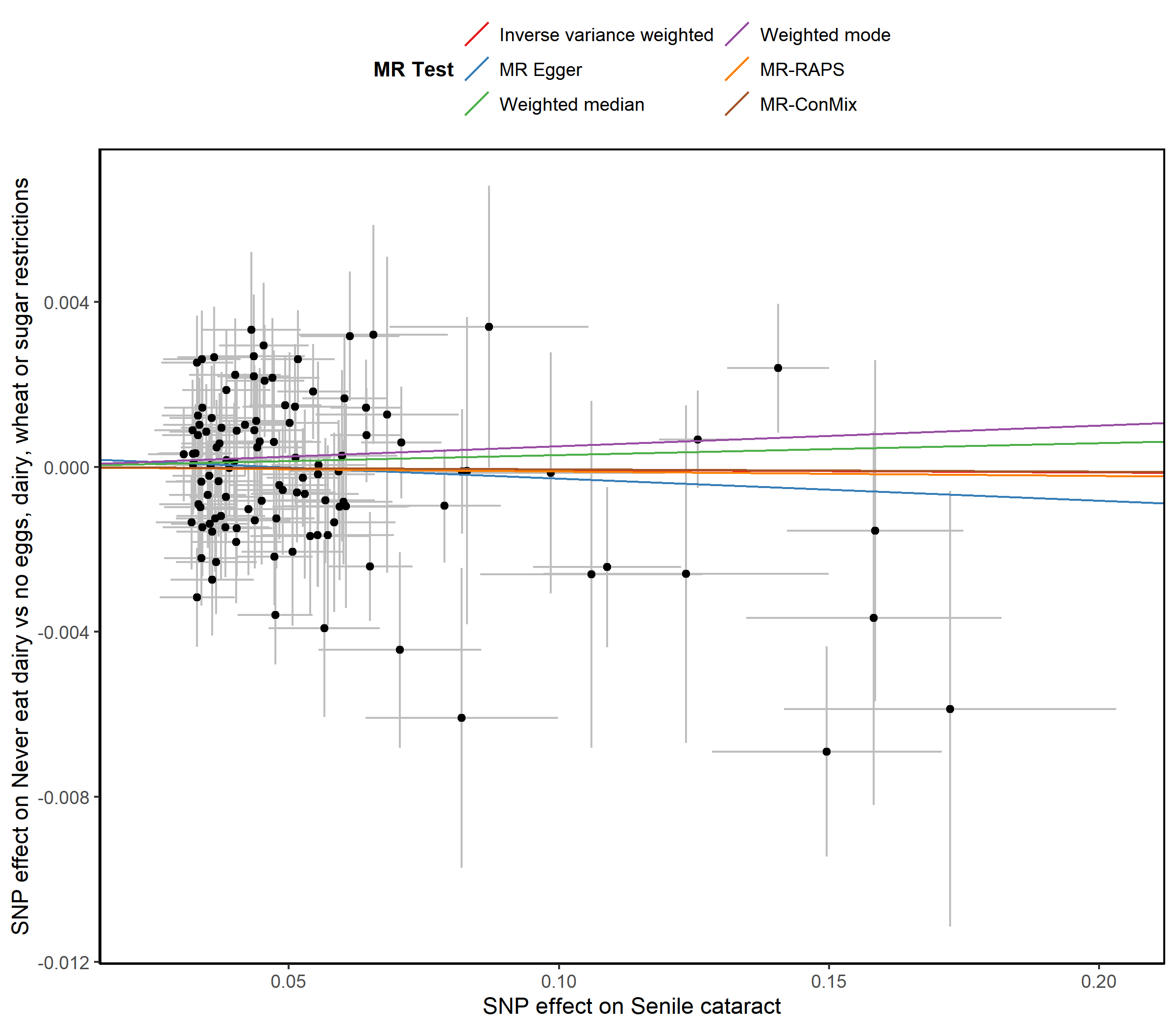


Figure S4.15 Scatter plot of SNPs associated with SC on Never eat dairy vs no eggs, dairy, wheat, or sugar restrictions.


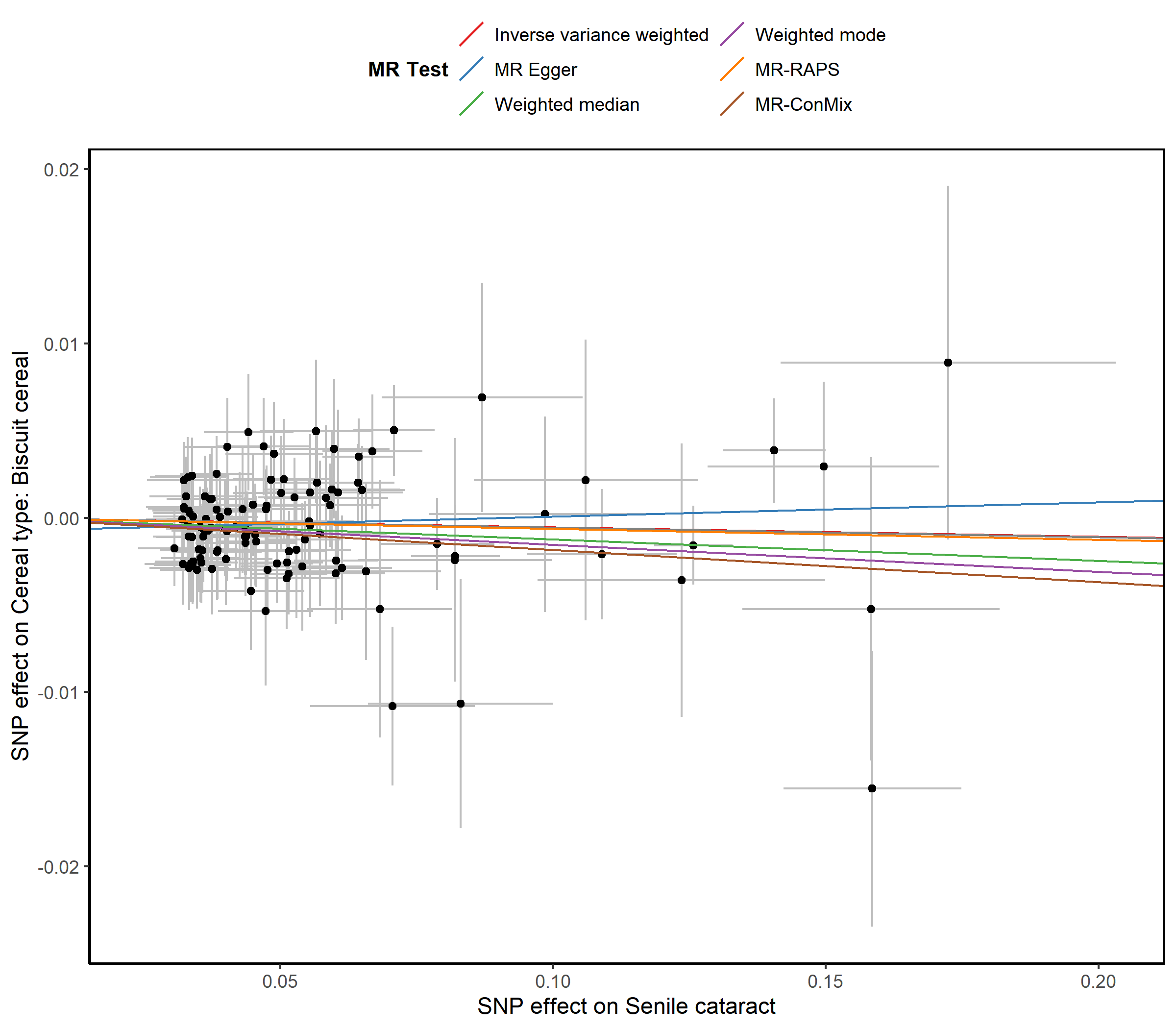


Figure S4.16 Scatter plot of SNPs associated with SC on Cereal type: biscuit cereals.

**Figure S5.** Funnel plot for the association between 16 significant dietary habits and SC in the reverse analysis of bidirectional MR.


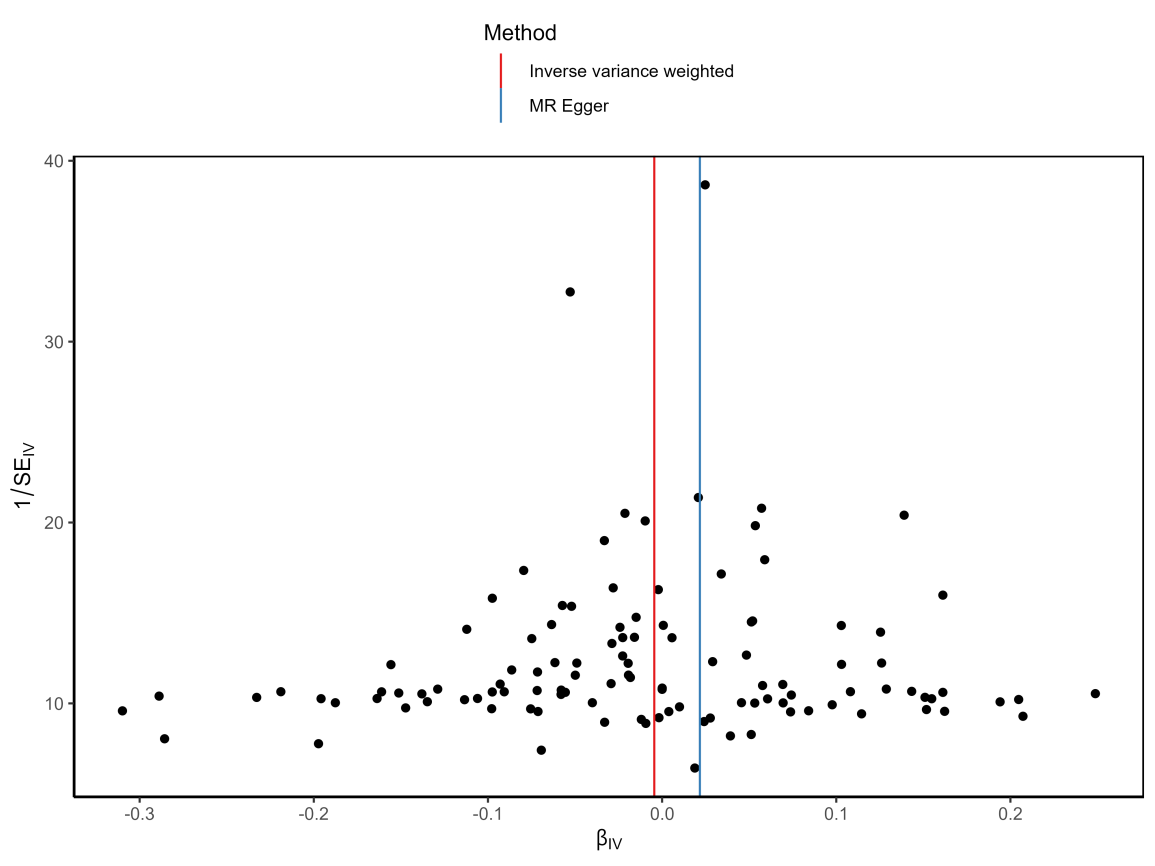


Figure S5.1 Funnel plot of SNPs associated with SC on Drinks usually with meals in current drinkers (yes vs no).


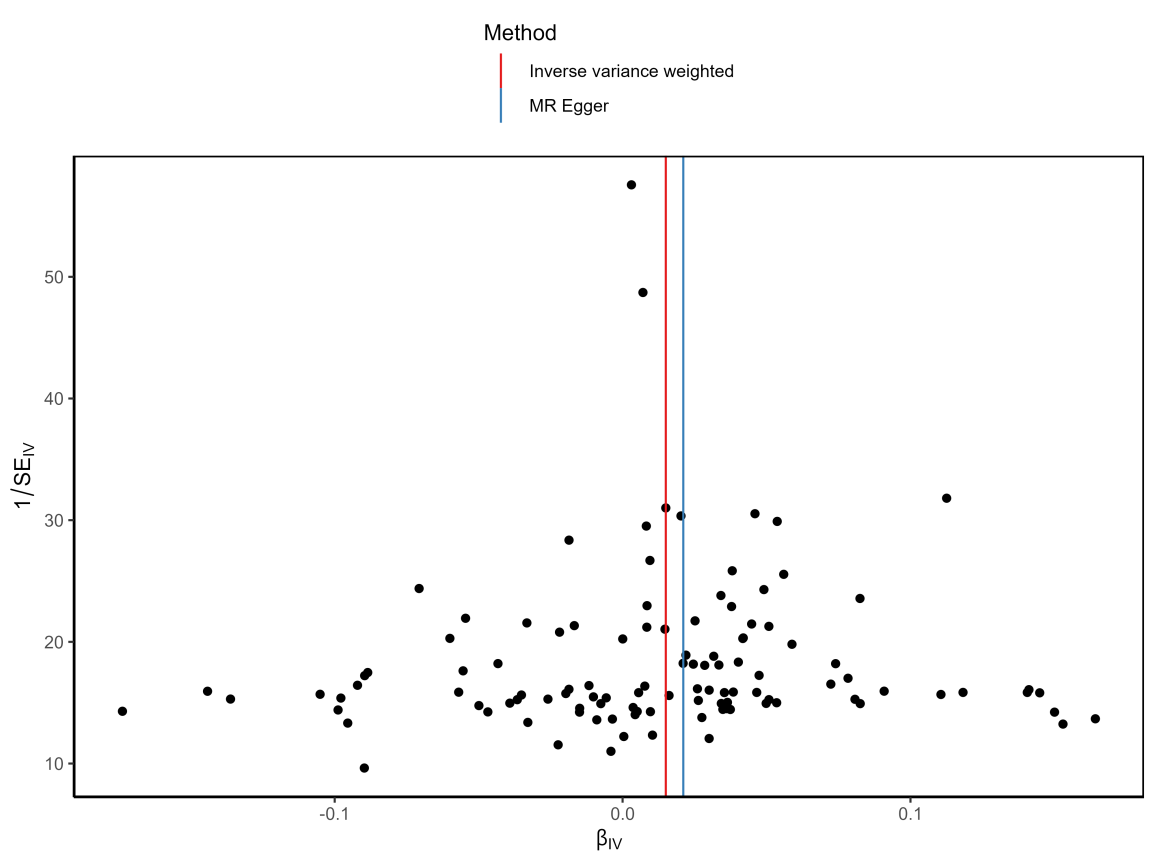


Figure S5.2 Funnel plot of SNPs associated with SC on Cups of tea per day.


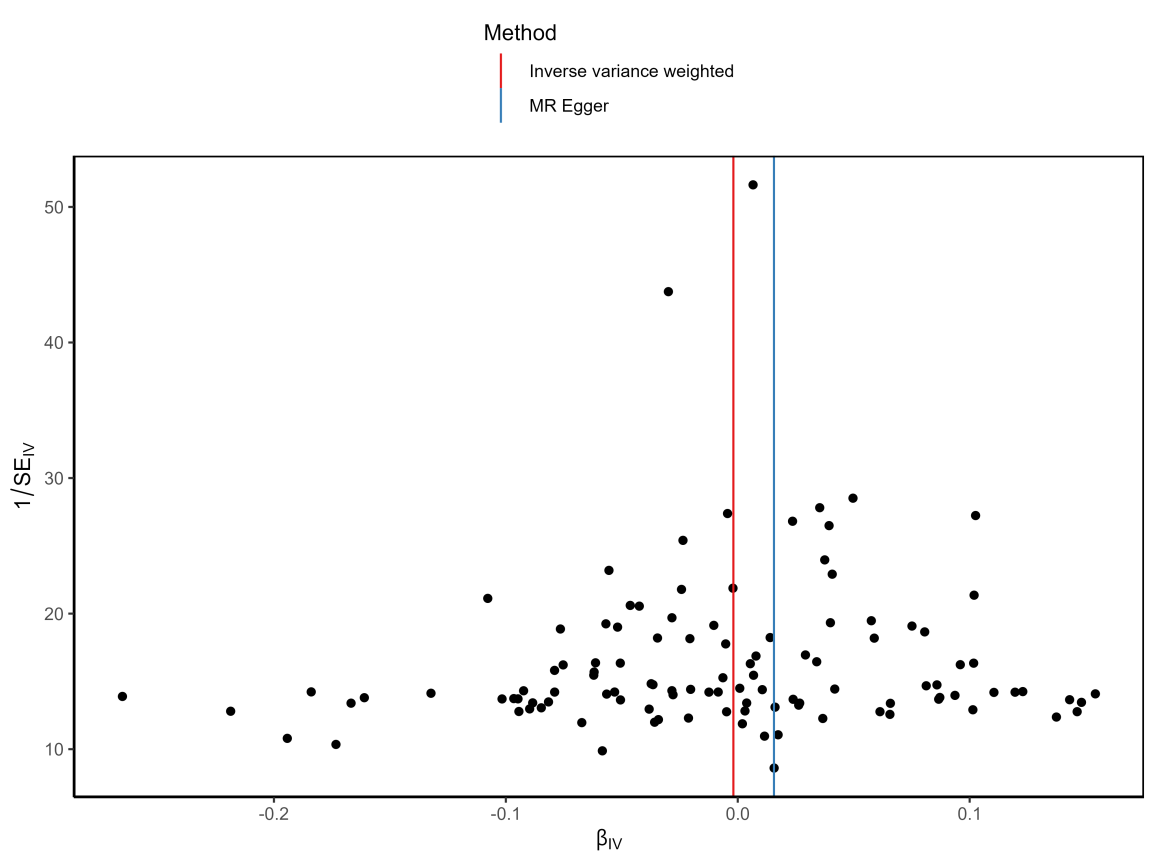


Figure S5.3 Funnel plot of SNPs associated with SC on Alcohol usually taken with meals (yes and it varies vs no).


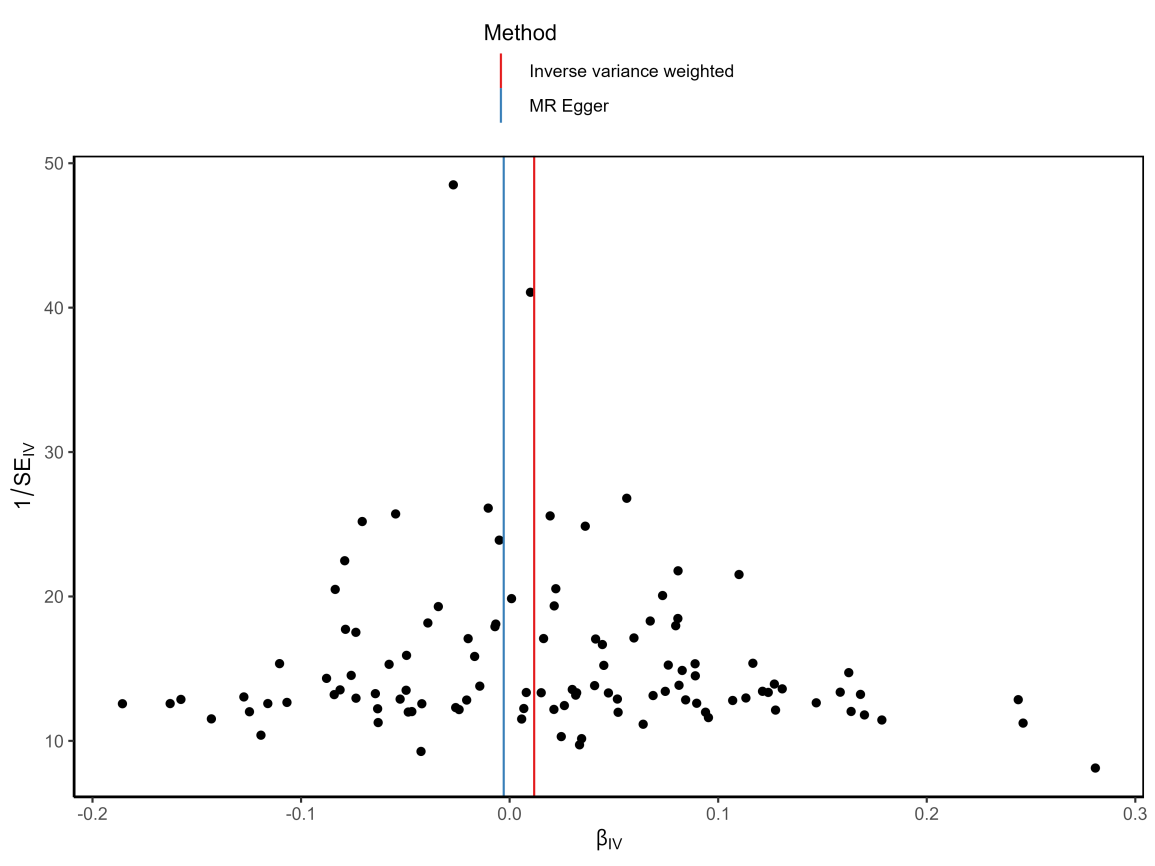


Figure S5.4 Funnel plot of SNPs associated with SC on Bread type: wholemeal/wholegrain vs white and brown.


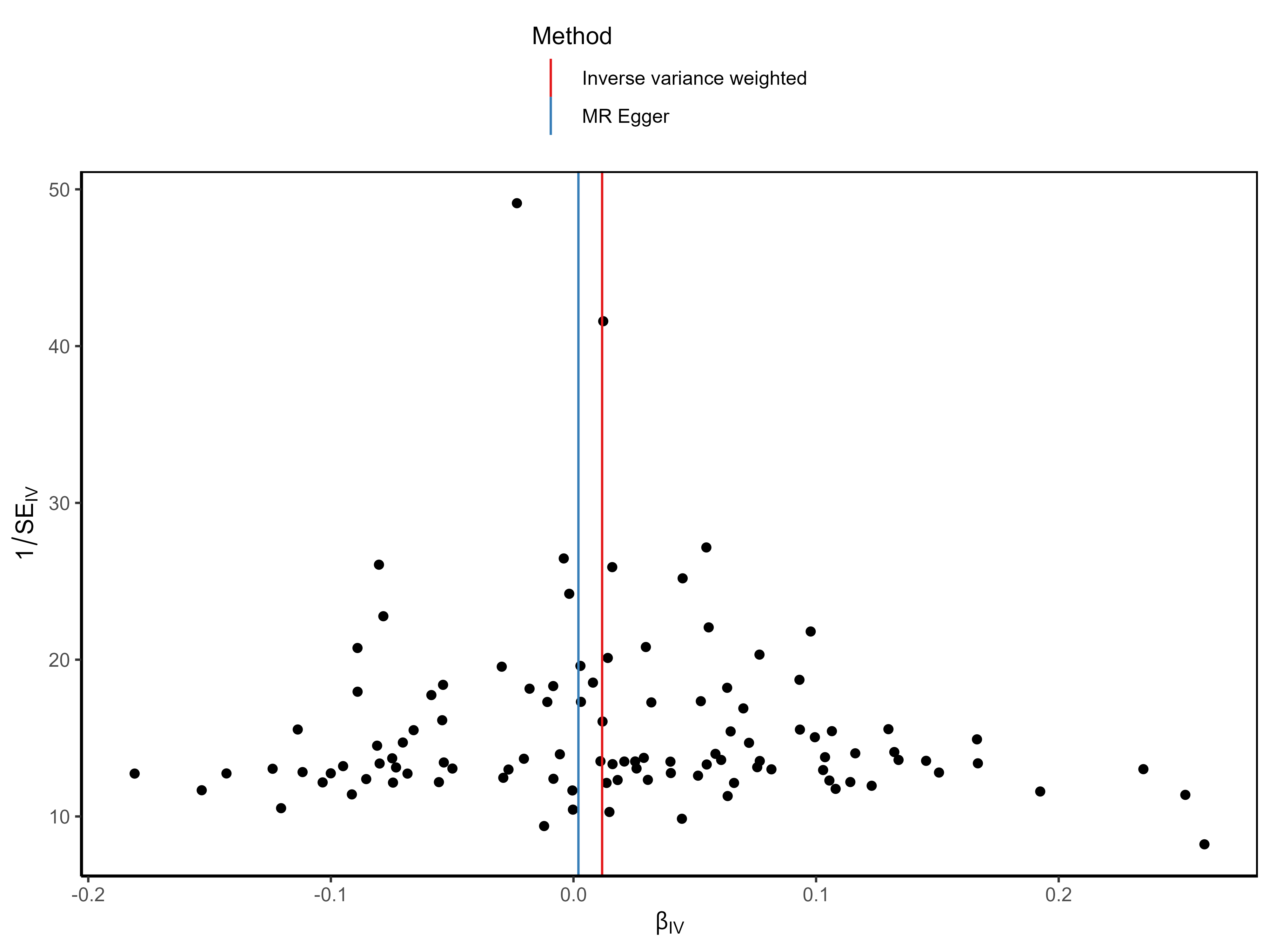


Figure S5.5 Funnel plot of SNPs associated with SC on Bread type: wholemeal or wholegrain.


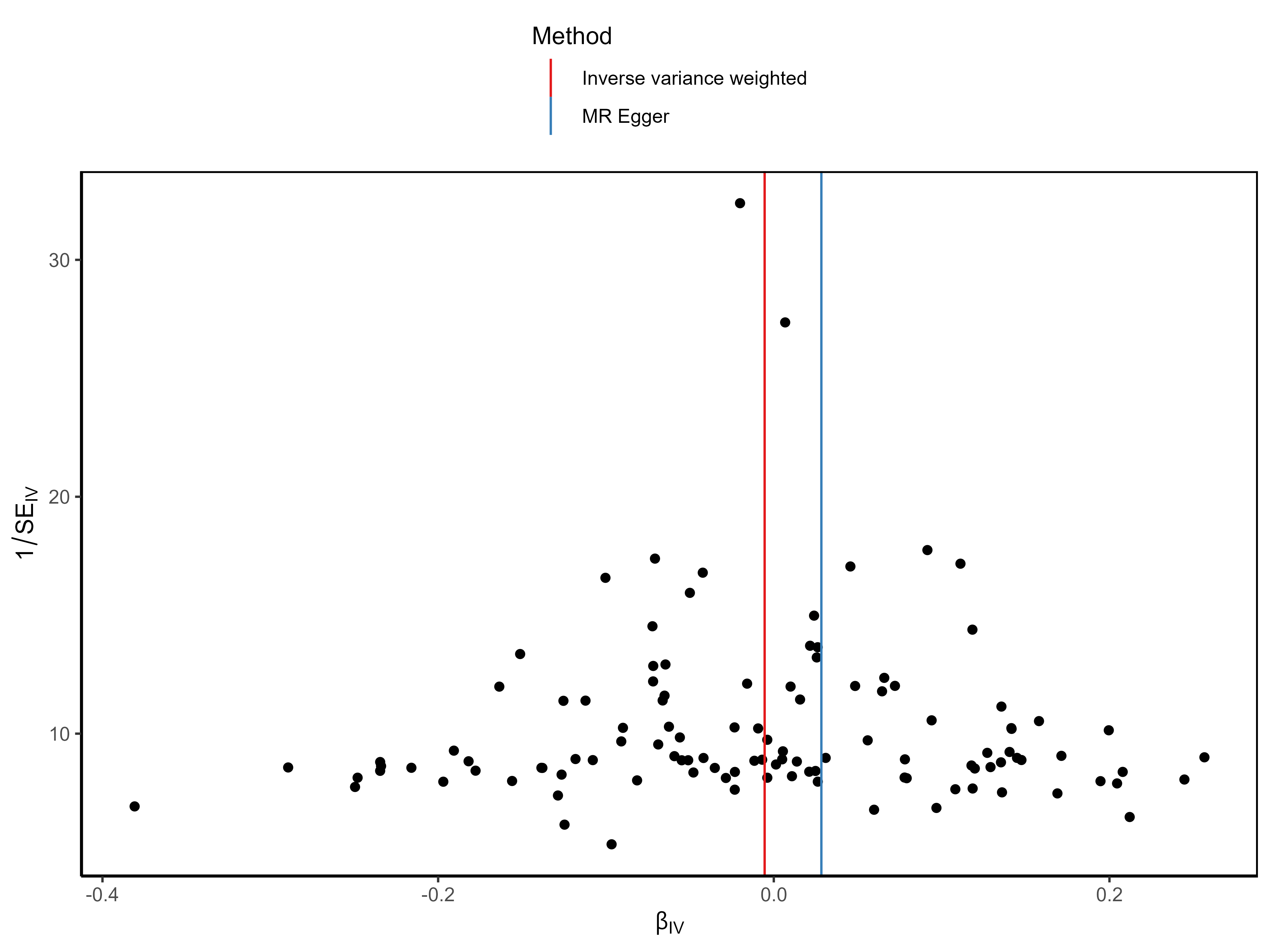


Figure S5.6 Funnel plot of SNPs associated with SC on Milk type used: skimmed vs never have milk.


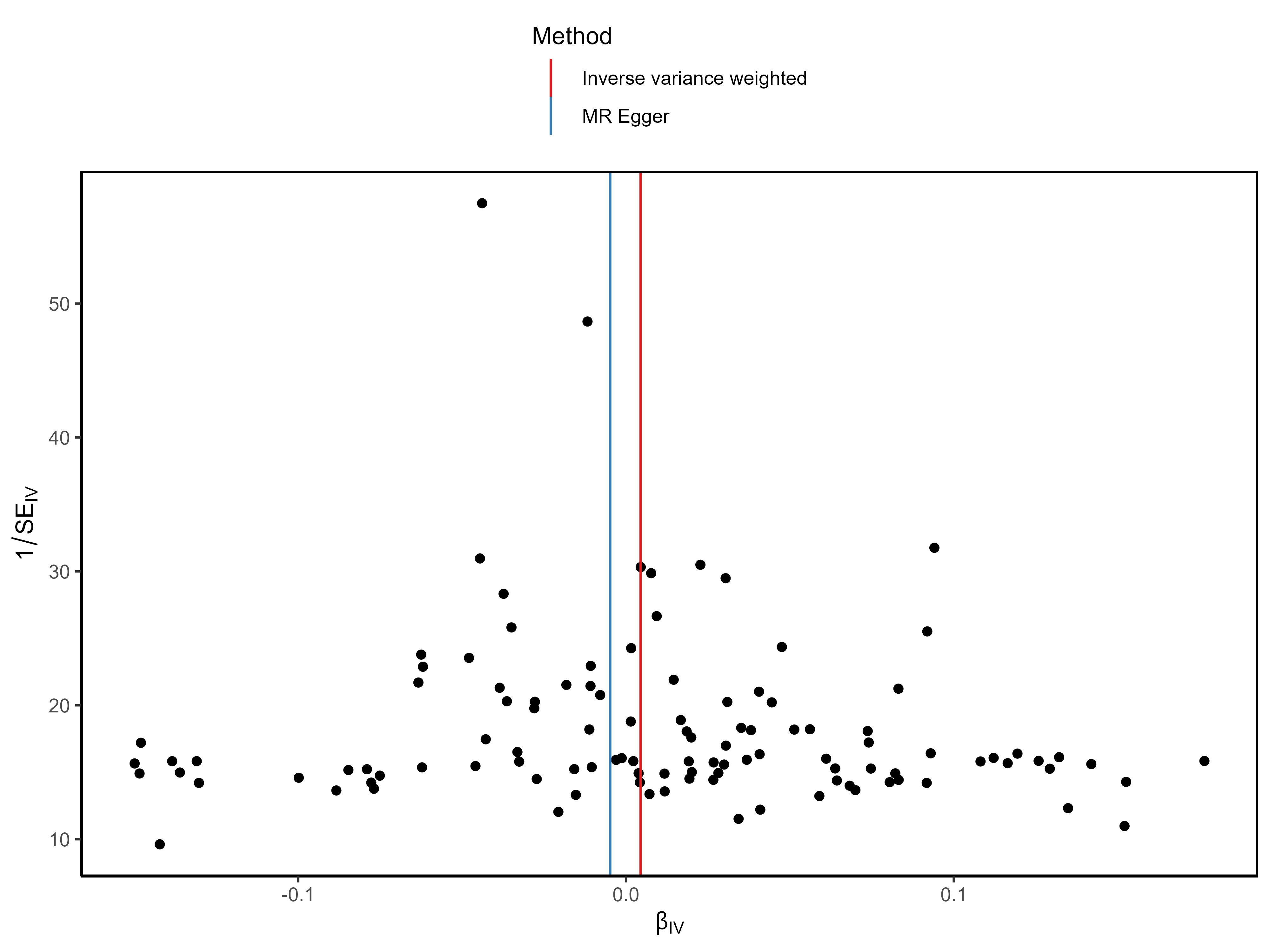


Figure S5.7 Funnel plot of SNPs associated with SC on Cereal consumption (bowls per week).


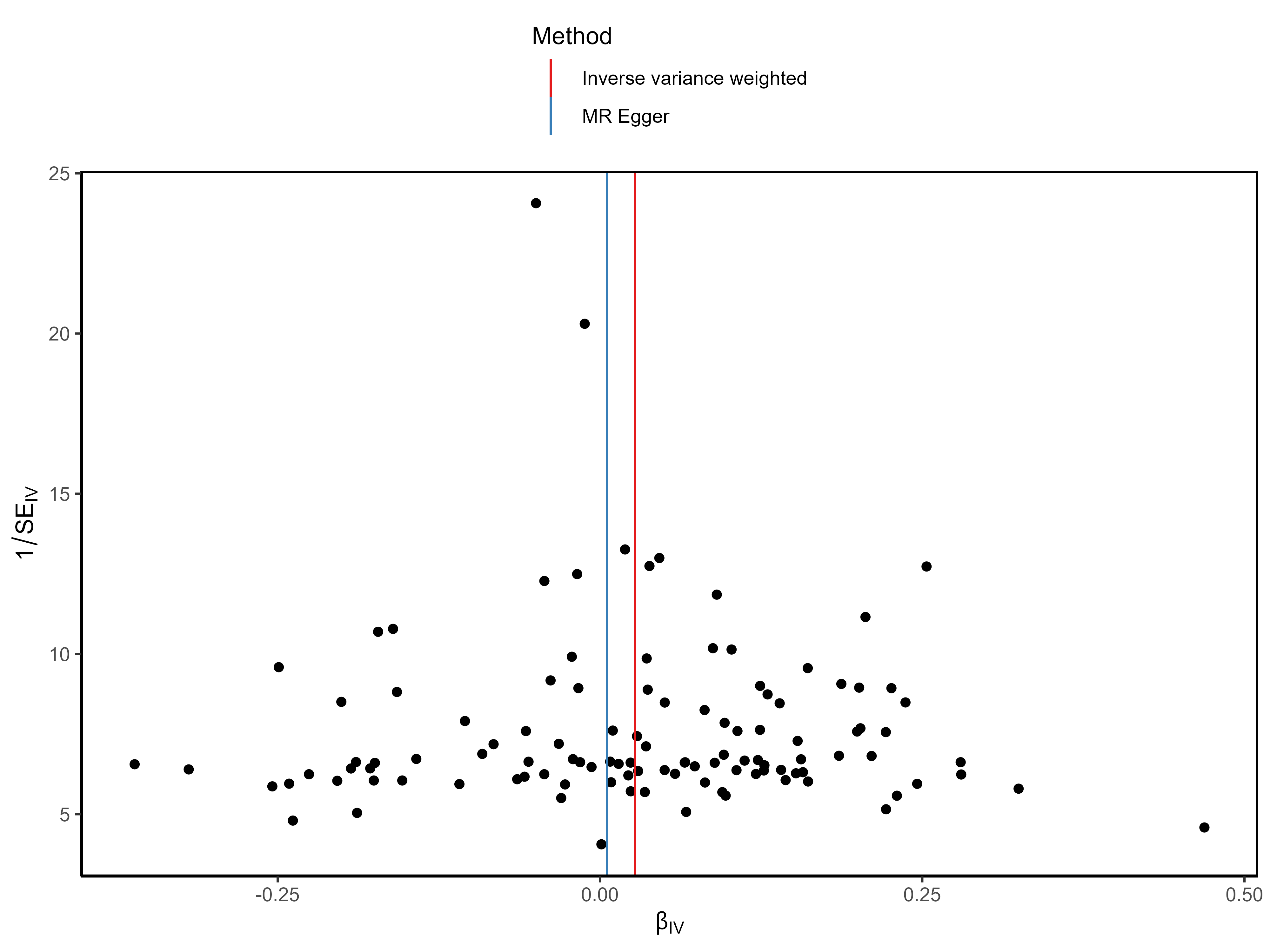


Figure S5.8 Funnel plot of SNPs associated with SC on Spread type: olive oil spread vs never use spread.


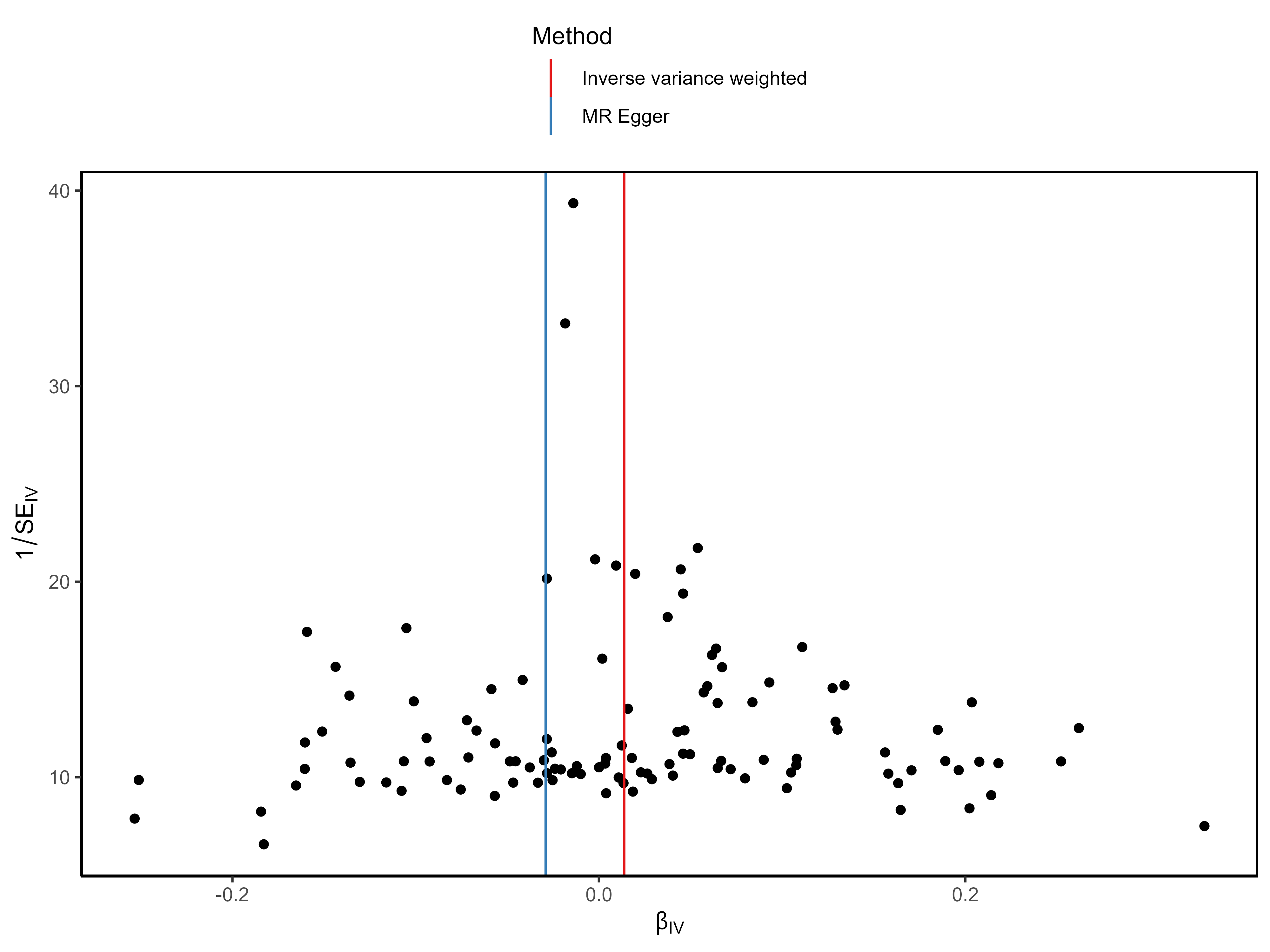


Figure S5.9 Funnel plot of SNPs associated with SC on Spread type: butter vs never use spread.


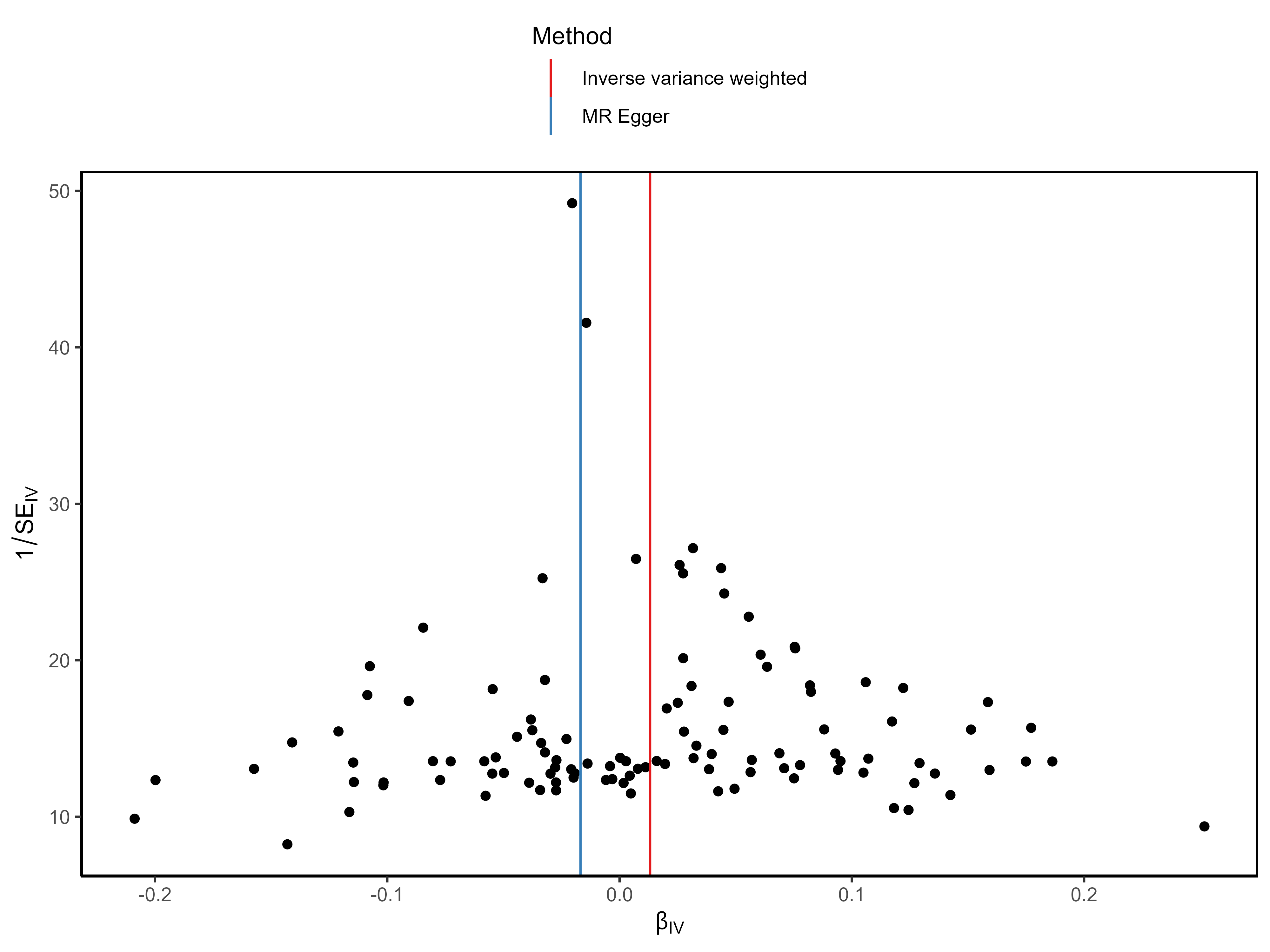


Figure S5.10 Funnel plot of SNPs associated with SC on Spread type: butter and margarine vs never use spread.


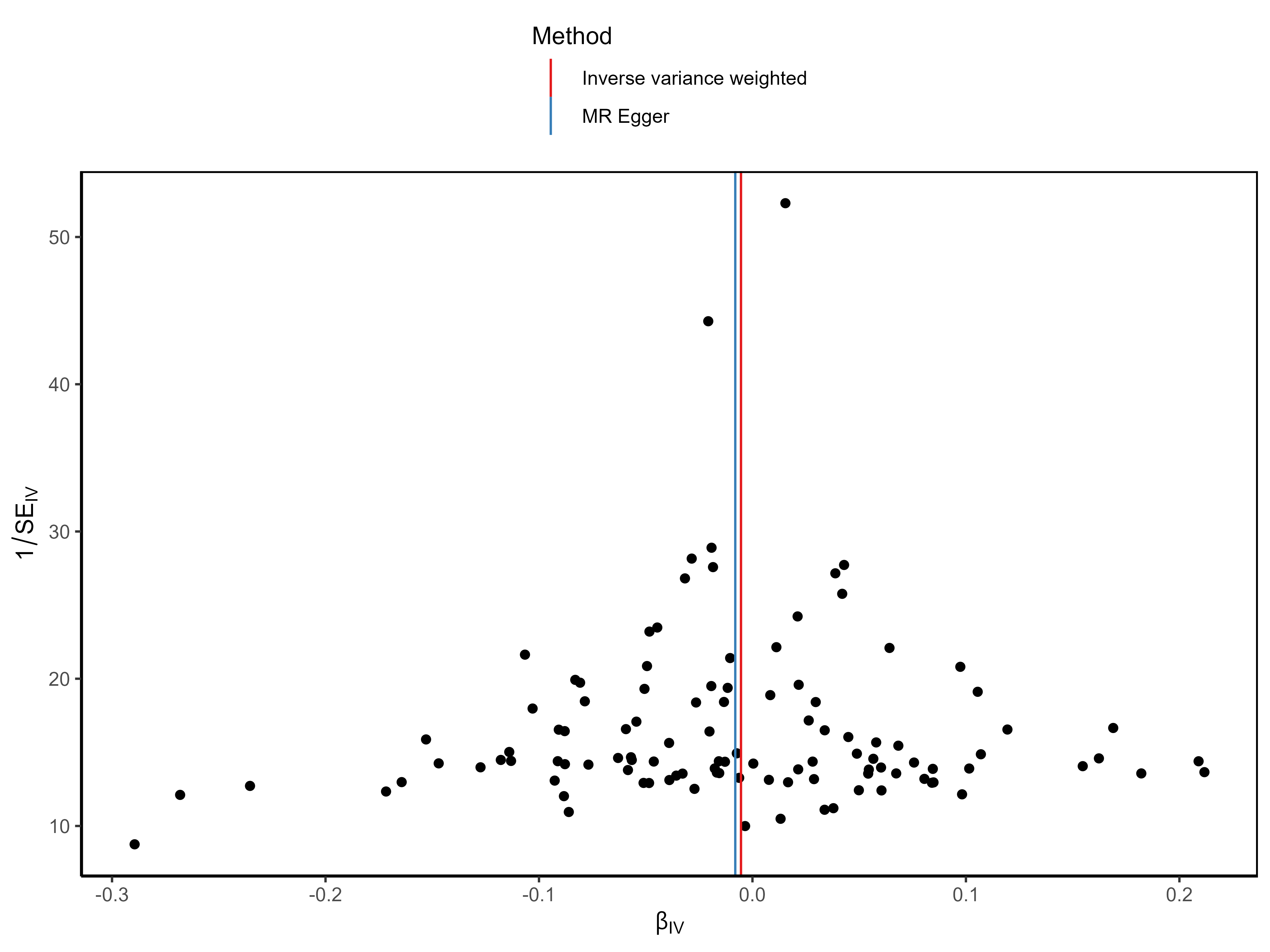


Figure S5.11 Funnel plot of SNPs associated with SC on Bread type: white vs wholemeal/wholegrain and brown.


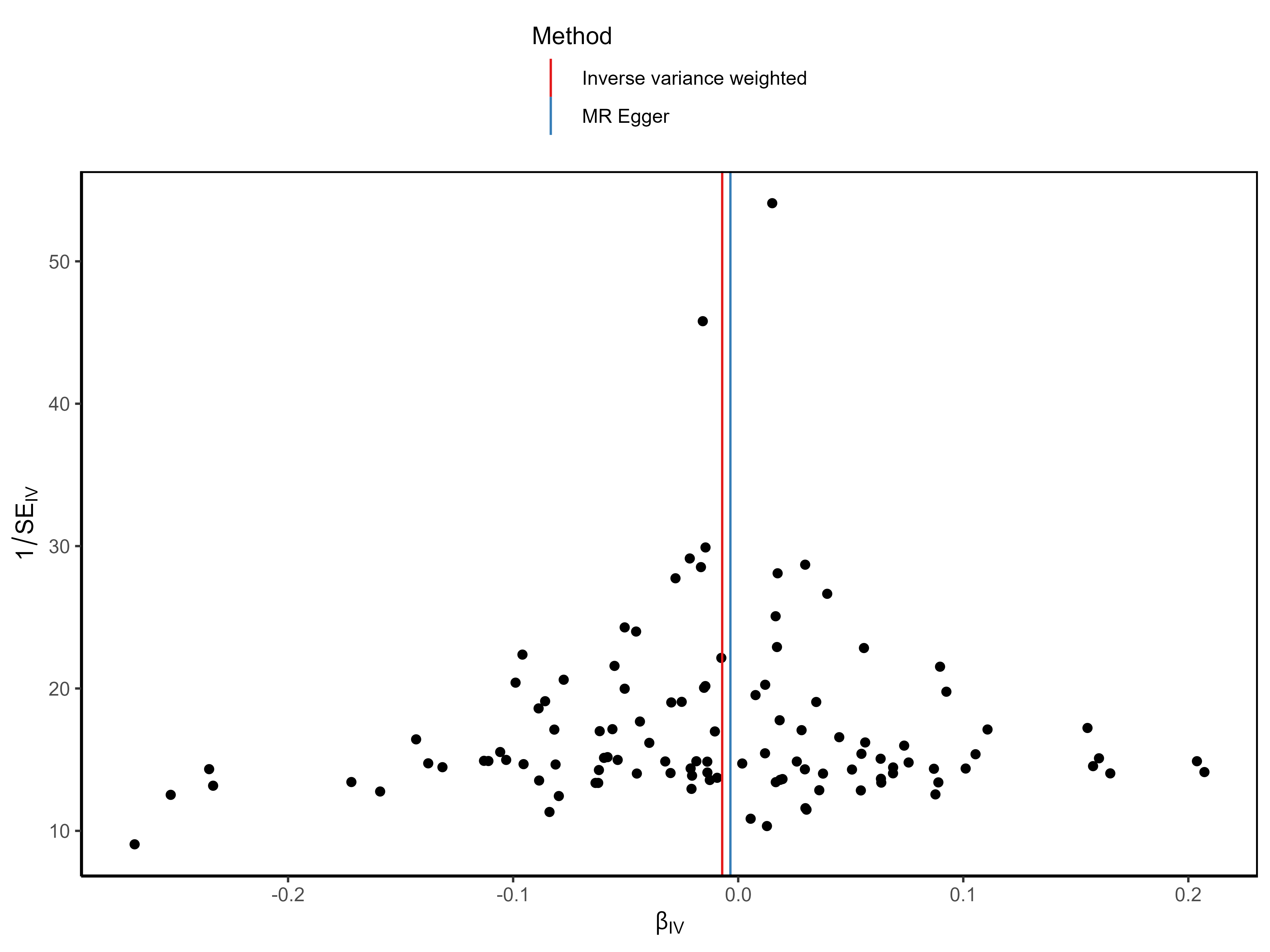


Figure S5.12 Funnel plot of SNPs associated with SC on Bread type: white.


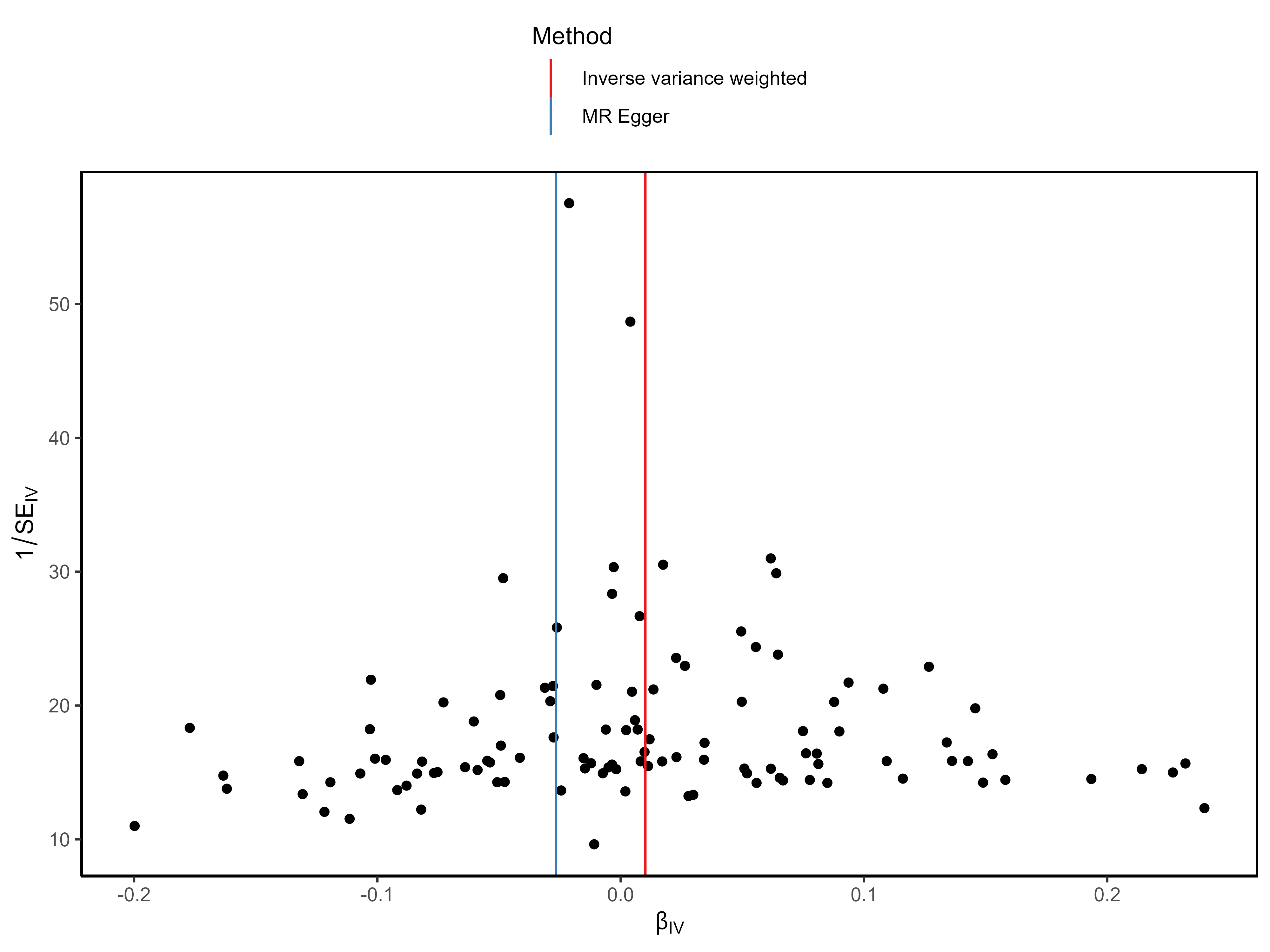


Figure S5.13 Funnel plot of SNPs associated with SC on Temperature of hot drinks.


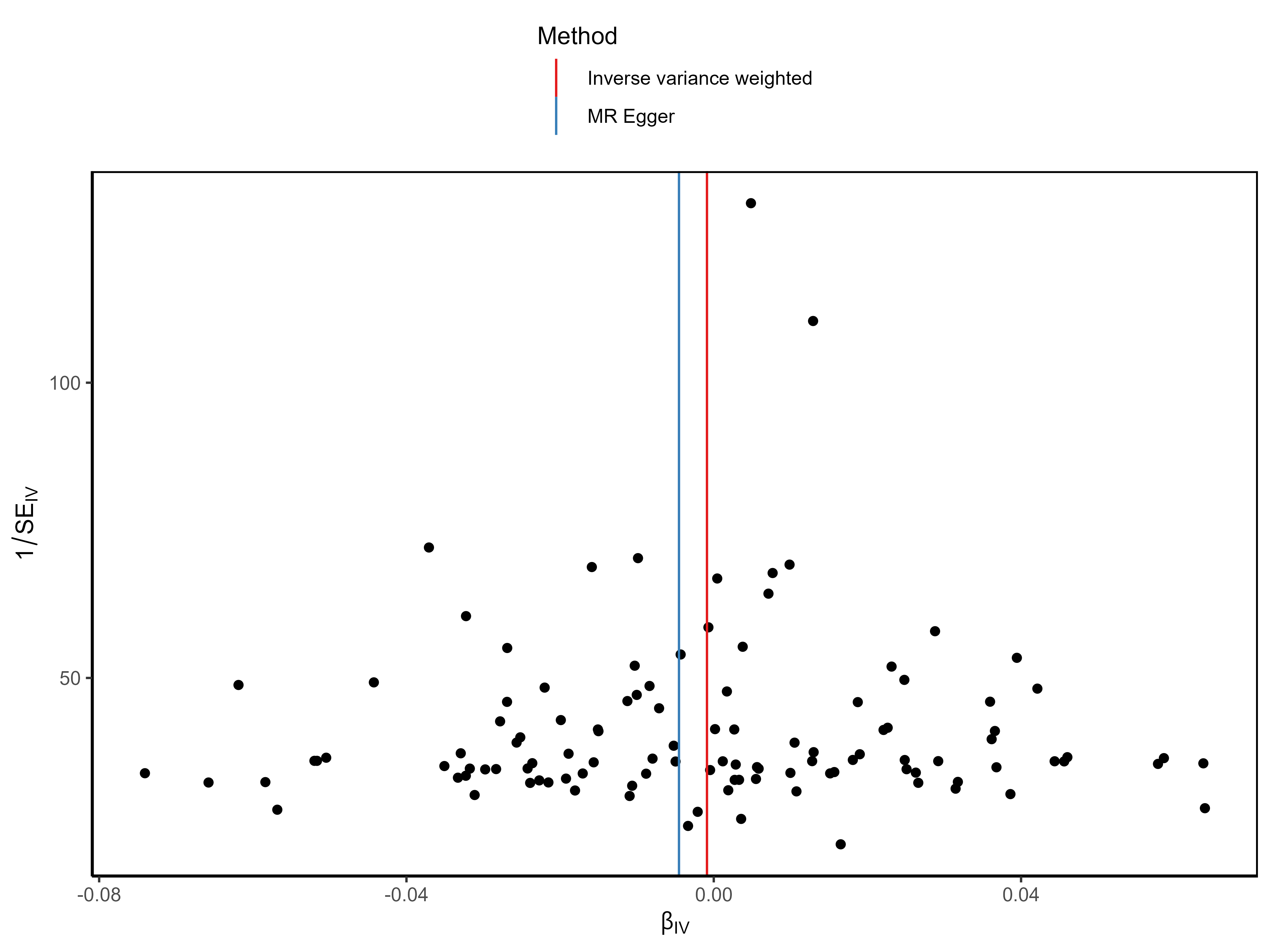


Figure S5.14 Funnel plot of SNPs associated with SC on Never eat dairy vs no dairy restrictions.


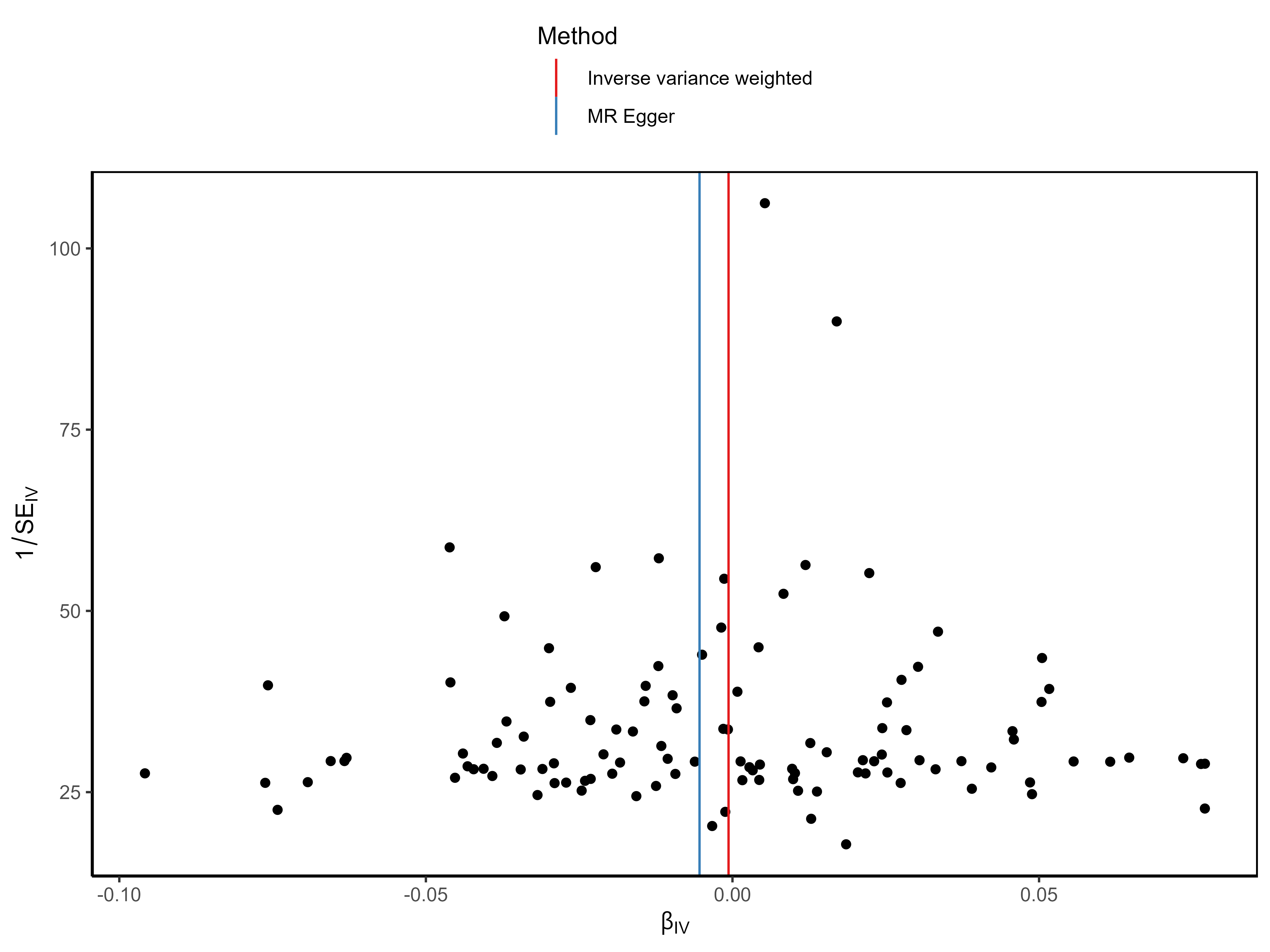


Figure S5.15 Funnel plot of SNPs associated with SC on Never eat dairy vs no eggs, dairy, wheat, or sugar restrictions.


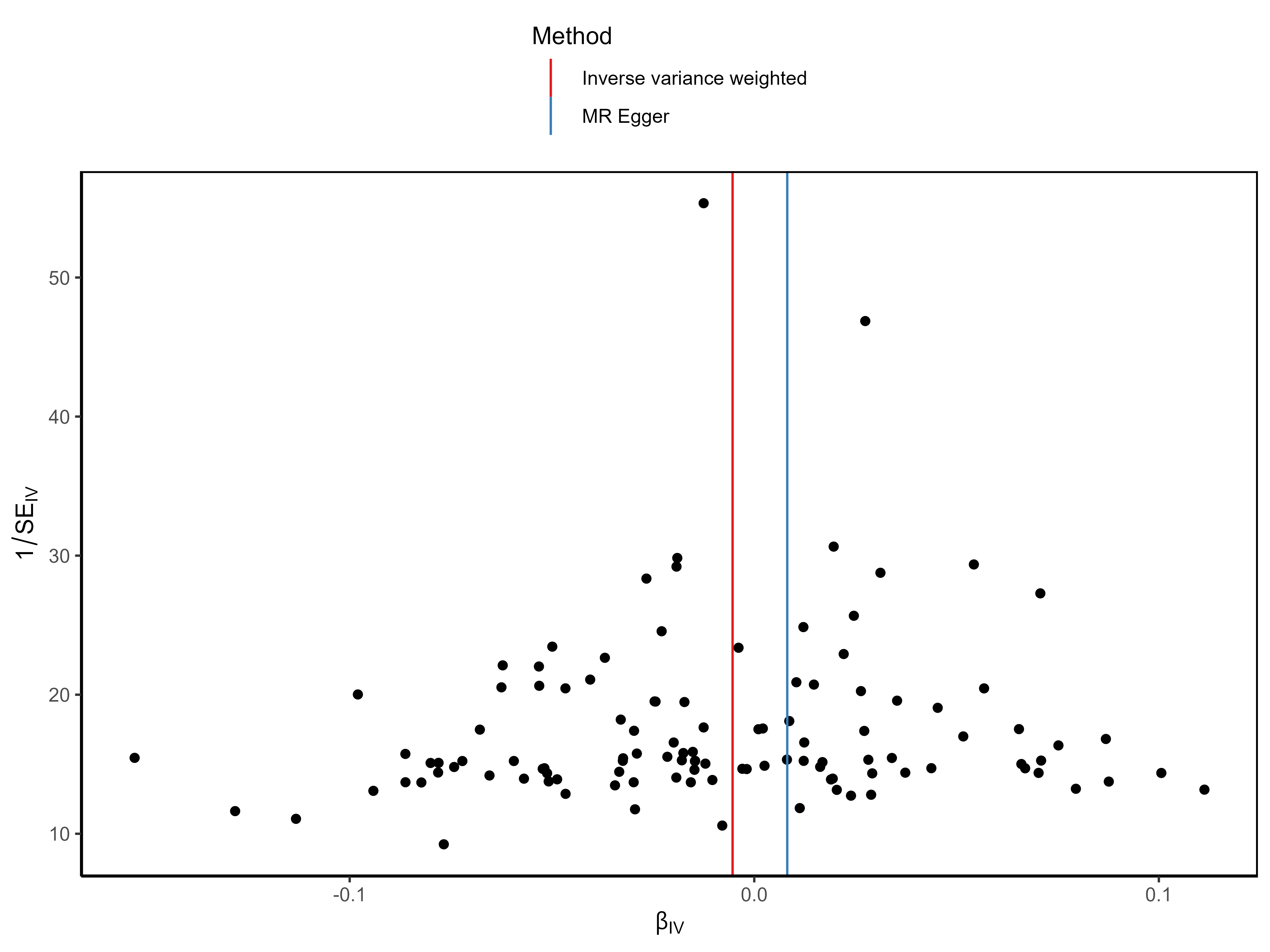


Figure S5.16 Funnel plot of SNPs associated with SC on Cereal type: biscuit cereals.

**Figure S6.** Leave-one-out analysis for the association between 16 significant dietary habits and SC in the reverse analysis of bidirectional MR.


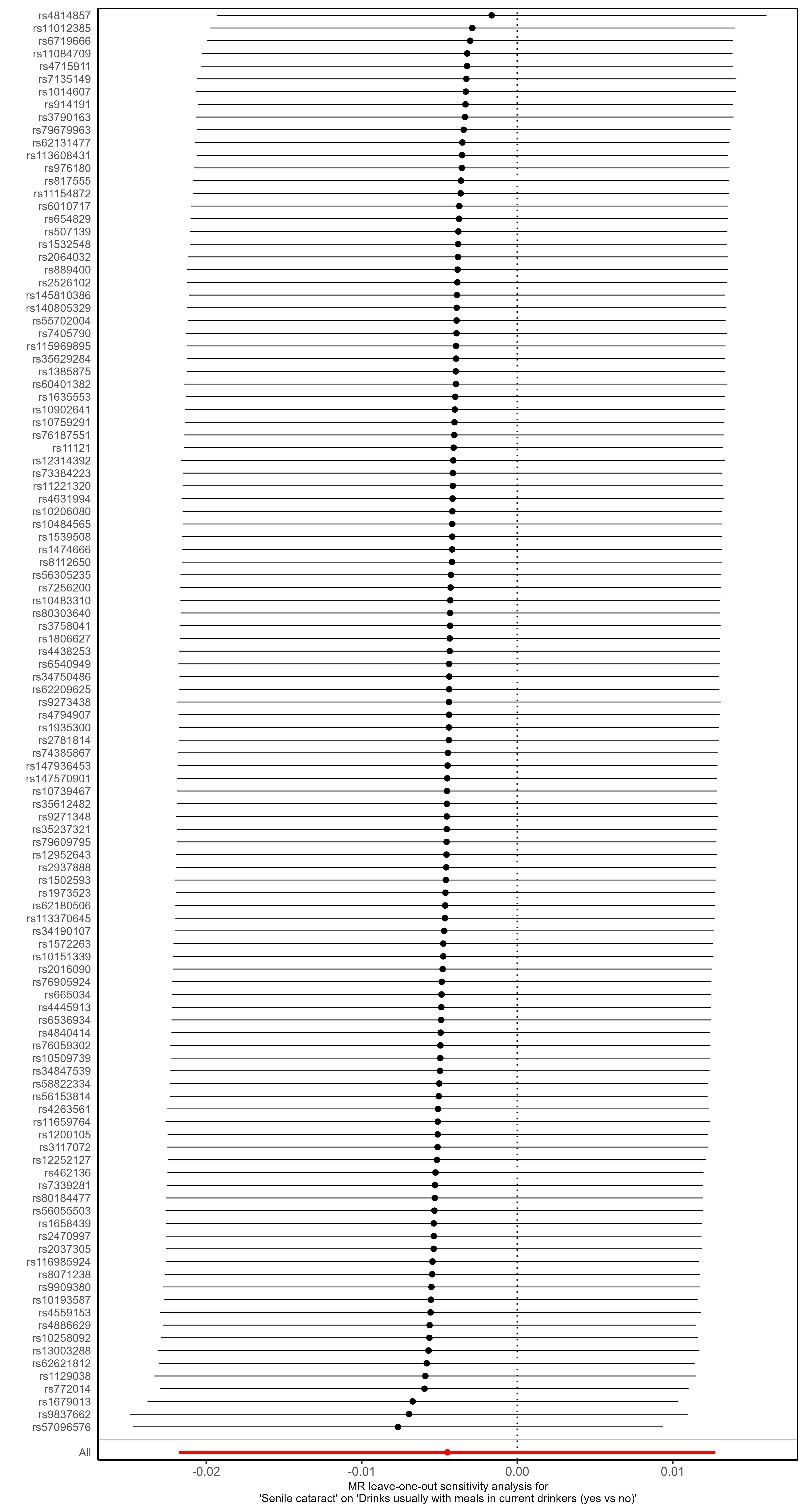


Figure S6.1 Leave-one-out analysis of SNPs associated with SC on Drinks usually with meals in current drinkers (yes vs no).


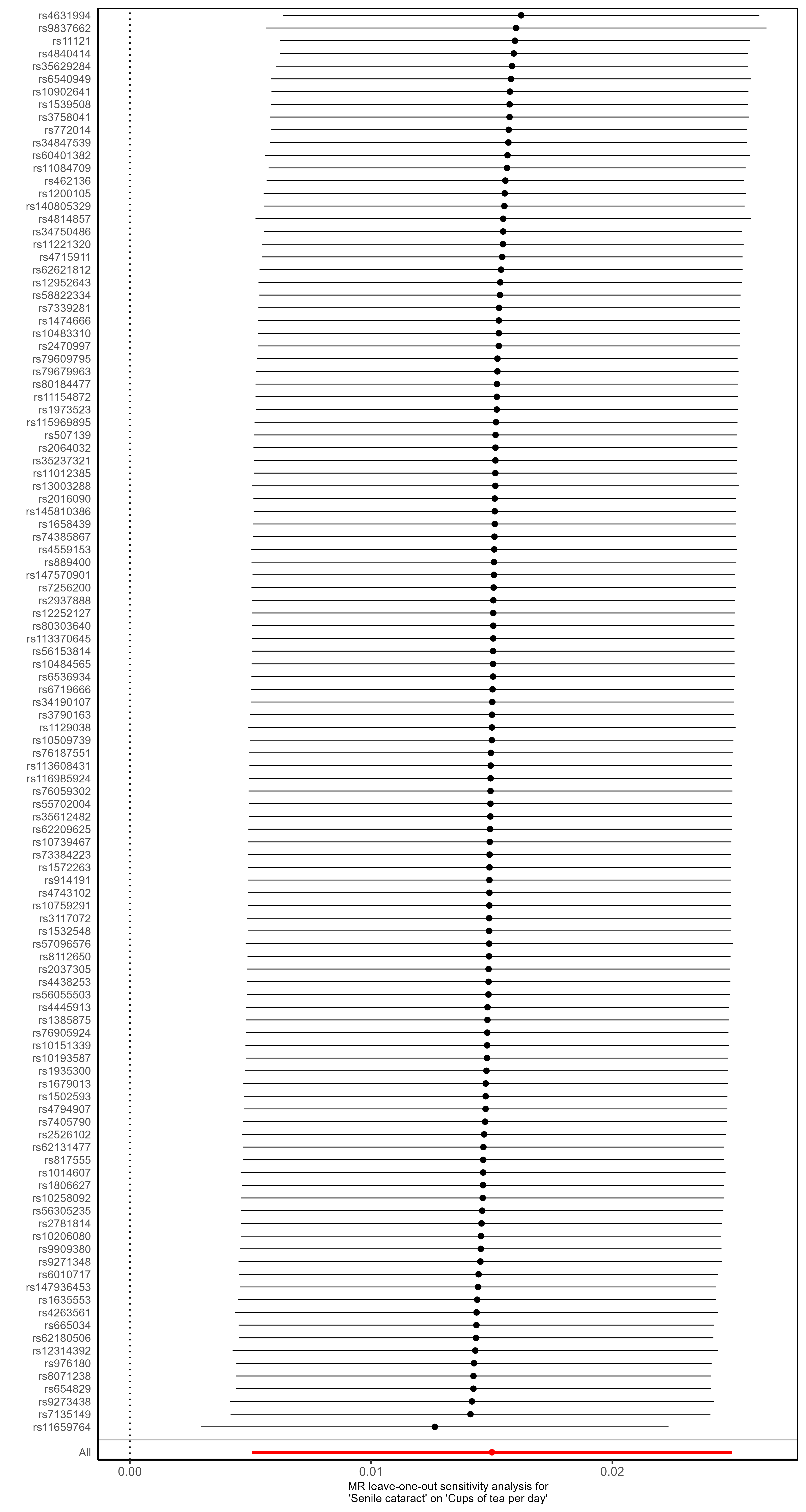


Figure S6.2 Leave-one-out analysis of SNPs associated with SC on Cups of tea per day.


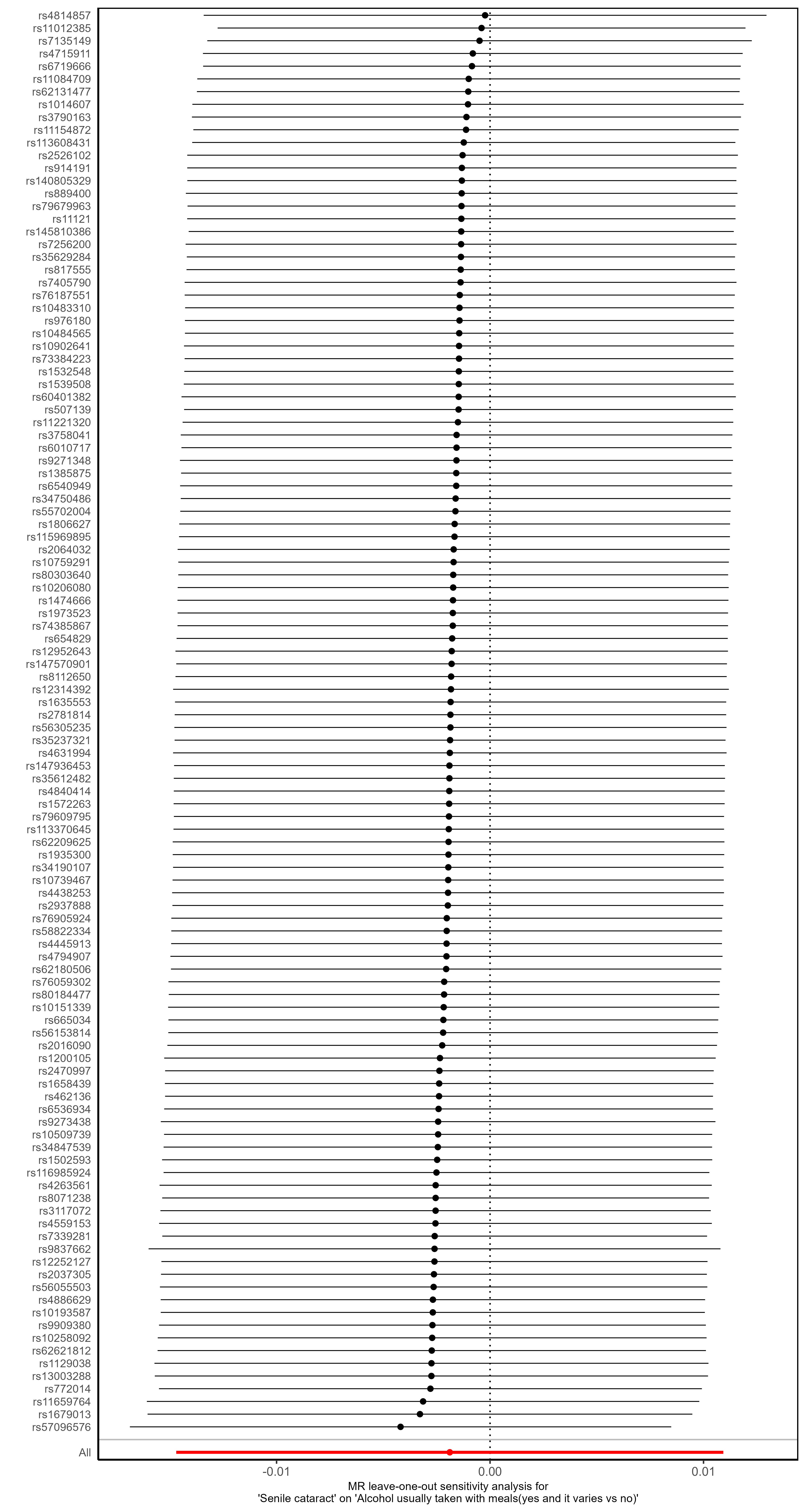


Figure S6.3 Leave-one-out analysis of SNPs associated with SC on Alcohol usually taken with meals (yes and it varies vs no).


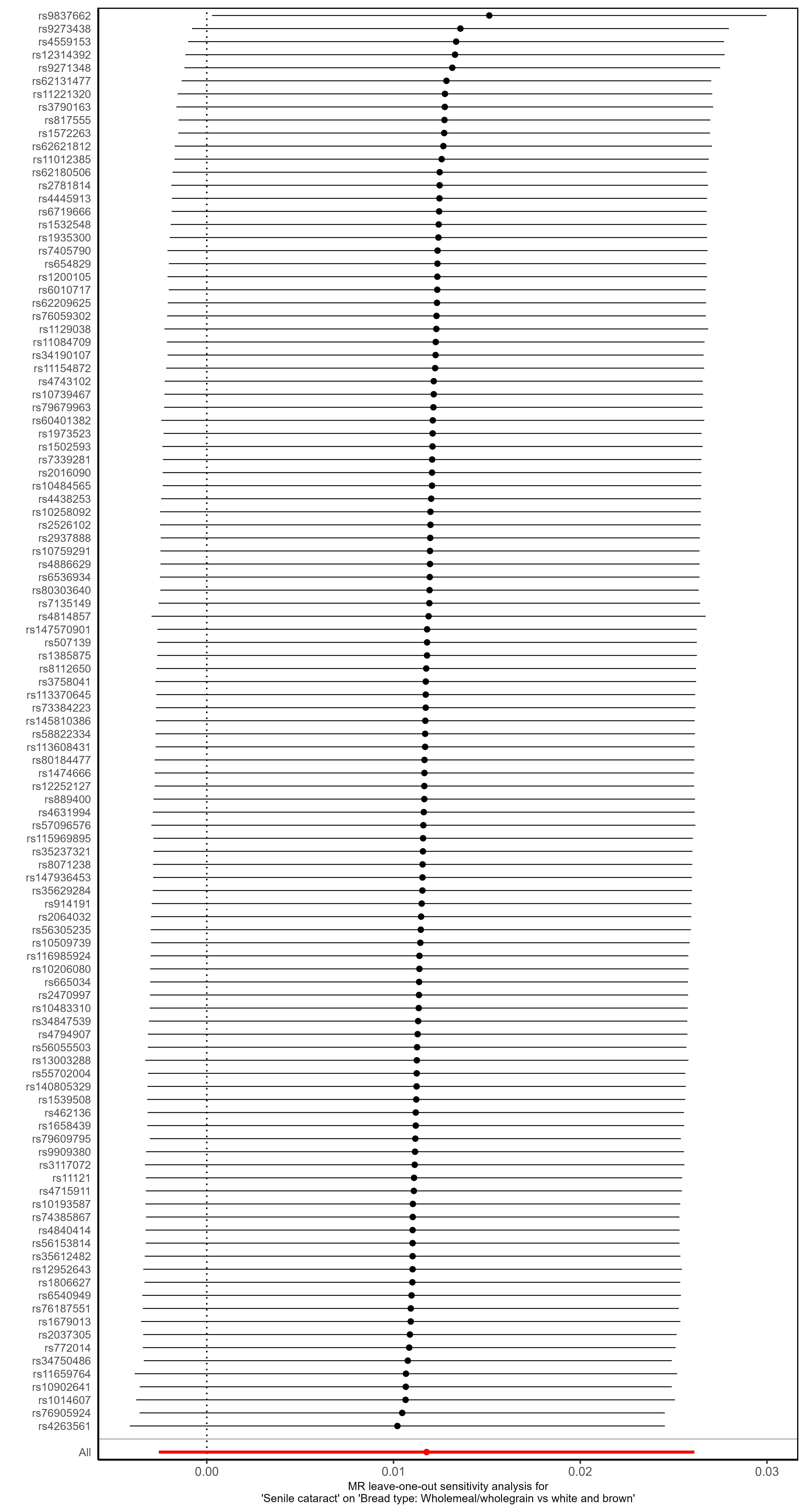


Figure S6.4 Leave-one-out analysis of SNPs associated with SC on Bread type: wholemeal/wholegrain vs white and brown.


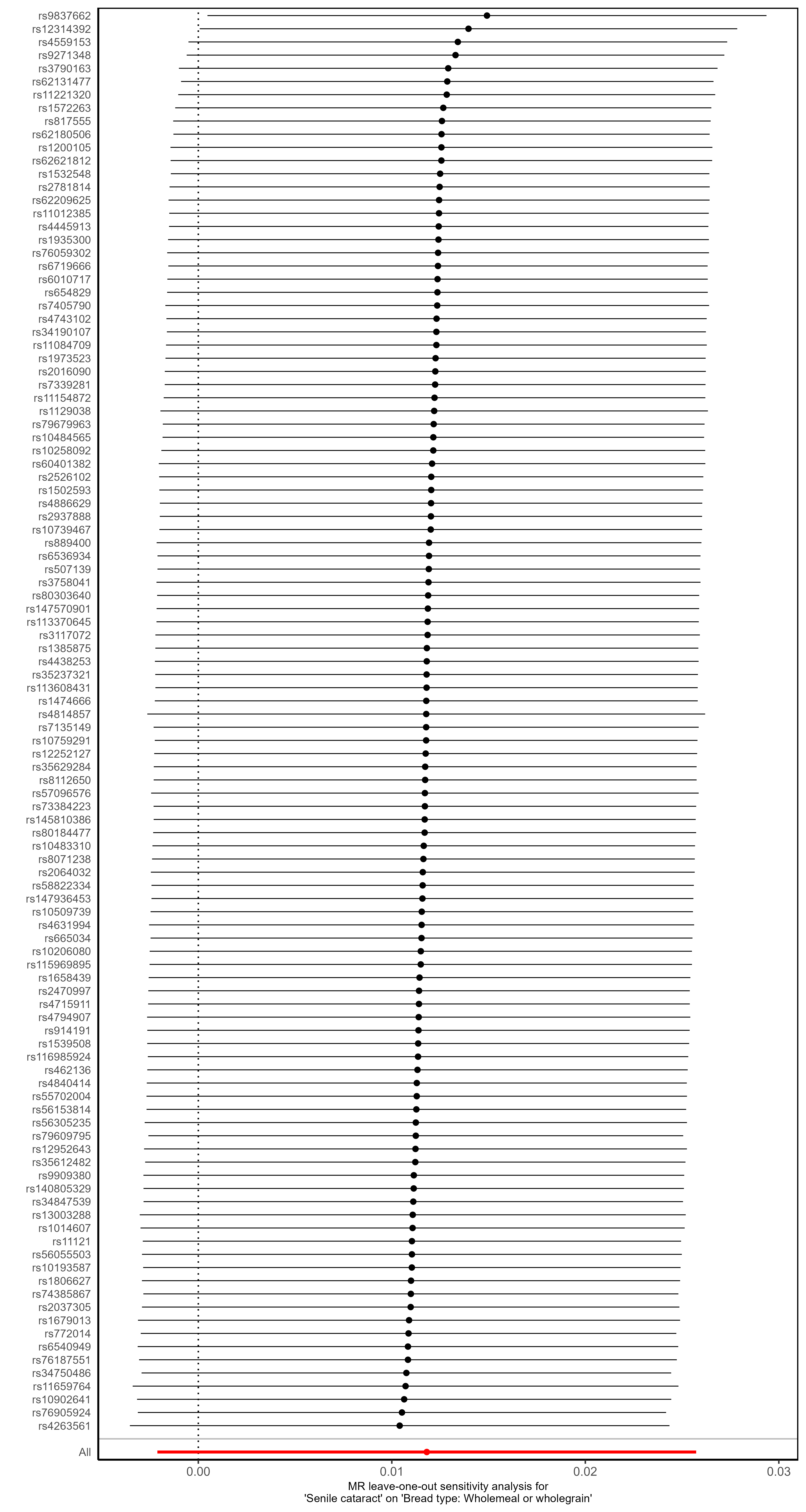


Figure S6.5 Leave-one-out analysis of SNPs associated with SC on Bread type: wholemeal or wholegrain.


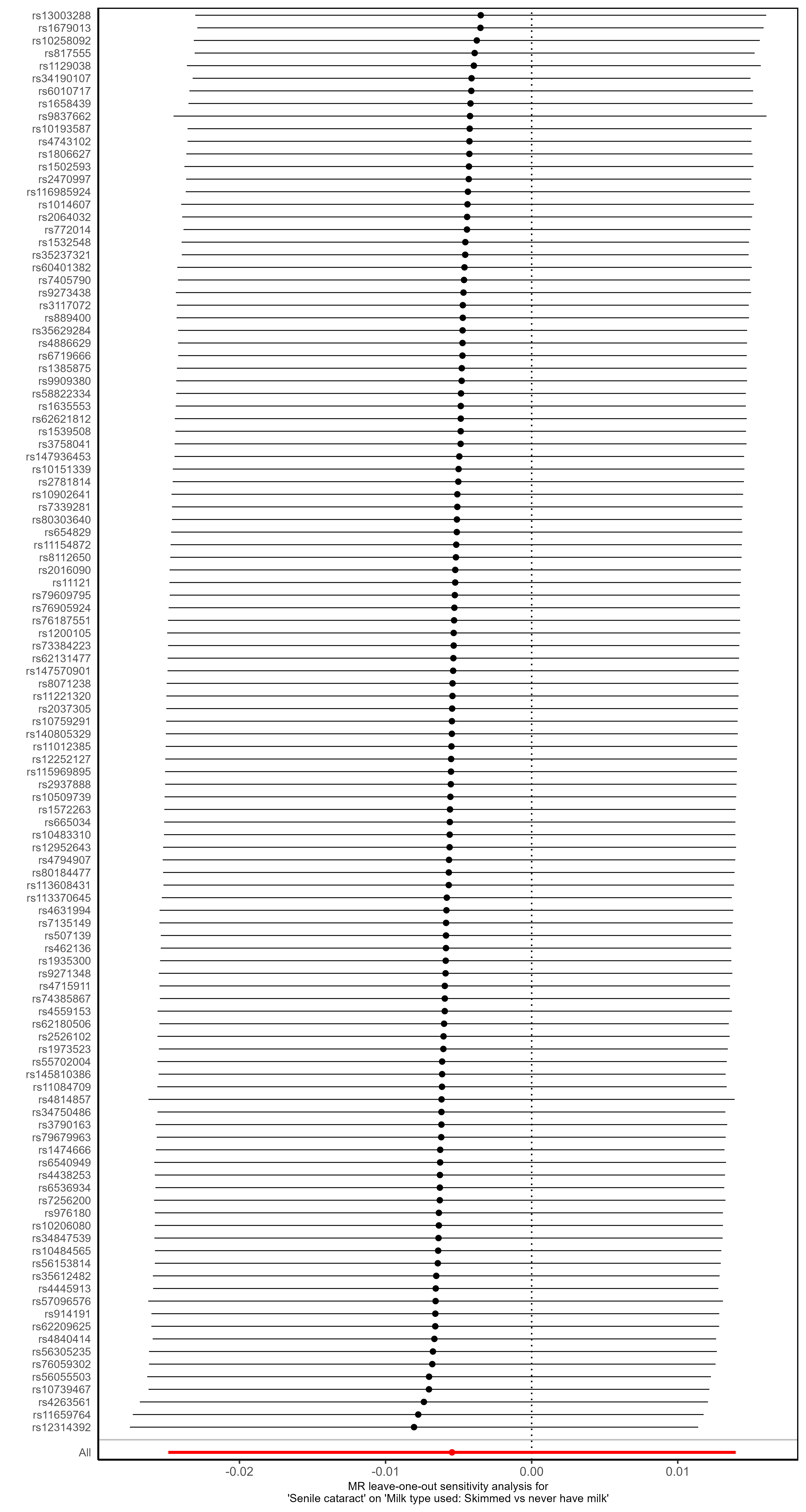


Figure S6.6 Leave-one-out analysis of SNPs associated with SC on Milk type used: skimmed vs never have milk.


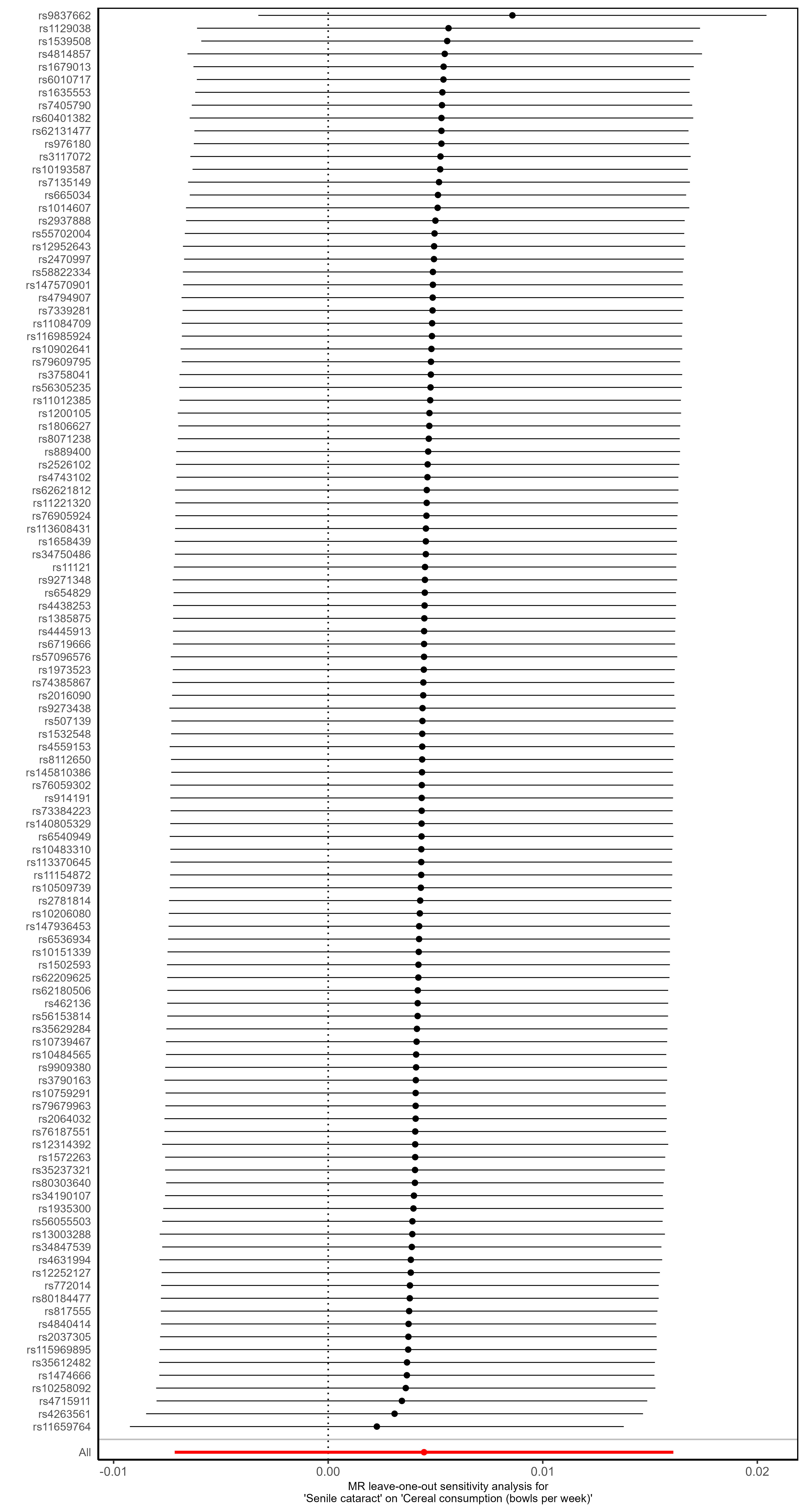


Figure S6.7 Leave-one-out analysis of SNPs associated with SC on Cereal consumption (bowls per week).


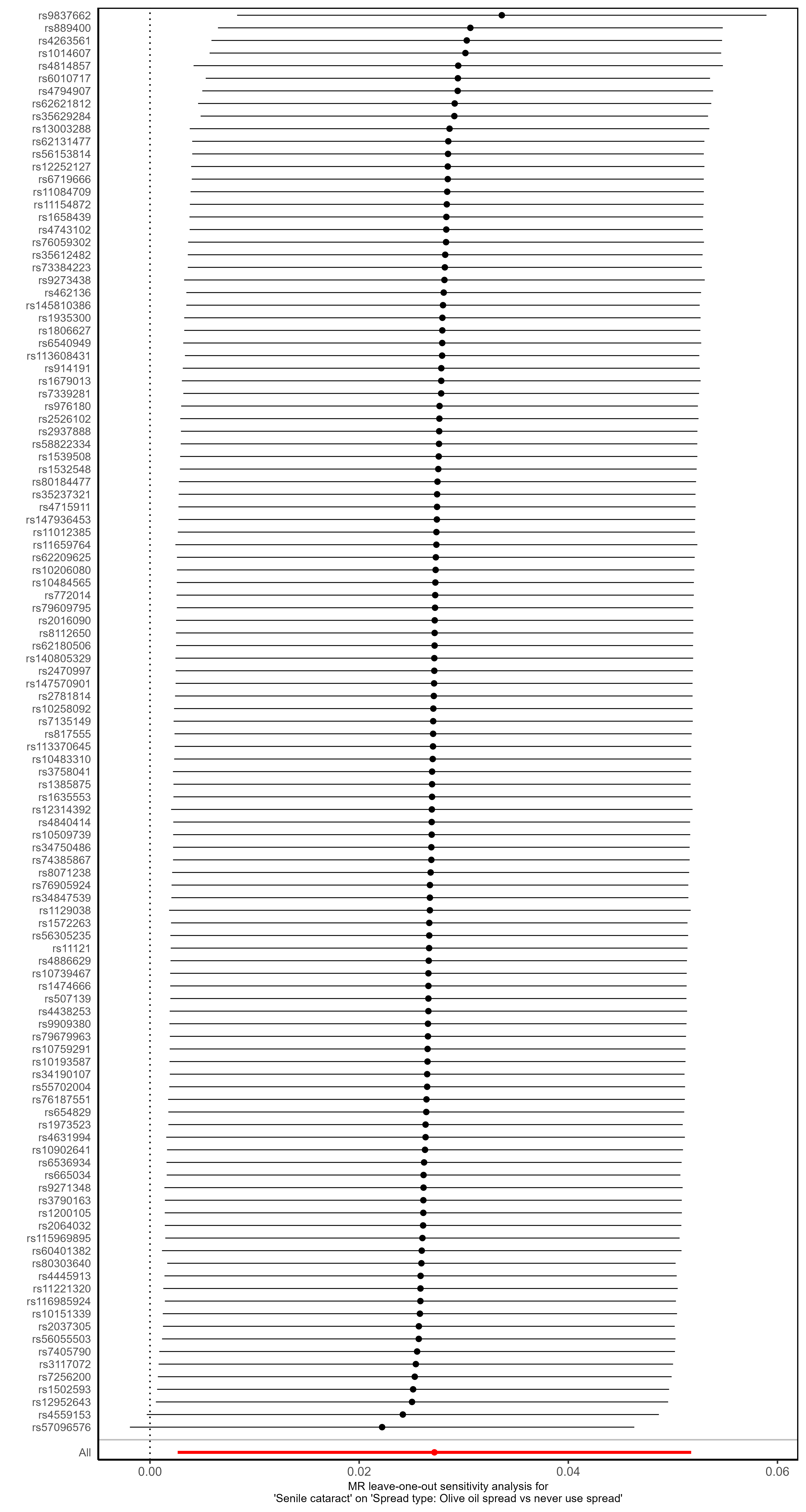


Figure S6.8 Leave-one-out analysis of SNPs associated with SC on Spread type: olive oil spread vs never use spread.


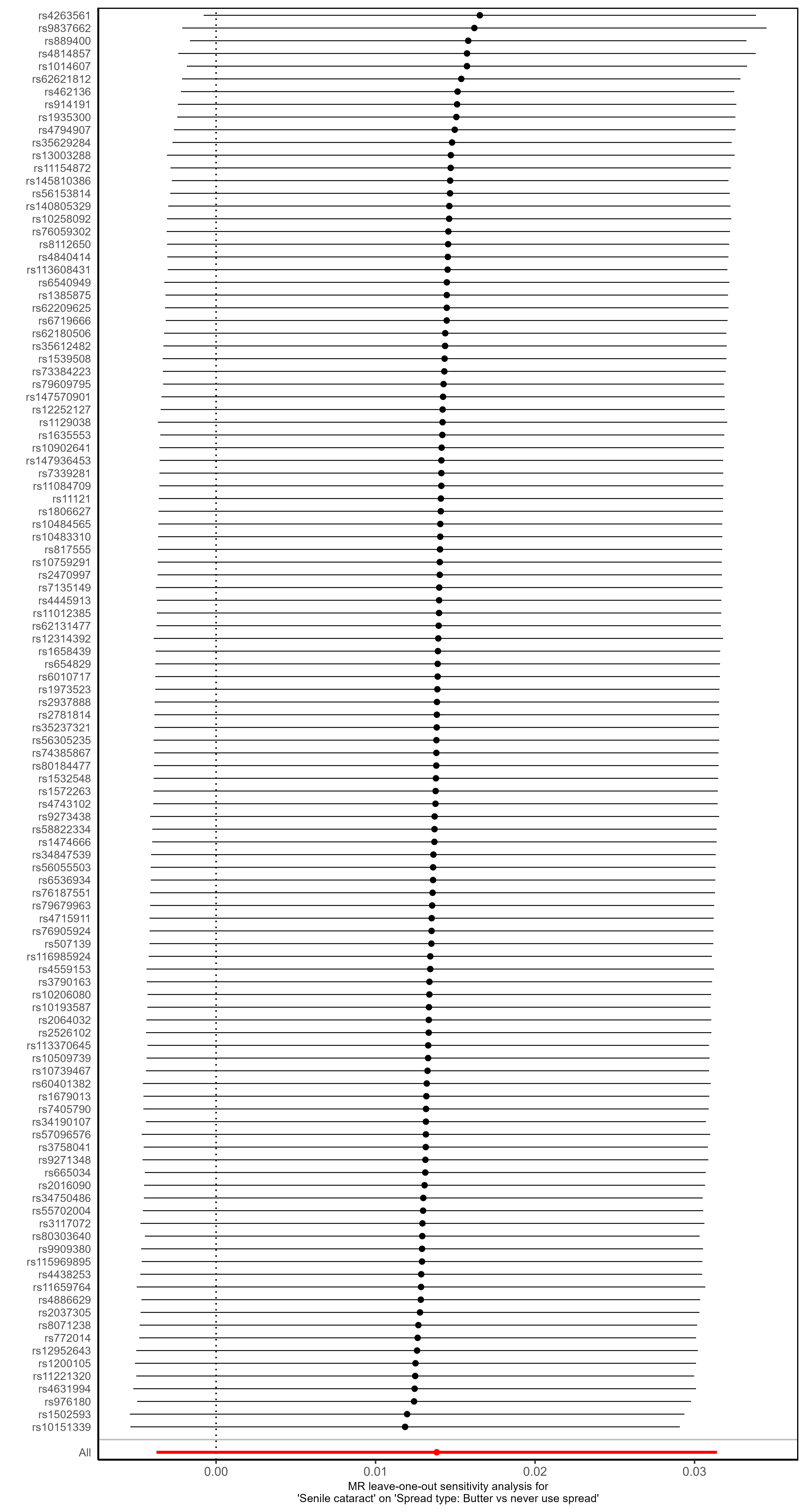


Figure S6.9 Leave-one-out analysis of SNPs associated with SC on Spread type: butter vs never use spread.


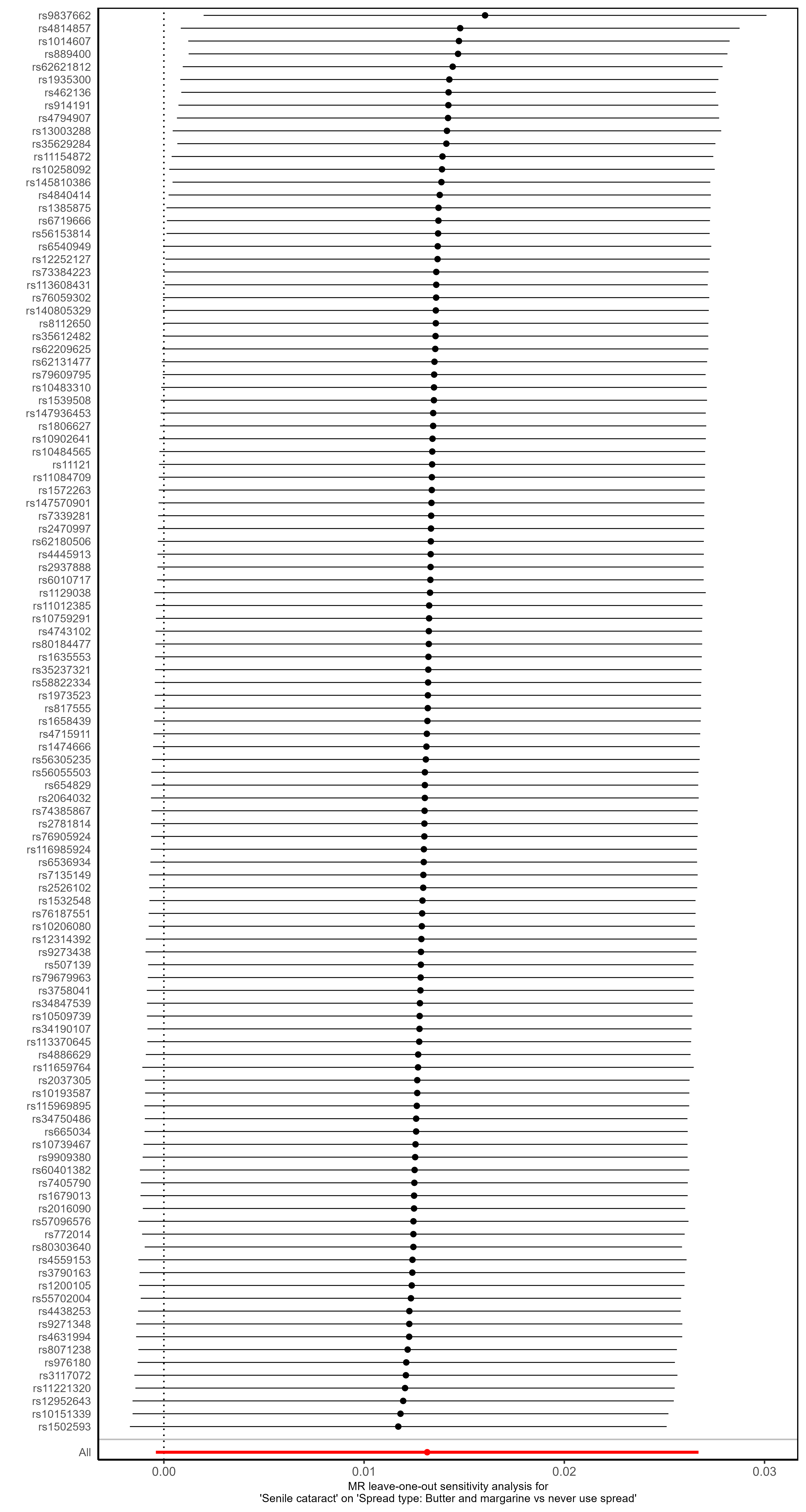


Figure S6.10 Leave-one-out analysis of SNPs associated with SC on Spread type: butter and margarine vs never use spread.


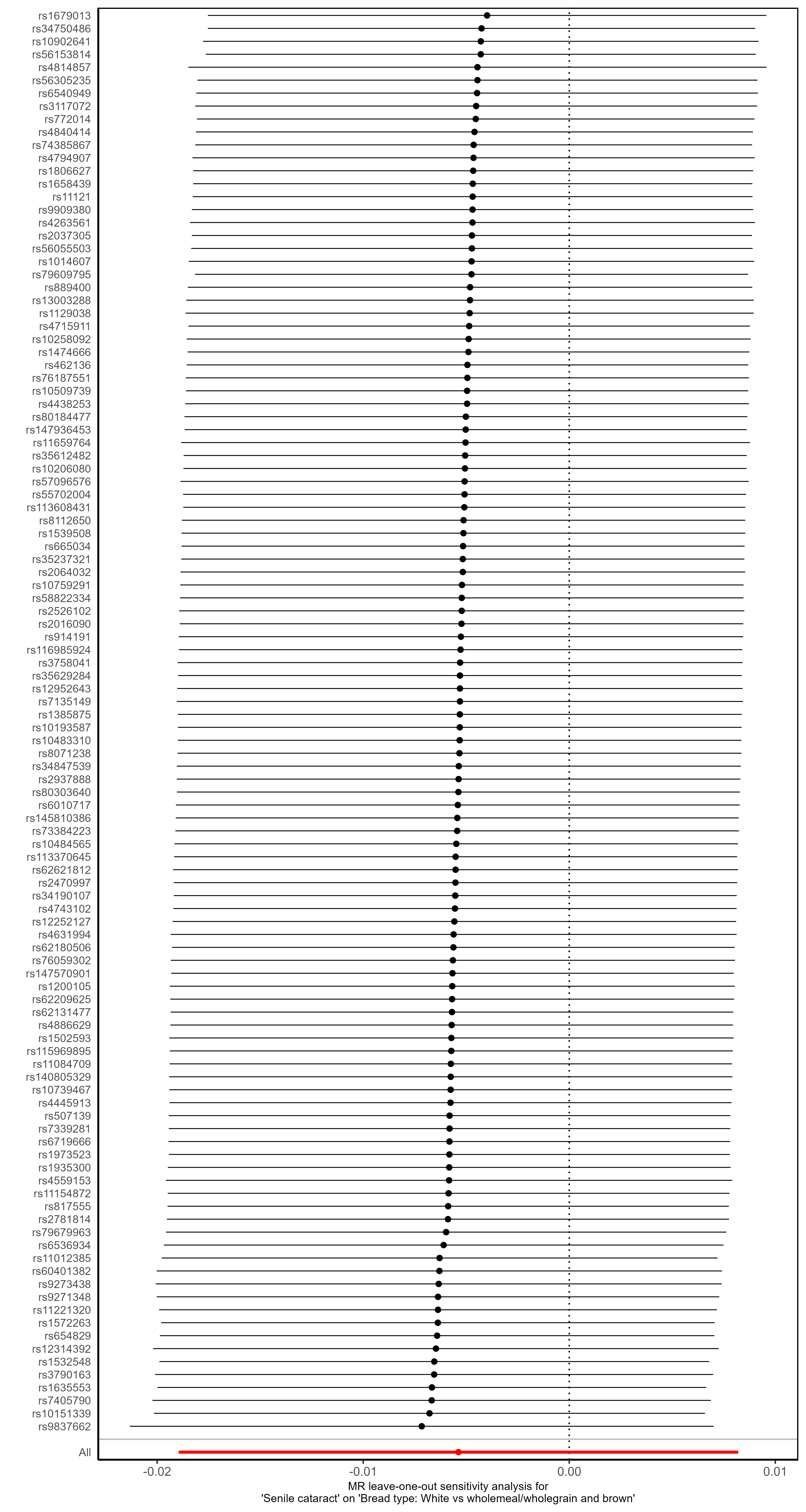


Figure S6.11 Leave-one-out analysis of SNPs associated with SC on Bread type: white vs wholemeal/wholegrain and brown.


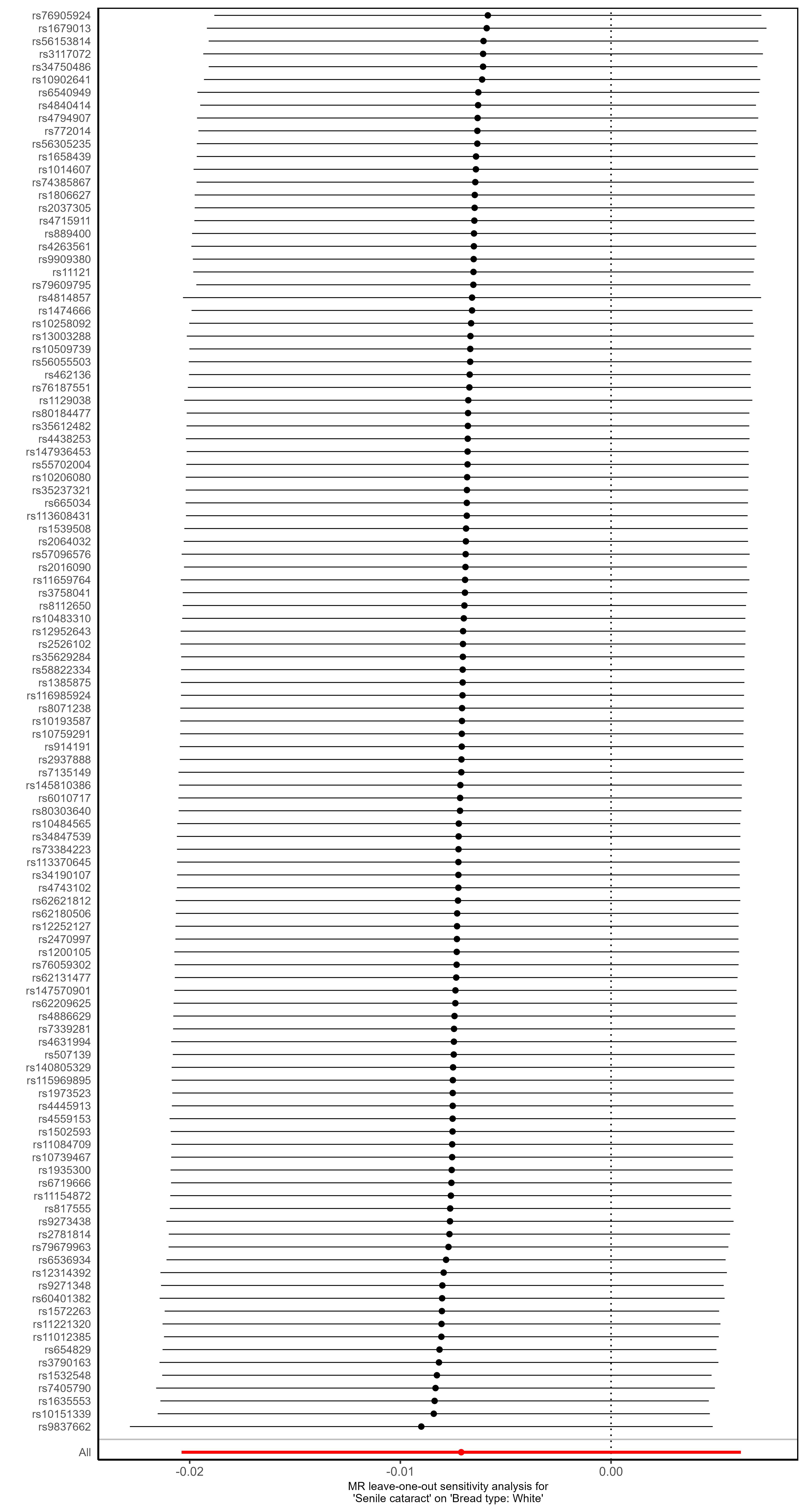


Figure S6.12 Leave-one-out analysis of SNPs associated with SC on bread type: white.


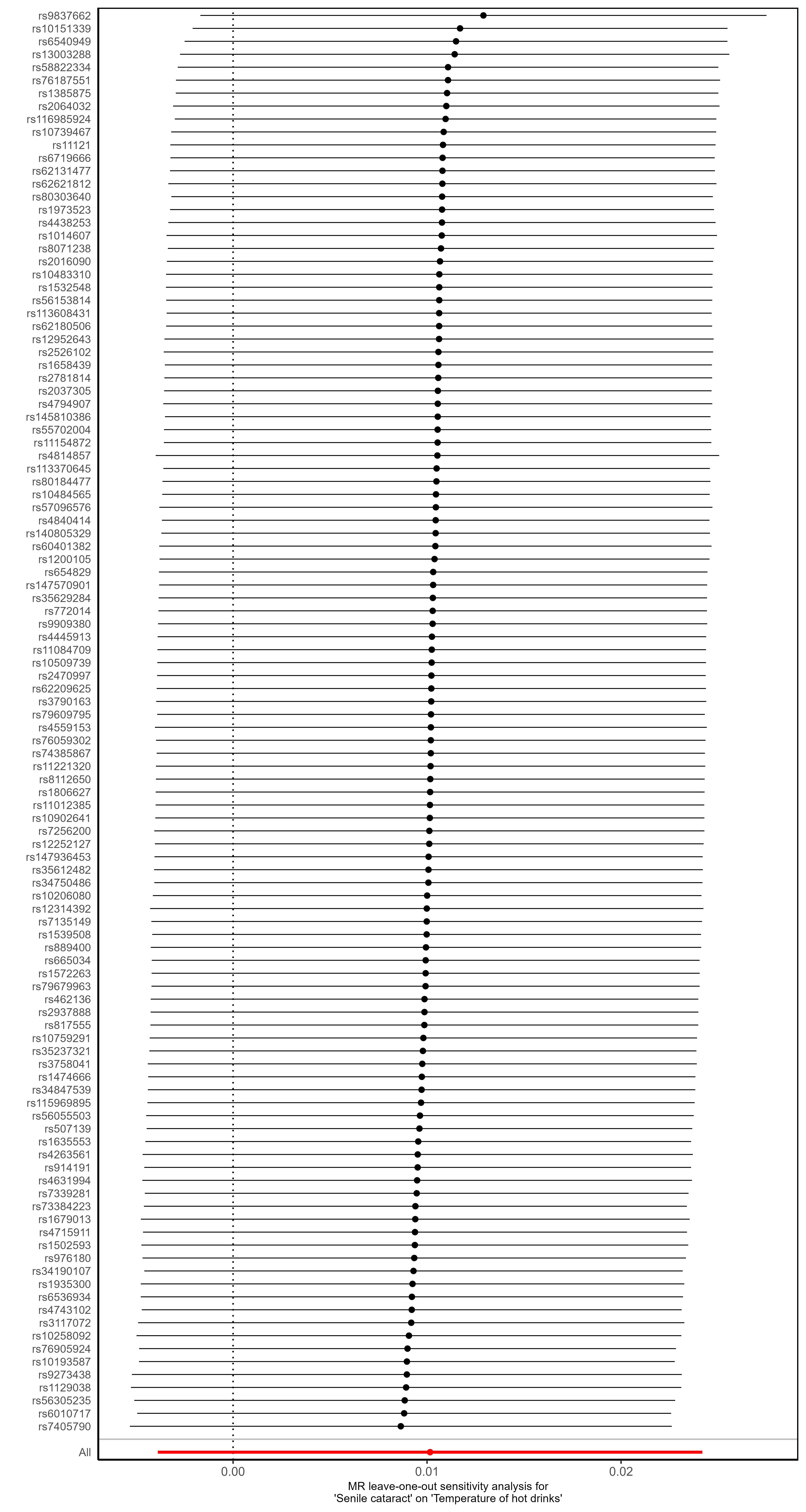


Figure S6.13 Leave-one-out analysis of SNPs associated with SC on Temperature of hot drinks.


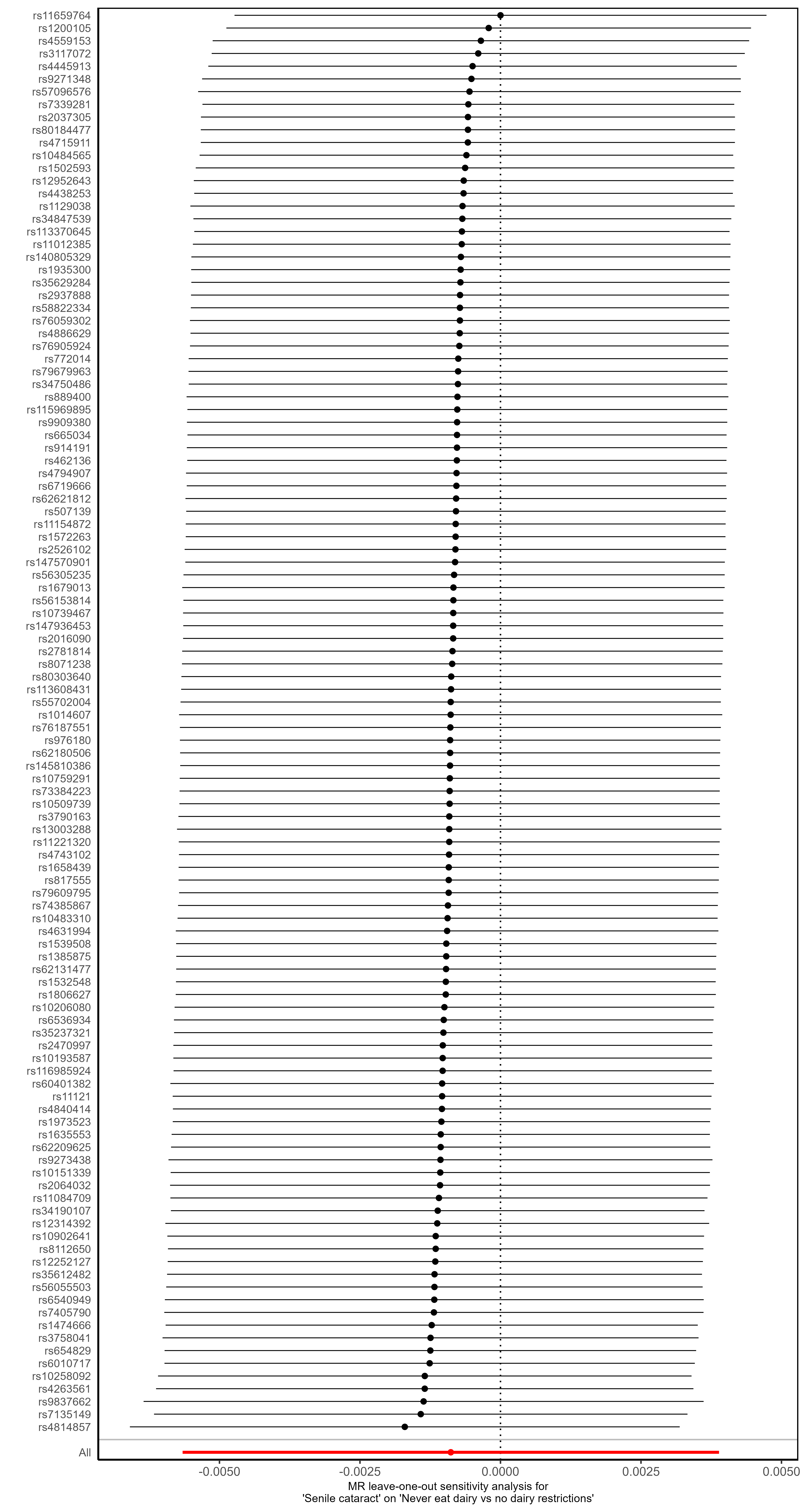


Figure S6.14 Leave-one-out analysis of SNPs associated with SC on Never eat dairy vs no dairy restrictions.


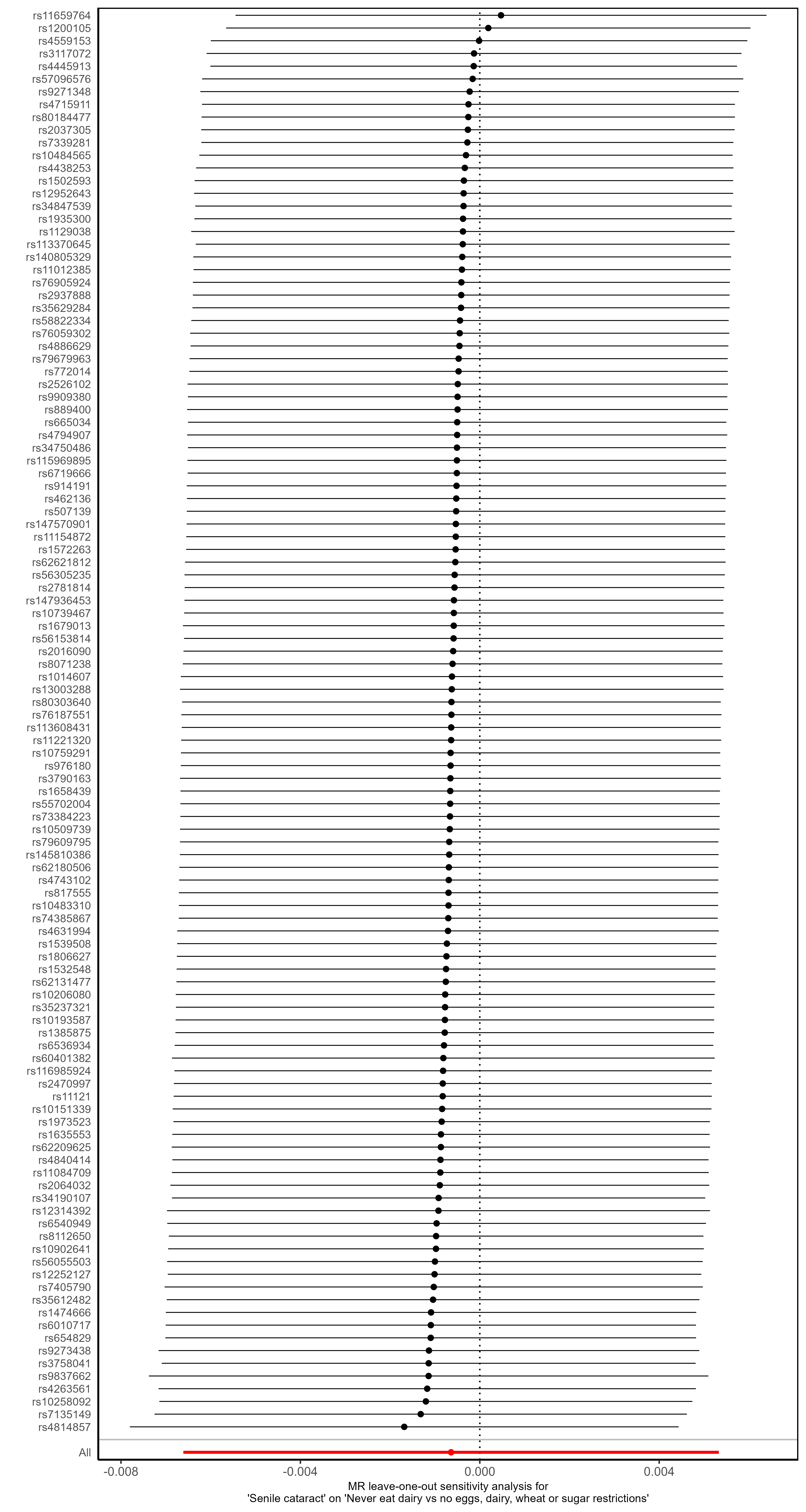


Figure S6.15 Leave-one-out analysis of SNPs associated with SC on Never eat dairy vs no eggs, dairy, wheat, or sugar restrictions.


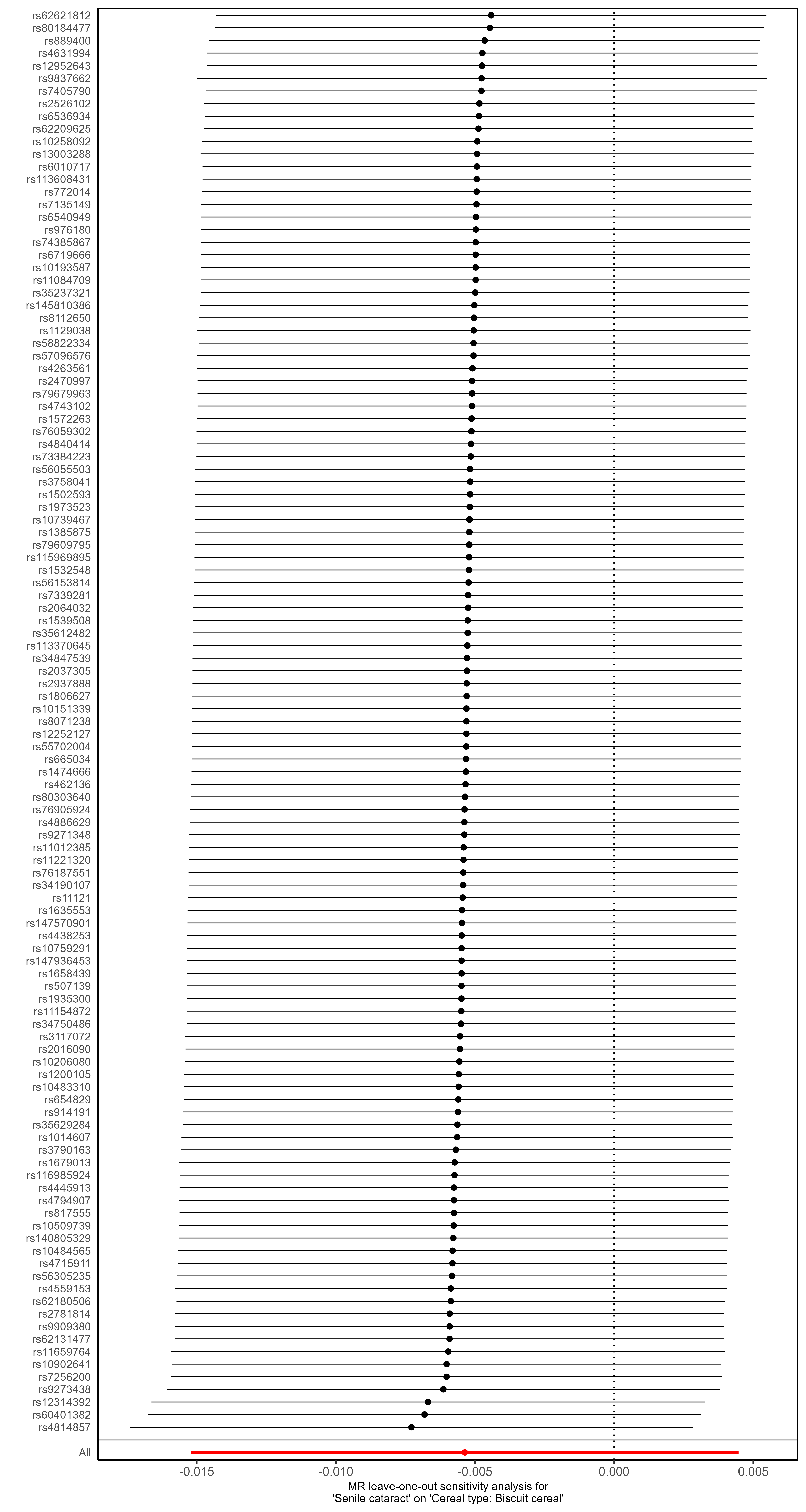


Figure S6.16 Leave-one-out analysis of SNPs associated with SC on Cereal type: biscuit cereal.

**Figure S7.** Scatter plots for the association between 13 significant dietary habits and SC in the replication sample MR analysis.


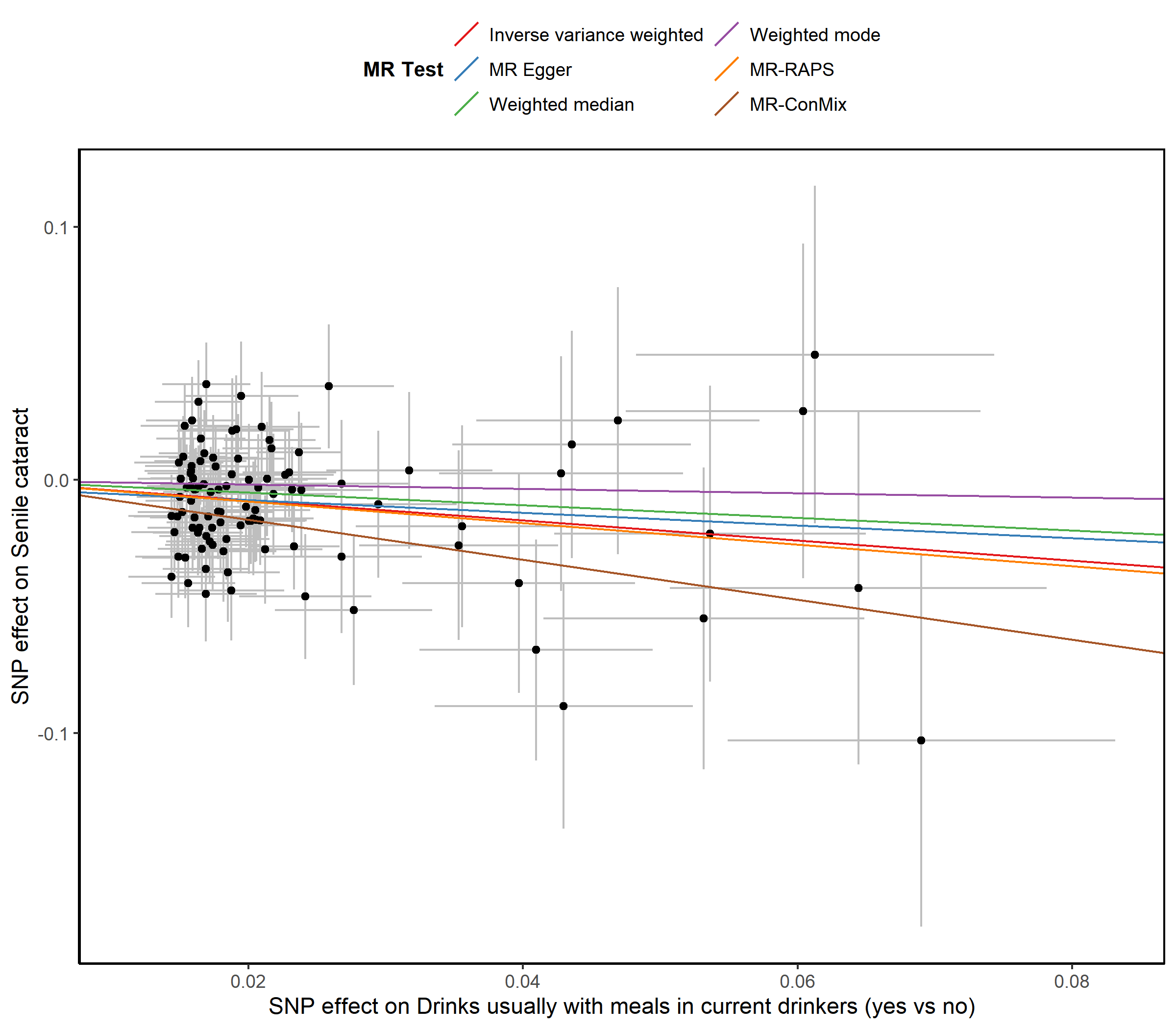


Figure S7.1 Scatter plot of SNPs associated with Drinks usually with meals in current drinkers (yes vs no) on SC.


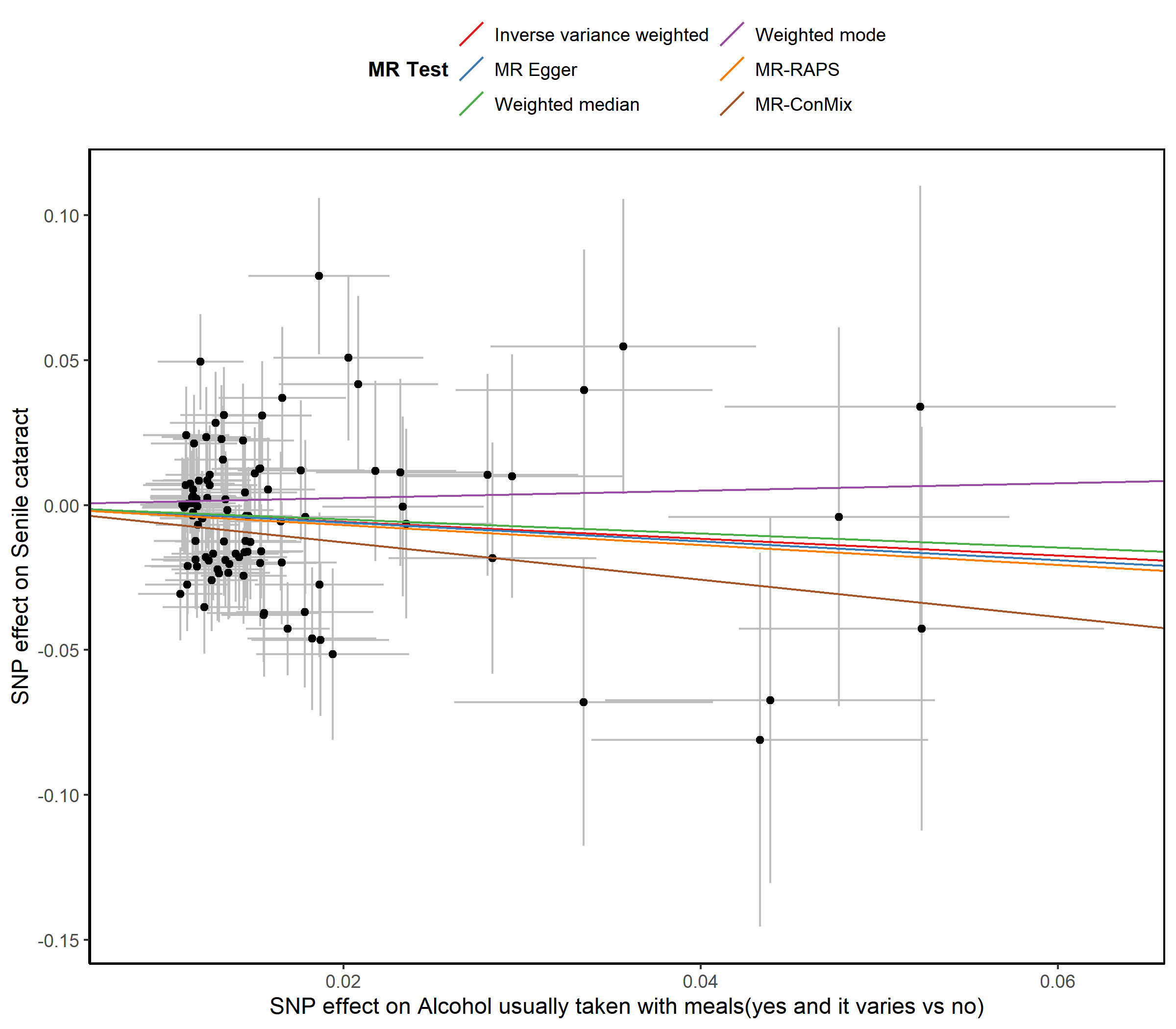


Figure S7.2 Scatter plot of SNPs associated with Alcohol usually taken with meals (yes and it varies vs no) on SC.


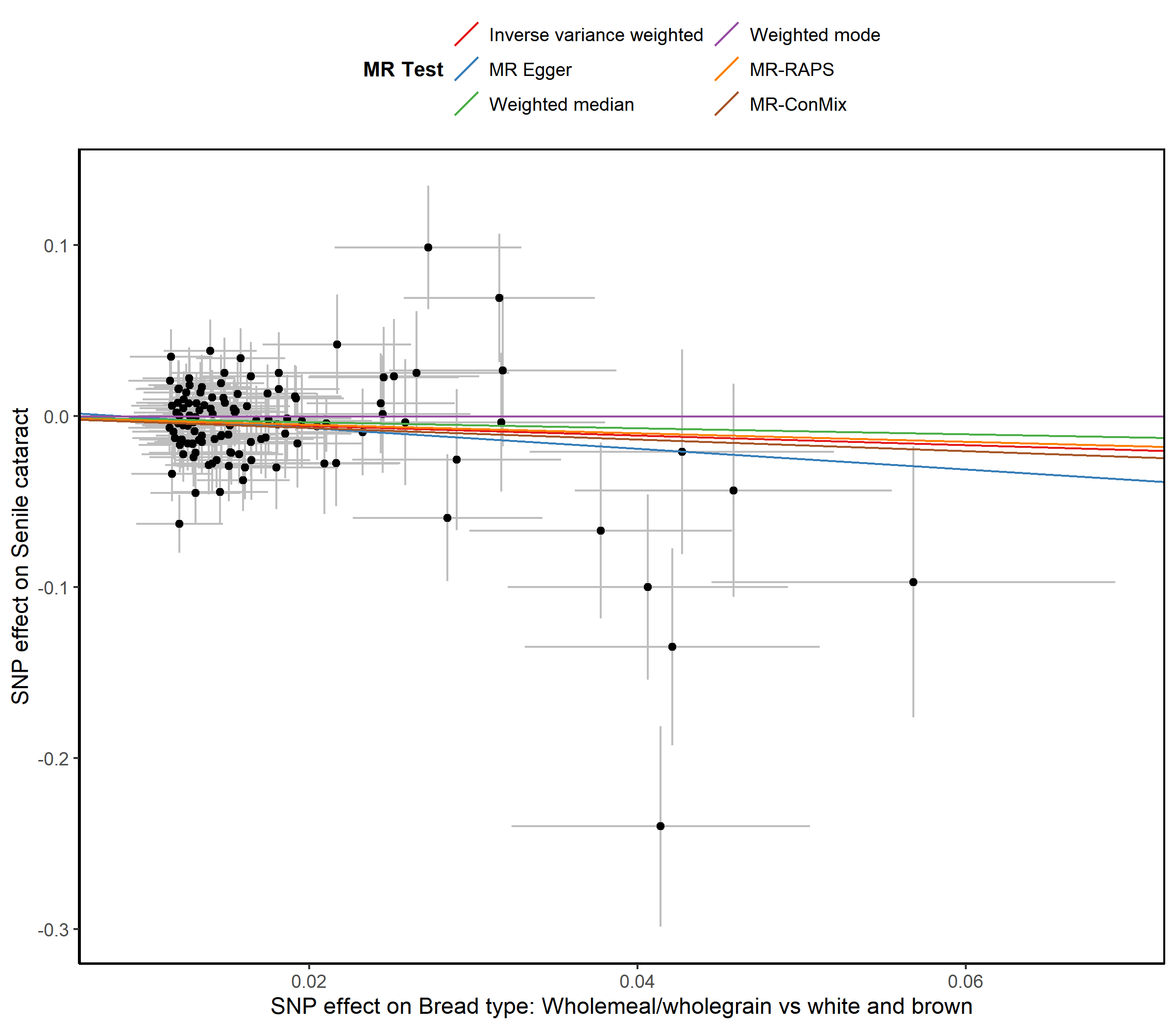


Figure S7.3 Scatter plot of SNPs associated with Bread type: wholemeal/wholegrain vs white and brown on SC.


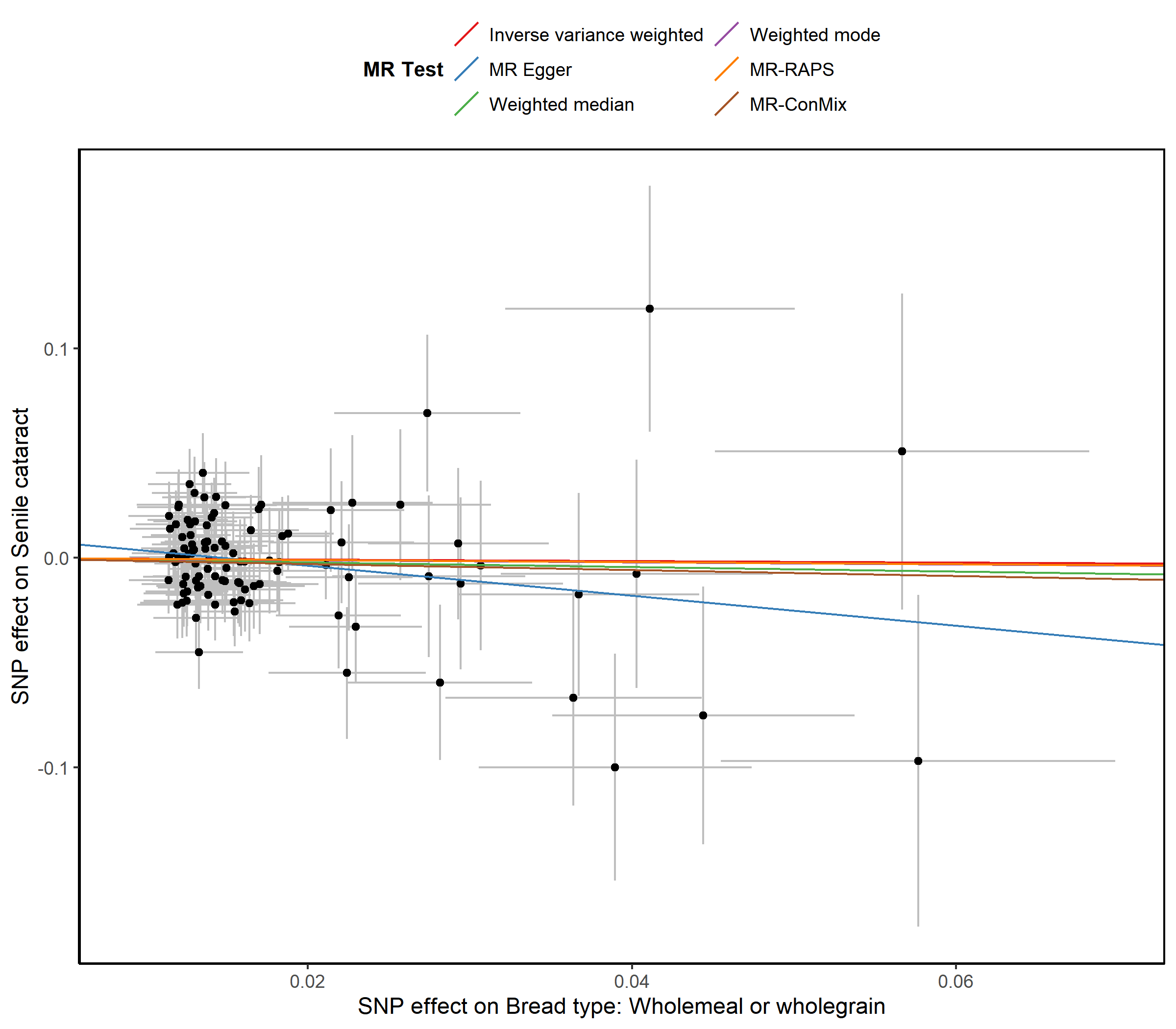


Figure S7.4 Scatter plot of SNPs associated with Bread type: wholemeal or wholegrain on SC.


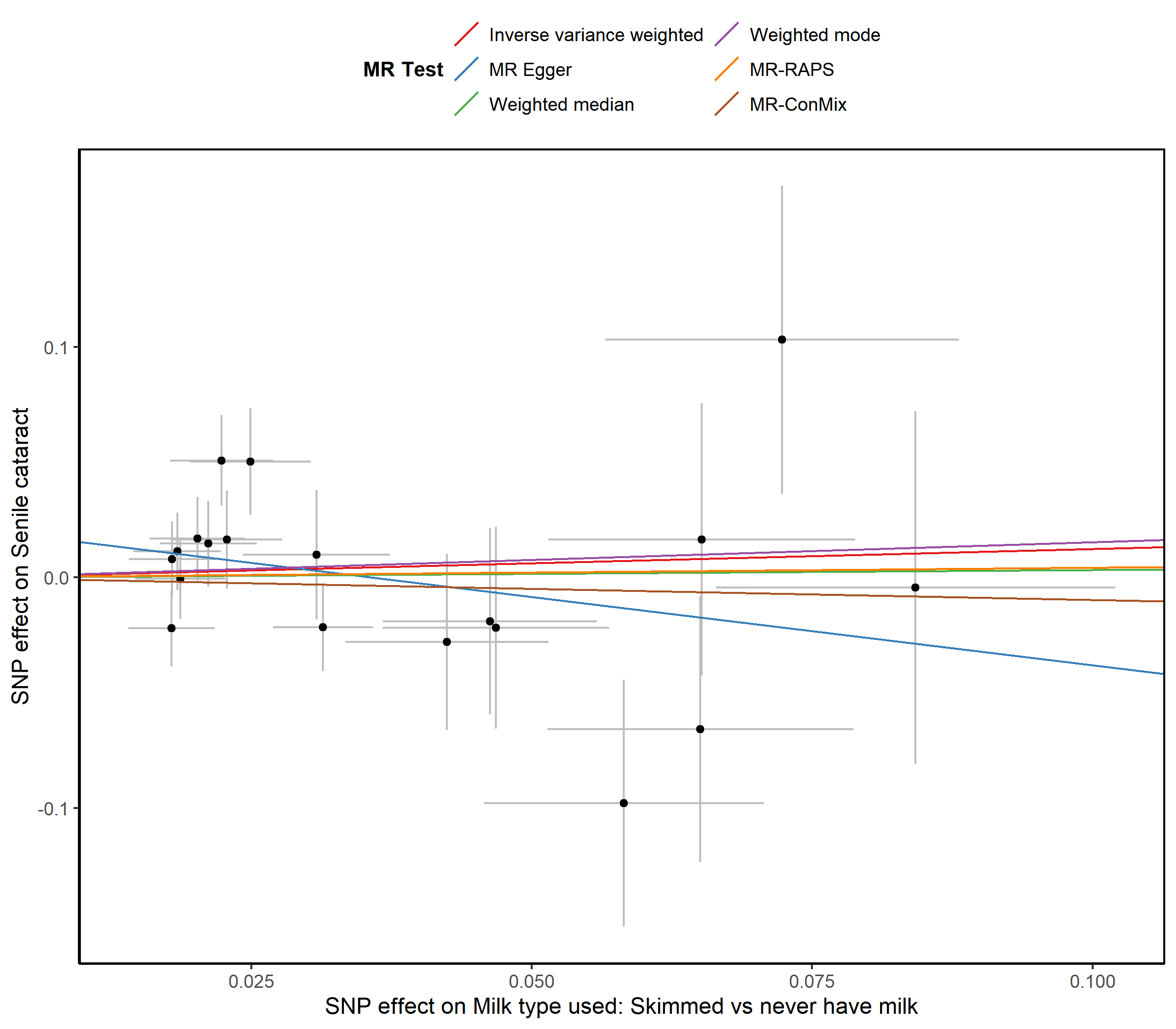


Figure S7.5 Scatter plot of SNPs associated with Milk type used: skimmed vs never have milk on SC.

Figure S7.6 Scatter plot of SNPs associated with Cereal consumption (bowls per week) on SC.

Figure S7.7 Scatter plot of SNPs associated with Spread type: butter and margarine vs never use spread on SC.

Figure S7.8 Scatter plot of SNPs associated with Bread type: white vs wholemeal/wholegrain and brown on SC.

Figure S7.9 Scatter plot of SNPs associated with Bread type: white on SC.

Figure S7.10 Scatter plot of SNPs associated with Temperature of hot drinks on SC.

Figure S7.11 Scatter plot of SNPs associated with Never eat dairy vs no dairy restrictions on SC.

Figure S7.12 Scatter plot of SNPs associated with Never eat dairy vs no eggs, dairy, wheat, or sugar restrictions on SC.

Figure S7.13 Scatter plot of SNPs associated with Cereal type: biscuit cereal on SC.

**Figure S8.** Funnel plot for the association between 13 significant dietary habits and SC in the replication sample MR analysis.

Figure S8.1 Funnel plot of SNPs associated with Drinks usually with meals in current drinkers (yes vs no) on SC.

Figure S8.2 Funnel plot of SNPs associated with Alcohol usually taken with meals (yes and it varies vs no) on SC.

Figure S8.3 Funnel plot of SNPs associated with Bread type: wholemeal/wholegrain vs white and brown on SC.

Figure S8.4 Funnel plot of SNPs associated with Bread type: wholemeal or wholegrain on SC.

Figure S8.5 Funnel plot of SNPs associated with Milk type used: skimmed vs never have milk on SC.

Figure S8.6 Funnel plot of SNPs associated with Cereal consumption (bowls per week) on SC.

Figure S8.7 Funnel plot of SNPs associated with Spread type: butter and margarine vs never use spread on SC.

Figure S8.8 Funnel plot of SNPs associated with Bread type: white vs wholemeal/wholegrain and brown on SC.

Figure S8.9 Funnel plot of SNPs associated with Bread type: white on SC.

Figure S8.10 Funnel plot of SNPs associated with Temperature of hot drinks on SC.

Figure S8.11 Funnel plot of SNPs associated with Never eat dairy vs no dairy restrictions on SC.

Figure S8.12 Funnel plot of SNPs associated with Never eat dairy vs no eggs, dairy, wheat, or sugar restrictions on SC.

Figure S8.13 Funnel plot of SNPs associated with Cereal type: biscuit cereal on SC.

**Figure S9.** Leave-one-out analysis for the association between 13 significant dietary habits and SC in the replication sample MR analysis.

Figure S9.1 Leave-one-out analysis of SNPs associated with Drinks usually with meals in current drinkers on SC.

Figure S9.2 Leave-one-out analysis of SNPs associated with Alcohol usually taken with meals (yes and it varies vs no) on SC.

Figure S9.3 Leave-one-out analysis of SNPs associated with Bread type: wholemeal/wholegrain vs white and brown on SC.

Figure S9.4 Leave-one-out analysis of SNPs associated with Bread type: wholemeal or wholegrain on SC.

Figure S9.5 Leave-one-out analysis of SNPs associated with Milk type used: skimmed vs never have milk on SC.

Figure S9.6 Leave-one-out analysis of SNPs associated with Cereal consumption (bowls per week) on SC.

Figure S9.7 Leave-one-out analysis of SNPs associated with Spread type: butter and margarine vs never use spread on SC.

Figure S9.8 Leave-one-out analysis of SNPs associated with Bread type: white vs wholemeal/wholegrain and brown on SC.

Figure S9.9 Leave-one-out analysis of SNPs associated with Bread type: white on SC.

Figure S9.10 Leave-one-out analysis of SNPs associated with Temperature of hot drinks on SC.

Figure S9.11 Leave-one-out analysis of SNPs associated with Never eat dairy vs no dairy restrictions on SC.

Figure S9.12 Leave-one-out analysis of SNPs associated with Never eat dairy vs no eggs, dairy, wheat, or sugar restrictions on SC.

Figure S9.13 Leave-one-out analysis of SNPs associated with Cereal type: biscuit cereal on SC.

**Figure S10.** MR results of Drinks usually with meals in current drinkers (yes vs no) on Cataract.

Figure S10.1 Scatter plot of SNPs associated with Drinks usually with meals in current drinkers (yes vs no) on Cataract.

Figure S10.2 Funnel plot of SNPs associated with Drinks usually with meals in current drinkers (yes vs no) on Cataract.

Figure S10.3 Leave-one-out analysis of SNPs associated with Drinks usually with meals in current drinkers (yes vs no) on Cataract.

Figure S10.4 Scatter plot of SNPs associated with Cataract on Drinks usually with meals in current drinkers (yes vs no).

Figure S10.5 Funnel plot of SNPs associated with Cataract on Drinks usually with meals in current drinkers (yes vs no).

Figure S10.6 Leave-one-out analysis of SNPs associated with Cataract on Drinks usually with meals in current drinkers (yes vs no).

**Figure S11.** Bidirectional MR results of Drinks usually with meals in current drinkers (yes vs no) on Cataracts operation.

Figure S11.1 Scatter plot of SNPs associated with Drinks usually with meals in current drinkers (yes vs no) on Cataracts operation.

Figure S11.2 Funnel plot of SNPs associated with Drinks usually with meals in current drinkers (yes vs no) on Cataracts operation.

Figure S11.3 Leave-one-out analysis of SNPs associated with Drinks usually with meals in current drinkers (yes vs no) on Cataracts operation.

Figure S11.4 Scatter plot of SNPs associated with Cataracts operation on Drinks usually with meals in current drinkers (yes vs no).

Figure S11.5 Funnel plot of SNPs associated with Cataracts operation on Drinks usually with meals in current drinkers (yes vs no).

Figure S11.6 Leave-one-out analysis of SNPs associated with Cataracts operation on Drinks usually with meals in current drinkers (yes vs no).

Figure S12. Results of the forward MR analysis. *nSNP* the number of SNPs, *IVW* inverse variance weighted, *BWMR* Bayesian weighted MR, *RAPS* robust adjusted profile score, *Con-Mix* contamination mixture.

Figure S13. Results of the reverse MR analysis. nSNP the number of SNPs, IVW inverse variance weighted, BWMR Bayesian weighted MR, RAPS robust adjusted profile score, Con-Mix contamination mixture.

Figure S14. Results of the replication MR analysis. nSNP the number of SNPs, IVW inverse variance weighted, BWMR Bayesian weighted MR, RAPS robust adjusted profile score, Con-Mix contamination mixture.
