# Supplementary material for: Modifiable Dietary Habits, Inflammatory Mediation, and Senile Cataract: Unraveling Causality via Mendelian Randomization
Source: Food Sci Nutr. 2025 Dec 17;13(12):e71366. doi: 10.1002/fsn3.71366 (PMC12710447; doi:10.1002/fsn3.71366)
Supplement: Supplementary file 4 — Data S4: fsn371366‐sup‐0004‐Supinfo2.docx. [file FSN3-13-e71366-s003.docx]

**Supplementary MR analysis methods**

**MR Egger regression**

The MR-Egger method assumes that the distribution of the direct impact of candidate IVs on the outcome is independent of the distribution associated with the exposure factor, referred to as the "Instrument Strength Independent of Direct Effect (InSIDE)" assumption(Bowden, Davey Smith, & Burgess, 2015). This is achieved through a regression analysis of the precision-weighted IVs estimates related to the exposure factor against the outcomes associated with the IVs. The intercept of the regression can be interpreted as an estimate of the average pleiotropic effect of the IVs. Even when all IVs are ineffective due to a violation of the third assumption of MR analysis, the regression slope can still provide an estimate of the causal effect.

**Weighted median**

The weighted median method ranks MR estimates for each IV based on its precision-weighted magnitude and generates an overall MR estimate using the median value(Bowden, Davey Smith, Haycock, & Burgess, 2016). It can offer a robust causal relationship analysis even in situations where up to 50% of instrumental variables are invalid.

**Weighted mode**

The weighted mode method offers a consistent estimator when instrumental variable assumptions are less stringent, resulting in reduced bias and a lower type I error rate(Hartwig, Davey Smith, & Bowden, 2017).

**Bayesian weighted Mendelian randomization**

Bayesian weighted Mendelian randomization (BWMR) integrates Bayesian inference with MR to address confounding factors by combining prior knowledge with observational data, thereby enabling more accurate estimation of causal relationships. Furthermore, it can account for uncertainties in estimating weak effects and weak horizontal pleiotropic effects, as well as adaptively detect outliers caused by certain strong horizontal pleiotropic effects(Zhao et al., 2020).

**Robust adjusted profile score**

The Robust adjusted profile score (MR-RAPS) method builds a model for the pleiotropic effects of instrumental variables directly using a random-effects distribution. It assumes that the pleiotropic effects are centered around zero and follow a normal distribution with an unknown variance. The estimate of the causal effect is obtained by fitting the profile-likelihood function to the variances of both the causal effect and pleiotropic effects distributions. MR-RAPS allows for the inclusion of some weak instrumental variables and provides robust statistical estimates for Mendelian Randomization by accounting for these weak instruments(Slob & Burgess, 2020).

**Contamination mixture**

The contamination mixture method (ConMix) is implemented by constructing a likelihood function based on variant-specific causal estimates. For each IV, an estimate of the causal effect can be obtained by dividing the genetic association with the outcome by the genetic association with the exposure. ConMix categorizes instrumental variables into effective and ineffective groups. Under the assumption that there is a single causal effect of the exposure on the outcome, the method can robustly and efficiently estimate this effect, even when some genetic variants are not valid IVs(Burgess, Foley, Allara, Staley, & Howson, 2020).

**Reference**

Bowden, J., G. Davey Smith, & S. Burgess. 2015. "Mendelian Randomization with Invalid Instruments: Effect Estimation and Bias Detection through Egger Regression." *International Journal of Epidemiology* 44, no. 2: 512-525. doi:10.1093/ije/dyv080

Bowden, J., G. Davey Smith, P. C. Haycock, & S. Burgess. 2016. "Consistent Estimation in Mendelian Randomization with Some Invalid Instruments Using a Weighted Median Estimator." *Genetic Epidemiology* 40, no. 4: 304-314. doi:10.1002/gepi.21965

Burgess, S., C. N. Foley, E. Allara, et al. 2020. "A Robust and Efficient Method for Mendelian Randomization with Hundreds of Genetic Variants." *Nature Communications* 11, no. 1: 376. doi:10.1038/s41467-019-14156-4

Hartwig, F. P., G. Davey Smith, & J. Bowden. 2017. "Robust Inference in Summary Data Mendelian Randomization Via the Zero Modal Pleiotropy Assumption." *International Journal of Epidemiology* 46, no. 6: 1985-1998. doi:10.1093/ije/dyx102

Slob, E. A. W., & S. Burgess. 2020. "A Comparison of Robust Mendelian Randomization Methods Using Summary Data." *Genetic Epidemiology* 44, no. 4: 313-329. doi:10.1002/gepi.22295

Zhao, J., J. Ming, X. Hu, et al. 2020. "Bayesian Weighted Mendelian Randomization for Causal Inference Based on Summary Statistics." *Bioinformatics* 36, no. 5: 1501-1508. doi:10.1093/bioinformatics/btz749
